# Associations of cardiovascular risk factors and lifestyle behaviors with neurodegenerative disease: a Mendelian randomization study

**DOI:** 10.1038/s41398-023-02553-9

**Published:** 2023-07-24

**Authors:** Liang-Yu Huang, Ya-Nan Ou, Yu-Xiang Yang, Zuo-Teng Wang, Lan Tan, Jin-Tai Yu

**Affiliations:** 1 Department of Neurology, Qingdao Municipal Hospital, Qingdao University, Qingdao, China; 2 Department of Neurology and Institute of Neurology, Huashan Hospital, State Key Laboratory of Medical Neurobiology and MOE Frontier Center for Brain Science, Shanghai Medical College, Fudan University, Shanghai, China

**Keywords:** Psychiatric disorders, Clinical genetics

## Abstract

Previous observational studies reported that midlife clustering of cardiovascular risk factors and lifestyle behaviors were associated with neurodegenerative disease; however, these findings might be biased by confounding and reverse causality. This study aimed to investigate the causal associations of cardiovascular risk factors and lifestyle behaviors with neurodegenerative disease, using the two-sample Mendelian randomization design. Genetic variants for the modifiable risk factors and neurodegenerative disease were extracted from large-scale genome-wide association studies. The inverse-variance weighted method was used as the main analysis method, and MR-Egger regression and leave-one-out analyses were performed to identify potential violations. Genetically predicted diastolic blood pressure (DBP: OR per 1 mmHg, 0.990 [0.979–1.000]), body mass index (BMI: OR per 1 SD, 0.880 [0.825–0.939]), and educational level (OR per 1 SD, 0.698 [0.602–0.810]) were associated with lower risk of late-onset Alzheimer’s disease (LOAD), while genetically predicted low-density lipoprotein (LDL: OR per 1 SD, 1.302 [1.066–1.590]) might increase LOAD risk. Genetically predicted exposures (including LDL and BMI) applied to familial AD showed the same effect. The association of LDL was also found with Amyotrophic lateral sclerosis (ALS) (LDL: OR per 1 SD, 1.180 [1.080–1.289]). This MR analysis showed that LDL, BMI, BP, and educational level were causally related to AD; a significant association between LDL and ALS risk, as well as the potential effect of sleep duration on PD risk, were also revealed. Targeting these modifiable factors was a promising strategy of neurodegenerative disease prevention.

## Introduction

There is an increasing prevalence of neurodegenerative disease in the global aging population [1], which places a major economic burden on healthcare services. In addition to age, certain environmental or lifestyle factors combined with genetic factors increase the risk of neurodegenerative disease [2]. Since neurodegenerative disease is incurable, a preventive strategy targeting its risk factors is warranted.

Previous epidemiological studies and meta-analyses identified potentially modifiable risk factors that could be targeted in preventive measures to reduce the incidence of Alzheimer’s disease (AD), the most common neurodegenerative disease [3]. These factors consist of cardiovascular risk factors, such as metabolic diseases [4] and blood pressure [5], lifestyle behaviors including smoking [6], drinking [7], and sleeping [6, 8], as well as an educational level [6]. Other neurodegenerative diseases, including Parkinson’s disease (PD) and amyotrophic lateral sclerosis (ALS), were reported as sharing some common contributing factors with AD, such as smoking, drinking, physical activity, body mass index (BMI), blood pressure (BP), and so on [9]. Thus, modifying cardiovascular risk factors and lifestyle behaviors may hold promise for reducing the burden of neurodegenerative disease. However, findings from observational studies may be influenced by reverse causation bias and unmeasured confounding factors that can obscure the true association. Besides, difficulties in conducting large-scale randomized clinical trials (RCTs) also restrict the exploration of this association. These limitations call for the use of alternative methods, for instance, those that target causal effects and use complementary data sources, rather than single cohorts.

Mendelian randomization (MR) is a novel method that can estimate the causal effects of risk factors in observational studies by using genetic variants to provide evidence of robust associations and incorporating summary statistics from large-cohort genome-wide association studies (GWASs) [10]. Previous studies using MR have explored the causal effects of some modified lifestyle behaviors, such as sleep characteristics, on AD [11]. However, they reached inconsistent results, possibly due to the differences in the number of single nucleotide polymorphisms (SNPs) included, the statistical power, collider bias, and so on; thereby, higher-quality studies with larger sample sizes are urgently needed to resolve the issues. A recent study explored the associations between midlife vascular risk factors and the risk of incident dementia [12], but it only adopted all-cause dementia as the outcome since AD and vascular dementia may not share the same risk factors.

Herein, we aimed to perform a two-sample MR analysis to assess the causal effects of genetically determined cardiovascular risk factors and lifestyle behaviors on the risk of LOAD as well as familial AD (including paternal AD and maternal AD) comprehensively. In spite of the diversity of clinical symptoms of neurodegenerative disease, their pathogenesis was linked by: the misfolding of proteins in specific brain regions; significant neuroinflammation and excessive oxidative stress in those areas [13]. To be a comparison, we also evaluated the causal association of cardiovascular risk factors and lifestyle behaviors with the risk of other two neurodegenerative diseases, including PD and ALS.

## Methods

### Two-sample MR design

**Fig. 1:**
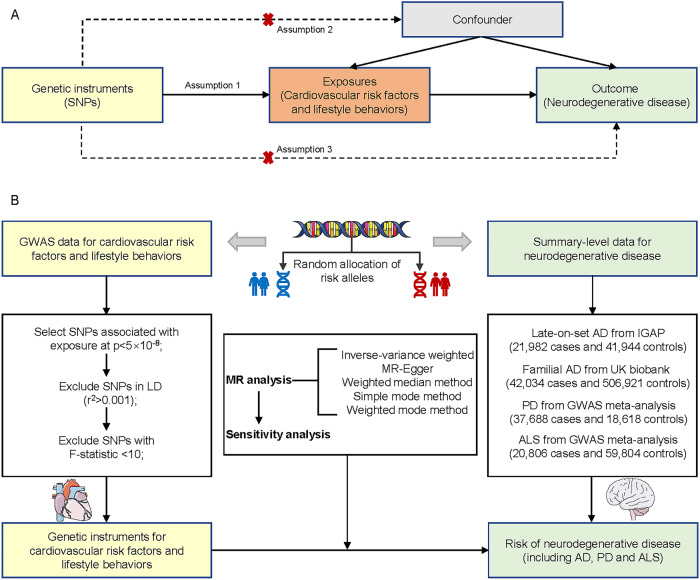
Study overview. **A** Schematic diagram of an MR analysis. Since genetic alleles are independently segregated and randomly assigned, SNPs are not associated with confounding factors that may bias estimates from observational studies. Three assumptions of MR are as follows: (1) the selected instrument is predictive of the exposure, (2) the instrument is independent of confounding factors, and (3) there is no horizontal pleiotropy (the instrument is associated with the outcome only through the exposure). **B** The study design overview of the current study. MR Mendelian randomization, GWAS genome-wide association studies, AD Alzheimer’s disease, PD Parkinson’s disease, ALS amyotrophic lateral sclerosis, LD linkage disequilibrium, SNP single nucleotide polymorphism.

In this study, we adopted the two-sample MR design (Fig. 1), a genetic instrumental variable analysis based on summary-level data with single nucleotide polymorphisms (SNPs) as instruments for the risk factor. This analytical approach minimizes the influence of confounding and reverse causation bias, since SNPs are randomly allocated at conception. To obtain valid instrumental variables, it is essential that the MR assumptions hold. These assumptions include: (1) the SNPs are associated with the exposure; (2) the SNPs are independent of confounders of the risk factor-outcome association; and (3) the SNPs influence the outcome only via the exposure [14].

### Data source and single nucleotide polymorphism selection

We used GWASs conducted primarily among individuals of European ancestry to identify the genetic instruments of the modifiable risk factors. Since GWAS, with a small sample size and limited statistical power, might fail to detect SNP-trait associations [15], we only kept datasets with *N* > 50,000 and both cases and controls are >10,000 for binary phenotypes. An overview of these data sources was presented in Table 1. From all the identified variants in each gene, only SNPs that were significantly associated with exposure factors (*P* < 5 × 10^−8^) and clumped to a linkage disequilibrium (LD) threshold of *r*^2^ < 0.001 were considered candidate proxies. This led to an inclusion of 143/132/142 (AD/PD/ALS) SNPs for hypertension, 400/380/380 SNPs for systolic blood pressure (SBP), 398/375/375 SNPs for diastolic blood pressure (DBP), 343/326/326 SNPs for pulse pressure (PP), 37/32/35 SNPs for type 2 diabetes (T2D), 64/60/61 SNPs for fasting glucose (FG), 35/31/33 SNPs for fasting insulin (FI), 12/10/10 SNPs for 2-hour postprandial blood glucose (2hGlu), 68/62/64 SNPs for glycosylated hemoglobin (HbA1c), 50/44/48 SNPs for dyslipidemia, 85/79/79 SNPs for high-density lipoprotein (HDL), 76/40/40 SNPs for low-density lipoprotein (LDL), 55/50/50 SNPs for triglyceride (TG), 34/33/33 SNPs for insomnia, 60/57/58 SNPs for overall-sleep duration, 7/5/7 SNPs for long-sleep duration (≥9 versus 7–8 h/day), 23/23/23 SNPs for short sleep duration (<7 versus 7–8 h/day), 715/680/680 SNPs for BMI, 23/20/22 SNPs for coffee intake, 16/25/18 SNPs for tea intake, 73/70/70 SNPs for smoking initiation, 20/17/17 SNPs for cigarettes per day, 32/32/32 SNPs for drinking per week, 262/253/253 SNPs for educational level. All data used in this MR study are publicly available and the data sources were shown and described in detail in Additional files 1–3; Table S1.

**Table 1:** Overview of the data sources of the instrumental variables used in the MR study.

Risk factor	Used SNPs†	Sample size	Ancestry	Unit‡
Blood pressure				
Hypertension	143/132/142	390406	European	Odds of hypertension
Systolic blood pressure	400/380/380	Over 1 million	European	1 mmHg increase
Diastolic blood pressure	398/375/375	Over 1 million	European	1 mmHg increase
Pulse pressure	343/326/326	Over 1 million	European	1 mmHg increase
Glucose				
Type 2 diabetes	37/32/35	898130	European	Odds of type 2 diabetes
Fasting glucose	64/60/61	281416	Mostly European	mmol/L
Fasting insulin	35/31/33	281416	Mostly European	mmol/L
2 h glucose	12/10/10	281416	Mostly European	mmol/L
HbA1c	68/62/64	281416	Mostly European	mmol/L
Lipids				
Dyslipidemia	50/44/48	390406	European	Odds of dyslipidemia
LDL-C	76/40/40	188577	Mostly European	1 SD increase
HDL-C	85/79/79	188577	Mostly European	1 SD increase
Triglycerides	55/50/50	188577	Mostly European	1 SD increase
Sleep				
Insomnia	34/33/33	1331010	European	Odds of insomnia
Overall-sleep duration	60/57/58	446118	European	h/d
Short sleep duration	23/23/23	411934	European	<7 h/d vs 7–8 h/d
Long-sleep duration	7/5/7	339926	European	≥9 h/d vs 7–8 h/d
Overweight				
Body mass index	715/680/680	≈700000	European	1 SD increase
Diet				
Coffee intake	23/20/22	501494	European	cup/d
Tea intake	16/25/18	501494	European	cup/d
Smoking				
Smoking initiation	73/70/70	1232091	European	Ever smoked regularly compared with never smoking
Cigarettes per day	20/17/17	337334	European	1 SD increase in the number of cigarettes smoked per day
Drinking				
Alcohol consumption	32/32/32	941280	European	1-SD increase in log-transformed alcoholic drinks/wk
Education				
Educational level	262/253/253	1131881	European	1 SD increase in years of educational attainment

*GWAS* genome-wide association study, *HDL-C* high-density lipoprotein cholesterol, *LDL-C* low-density lipoprotein cholesterol, *MR* Mendelian randomization, *NA* not available, *SNP* single nucleotide polymorphism, *HbAlc* glycosylated hemoglobin, *PD* Parkinson’s disease, *AD* Alzheimer’s disease, *ALS* amyotrophic lateral sclerosis.

†SNPs used in investigating associations of modifiable risk factors and AD/PD/ALS risks.

‡Units used in the present MR analysis.

### Statistical analyses

The inverse-variance weighted (IVW) method with a multiplicative random-effects model was used as the main analysis method. Since this method might be affected by pleiotropy or invalid instrument bias in case not all MR assumptions hold, we tested the validity and robustness of the results by conducting several sensitivity analyses: MR-Egger, weighted median, simple mode, and weighted mode method. The weighted median method, which is used to check invalid instrument bias, provides a consistent estimate even if over 50% of the information comes from invalid or weak instruments [16]. The estimate of the causal effect provided by the MR-Egger method is less susceptible to the presence of pleiotropy; therefore, the MR-Egger method is better in the presence of pleiotropy [10]. We estimated the intercept of MR-Egger regression, which represented the average horizontal pleiotropy. We also conducted a leave-one-SNP-out analysis to assess the influence of potentially pleiotropic SNPs on the causal estimates by systematically removing one SNP at a time. The strength of the genetic instrument was estimated using F-statistics. If F-statistics are greater than 10, the instrument strength is sufficient for MR analysis [17].

The principal statistical analyses were conducted using R (version 4.2.2) and MR analyses were conducted using the “TwoSampleMR” package. We also performed additional MR analyses by the MRlap, an R-package to perform MR analyses permitting overlapping samples (Additional file 7). Results were reported as odds ratios (OR) with corresponding 95% CIs. Statistical significance was determined by a two-tailed *P* value <0.05.

## Results

### Principal analyses

#### Genetically determined exposures and risk of AD

**Fig. 2:**
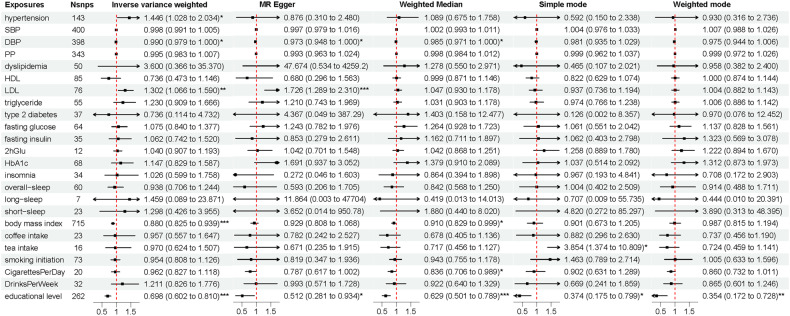
The associations of cardiovascular risk factors and lifestyle behaviors with AD risk. Genome-wide significantly associated (*P* < 5 × 10^−8^) independent (linkage disequilibrium *r*^2^ = 0.001, clumping distance = 10,000 kb) SNPs were used as instruments. AD Alzheimer’s disease, SNP single nucleotide polymorphism, HDL high-density lipoprotein cholesterol, LDL low-density lipoprotein cholesterol, SBP systolic blood pressure, DBP diastolic blood pressure, PP pulse pressure, HbAlc glycosylated hemoglobin, 2 h Glu 2-hour post-meal blood glucose. *, 0.01 < *p* < 0.05; **, 0.001 < *p* < 0.01; ***, *p* < 0.001.

Results using the International Genomics of Alzheimer’s Project (IGAP) dataset showed a statistically significant causal effect of educational level on LOAD (OR_-IVW_ per 1 SD increase = 0.698, 95% CI = 0.602–0.810, *p* = 5.28E-6). The causal effect was confirmed by sensitivity analyses including weighted median (OR_- weighted median_ per 1 SD increase = 0.629, 95% CI = 0.501–0.789, *p* = 6.36E-5) and MR-Egger (OR_-MR-Egger_ per 1 SD increase = 0.512, 95% CI = 0.281–0.934, *p* = 0.029) methods. Sensitivity analyses found no evidence for heterogeneity of effect sizes (leave-one-out; Cochran Q statistic, *p* > 0.05) or for pleiotropy (MR-Egger intercept, *p* > 0.05) (Additional file 5; Table 4). The IVW estimate also showed a protective causal relationship between BMI and LOAD (OR_-IVW_ per 1 SD increase = 0.880, 95% CI = 0.825–0.939, *p* = 1.04E-4). The weighted median method showed similar results (OR_-weighted median_ per 1 SD increase = 0.910, 95% CI = 0.829–0.999, *p* = 0.048). There was evidence of heterogeneity in the causal effect estimates from all of the MR analyses (all *P* values <0.05). Nonetheless, horizontal pleiotropic effects were absent in MR-Egger regression (intercept: −0.001, *P* = 0.387) (Additional file 5; Table 4). Unlike educational level and BMI, LDL might increase LOAD risk (OR_-IVW_ per 1 SD increase = 1.302, 95% CI = 1.066–1.590, *p* = 0.010). The evidence was confirmed by MR-Egger (OR_-MR-Egger_ per 1 SD increase = 1.726, 95% CI = 1.289–2.310, *p* = 4.56E-4) but challenged by the presence of heterogeneity amongst SNPs (*P* values <0.05). Besides, potential evidence of a relationship between hypertension and LOAD (hypertension: OR_-IVW_ per 1 SD increase = 1.446, 95% CI = 1.028–2.034, *p* = 0.034) was obtained; the evidence was challenged by the absence of support from sensitivity analyses. (Fig. 2).

In the UK Biobank (UKB) cohort, results showed suggestive associations between several risk factors and familial AD. BMI was associated with reduced maternal AD risk (OR_-IVW_ per 1 SD increase = 0.922, 95% CI = 0.859–0.990, *p* = 0.025; OR_-MR-Egger_ per 1 SD increase = 0.844, 95% CI = 0.724–0.984, *p* = 0.030), without heterogeneity of effect size (leave-one-out; Cochran Q statistic, *p* > 0.05) and directional pleiotropy (MR-Egger intercept, *p* > 0.05). However, high LDL was found to increase maternal AD risk using IVW and MR-Egger (OR_-IVW_ per 1 SD increase = 2.332, 95% CI = 1.370–3.970, *p* = 0.002; OR_-MR-Egger_ per 1 SD increase = 5.266, 95% CI = 2.259–12.276, *p* = 3.99E-4). Even without support from sensitivity analyses, the IVW estimate revealed a potential casual effect of smoking on maternal AD risk (cigarettes per day: OR_-IVW_ per 1 SD increase = 1.176, 95% CI = 1.010–1.370, *p* = 0.036). A positive association was obtained between LDL level and paternal AD risk (OR_-IVW_ per 1 SD increase = 1.914, 95% CI = 1.170–3.132, *p* = 0.009; OR_-MR-Egger_ per 1 SD increase = 4.127, 95% CI = 1.889-0.013, *p* = 9.48E-4; Cochran Q statistic, *p* < 0.05; intercept: −0.023, *p* = 0.018). The associations of hypertension and BP with paternal AD were not confirmed by sensitivity analyses. (Additional file 4; Tables S2, 3).

#### Genetically determined exposures and risk of PD

**Fig. 3:**
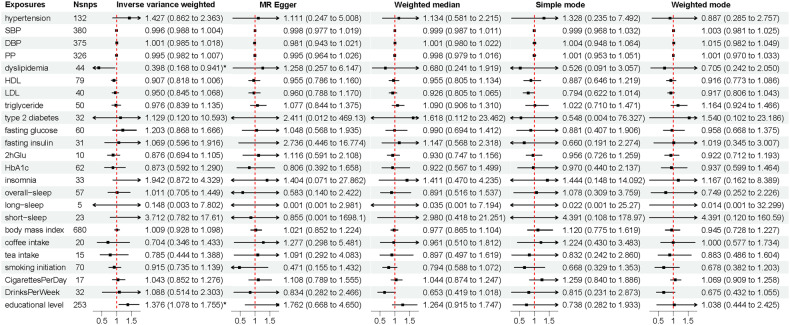
The associations of cardiovascular risk factors and lifestyle behaviors with PD risk. Genome-wide significantly associated (*P* < 5 × 10^−8^) independent (linkage disequilibrium *r*^2^ = 0.001, clumping distance = 10,000 kb) SNPs were used as instruments. PD Parkinson’s disease, SNP single nucleotide polymorphism, HDL high-density lipoprotein cholesterol, LDL low-density lipoprotein cholesterol, SBP systolic blood pressure, DBP diastolic blood pressure, PP pulse pressure, HbAlc glycosylated hemoglobin, 2 h Glu 2-hour post-meal blood glucose. *, 0.01 < *p* < 0.05.

The association between educational level and PD (OR_-IVW_ per 1 SD increase = 1.376, 95% CI = 1.078–1.755, *p* = 0.010) showed suggestive significance but without confirmation by sensitivity analyses (Fig. 3).

#### Genetically determined exposures and risk of ALS

**Fig. 4:**
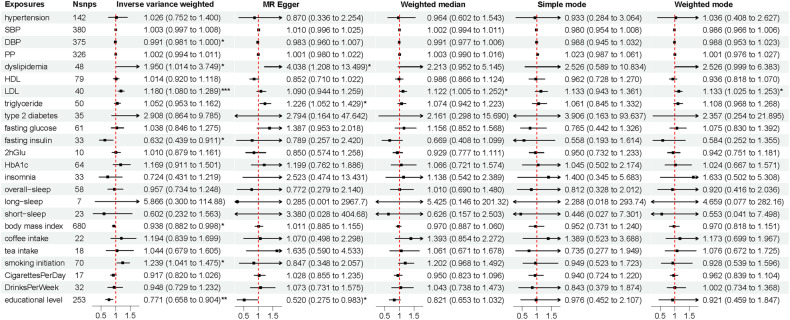
The associations of cardiovascular risk factors and lifestyle behaviors with ALS risk. Genome-wide significantly associated (*P* < 5 × 10^−8^) independent (linkage disequilibrium *r*^2^ = 0.001, clumping distance = 10,000 kb) SNPs were used as instruments. ALS amyotrophic lateral sclerosis, SNP single nucleotide polymorphism, HDL high-density lipoprotein cholesterol, LDL low-density lipoprotein cholesterol, SBP systolic blood pressure, DBP diastolic blood pressure, PP pulse pressure, HbAlc glycosylated hemoglobin, 2 h Glu 2-hour post-meal blood glucose. *, 0.01 < *p* < 0.05; **, 0.001 < *p* < 0.01; ***, *p* < 0.001.

Among the risk factors, LDL was positively associated with ALS (LDL: OR_-IVW_ per 1 SD increase = 1.180, 95% CI = 1.080–1.289, *p* = 2.40E-4; OR_- weighted median_ per 1 SD increase = 1.122, 95% CI = 1.005–1.252, *p* = 0.040; OR_-weighted mode_ per 1 SD increase = 1.133, 95% CI = 1.025–1.253, *p* = 0.020) at a nominal *P* value threshold of 0.05, while the educational level was negatively associated with ALS (OR_-IVW_ per 1 SD increase = 0.771, 95% CI = 0.658–0.904, *p* = 0.001). Without confirmation from sensitivity analyses, the associations of dyslipidemia, FI, BMI, and smoking initiation with ALS were less robust (Fig. 4).

### Additional analyses

The causal evidence by the “TwoSampleMR” package did not change its significance in the additional analyses. We further discovered the causal relationship between SBP and LOAD as well as familial AD. Besides, short sleep duration also suggested significance in correlation with PD. And then, the additional MR analyses by the “MRlap” package drew another four potential risk factors, including hypertension, DBP, diabetes, and 2hGlu. Further details were exhibited in Table 2.

**Table 2:** Associations of cardiovascular risk factors and lifestyle behaviors with AD, PD, and ALS (by “MRlap” package).

Exposures	AD_IVW beta (se)	Paternal AD_IVW beta (se)	Maternal AD_IVW beta (se)	PD_IVW beta (se)	ALS_IVW beta (se)
Hypertension	0.095 (0.026) ^***^	0.040 (0.012) ^***^	0.031 (0.011) ^**^	0.011 (0.010)	0.049 (0.024) ^*^
SBP	−0.062 (0.02) ^**^	−0.019 (0.009) ^*^	−0.023 (0.008) ^**^	−0.0001 (0.007)	−0.032 (0.017)
DBP	−0.052 (0.019) ^**^	−0.030 (0.008) ^***^	−0.019 (0.008) ^*^	−0.005 (0.007)	−0.051 (0.017) ^**^
PP	−0.058 (0.023) ^*^	−0.014 (0.010)	−0.017 (0.009)	0.003 (0.008)	0.002 (0.017)
Dyslipidemia	0.028 (0.057)	0.074 (0.033) ^*^	0.070 (0.035) ^*^	−0.025 (0.014)	0.080 (0.032) ^*^
HDL	0.013 (0.023)	0.0001 (0.009)	0.001 (0.011)	−0.013 (0.006) ^*^	0.026 (0.019)
LDL	0.067 (0.021) ^**^	0.029 (0.011) ^**^	0.047 (0.015) ^**^	−0.004 (0.006)	0.041 (0.014) ^**^
Triglyceride	0.044 (0.026)	0.010 (0.014)	0.017 (0.017)	−0.011 (0.009)	0.021 (0.020)
Type 2 diabetes	−0.038 (0.075)	−0.035 (0.031)	−0.006 (0.025)	−0.003 (0.029)	0.104 (0.051) ^*^
Fasting glucose	0.006 (0.03)	0.0001 (0.010)	−0.002 (0.008)	0.010 (0.008)	0.005 (0.009)
Fasting insulin	0.004 (0.038)	0.001 (0.002)	−0.001 (0.002)	−0.002 (0.011)	−0.011 (0.010)
2 h Glu	0.043 (0.022)	−0.004 (0.004)	−0.001 (0.004)	−0.027 (0.015)	0.023 (0.010) ^*^
HbA1c	0.021 (0.022)	−0.0001 (0.008)	−0.005 (0.008)	−0.009 (0.007)	0.017 (0.017)
Insomnia	−0.083 (0.142)	−0.002 (0.064)	−0.047 (0.064)	0.038 (0.068)	−0.151 (0.143)
Overall-sleep	0.031 (0.066)	0.012 (0.025)	−0.006 (0.026)	0.067 (0.042)	−0.022 (0.060)
Long-sleep	0.192 (0.321)	0.051 (0.093)	−0.061 (0.093)	0.643 (0.332) ^*^	0.214 (0.284)
Short sleep	0.166 (0.135)	0.010 (0.055)	0.069 (0.054)	0.099 (0.048) ^*^	−0.014 (0.122)
Body mass index	−0.03 (0.008) ^***^	−0.006 (0.003)	−0.008 (0.003) ^*^	0.003 (0.003)	−0.002 (0.007)
Coffee intake	−0.015 (0.085)	−0.012 (0.033)	0.010 (0.032)	−0.028 (0.033)	0.066 (0.055)
Tea intake	−0.021 (0.076)	0.018 (0.038)	0.006 (0.034)	−0.036 (0.033)	0.082 (0.078)
Smoking initiation	−0.112 (0.07)	−0.032 (0.030)	0.002 (0.026)	−0.024 (0.025)	0.179 (0.066) ^**^
CigarettesPerDay	0.033 (0.064)	0.018 (0.026)	0.058 (0.027) ^*^	0.007 (0.029)	−0.066 (0.050)
DrinksPerWeek	0.106 (0.088)	0.008 (0.033)	0.005 (0.035)	0.075 (0.064)	0.031 (0.059)
Educational level	−0.205 (0.036) ^***^	0.015 (0.015)	0.011 (0.005) ^*^	0.055 (0.015) ^***^	−0.130 (0.035) ^***^

*HDL* high-density lipoprotein cholesterol, *LDL* low-density lipoprotein cholesterol, *SBP* systolic blood pressure, *DBP* diastolic blood pressure, *PP* pulse pressure, *HbAlc* glycosylated hemoglobin, *2* *h Glu* 2-hour post-meal blood glucose, *PD* Parkinson’s disease, *AD* Alzheimer’s disease, *ALS* amyotrophic lateral sclerosis, *IVW* Inverse-variance weighted.

*, 0.01 < *p* < 0.05; **, 0.001 < *p* < 0.01; ***, *p* < 0.001.

## Discussion

In this two-sample MR study, we found that both LOAD and familial AD had significant associations with genetically determined cardiovascular risk factors and lifestyle behaviors, including educational level, BMI, plasma lipids, and BP categories. Furthermore, we found the causal associations of dyslipidemia and LDL with ALS, as well as the potential associations of sleep duration and smoking initiation with PD and ALS, respectively. However, all these results should be interpreted with great caution.

There was accumulating observational evidence that a high educational level may protect against AD [18]. Besides, educational level was identified as a protective factor for cognitive deterioration related to other risk factors [19]. For one thing, the current study also showed a protective effect of high educational levels on AD risk. The polymorphism rs9320913 (closest gene = MMS22L), which was genome-wide and significantly associated with educational level, might indirectly involve in processes contributing to cognitive reserve for its function in neuron apoptosis and neuroinflammation [20]. Therefore, as a proxy of cognitive reserve, a high educational level might show resilience against cognitive decline even in the presence of neuropathology [21]. For another, one MR study reported a bidirectional association between intelligence and educational level, so prior intelligence might also mediate the association between educational level and AD risk [22]. Together, these findings suggested the potential of reducing AD risk by improving various aspects of cognitive reserve (e.g., with cognitive training), which might compensate for lower educational attainment.

As a genetic instrument of BMI, rs17125944 (closest gene = FERMT2) was also associated with AD risk. Therefore, BMI and AD might be linked by the dysfunction of scaffolding protein. Using the MR approach, the current study proved the protective effect of BMI on AD dementia, which were inconsistent with that of another MR study [23]. Mukherjee and colleagues used the genome-wide significant SNPs and the associated β weights from the published meta-analysis (249,796 individuals included) to construct polygenic scores. Though no evidence from individual SNPs or polygenic scores indicated BMI increased AD risk, the effect estimates suggested a potential protective role of BMI in AD risk. Based on Mukherjee’s work, we updated the MR analyses using the summary data from a recent meta-analysis with a bigger sample (over 1 million participants) and further confirmed the protective role of BMI in AD risk. The hormone leptin, mainly secreted by the adipose tissue, might work as a cognitive enhancer. In animal models, leptin was found to enhance adult neurogenesis and reduce pathological features by modulating the formation of senile plaque, as well as attenuating Aβ-induced neurodegeneration and superoxide anion production [24]. Besides, in clinical practice, lower levels of leptin were reported as a risk factor for developing AD after a 12-year follow-up by the Framingham study [25]. However, studies showed higher BMI in midlife increased dementia risk [26], while late-life BMI might exert the opposite effect [27]. Hence, BMI might have age-depended effects on AD risk. In addition, the true association between BMI and AD risk might also be biased by selection bias and epigenetics. As a result, the spurious association between BMI and AD could occur when competing risks and epigenetics of the outcome existed.

Dyslipidemia was implicated as a risk factor for AD [28]. Genetic enrichment in AD was predominantly related to plasma lipids, such as rs3844143 (closest gene = PICALM) [29], but the mechanism by which LDL modulates the risk for AD remained elusive. A high level of LDL in the plasma affected the flux of oxidized metabolite 27-hydroxycholesterol (27OH) from circulation into the brain. And then, the excessive accumulation of 27OH in the brain leads to the elevated deposition of Aβ, a key initiating event in AD [30]. In addition, a high plasma level of LDL was implicated in the impairment of the blood–brain barrier (BBB) [31], which enabled circulating LDL to enter the brain and executed a direct effect on the pathogenesis of AD, further promoting Aβ deposition [32]. However, this MR analysis showed evidence of horizontal pleiotropy in the relationship between LDL and AD, and therefore we could not ignore the effects of confounders. Many studies have demonstrated that high concentrations of LDL are associated with coronary heart disease and carotid artery atherosclerosis, which, in turn, may lead to cognitive decline through cerebral embolism or hypoperfusion [33].

Using neuroimaging, previous prospective studies showed significant associations between increased BP with smaller brain volumes [34] as well as increased Aβ brain burden[35]. All the above evidence indicated that BP might affect cognitive function by regulating brain volume and Aβ deposition. However, several previous studies indicated that higher AD polygenic risk scores (PRS) were associated with lower BP [36], which was confirmed by the current study demonstrating an inverse association between BP and the risk of AD using MR. The inconsistency might be caused by the following reasons: antihypertensive medications might cover up the real association between BP and the risk of AD since calcium channel blocker (CCB) was identified as a promising strategy for AD prevention [37]; BP might be positively relevant for AD risk only above a certain threshold, that is, BP served as a double-edged sword; like BMI, BP might also have age-depended effects on AD risk; the true correlation between BP and AD risk could be confused by BP-related cardiovascular diseases, which served as competing risks.

Both dyslipidemia and LDL accounted for the ALS risk in this MR analysis; the results were in line with a more than 20-year follow-up study [38]. LDL was the major carrier of cholesterol in the peripheral circulation. Excess cholesterol was metabolized to a more soluble form, oxysterols. Oxysterols could readily cross the barrier and the increased intracellular oxysterol levels eroded cell viability, especially neuronal cells [39]. BBB and blood–spinal cord barrier (BSCB) impairments were reported in ALS patients and SOD-1 mouse models [40, 41]. Thereby, we hypothesized that oxysterols might involve in oxidative stress, resulting in the dysfunction of neurons and the destruction of BBB and BSCB.

We also found a positive association between educational level and PD risk; the finding contradicts the motor reserve theory that high educational levels protect against PD by exerting a protective effect on white matter integrity [42]. Since the educational level is influenced by both genetic and familial environmental factors, future longitudinal co-twin control studies may explain the conflicting results. Using the “MRlap” package, a short sleep duration and long-sleep duration were identified as the potential risk factors for PD, which revealed a non-linear association of sleep duration with the risk of PD. The sleep-wake cycle was reported to increase extracellular levels of tau and alpha-synuclein [43]. Besides, brain autopsy revealed that increased actigraphy-derived sleep fragmentation in old subjects without PD was associated with an increased burden of PD pathology [44]. All these indicated that potential pathways between sleep duration and PD might include increased oxidative stress, or reduced clearance of extracellular alpha-synuclein. Interestingly, another study demonstrated that the rs2028122 genotype partially mediated the causal pathway of sleep duration, leading to the development of PD on a positive effect [45]; therefore, the association between sleep duration and PD risk might vary across different rs2028122 genotypes.

To sum up, we confirmed a set of cardiovascular risk factors and lifestyle behaviors leading to neurodegenerative diseases in the current MR study. All the identified risk factors indicated common underlying mechanisms among neurodegenerative diseases: neuroinflammation and excessive oxidative stress. Since BBB was the main protective barrier of the central nervous system (CNS), an increased BBB permeability could disbalance the homeostasis and affect innate and adaptative immune responses. When optimal communication between the brain and systemic immune system fails to occur appropriately, inflammatory mediators accumulate in the CNS in a process known as neuroinflammation and trigger brain damage [46]. Besides, the CNS had a high metabolic rate with favoring free radical formation. Under oxidative stress conditions, dysfunctional mitochondria fail to produce the high energy levels required by neuronal cells to perform their normal biochemical and physiological functions, leading to rapid cell death [47]. Neuroinflammation, oxidative stress, and mitochondrial dysfunction lead to aggregation of misfolded protein (Aβ and tau, α-Syn, and TDP-43 [TAR DNA-binding protein] and SOD-1[superoxide dismutase] are the proteins involved in AD, PD, and ALS, respectively) which could trigger each disease [48]. Therefore, preventing or minimizing neuroinflammation throughout all stages of life might proactively and cumulatively reduce the risk of developing neurodegenerative diseases.

Several limitations should be considered. First, although genetic variants were derived from studies with relatively large sample sizes, our finding might still be affected by weak instrument bias. Subsequent studies need to struck a balance between including fewer variants (potentially having insufficient power) and including more variants (potentially including pleiotropic variants). Second, we couldn’t exclude the possibility of inflating the type 1 error rate since there were overlaps between exposure GWASs and outcome GWASs. In addition, most GWAS studies recruited in middle-to-old age, in which participants were inevitably survivors of the genetic instruments. As a result, the effect estimates might be distorted in survivor bias and selection bias. Third, A growing body of evidence suggested that the impact of environmental influences might extend beyond the DNA sequence, so epigenetic bias could distort the effects detected in our MR study. Future two-step epigenetic MR study, with a further understanding of the causal role of epigenetics (such as DNA methylation) in mediating environmental influences on common complex disease, would overcome the potential for confounding and reverse causation. Fourth, the explanation concerning the current MR-based results by amyloid hypothesis required further investigation, since still other studies yielded null findings regarding amyloid and AD [49]. Future progress in progress in neuroimaging might help to figure out the uncertain mechanisms. Last, our population was limited to individuals of European ancestry, which might limit the generalizability of our findings to other ethnic groups.

In this two-sample MR study, we found that LDL, BMI, BP, and educational level were causally related to AD. Besides, we also observed a significant association between LDL and ALS risk as well as the potential effect of sleep duration on PD risk. All these results implied that targeting these modifiable factors could facilitate the prevention of neurodegenerative disease.

## Supplementary information

Supplemental material

## Data Availability

All the data used in this study can be acquired from the original genome-wide association studies that are mentioned in the text or in its additional files. Any other data generated in the analysis process can be requested from the corresponding author.
