# Supplementary material for: Associations of cardiovascular risk factors and lifestyle behaviors with neurodegenerative disease: a Mendelian randomization study
Source: Transl Psychiatry. 2023 Jul 24;13:267. doi: 10.1038/s41398-023-02553-9 (PMC10366095; doi:10.1038/s41398-023-02553-9)
Supplement: Supplementary file 1 — Supplemental material [file 41398_2023_2553_MOESM1_ESM.docx]

**Associations of Cardiovascular Risk Factors and Lifestyle Behaviors with Neurodegenerative Diseases: A** **Mendelian Randomization Study**

**Content:**

| **TITLE** | **PAGE** |
| --- | --- |
| Additional file 1. Detailed description of data sources for exposures and neurodegenerative diseases | 2 |
| Additional file 2. Data resources for exposures and neurodegenerative diseases | 3 |
| Additional file 3. Demographic characteristics of included GWASs used in the present MR. (Table S1) | 4 |
| Additional file 4. Results of associations between modifiable risk factors and familial AD. (Table S2-3) | 9 to 10 |
| Additional file 5. Sensitivity analysis of instrument effects. (Table S4) | 11 to 12 |
| Additional file 6. Genome-wide significant and independent SNPs that were used as instruments. (Table. 1-20) | 13 to 99 |
| Additional file 7. MRlap package | 100 |

**Additional file 1. Detailed description of data sources for exposures and neurodegenerative diseases**

**Supplementary file 1a. Data source and single‑nucleotide polymorphism selection for exposures.**

We used GWASs conducted primarily among individuals of European ancestry to identify the genetic instruments of the modifiable risk factors. An overview of these data sources was presented in the **Table 1**. From all the identified variants in each gene, only SNPs that were significantly associated with exposure factors (P < 5×10^-8^) and clumped to a linkage disequilibrium (LD) threshold of r^2^ < 0.001 were considered as candidate proxies. This led to an inclusion of 143/132/142 (AD/PD/ALS) SNPs for hypertension **[1]**, 400/380/380 SNPs for systolic blood pressure (SBP) **[2]**, 398/375/375 SNPs for diastolic blood pressure (DBP) **[2]**, 343/326/326 SNPs for pulse pressure (PP) **[2]**, 37/32/35 SNPs for type 2 diabetes (T2D) **[1]**, 64/60/61 SNPs for fasting glucose (FG) **[3]**, 35/31/33 SNPs for fasting insulin (FI) **[3]**, 12/10/10 SNPs for 2-hour postprandial blood glucose (2hGlu) **[3]**, 68/62/64 SNPs for glycosylated hemoglobin (HbA1c) **[3]**, 50/44/48 SNPs for dyslipidemia **[1]**, 85/79/79 SNPs for high-density lipoprotein (HDL) **[4]**, 76/40/40 SNPs for low-density lipoprotein (LDL) **[4]**, 55/50/50 SNPs for triglyceride (TG) **[4]**, 34/33/33 SNPs for insomnia **[5]**, 60/57/58 SNPs for overall sleep-duration **[6]**, 7/5/7 SNPs for long sleep-duration (≥ 9 versus 7–8 hours/day) **[6]**, 23/23/23 SNPs for short sleep-duration ((< 7 versus 7-8 hour/day)) **[6]**, 715/680/680 SNPs for BMI **[7]**, 23/20/22 SNPs for coffee intake **[1]**, 16/25/18 SNPs for tea intake **[1]**, 73/70/70 SNPs for smoking initiation **[8]**, 20/17/17 SNPs for cigarettes per day **[8]**, 32/32/32 SNPs for drinking per week **[8]**, 262/253/253 SNPs for educational level **[9]**.

**Supplementary file 1b. Data Sources for Alzheimer’s disease**

We extracted the genetic associations of the instrumental variables with Alzheimer’s disease from the GWAS summary statistics: 1) the International Genomics of Alzheimer’s Project (IGAP) GWAS Stage 1 result (N = 21,982 cases, 41,944 controls) **[10]**; 2) maternal UK Biobank family history of Alzheimer’s Disease (N = 27,696 cases, 260,980 controls); 3) paternal UK Biobank family history of Alzheimer’s Disease (N = 14,338 cases, 245,941 controls) **[11]**. The IGAP consists of the Alzheimer Disease Genetics Consortium (ADGC), the Cohorts for Heart and Aging Research in Genomic Epidemiology Consortium (CHARGE), the European Alzheimer’s Disease Initiative (EADI), as well as Genetic and Environmental Risk in AD/Defining Genetic, Polygenic and Environmental Risk for Alzheimer's Disease Consortium (GERAD/PERADES). The published criteria were used to confirm Alzheimer’s disease by autopsy or clinical methods. The mean age of AD cases ranged from 71.1 to 82.6 years, and the mean age of healthy controls ranged from 51.0 to 78.9 years. UK Biobank is a population-based prospective cohort collecting data from individuals aged 37–73 years. Self-reported family history of AD/dementia was used as a proxy for AD diagnosis in the UKBB cohort.

**Supplementary file 1c. Data Sources for PD and ALS**

The PD GWAS dataset was collected from the recently published GWAS meta-analysis **[12]** (data resources were provided in **supplementary file 2**). This meta-analysis included three sources of data (three previously published GWAS studies, 13 new datasets, and proxy-case data from the UK Biobank), resulting in 37,688 cases, 18,618 UK Biobank proxy-cases (i.e, individuals who do not have Parkinson’s disease but have a first degree relative that does), and 1.4 million controls. The summary statistics for ALS were obtained from a largest GWAS **[13]** involving 80,610 participants of European descent (20,806 ALS cases and 59,804 control samples). All cases included in the large-scale GWAS study were diagnosed with probable or definite ALS according to EI Escorial criteria ^16^ (**see Supplementary file 3**).

**Additional 2. Data resources for exposures and neurodegenerative diseases**

**References**

1. <http://geneatlas.roslin.ed.ac.uk/>.
2. Evangelou E, Warren HR, Mosen-Ansorena D, et al. Genetic analysis of over 1 million people identifies 535 new loci associated with blood pressure traits. Nat Genet 2018;50:1412-1425.

3. Ji C, Cassandra NS, Gaelle M, et al. The trans-ancestral genomic architecture of glycemic traits. Nat Genet 2021 Jun;53(6):840-860.

4. Willer CJ, Schmidt EM, Sengupta S, et al. Discovery and refinement of loci associated with lipid levels. Nat Genet 2013;45:1274-1283.

5. Jansen PR, Watanabe K, Stringer S, et al. Genome-wide analysis of insomnia in 1,331,010 individuals identifies new risk loci and functional pathways. Nat Genet 2019;51:394-403.

6. Dashti HS, Jones SE, Wood AR, et al. Genome-wide association study identifies genetic loci for self-reported habitual sleep duration supported by accelerometer-derived estimates. Nat Commun 2019;10:1100.

7. Yengo L, Sidorenko J, Kemper KE, et al. Meta-analysis of genome-wide association studies for height and body mass index in approximately 700000 individuals of European ancestry. Hum Mol Genet 2018;27:3641-3649.

8. Liu M, Jiang Y, Wedow R, et al. Association studies of up to 1.2 million individuals yield new insights into the genetic etiology of tobacco and alcohol use. Nat Genet 2019;51:237-244.

9. Lee JJ, Wedow R, Okbay A, et al. Gene discovery and polygenic prediction from a genome-wide association study of educational attainment in 1.1 million individuals. Nat Genet 2018;50:1112-1121.

10. Kunkle BW, Grenier-Boley B, Sims R, et al. Genetic meta-analysis of diagnosed Alzheimer's disease identifies new risk loci and implicates Abeta, tau, immunity and lipid processing. Nat Genet 2019;51:414-430.

11. Marioni RE, Harris SE, Zhang Q, et al. GWAS on family history of Alzheimer's disease. Transl Psychiatry 2018;8:99.

12. Nalls MA, Blauwendraat C, Vallerga CL, et al. Identification of novel risk loci, causal insights, and heritable risk for Parkinson's disease: a meta-analysis of genome-wide association studies. Lancet Neurol 2019;18:1091-1102.

13. Nicolas A, Kenna KP, Renton AE, et al. Genome-wide Analyses Identify KIF5A as a Novel ALS Gene. Neuron 2018;97:1268-1283 e1266.

**Additional file 3. Table S1. Demographic characteristics of included GWASs used in the present MR**

| **GWAS type** | **Sample size (case/control)** | **Phenotype ascertainment** |
| --- | --- | --- |
| **Late-on-set AD GWAS** | 21982/41994 |  |
| ADGC | 14428/14562 | Autopsy-confirmed or clinically-confirmed |
| CHARGE | 2137/13474 | Autopsy-confirmed or clinically-confirmed |
| EADI | 2240/6631 | Autopsy-confirmed or clinically-confirmed |
| GERAD/PERADES | 3177/7277 | Autopsy-confirmed or clinically-confirmed |
| **Paternal AD GWAS** | 14338/245941 | Self-reported |
| **Maternal AD GWAS** | 27696/260980 | Self-reported |
| **PD GWAS** |  |  |
| Nalls and colleagues | 13708/95282 | Clinically-confirmed |
| IPDGC-NeuroX | 5851/5866 | Clinically-confirmed |
| PDWBS | 6476/302042 | Clinically-confirmed |
| UK Biobank | 18618/436419 | Self-reported |
| SGPD | 1169/968 | Clinically-confirmed |
| IPDGC | 8036/5803 | Clinically-confirmed |
| Post-Chang, 23andMe | 2448/571411 | Clinically-confirmed |
| **ALS GWAS** | 20806/59804 |  |
| Italy | 2853/2143 | Clinically-confirmed |
| United Kingdom | 449/226 | Clinically-confirmed |
| Belgium/France | 1150/595 | Clinically-confirmed |
| USA | 3777/33365 | Clinically-confirmed |
| Van Rheenen Study | 12577/23475 | Clinically-confirmed |

Abbreviations: GWAS, Genome wide association study; MR, Mendelian randomization; ADGC, Alzheimer Disease Genetics Consortium; CHARGE, Cohorts for Heart and Aging Research in Genomic Epidemiology Consortium; EADI, Genetic and Environmental Risk in AD/Defining Genetic; GERAD/PERADES, Polygenic and Environmental Risk for Alzheimer's Disease Consortium; UKB, UK Biobank; ICBP, International Consortium of Blood Pressure Genome Wide Association Studies; IPDGC=International Parkinson’s Disease Genomics Consortium. PDWBS=Parkinson’s disease web-based study. SGPD= Systems genomics of Parkinson’s disease consortium.

**Additional file 4. Table S2. Results of associations between modifiable risk factors and maternal AD.**

| Exposures | Methods | Nsnps | OR | OR (95% CI) | pval |
| --- | --- | --- | --- | --- | --- |
| hypertension | MR Egger | 138 | 0.357 | 0.357 (0.102 to 1.251) | 0.11 |
| hypertension | Weighted median | 138 | 1.141 | 1.141 (0.63 to 2.066) | 0.664 |
| hypertension | IVW | 138 | 1.503 | 1.503 (0.983 to 2.298) | 0.06 |
| hypertension | Simple mode | 138 | 1.958 | 1.958 (0.471 to 8.141) | 0.357 |
| hypertension | Weighted mode | 138 | 0.779 | 0.779 (0.252 to 2.405) | 0.664 |
| SBP | MR Egger | 380 | 1.005 | 1.005 (0.986 to 1.024) | 0.601 |
| SBP | Weighted median | 380 | 0.994 | 0.994 (0.983 to 1.005) | 0.285 |
| SBP | IVW | 380 | 0.994 | 0.994 (0.987 to 1.002) | 0.132 |
| SBP | Simple mode | 380 | 0.992 | 0.992 (0.963 to 1.023) | 0.611 |
| SBP | Weighted mode | 380 | 1.002 | 1.002 (0.981 to 1.024) | 0.858 |
| DBP | MR Egger | 374 | 1.029 | 1.029 (0.999 to 1.06) | 0.061 |
| DBP | Weighted median | 374 | 0.995 | 0.995 (0.977 to 1.013) | 0.567 |
| DBP | IVW | 374 | 0.995 | 0.995 (0.983 to 1.007) | 0.433 |
| DBP | Simple mode | 374 | 1 | 1 (0.952 to 1.051) | 0.991 |
| DBP | Weighted mode | 374 | 0.996 | 0.996 (0.96 to 1.033) | 0.818 |
| PP | MR Egger | 326 | 1.013 | 1.013 (0.978 to 1.05) | 0.464 |
| PP | Weighted median | 326 | 0.991 | 0.991 (0.975 to 1.008) | 0.292 |
| PP | IVW | 326 | 0.993 | 0.993 (0.979 to 1.008) | 0.348 |
| PP | Simple mode | 326 | 1.006 | 1.006 (0.958 to 1.057) | 0.81 |
| PP | Weighted mode | 326 | 0.995 | 0.995 (0.961 to 1.031) | 0.786 |
| dyslipidemia | MR Egger | 46 | 178.272 | 178.272 (1.149 to 27670.713) | 0.05 |
| dyslipidemia | Weighted median | 46 | 1.337 | 1.337 (0.457 to 3.918) | 0.596 |
| dyslipidemia | IVW | 46 | 7.332 | 7.332 (0.509 to 105.716) | 0.143 |
| dyslipidemia | Simple mode | 46 | 0.968 | 0.968 (0.131 to 7.126) | 0.974 |
| dyslipidemia | Weighted mode | 46 | 1.677 | 1.677 (0.519 to 5.425) | 0.392 |
| HDL | MR Egger | 85 | 0.78 | 0.78 (0.349 to 1.745) | 0.547 |
| HDL | Weighted median | 85 | 1.138 | 1.138 (0.968 to 1.339) | 0.118 |
| HDL | IVW | 85 | 0.856 | 0.856 (0.558 to 1.312) | 0.476 |
| HDL | Simple mode | 85 | 1.193 | 1.193 (0.852 to 1.668) | 0.307 |
| HDL | Weighted mode | 85 | 1.15 | 1.15 (0.937 to 1.411) | 0.185 |
| LDL | MR Egger | 44 | 5.266 | 5.266 (2.259 to 12.276) | 0 |
| LDL | Weighted median | 44 | 1.129 | 1.129 (0.958 to 1.33) | 0.147 |
| LDL | IVW | 44 | 2.332 | 2.332 (1.37 to 3.97) | 0.002 |
| LDL | Simple mode | 44 | 1.211 | 1.211 (0.894 to 1.641) | 0.223 |
| LDL | Weighted mode | 44 | 1.096 | 1.096 (0.921 to 1.304) | 0.308 |
| triglyceride | MR Egger | 54 | 1.292 | 1.292 (0.846 to 1.972) | 0.241 |
| triglyceride | Weighted median | 54 | 1.141 | 1.141 (0.962 to 1.352) | 0.13 |
| triglyceride | IVW | 54 | 1.181 | 1.181 (0.907 to 1.537) | 0.216 |
| triglyceride | Simple mode | 54 | 1.139 | 1.139 (0.81 to 1.603) | 0.457 |
| triglyceride | Weighted mode | 54 | 1.096 | 1.096 (0.934 to 1.287) | 0.265 |
| diabetes | MR Egger | 34 | 5.086 | 5.086 (0.133 to 194.504) | 0.388 |
| diabetes | Weighted median | 34 | 2.21 | 2.21 (0.17 to 28.735) | 0.545 |
| diabetes | IVW | 34 | 2.727 | 2.727 (0.589 to 12.621) | 0.199 |
| diabetes | Simple mode | 34 | 3.572 | 3.572 (0.039 to 325.393) | 0.584 |
| diabetes | Weighted mode | 34 | 2.167 | 2.167 (0.114 to 41.222) | 0.61 |
| fasting glucose | MR Egger | 64 | 1.447 | 1.447 (0.376 to 5.572) | 0.595 |
| fasting glucose | Weighted median | 64 | 1.145 | 1.145 (0.605 to 2.168) | 0.678 |
| fasting glucose | IVW | 64 | 0.885 | 0.885 (0.571 to 1.369) | 0.582 |
| fasting glucose | Simple mode | 64 | 1.159 | 1.159 (0.3 to 4.481) | 0.832 |
| fasting glucose | Weighted mode | 64 | 1.432 | 1.432 (0.359 to 5.723) | 0.615 |
| fasting insulin | MR Egger | 33 | 1.004 | 1.004 (0.653 to 1.542) | 0.987 |
| fasting insulin | Weighted median | 33 | 0.895 | 0.895 (0.619 to 1.292) | 0.553 |
| fasting insulin | IVW | 33 | 0.982 | 0.982 (0.782 to 1.233) | 0.873 |
| fasting insulin | Simple mode | 33 | 1.046 | 1.046 (0.512 to 2.135) | 0.902 |
| fasting insulin | Weighted mode | 33 | 0.826 | 0.826 (0.593 to 1.15) | 0.261 |
| 2hGlu | MR Egger | 10 | 1.12 | 1.12 (0.698 to 1.798) | 0.651 |
| 2hGlu | Weighted median | 10 | 1.023 | 1.023 (0.812 to 1.288) | 0.847 |
| 2hGlu | IVW | 10 | 1.039 | 1.039 (0.871 to 1.239) | 0.669 |
| 2hGlu | Simple mode | 10 | 0.982 | 0.982 (0.699 to 1.38) | 0.919 |
| 2hGlu | Weighted mode | 10 | 0.999 | 0.999 (0.732 to 1.363) | 0.994 |
| HbA1c | MR Egger | 66 | 0.759 | 0.759 (0.438 to 1.314) | 0.328 |
| HbA1c | Weighted median | 66 | 0.79 | 0.79 (0.489 to 1.275) | 0.334 |
| HbA1c | IVW | 66 | 0.925 | 0.925 (0.683 to 1.251) | 0.611 |
| HbA1c | Simple mode | 66 | 0.709 | 0.709 (0.3 to 1.672) | 0.434 |
| HbA1c | Weighted mode | 66 | 0.805 | 0.805 (0.493 to 1.313) | 0.388 |
| insomnia | MR Egger | 34 | 1.09 | 1.09 (0.125 to 9.521) | 0.938 |
| insomnia | Weighted median | 34 | 0.727 | 0.727 (0.266 to 1.985) | 0.533 |
| insomnia | IVW | 34 | 0.633 | 0.633 (0.328 to 1.224) | 0.174 |
| insomnia | Simple mode | 34 | 1.166 | 1.166 (0.14 to 9.681) | 0.888 |
| insomnia | Weighted mode | 34 | 0.846 | 0.846 (0.165 to 4.336) | 0.843 |
| overall-sleep | MR Egger | 59 | 0.616 | 0.616 (0.197 to 1.926) | 0.408 |
| overall-sleep | Weighted median | 59 | 0.97 | 0.97 (0.616 to 1.527) | 0.895 |
| overall-sleep | IVW | 59 | 0.934 | 0.934 (0.687 to 1.271) | 0.665 |
| overall-sleep | Simple mode | 59 | 0.753 | 0.753 (0.283 to 2) | 0.571 |
| overall-sleep | Weighted mode | 59 | 1.221 | 1.221 (0.548 to 2.721) | 0.628 |
| long-sleep | MR Egger | 5 | 2.253 | 2.253 (0 to 105887.758) | 0.892 |
| long-sleep | Weighted median | 5 | 0.902 | 0.902 (0.013 to 62.828) | 0.962 |
| long-sleep | IVW | 5 | 0.36 | 0.36 (0.012 to 11.23) | 0.561 |
| long-sleep | Simple mode | 5 | 0.777 | 0.777 (0.004 to 134.904) | 0.928 |
| long-sleep | Weighted mode | 5 | 0.837 | 0.837 (0.006 to 119.259) | 0.947 |
| short-sleep | MR Egger | 23 | 1.082 | 1.082 (0.003 to 425.497) | 0.98 |
| short-sleep | Weighted median | 23 | 1.078 | 1.078 (0.202 to 5.755) | 0.93 |
| short-sleep | IVW | 23 | 1.372 | 1.372 (0.409 to 4.607) | 0.609 |
| short-sleep | Simple mode | 23 | 1.274 | 1.274 (0.07 to 23.129) | 0.871 |
| short-sleep | Weighted mode | 23 | 1.146 | 1.146 (0.076 to 17.175) | 0.922 |
| body mass index | MR Egger | 715 | 0.844 | 0.844 (0.724 to 0.984) | 0.03 |
| body mass index | Weighted median | 715 | 0.907 | 0.907 (0.812 to 1.014) | 0.086 |
| body mass index | IVW | 715 | 0.922 | 0.922 (0.859 to 0.99) | 0.025 |
| body mass index | Simple mode | 715 | 0.829 | 0.829 (0.599 to 1.147) | 0.258 |
| body mass index | Weighted mode | 715 | 0.904 | 0.904 (0.732 to 1.116) | 0.349 |
| coffee intake | MR Egger | 20 | 1.546 | 1.546 (0.482 to 4.961) | 0.473 |
| coffee intake | Weighted median | 20 | 0.901 | 0.901 (0.498 to 1.632) | 0.731 |
| coffee intake | IVW | 20 | 0.997 | 0.997 (0.566 to 1.755) | 0.991 |
| coffee intake | Simple mode | 20 | 1.037 | 1.037 (0.39 to 2.761) | 0.942 |
| coffee intake | Weighted mode | 20 | 1.113 | 1.113 (0.606 to 2.042) | 0.734 |
| tea intake | MR Egger | 14 | 0.965 | 0.965 (0.381 to 2.443) | 0.941 |
| tea intake | Weighted median | 14 | 1.04 | 1.04 (0.59 to 1.832) | 0.893 |
| tea intake | IVW | 14 | 1.091 | 1.091 (0.72 to 1.654) | 0.682 |
| tea intake | Simple mode | 14 | 0.589 | 0.589 (0.204 to 1.697) | 0.345 |
| tea intake | Weighted mode | 14 | 1.001 | 1.001 (0.526 to 1.906) | 0.998 |
| SmokingInitiation | MR Egger | 73 | 0.773 | 0.773 (0.318 to 1.876) | 0.571 |
| SmokingInitiation | Weighted median | 73 | 0.89 | 0.89 (0.681 to 1.162) | 0.391 |
| SmokingInitiation | IVW | 73 | 0.962 | 0.962 (0.809 to 1.144) | 0.664 |
| SmokingInitiation | Simple mode | 73 | 0.824 | 0.824 (0.433 to 1.569) | 0.558 |
| SmokingInitiation | Weighted mode | 73 | 0.773 | 0.773 (0.431 to 1.384) | 0.389 |
| CigarettesPerDay | MR Egger | 20 | 1.128 | 1.128 (0.862 to 1.477) | 0.392 |
| CigarettesPerDay | Weighted median | 20 | 1.15 | 1.15 (0.964 to 1.373) | 0.12 |
| CigarettesPerDay | IVW | 20 | 1.176 | 1.176 (1.01 to 1.37) | 0.036 |
| CigarettesPerDay | Simple mode | 20 | 1.061 | 1.061 (0.76 to 1.481) | 0.732 |
| CigarettesPerDay | Weighted mode | 20 | 1.134 | 1.134 (0.956 to 1.344) | 0.164 |
| DrinksPerWeek | MR Egger | 32 | 0.648 | 0.648 (0.344 to 1.222) | 0.19 |
| DrinksPerWeek | Weighted median | 32 | 0.721 | 0.721 (0.417 to 1.247) | 0.241 |
| DrinksPerWeek | IVW | 32 | 1.277 | 1.277 (0.839 to 1.944) | 0.254 |
| DrinksPerWeek | Simple mode | 32 | 3.54 | 3.54 (0.868 to 14.437) | 0.088 |
| DrinksPerWeek | Weighted mode | 32 | 0.718 | 0.718 (0.421 to 1.224) | 0.233 |
| educational level | MR Egger | 262 | 0.548 | 0.548 (0.266 to 1.126) | 0.103 |
| educational level | Weighted median | 262 | 1.295 | 1.295 (0.982 to 1.706) | 0.067 |
| educational level | IVW | 262 | 1.311 | 1.311 (1.092 to 1.574) | 0.004 |
| educational level | Simple mode | 262 | 1.737 | 1.737 (0.68 to 4.44) | 0.25 |
| educational level | Weighted mode | 262 | 1.491 | 1.491 (0.697 to 3.189) | 0.304 |

**Additional file 4. Table S3. Results of associations between modifiable risk factors and paternal AD.**

| Exposures | Methods | Nsnps | OR | OR (95% CI) | pval |
| --- | --- | --- | --- | --- | --- |
| hypertension | MR Egger | 138 | 1.149 | 1.149 (0.201 to 6.553) | 0.876 |
| hypertension | Weighted median | 138 | 1.657 | 1.657 (0.731 to 3.76) | 0.227 |
| hypertension | IVW | 138 | 2.242 | 2.242 (1.257 to 4) | 0.006 |
| hypertension | Simple mode | 138 | 0.942 | 0.942 (0.126 to 7.05) | 0.954 |
| hypertension | Weighted mode | 138 | 1.4 | 1.4 (0.294 to 6.678) | 0.673 |
| SBP | MR Egger | 380 | 0.987 | 0.987 (0.962 to 1.013) | 0.32 |
| SBP | Weighted median | 380 | 0.993 | 0.993 (0.978 to 1.009) | 0.397 |
| SBP | IVW | 380 | 0.99 | 0.99 (0.98 to 1) | 0.044 |
| SBP | Simple mode | 380 | 1.027 | 1.027 (0.977 to 1.078) | 0.296 |
| SBP | Weighted mode | 380 | 1.004 | 1.004 (0.972 to 1.037) | 0.808 |
| DBP | MR Egger | 374 | 0.988 | 0.988 (0.949 to 1.028) | 0.537 |
| DBP | Weighted median | 374 | 0.986 | 0.986 (0.962 to 1.011) | 0.276 |
| DBP | IVW | 374 | 0.977 | 0.977 (0.961 to 0.993) | 0.005 |
| DBP | Simple mode | 374 | 0.997 | 0.997 (0.925 to 1.074) | 0.935 |
| DBP | Weighted mode | 374 | 0.988 | 0.988 (0.941 to 1.038) | 0.635 |
| PP | MR Egger | 326 | 0.982 | 0.982 (0.942 to 1.024) | 0.4 |
| PP | Weighted median | 326 | 1 | 1 (0.976 to 1.024) | 0.992 |
| PP | IVW | 326 | 0.994 | 0.994 (0.978 to 1.011) | 0.49 |
| PP | Simple mode | 326 | 1.017 | 1.017 (0.944 to 1.096) | 0.663 |
| PP | Weighted mode | 326 | 1.032 | 1.032 (0.969 to 1.1) | 0.329 |
| dyslipidemia | MR Egger | 46 | 36.898 | 36.898 (0.331 to 4110.885) | 0.141 |
| dyslipidemia | Weighted median | 46 | 0.353 | 0.353 (0.075 to 1.651) | 0.186 |
| dyslipidemia | IVW | 46 | 7.769 | 7.769 (0.666 to 90.579) | 0.102 |
| dyslipidemia | Simple mode | 46 | 0.255 | 0.255 (0.019 to 3.482) | 0.311 |
| dyslipidemia | Weighted mode | 46 | 0.392 | 0.392 (0.079 to 1.943) | 0.258 |
| HDL | MR Egger | 85 | 1.474 | 1.474 (0.705 to 3.083) | 0.305 |
| HDL | Weighted median | 85 | 0.969 | 0.969 (0.784 to 1.199) | 0.775 |
| HDL | IVW | 85 | 0.795 | 0.795 (0.537 to 1.176) | 0.251 |
| HDL | Simple mode | 85 | 0.882 | 0.882 (0.614 to 1.266) | 0.497 |
| HDL | Weighted mode | 85 | 0.914 | 0.914 (0.748 to 1.117) | 0.384 |
| LDL | MR Egger | 44 | 4.127 | 4.127 (1.889 to 9.013) | 0.001 |
| LDL | Weighted median | 44 | 0.91 | 0.91 (0.732 to 1.132) | 0.399 |
| LDL | IVW | 44 | 1.914 | 1.914 (1.17 to 3.132) | 0.01 |
| LDL | Simple mode | 44 | 0.867 | 0.867 (0.602 to 1.247) | 0.446 |
| LDL | Weighted mode | 44 | 0.881 | 0.881 (0.708 to 1.095) | 0.26 |
| triglyceride | MR Egger | 54 | 1.236 | 1.236 (0.779 to 1.961) | 0.373 |
| triglyceride | Weighted median | 54 | 0.962 | 0.962 (0.755 to 1.225) | 0.752 |
| triglyceride | IVW | 54 | 1.119 | 1.119 (0.839 to 1.492) | 0.443 |
| triglyceride | Simple mode | 54 | 0.89 | 0.89 (0.587 to 1.348) | 0.584 |
| triglyceride | Weighted mode | 54 | 0.961 | 0.961 (0.78 to 1.184) | 0.71 |
| diabetes | MR Egger | 34 | 0.303 | 0.303 (0 to 204.761) | 0.722 |
| diabetes | Weighted median | 34 | 3.604 | 3.604 (0.139 to 93.541) | 0.44 |
| diabetes | IVW | 34 | 0.287 | 0.287 (0.019 to 4.232) | 0.363 |
| diabetes | Simple mode | 34 | 3.419 | 3.419 (0.005 to 2489.528) | 0.717 |
| diabetes | Weighted mode | 34 | 5.202 | 5.202 (0.16 to 169.17) | 0.36 |
| fasting glucose | MR Egger | 64 | 1.049 | 1.049 (0.53 to 2.076) | 0.891 |
| fasting glucose | Weighted median | 64 | 0.933 | 0.933 (0.563 to 1.547) | 0.789 |
| fasting glucose | IVW | 64 | 0.891 | 0.891 (0.62 to 1.28) | 0.531 |
| fasting glucose | Simple mode | 64 | 1.292 | 1.292 (0.519 to 3.22) | 0.584 |
| fasting glucose | Weighted mode | 64 | 1.014 | 1.014 (0.648 to 1.589) | 0.951 |
| fasting insulin | MR Egger | 33 | 0.616 | 0.616 (0.079 to 4.833) | 0.648 |
| fasting insulin | Weighted median | 33 | 0.773 | 0.773 (0.328 to 1.821) | 0.556 |
| fasting insulin | IVW | 33 | 0.815 | 0.815 (0.42 to 1.582) | 0.546 |
| fasting insulin | Simple mode | 33 | 1.257 | 1.257 (0.27 to 5.856) | 0.773 |
| fasting insulin | Weighted mode | 33 | 1.057 | 1.057 (0.289 to 3.867) | 0.934 |
| 2hGlu | MR Egger | 10 | 1.162 | 1.162 (0.604 to 2.235) | 0.665 |
| 2hGlu | Weighted median | 10 | 0.995 | 0.995 (0.731 to 1.354) | 0.975 |
| 2hGlu | IVW | 10 | 1.02 | 1.02 (0.801 to 1.299) | 0.874 |
| 2hGlu | Simple mode | 10 | 0.778 | 0.778 (0.428 to 1.416) | 0.433 |
| 2hGlu | Weighted mode | 10 | 0.79 | 0.79 (0.45 to 1.386) | 0.432 |
| HbA1c | MR Egger | 66 | 0.864 | 0.864 (0.388 to 1.925) | 0.722 |
| HbA1c | Weighted median | 66 | 0.633 | 0.633 (0.332 to 1.205) | 0.164 |
| HbA1c | IVW | 66 | 1.09 | 1.09 (0.702 to 1.693) | 0.701 |
| HbA1c | Simple mode | 66 | 0.528 | 0.528 (0.134 to 2.076) | 0.364 |
| HbA1c | Weighted mode | 66 | 0.644 | 0.644 (0.327 to 1.269) | 0.208 |
| insomnia | MR Egger | 34 | 1.238 | 1.238 (0.068 to 22.484) | 0.886 |
| insomnia | Weighted median | 34 | 0.843 | 0.843 (0.23 to 3.091) | 0.797 |
| insomnia | IVW | 34 | 0.891 | 0.891 (0.365 to 2.174) | 0.8 |
| insomnia | Simple mode | 34 | 0.862 | 0.862 (0.078 to 9.55) | 0.905 |
| insomnia | Weighted mode | 34 | 0.961 | 0.961 (0.118 to 7.841) | 0.97 |
| overall-sleep | MR Egger | 59 | 0.72 | 0.72 (0.151 to 3.423) | 0.681 |
| overall-sleep | Weighted median | 59 | 0.985 | 0.985 (0.526 to 1.845) | 0.964 |
| overall-sleep | IVW | 59 | 0.858 | 0.858 (0.563 to 1.306) | 0.475 |
| overall-sleep | Simple mode | 59 | 0.946 | 0.946 (0.251 to 3.564) | 0.935 |
| overall-sleep | Weighted mode | 59 | 0.995 | 0.995 (0.319 to 3.102) | 0.993 |
| long-sleep | MR Egger | 5 | 64111.83 | 64111.826 (0.205 to 20015213169.935) | 0.185 |
| long-sleep | Weighted median | 5 | 0.118 | 0.118 (0 to 56.115) | 0.496 |
| long-sleep | IVW | 5 | 0.161 | 0.161 (0.001 to 26.96) | 0.485 |
| long-sleep | Simple mode | 5 | 0.013 | 0.013 (0 to 315.234) | 0.447 |
| long-sleep | Weighted mode | 5 | 0.07 | 0.07 (0 to 380.764) | 0.577 |
| short-sleep | MR Egger | 23 | 123.546 | 123.546 (0.016 to 942878.196) | 0.303 |
| short-sleep | Weighted median | 23 | 1.324 | 1.324 (0.117 to 15.037) | 0.821 |
| short-sleep | IVW | 23 | 2.464 | 2.464 (0.402 to 15.094) | 0.33 |
| short-sleep | Simple mode | 23 | 0.231 | 0.231 (0.004 to 14.503) | 0.495 |
| short-sleep | Weighted mode | 23 | 0.406 | 0.406 (0.007 to 23.221) | 0.666 |
| body mass index | MR Egger | 715 | 0.914 | 0.914 (0.746 to 1.12) | 0.387 |
| body mass index | Weighted median | 715 | 0.929 | 0.929 (0.799 to 1.079) | 0.334 |
| body mass index | IVW | 715 | 0.923 | 0.923 (0.84 to 1.015) | 0.097 |
| body mass index | Simple mode | 715 | 0.718 | 0.718 (0.479 to 1.076) | 0.109 |
| body mass index | Weighted mode | 715 | 0.859 | 0.859 (0.659 to 1.118) | 0.258 |
| coffee intake | MR Egger | 20 | 1.235 | 1.235 (0.25 to 6.088) | 0.798 |
| coffee intake | Weighted median | 20 | 0.493 | 0.493 (0.184 to 1.323) | 0.16 |
| coffee intake | IVW | 20 | 0.953 | 0.953 (0.445 to 2.045) | 0.902 |
| coffee intake | Simple mode | 20 | 0.359 | 0.359 (0.056 to 2.31) | 0.295 |
| coffee intake | Weighted mode | 20 | 0.487 | 0.487 (0.148 to 1.606) | 0.252 |
| tea intake | MR Egger | 14 | 0.406 | 0.406 (0.101 to 1.627) | 0.227 |
| tea intake | Weighted median | 14 | 1.089 | 1.089 (0.416 to 2.85) | 0.862 |
| tea intake | IVW | 14 | 1.693 | 1.693 (0.829 to 3.456) | 0.148 |
| tea intake | Simple mode | 14 | 8.482 | 8.482 (1.069 to 67.295) | 0.064 |
| tea intake | Weighted mode | 14 | 0.563 | 0.563 (0.169 to 1.883) | 0.368 |
| SmokingInitiation | MR Egger | 73 | 0.351 | 0.351 (0.076 to 1.625) | 0.185 |
| SmokingInitiation | Weighted median | 73 | 0.83 | 0.83 (0.569 to 1.213) | 0.336 |
| SmokingInitiation | IVW | 73 | 0.911 | 0.911 (0.675 to 1.23) | 0.542 |
| SmokingInitiation | Simple mode | 73 | 0.894 | 0.894 (0.357 to 2.241) | 0.812 |
| SmokingInitiation | Weighted mode | 73 | 0.797 | 0.797 (0.387 to 1.641) | 0.541 |
| CigarettesPerDay | MR Egger | 20 | 0.846 | 0.846 (0.599 to 1.193) | 0.352 |
| CigarettesPerDay | Weighted median | 20 | 1.075 | 1.075 (0.841 to 1.374) | 0.561 |
| CigarettesPerDay | IVW | 20 | 1.092 | 1.092 (0.885 to 1.347) | 0.41 |
| CigarettesPerDay | Simple mode | 20 | 1.138 | 1.138 (0.681 to 1.903) | 0.627 |
| CigarettesPerDay | Weighted mode | 20 | 1.03 | 1.03 (0.788 to 1.347) | 0.829 |
| DrinksPerWeek | MR Egger | 32 | 1.26 | 1.26 (0.474 to 3.349) | 0.647 |
| DrinksPerWeek | Weighted median | 32 | 1.436 | 1.436 (0.667 to 3.093) | 0.355 |
| DrinksPerWeek | IVW | 32 | 0.945 | 0.945 (0.528 to 1.692) | 0.849 |
| DrinksPerWeek | Simple mode | 32 | 2.112 | 2.112 (0.354 to 12.588) | 0.418 |
| DrinksPerWeek | Weighted mode | 32 | 1.502 | 1.502 (0.711 to 3.173) | 0.294 |
| educational level | MR Egger | 262 | 1.193 | 1.193 (0.432 to 3.294) | 0.733 |
| educational level | Weighted median | 262 | 1.031 | 1.031 (0.722 to 1.471) | 0.868 |
| educational level | IVW | 262 | 1.208 | 1.208 (0.937 to 1.558) | 0.146 |
| educational level | Simple mode | 262 | 0.998 | 0.998 (0.313 to 3.177) | 0.997 |
| educational level | Weighted mode | 262 | 0.845 | 0.845 (0.328 to 2.181) | 0.728 |

**Additional file 5. Table S4. Sensitivity analysis of instrument effects.**

| Risk factors | N SNPs | Heterogeneity analysis | | | | Pleiotropy analysis | | | Leave-one-out |
| --- | --- | --- | --- | --- | --- | --- | --- | --- | --- |
|  |  | Method | Q | DF | P | Egger intercept | SE | P |  |
| (Late-on-set AD) |  |  |  |  |  |  |  |  |  |
| Educational level | 331 | MR Egger  IVW weighted | 267.39  268.51 | 260  261 | 0.363  0.361 | 4.34E-03 | 4.16E-03 | 0.297 | NA |
| BMI | 715 | MR Egger  IVW | 939.15  940.13 | 713  714 | 2.26E-08  2.31E-08 | -1.33E-03 | 1.54E-03 | 0.387 | NA |
| DBP | 398 | MR Egger  IVW | 524.27  526.63 | 396  397 | 1.56E-05  1.33E-05 | 3.25E-03 | 2.43E-03 | 0.182 | Fig.a |
| LDL | 76 | MR Egger  IVW | 600.22  652.27 | 74  75 | 2.14E-83  6.29E-93 | -2.30E-02 | 9.06E-03 | 0.013 | rs7254892 |
| (Paternal AD) |  |  |  |  |  |  |  |  |  |
| LDL | 76 | MR Egger  IVW | 248.44  268.06 | 74  75 | 1.05E-20  1.65E-23 | 2.26E-02 | 9.35E-03 | 0.018 | NA |
| (Maternal AD) |  |  |  |  |  |  |  |  |  |
| LDL | 76 | MR Egger  IVW | 508.62  543.51 | 74  75 | 4.39E-66  3.42E-72 | -2.20E-02 | 9.77E-03 | 0.027 | NA |
| BMI | 715 | MR Egger  IVW | 767.57  769.32 | 713  714 | 0.077  0.074 | 2.16E-03 | 1.69E-03 | 0.203 | NA |
| (PD) |  |  |  |  |  |  |  |  |  |
| Short sleep-duration | 25 | MR Egger  IVW | 27.61  27.81 | 21  22 | 0.151  0.182 | 0.010 | 0.026 | 0.702 | NA |
| Long sleep-duration | 6 | MR Egger  IVW | 0.927  3.233 | 3  4 | 0.819  0.520 | 0.048 | 0.032 | 0.226 | NA |
| (ALS) |  |  |  |  |  |  |  |  |  |
| Educational level | 374 | MR Egger  IVW | 309.74  311.68 | 251  251 | 0.007  0.006 | 0.006 | 0.004 | 0.212 | NA |
| LDL | 77 | MR Egger  IVW | 113.95  113.96 | 75  76 | 2.51E-03  3.17E-03 | -9.86E-05 | 3.61E-03 | 0.978 | NA |
|  |  |  |  |  |  |  |  |  |  |

Abbreviations: AD, Alzheimer’s disease; PD, Parkinson’s disease; ALS, amyotrophic lateral sclerosis; LDL, low-density lipoprotein cholesterol;

SBP, systolic blood pressure; DBP, diastolic blood pressure; BMI, body mass index. MVPA, mild to vigorous physical activity; DF, Degree of freedom

MR, Mendelian randomization; IVW, inverse variance weighted; SE, standard error;

**Additional file 6 Genome-wide significant and independent SNPs that were used as instruments.**

**Table S5. Genome-wide significant and independent SNPs that were used as instruments for type 2 diabetes**

| SNP | Effect | other | eaf. | beta | se | pval |
| --- | --- | --- | --- | --- | --- | --- |
| rs2476601 | A | G | 0.102414 | -0.00411 | 0.000746 | 3.52E-08 |
| rs2487569 | A | T | 0.110721 | 0.004492 | 0.000729 | 7.11E-10 |
| rs340882 | C | G | 0.378507 | 0.0028 | 0.000469 | 2.45E-09 |
| rs2972145 | T | C | 0.353266 | 0.003179 | 0.000474 | 1.94E-11 |
| rs1260326 | T | C | 0.393157 | 0.003139 | 0.000464 | 1.26E-11 |
| rs77101426 | G | A | 0.073927 | -0.00526 | 0.000871 | 1.51E-09 |
| rs6807753 | T | C | 0.446639 | -0.0025 | 0.000455 | 4.27E-08 |
| rs6765808 | C | T | 0.314144 | 0.004927 | 0.000488 | 5.77E-24 |
| rs1496653 | A | G | 0.204451 | -0.00364 | 0.000562 | 9.58E-11 |
| rs73077175 | G | A | 0.333492 | -0.00269 | 0.000481 | 2.21E-08 |
| rs112674299 | C | T | 0.132656 | -0.00369 | 0.000668 | 3.24E-08 |
| rs1046319 | C | T | 0.281104 | 0.004482 | 0.000505 | 6.85E-19 |
| rs80134320 | G | T | 0.054271 | 0.00586 | 0.001011 | 6.79E-09 |
| rs6885132 | C | G | 0.099899 | -0.00471 | 0.000768 | 8.62E-10 |
| rs13179413 | C | T | 0.285061 | 0.002958 | 0.000513 | 8.22E-09 |
| rs34872471 | T | C | 0.291254 | 0.012125 | 0.000499 | ###### |
| rs1317548 | G | A | 0.203339 | 0.00339 | 0.000568 | 2.33E-09 |
| rs697239 | T | C | 0.458213 | -0.00295 | 0.000455 | 8.26E-11 |
| rs11187139 | T | C | 0.408705 | -0.00391 | 0.000461 | 2.17E-17 |
| rs5215 | C | T | 0.358269 | -0.00297 | 0.000472 | 3.08E-10 |
| rs2237895 | A | C | 0.415177 | 0.003838 | 0.000459 | 6.10E-17 |
| rs11602873 | A | T | 0.157365 | -0.00417 | 0.000622 | 2.00E-11 |
| rs10830963 | C | G | 0.27506 | 0.003436 | 0.000507 | 1.20E-11 |
| rs76895963 | T | G | 0.02102 | -0.0156 | 0.001742 | 3.42E-19 |
| rs2258238 | A | T | 0.104907 | 0.004521 | 0.000742 | 1.11E-09 |
| rs7766070 | C | A | 0.263436 | 0.004493 | 0.000514 | 2.35E-18 |
| rs3104414 | G | A | 0.337256 | 0.003468 | 0.000482 | 6.44E-13 |
| rs9273363 | C | A | 0.303844 | 0.006651 | 0.000493 | 1.86E-41 |
| rs9379084 | G | A | 0.115387 | -0.00546 | 0.000731 | 8.32E-14 |
| rs4719433 | T | C | 0.450981 | 0.003126 | 0.000455 | 6.58E-12 |
| rs849135 | G | A | 0.498618 | -0.0038 | 0.000453 | 5.12E-17 |
| rs3802177 | G | A | 0.311003 | -0.00427 | 0.00049 | 2.73E-18 |
| rs13262861 | C | A | 0.1737 | -0.00425 | 0.000606 | 2.22E-12 |
| rs77684335 | A | G | 0.285908 | 0.002922 | 0.000511 | 1.06E-08 |
| rs10965248 | T | C | 0.177068 | -0.00575 | 0.000595 | 4.43E-22 |
| rs2796441 | G | A | 0.419932 | -0.00344 | 0.000458 | 6.45E-14 |
| rs522317 | C | T | 0.433057 | 0.002747 | 0.000458 | 1.97E-09 |
| rs34715063 | T | C | 0.12963 | 0.003851 | 0.000678 | 1.36E-08 |
| rs1421085 | T | C | 0.401856 | 0.003922 | 0.000462 | 2.16E-17 |
| rs2917677 | C | T | 0.410099 | -0.00294 | 0.000462 | 1.86E-10 |
| rs11657964 | A | G | 0.397883 | -0.00336 | 0.000464 | 4.25E-13 |
| rs3111316 | G | A | 0.414579 | 0.002607 | 0.000459 | 1.36E-08 |
| rs8106861 | A | G | 0.277068 | 0.002882 | 0.000508 | 1.38E-08 |

**Table S6. Genome-wide significant and independent SNPs that were used as instruments for fasting glucose**

| SNP | effect | other | eaf | beta | se. | pval. |
| --- | --- | --- | --- | --- | --- | --- |
| rs12784552 | A | G | 0.924 | 0.0329 | 0.003 | 2.86E-31 |
| rs7903146 | T | C | 0.307 | 0.0259 | 0.0019 | 2.00E-35 |
| rs2839671 | A | G | 0.164 | -0.016 | 0.0022 | 8.38E-14 |
| rs7095788 | T | C | 0.358 | -0.0106 | 0.0018 | 1.98E-09 |
| rs3842753 | T | G | 0.28 | 0.0134 | 0.0022 | 2.84E-09 |
| rs10838524 | A | G | 0.48 | 0.0238 | 0.0016 | 1.56E-40 |
| rs10838693 | C | G | 0.314 | 0.0177 | 0.0018 | 3.44E-23 |
| rs174583 | T | C | 0.375 | -0.0168 | 0.0017 | 3.37E-22 |
| rs11603349 | T | C | 0.832 | 0.0236 | 0.0022 | 3.12E-25 |
| rs11020124 | T | C | 0.722 | -0.0599 | 0.0019 | ###### |
| rs192701415 | A | G | 0.904 | 0.0201 | 0.0034 | 8.02E-09 |
| rs6489811 | A | G | 0.488 | -0.011 | 0.0018 | 3.27E-09 |
| rs11610045 | A | G | 0.454 | 0.0144 | 0.0019 | 3.26E-13 |
| rs2657879 | A | G | 0.798 | -0.0119 | 0.0022 | 7.33E-09 |
| rs4760278 | A | C | 0.181 | -0.011 | 0.002 | 3.33E-08 |
| rs6538804 | C | G | 0.623 | 0.0142 | 0.0019 | 9.41E-14 |
| rs11619319 | A | G | 0.766 | -0.0173 | 0.002 | 3.41E-20 |
| rs12888855 | A | C | 0.189 | -0.0135 | 0.002 | 6.02E-12 |
| rs35889227 | T | G | 0.617 | -0.013 | 0.0019 | 3.37E-10 |
| rs17270243 | A | G | 0.76 | -0.0104 | 0.0021 | 3.62E-08 |
| rs7163757 | T | C | 0.433 | -0.0217 | 0.0016 | 2.64E-36 |
| rs12898997 | T | C | 0.598 | -0.0098 | 0.0017 | 4.64E-09 |
| rs7178572 | A | G | 0.321 | -0.0121 | 0.0018 | 7.09E-10 |
| rs6598541 | A | G | 0.352 | 0.0114 | 0.0017 | 4.12E-12 |
| rs2238435 | C | G | 0.381 | 0.0112 | 0.0019 | 3.82E-09 |
| rs7256920 | A | G | 0.491 | -0.0104 | 0.0018 | 2.75E-09 |
| rs6662924 | A | C | 0.198 | 0.0143 | 0.0023 | 3.34E-10 |
| rs78132593 | A | C | 0.203 | -0.0147 | 0.0022 | 2.60E-10 |
| rs2075423 | T | G | 0.376 | -0.0161 | 0.0017 | 3.18E-21 |
| rs348330 | A | G | 0.631 | -0.0122 | 0.002 | 3.04E-10 |
| rs6113722 | A | G | 0.066 | -0.0424 | 0.0044 | 7.66E-25 |
| rs17265513 | T | C | 0.796 | -0.0158 | 0.0021 | 5.10E-14 |
| rs39713 | T | C | 0.062 | -0.0169 | 0.0031 | 1.77E-08 |
| rs7584277 | A | G | 0.075 | 0.0266 | 0.0036 | 1.61E-14 |
| rs180935712 | A | G | 0.011 | 0.057 | 0.0082 | 2.12E-11 |
| rs140809953 | A | G | 0.024 | 0.0369 | 0.0073 | 2.11E-08 |
| rs13431652 | T | C | 0.683 | 0.0693 | 0.0018 | ###### |
| rs13389076 | A | G | 0.034 | 0.0609 | 0.0049 | 1.72E-36 |
| rs1057394 | A | G | 0.625 | -0.0124 | 0.0018 | 1.91E-12 |
| rs1260326 | T | C | 0.413 | -0.0282 | 0.0017 | 4.48E-65 |
| rs111884754 | A | C | 0.055 | -0.0248 | 0.0035 | 7.81E-15 |
| rs189548 | A | G | 0.723 | -0.0123 | 0.002 | 2.81E-09 |
| rs11708067 | A | G | 0.823 | 0.0281 | 0.002 | 1.63E-43 |
| rs16851397 | A | G | 0.955 | 0.0327 | 0.0042 | 1.26E-12 |
| rs17437560 | T | C | 0.11 | -0.0175 | 0.0032 | 3.33E-08 |
| rs1604038 | T | C | 0.288 | -0.0198 | 0.0018 | 4.47E-28 |
| rs6808574 | T | C | 0.394 | -0.0127 | 0.0017 | 7.21E-14 |
| rs4862423 | T | C | 0.401 | 0.0123 | 0.0019 | 4.45E-10 |
| rs157512 | T | C | 0.73 | 0.0134 | 0.0021 | 5.43E-10 |
| rs7708285 | A | G | 0.686 | -0.0133 | 0.0019 | 1.25E-09 |
| rs1820176 | T | C | 0.704 | 0.0247 | 0.002 | 1.91E-34 |
| rs12055786 | T | C | 0.384 | 0.012 | 0.0017 | 1.17E-11 |
| rs9348441 | A | T | 0.272 | 0.0176 | 0.0018 | 4.40E-20 |
| rs10305457 | T | C | 0.065 | 0.0235 | 0.0032 | 1.21E-14 |
| rs3778321 | A | G | 0.176 | -0.0186 | 0.0021 | 3.16E-17 |
| rs17168486 | T | C | 0.176 | 0.028 | 0.0021 | 4.17E-36 |
| rs10487796 | A | T | 0.484 | -0.0261 | 0.0016 | 4.62E-52 |
| rs2979422 | T | C | 0.869 | -0.0243 | 0.0024 | 3.85E-26 |
| rs878521 | A | G | 0.249 | 0.0549 | 0.002 | ###### |
| rs58925536 | T | C | 0.032 | 0.0306 | 0.0053 | 5.82E-09 |
| rs194518 | A | G | 0.52 | 0.0102 | 0.0018 | 8.76E-09 |
| rs9650069 | T | C | 0.284 | -0.0286 | 0.0018 | 8.31E-58 |
| rs12541643 | T | C | 0.479 | 0.0118 | 0.0019 | 4.51E-09 |
| rs7012637 | A | G | 0.47 | -0.018 | 0.0017 | 9.75E-25 |
| rs896854 | T | C | 0.458 | 0.0099 | 0.0016 | 5.61E-09 |
| rs16913693 | T | G | 0.971 | 0.0394 | 0.0049 | 2.82E-16 |
| rs507666 | A | G | 0.189 | 0.0164 | 0.0021 | 6.99E-17 |
| rs3829109 | A | G | 0.276 | -0.0163 | 0.002 | 1.09E-15 |
| rs10811660 | A | G | 0.165 | -0.0223 | 0.0022 | 7.94E-25 |
| rs10974438 | A | C | 0.62 | -0.0198 | 0.0017 | 9.85E-31 |

**Table S7. Genome-wide significant and independent SNPs that were used as instruments for fasting insulin**

| SNP | effect | other | eaf. | beta | se | pval |
| --- | --- | --- | --- | --- | --- | --- |
| rs7903146 | T | C | 0.307 | -0.0116 | 0.0021 | 1.24E-09 |
| rs118164457 | T | C | 0.963 | -0.0345 | 0.0057 | 3.86E-10 |
| rs2845885 | T | C | 0.931 | -0.0204 | 0.0039 | 1.18E-08 |
| rs860598 | A | G | 0.824 | 0.0177 | 0.0025 | 6.88E-12 |
| rs7133378 | A | G | 0.339 | -0.0127 | 0.002 | 6.00E-11 |
| rs6487237 | A | C | 0.79 | 0.0154 | 0.0026 | 4.68E-09 |
| rs1351394 | T | C | 0.471 | -0.0111 | 0.0018 | 2.71E-09 |
| rs12454712 | T | C | 0.602 | 0.0142 | 0.0025 | 1.78E-09 |
| rs731839 | A | G | 0.658 | -0.0121 | 0.0019 | 3.87E-11 |
| rs6674544 | A | G | 0.574 | 0.0177 | 0.002 | 6.97E-21 |
| rs1206760 | A | G | 0.522 | -0.0112 | 0.0019 | 8.82E-10 |
| rs13389219 | T | C | 0.409 | -0.0199 | 0.0019 | 5.84E-28 |
| rs2943646 | A | G | 0.377 | -0.025 | 0.0019 | 8.47E-39 |
| rs1260326 | T | C | 0.413 | -0.0231 | 0.0019 | 8.42E-38 |
| rs5017305 | A | T | 0.236 | 0.0137 | 0.0026 | 1.07E-08 |
| rs17036126 | T | C | 0.129 | 0.0209 | 0.003 | 1.28E-10 |
| rs11708067 | A | G | 0.823 | -0.0135 | 0.0023 | 1.30E-09 |
| rs35000407 | T | G | 0.883 | 0.0258 | 0.0028 | 1.50E-21 |
| rs62271373 | A | T | 0.059 | 0.0256 | 0.0048 | 1.60E-08 |
| rs10865959 | C | G | 0.3 | 0.0138 | 0.0022 | 1.99E-08 |
| rs17331151 | T | C | 0.106 | -0.0162 | 0.0031 | 1.52E-08 |
| rs9884482 | T | C | 0.608 | -0.0125 | 0.0019 | 2.88E-11 |
| rs11727676 | T | C | 0.916 | -0.0203 | 0.0039 | 2.90E-08 |
| rs6855363 | T | C | 0.653 | 0.0125 | 0.002 | 4.04E-08 |
| rs3775380 | A | G | 0.5 | -0.0119 | 0.0018 | 1.48E-11 |
| rs10050393 | T | C | 0.54 | 0.009 | 0.0019 | 4.84E-08 |
| rs4865796 | A | G | 0.707 | 0.0165 | 0.002 | 7.33E-17 |
| rs459193 | A | G | 0.285 | -0.0181 | 0.0021 | 1.12E-18 |
| rs1474696 | A | G | 0.524 | -0.0147 | 0.0018 | 3.02E-16 |
| rs73013411 | A | C | 0.12 | -0.018 | 0.0032 | 2.08E-08 |
| rs116141873 | T | G | 0.032 | 0.0428 | 0.0059 | 1.42E-11 |
| rs2780215 | A | G | 0.958 | 0.0392 | 0.0063 | 1.06E-09 |
| rs6905288 | A | G | 0.602 | 0.0112 | 0.0019 | 7.75E-09 |
| rs972283 | A | G | 0.456 | -0.0105 | 0.0019 | 1.09E-08 |
| rs2108349 | A | G | 0.686 | -0.0115 | 0.002 | 1.13E-08 |
| rs13258890 | T | C | 0.748 | 0.0128 | 0.0025 | 2.77E-08 |
| rs7012814 | A | G | 0.471 | -0.0219 | 0.0019 | 8.34E-30 |
| rs75179845 | T | C | 0.921 | -0.0216 | 0.0035 | 6.05E-11 |

**Table S8. Genome-wide significant and independent SNPs that were used as instruments for 2h glucose**

| SNP | effect | other | eaf. | beta. | se. | pval |
| --- | --- | --- | --- | --- | --- | --- |
| rs7903146 | T | C | 0.307 | 0.0854 | 0.0087 | 2.79E-26 |
| rs4148646 | C | G | 0.336 | 0.0397 | 0.0078 | 4.39E-08 |
| rs2649999 | T | C | 0.359 | 0.0498 | 0.0082 | 2.01E-10 |
| rs112824462 | A | G | 0.276 | -0.0584 | 0.0102 | 6.73E-09 |
| rs17271305 | A | G | 0.591 | -0.0587 | 0.0077 | 2.88E-14 |
| rs117643180 | A | C | 0.033 | 0.234 | 0.0327 | 7.31E-14 |
| rs1800437 | C | G | 0.208 | 0.1004 | 0.0099 | 4.79E-26 |
| rs12692738 | T | C | 0.735 | 0.0486 | 0.009 | 2.72E-08 |
| rs1260326 | T | C | 0.413 | 0.0486 | 0.0078 | 5.93E-12 |
| rs11708067 | A | G | 0.823 | 0.0872 | 0.0093 | 1.98E-22 |
| rs150111048 | A | G | 0.751 | -0.0596 | 0.0089 | 1.14E-11 |
| rs878521 | A | G | 0.249 | 0.099 | 0.0094 | 1.25E-28 |
| rs2126259 | T | C | 0.091 | -0.0716 | 0.0121 | 2.98E-10 |
| rs550057 | T | C | 0.286 | 0.0526 | 0.0085 | 3.62E-11 |

**Table S9. Genome-wide significant and independent SNPs that were used as instruments for HbA1c**

| SNP | effect | other | eaf | beta. | se. | pval |
| --- | --- | --- | --- | --- | --- | --- |
| rs7903146 | T | C | 0.307 | 0.0133 | 0.0014 | 1.04E-22 |
| rs11257655 | T | C | 0.241 | 0.011 | 0.0016 | 1.91E-13 |
| rs2102339 | T | C | 0.334 | -0.0087 | 0.0014 | 3.42E-10 |
| rs16926246 | T | C | 0.136 | -0.0727 | 0.0021 | ###### |
| rs7127313 | T | C | 0.336 | 0.0066 | 0.0013 | 4.85E-08 |
| rs608793 | T | C | 0.479 | 0.0065 | 0.0013 | 4.55E-08 |
| rs4980325 | T | G | 0.532 | 0.0108 | 0.0014 | 4.70E-14 |
| rs11039154 | T | C | 0.277 | -0.0087 | 0.0014 | 3.11E-09 |
| rs174559 | A | G | 0.285 | -0.0106 | 0.0014 | 3.31E-13 |
| rs10830963 | C | G | 0.714 | -0.0197 | 0.0015 | 1.54E-36 |
| rs360147 | T | C | 0.264 | -0.0086 | 0.0015 | 2.08E-09 |
| rs10774624 | A | G | 0.525 | 0.0093 | 0.0013 | 4.17E-14 |
| rs117233107 | A | G | 0.02 | -0.047 | 0.0072 | 8.45E-11 |
| rs4760682 | A | C | 0.817 | 0.0164 | 0.0018 | 3.20E-20 |
| rs76533333 | A | G | 0.913 | -0.0265 | 0.0025 | 2.81E-29 |
| rs1278769 | A | G | 0.231 | -0.0091 | 0.0015 | 5.52E-12 |
| rs1535464 | A | G | 0.212 | -0.0086 | 0.0017 | 1.11E-08 |
| rs151165 | A | T | 0.397 | 0.0079 | 0.0014 | 2.04E-09 |
| rs10151436 | A | T | 0.89 | 0.013 | 0.0021 | 3.85E-11 |
| rs452306 | T | C | 0.627 | -0.0098 | 0.0014 | 5.51E-13 |
| rs11643024 | A | G | 0.303 | 0.0084 | 0.0015 | 7.98E-10 |
| rs7190771 | A | G | 0.332 | 0.0085 | 0.0013 | 6.02E-11 |
| rs11248914 | T | C | 0.698 | 0.0114 | 0.0014 | 1.42E-14 |
| rs7198799 | T | C | 0.281 | 0.0083 | 0.0014 | 4.76E-09 |
| rs837763 | T | C | 0.578 | 0.0176 | 0.0013 | 5.20E-38 |
| rs9914988 | A | G | 0.802 | 0.0125 | 0.0016 | 4.66E-17 |
| rs2748427 | A | G | 0.803 | -0.0307 | 0.0022 | 9.82E-49 |
| rs9909940 | T | C | 0.323 | 0.0322 | 0.0014 | ###### |
| rs28671200 | T | G | 0.646 | 0.0086 | 0.0017 | 1.56E-08 |
| rs17533945 | T | C | 0.582 | -0.0128 | 0.0014 | 1.62E-23 |
| rs10405535 | A | G | 0.29 | 0.0122 | 0.0016 | 6.47E-14 |
| rs267738 | T | G | 0.797 | 0.0109 | 0.0016 | 1.14E-11 |
| rs857725 | T | G | 0.723 | -0.0208 | 0.0014 | 5.43E-55 |
| rs7547793 | A | C | 0.12 | -0.0118 | 0.0021 | 6.61E-09 |
| rs340882 | C | G | 0.42 | -0.0084 | 0.0013 | 1.48E-10 |
| rs2375278 | A | G | 0.176 | 0.0112 | 0.0017 | 1.05E-11 |
| rs1175549 | A | C | 0.786 | 0.0098 | 0.0015 | 7.13E-13 |
| rs737092 | T | C | 0.501 | -0.0073 | 0.0013 | 7.57E-09 |
| rs855791 | A | G | 0.4 | 0.0188 | 0.0013 | 1.34E-56 |
| rs8138197 | A | G | 0.488 | -0.0073 | 0.0014 | 3.54E-08 |
| rs560887 | T | C | 0.306 | -0.0307 | 0.0014 | ###### |
| rs13389076 | A | G | 0.034 | 0.0332 | 0.0038 | 3.04E-18 |
| rs13419763 | T | C | 0.588 | 0.008 | 0.0014 | 5.48E-09 |
| rs12612492 | T | C | 0.148 | 0.0188 | 0.0019 | 1.88E-26 |
| rs1367173 | T | C | 0.106 | -0.0152 | 0.002 | 1.66E-14 |
| rs79403657 | C | G | 0.823 | -0.009 | 0.0017 | 2.03E-08 |
| rs10169706 | T | C | 0.04 | 0.026 | 0.0046 | 1.48E-08 |
| rs12491937 | A | G | 0.555 | 0.009 | 0.0013 | 1.42E-13 |
| rs11719201 | T | C | 0.182 | -0.0129 | 0.0015 | 2.43E-18 |
| rs6804915 | A | C | 0.288 | -0.0108 | 0.0014 | 2.76E-16 |
| rs13089972 | A | T | 0.584 | 0.0111 | 0.0014 | 1.87E-15 |
| rs9818758 | A | G | 0.204 | 0.0131 | 0.0017 | 1.49E-13 |
| rs6798941 | T | C | 0.322 | 0.0086 | 0.0015 | 1.49E-08 |
| rs13134327 | A | G | 0.331 | 0.0144 | 0.0014 | 2.81E-26 |
| rs6877043 | T | C | 0.638 | 0.0085 | 0.0014 | 1.99E-10 |
| rs9376090 | T | C | 0.728 | 0.0247 | 0.0014 | 1.90E-62 |
| rs10946402 | T | G | 0.831 | -0.0101 | 0.0016 | 1.12E-10 |
| rs1800562 | A | G | 0.046 | -0.0383 | 0.0027 | 2.33E-50 |
| rs204995 | A | G | 0.781 | -0.0098 | 0.0018 | 1.93E-09 |
| rs3778321 | A | G | 0.176 | -0.0106 | 0.0016 | 4.18E-11 |
| rs4727979 | A | C | 0.906 | 0.0121 | 0.0024 | 4.61E-08 |
| rs10231021 | A | T | 0.492 | 0.0089 | 0.0013 | 8.69E-14 |
| rs2908277 | A | G | 0.117 | 0.0166 | 0.002 | 1.29E-18 |
| rs2971670 | T | C | 0.181 | 0.0316 | 0.0017 | 5.10E-88 |
| rs13234131 | A | G | 0.876 | -0.0113 | 0.002 | 2.06E-09 |
| rs11558471 | A | G | 0.707 | 0.0151 | 0.0014 | 3.38E-25 |
| rs2001846 | T | C | 0.471 | -0.0069 | 0.0013 | 8.58E-10 |
| rs6474359 | T | C | 0.978 | 0.0427 | 0.0038 | 1.91E-33 |
| rs4737009 | A | G | 0.262 | 0.0228 | 0.0015 | 8.29E-56 |
| rs7042939 | A | G | 0.418 | 0.0102 | 0.0013 | 1.50E-15 |
| rs651007 | T | C | 0.215 | 0.0108 | 0.0015 | 3.28E-15 |
| rs3829109 | A | G | 0.276 | -0.0086 | 0.0015 | 2.68E-08 |
| rs10811661 | T | C | 0.835 | 0.0128 | 0.0017 | 1.74E-14 |
| rs7861647 | T | C | 0.193 | 0.0128 | 0.0016 | 4.50E-14 |
| rs61750929 | T | C | 0.041 | -0.0284 | 0.0029 | 9.49E-24 |

**Table S10. Genome-wide significant and independent SNPs that were used as instruments for dyslipidemia**

| SNP | effect | other | eaf | beta. | se. | pval |
| --- | --- | --- | --- | --- | --- | --- |
| rs12740374 | G | T | 0.221803 | -0.01884 | 0.000902 | 8.26E-97 |
| rs2902491 | A | T | 0.258828 | -0.00501 | 0.000866 | 7.37E-09 |
| rs11580878 | A | G | 0.496166 | -0.00479 | 0.000769 | 4.76E-10 |
| rs193084249 | A | G | 0.02389 | 0.014211 | 0.002511 | 1.53E-08 |
| rs11591147 | G | T | 0.01767 | -0.04867 | 0.002844 | 1.28E-65 |
| rs472495 | G | T | 0.348982 | 0.0061 | 0.000788 | 9.68E-15 |
| rs7534572 | C | G | 0.354373 | 0.007935 | 0.000785 | 4.80E-24 |
| rs6739198 | G | T | 0.120546 | 0.008183 | 0.001152 | 1.23E-12 |
| rs1367117 | G | A | 0.338808 | 0.015953 | 0.000792 | 4.07E-90 |
| rs114562459 | T | G | 0.324716 | 0.00514 | 0.000808 | 2.01E-10 |
| rs1260326 | T | C | 0.393157 | -0.01003 | 0.000767 | 4.70E-39 |
| rs4299376 | G | T | 0.323617 | -0.01116 | 0.000802 | 5.56E-44 |
| rs58691354 | C | T | 0.155579 | 0.006592 | 0.001044 | 2.72E-10 |
| rs1154988 | T | A | 0.226356 | 0.005436 | 0.000897 | 1.33E-09 |
| rs184901863 | G | A | 0.199875 | 0.007701 | 0.001001 | 1.43E-14 |
| rs34707604 | T | C | 0.260537 | 0.005693 | 0.000902 | 2.73E-10 |
| rs4704826 | C | A | 0.36431 | 0.006758 | 0.000779 | 4.19E-18 |
| rs12916 | T | C | 0.399757 | 0.010521 | 0.000766 | 6.44E-43 |
| rs603424 | G | A | 0.168109 | 0.005706 | 0.001003 | 1.29E-08 |
| rs56299331 | C | T | 0.201333 | 0.005269 | 0.000935 | 1.78E-08 |
| rs2068888 | G | A | 0.448688 | -0.00431 | 0.000754 | 1.06E-08 |
| rs964184 | G | C | 0.132181 | -0.02102 | 0.001107 | 2.28E-80 |
| rs141469619 | A | G | 0.010149 | 0.023613 | 0.003954 | 2.34E-09 |
| rs11601507 | C | A | 0.068393 | 0.008508 | 0.001467 | 6.61E-09 |
| rs1169288 | A | C | 0.313007 | 0.006996 | 0.000814 | 8.09E-18 |
| rs10846740 | T | C | 0.140317 | -0.00607 | 0.001099 | 3.39E-08 |
| rs1042725 | C | T | 0.491401 | 0.004255 | 0.000752 | 1.50E-08 |
| rs9376091 | C | T | 0.260624 | -0.00503 | 0.000858 | 4.57E-09 |
| rs456598 | G | A | 0.142135 | 0.006916 | 0.001077 | 1.37E-10 |
| rs117733303 | A | G | 0.018612 | 0.030942 | 0.002776 | 7.39E-29 |
| rs74617384 | A | T | 0.080639 | 0.019391 | 0.001378 | 5.93E-45 |
| rs2237107 | C | G | 0.203915 | -0.00659 | 0.000932 | 1.53E-12 |
| rs28752924 | T | C | 0.440563 | 0.004342 | 0.000781 | 2.77E-08 |
| rs622871 | A | G | 0.315941 | 0.006372 | 0.000809 | 3.29E-15 |
| rs6905288 | G | A | 0.432658 | 0.005202 | 0.000758 | 6.66E-12 |
| rs3918226 | C | T | 0.081323 | 0.007958 | 0.001393 | 1.10E-08 |
| rs55649657 | C | G | 0.220322 | 0.006755 | 0.000906 | 8.92E-14 |
| rs10260606 | G | C | 0.182264 | 0.007541 | 0.00097 | 7.44E-15 |
| rs799157 | T | C | 0.043509 | -0.01092 | 0.001839 | 2.95E-09 |
| rs28601761 | C | G | 0.419471 | -0.01391 | 0.000769 | 4.73E-73 |
| rs57180587 | A | T | 0.145803 | 0.007572 | 0.001061 | 9.63E-13 |
| rs10096633 | C | T | 0.122814 | -0.0098 | 0.001143 | 9.95E-18 |
| rs10504255 | G | A | 0.33714 | -0.00541 | 0.000794 | 9.44E-12 |
| rs2126263 | G | A | 0.100814 | 0.007693 | 0.001253 | 8.18E-10 |
| rs1883025 | C | T | 0.254383 | -0.00686 | 0.000862 | 1.79E-15 |
| rs635634 | C | T | 0.184154 | 0.009612 | 0.000973 | 5.06E-23 |
| rs1601935 | G | T | 0.344328 | -0.00596 | 0.000795 | 6.12E-14 |
| rs588136 | C | T | 0.213012 | -0.00518 | 0.00092 | 1.77E-08 |
| rs11864054 | G | A | 0.386871 | 0.004635 | 0.000775 | 2.23E-09 |
| rs34042070 | C | G | 0.186115 | 0.009848 | 0.000968 | 2.63E-24 |
| rs3874910 | C | T | 0.438576 | -0.00533 | 0.00076 | 2.39E-12 |
| rs77542162 | A | G | 0.02301 | 0.02364 | 0.002505 | 3.82E-21 |
| rs7210574 | T | C | 0.327834 | -0.00514 | 0.000801 | 1.42E-10 |
| rs8090363 | A | G | 0.396865 | 0.004216 | 0.000769 | 4.27E-08 |
| rs6511720 | G | T | 0.118872 | -0.02605 | 0.001159 | ###### |
| rs2738447 | A | C | 0.407338 | 0.007685 | 0.000764 | 8.94E-24 |
| rs58542926 | C | T | 0.075432 | -0.0148 | 0.001422 | 2.36E-25 |
| rs28807203 | A | C | 0.048866 | -0.01135 | 0.001747 | 8.31E-11 |
| rs10410835 | T | C | 0.476126 | 0.007449 | 0.00076 | 1.17E-22 |
| rs1081105 | A | C | 0.027887 | 0.02642 | 0.002281 | 5.26E-31 |
| rs1065853 | G | T | 0.080299 | -0.03727 | 0.00138 | ###### |
| rs2618567 | G | T | 0.33983 | -0.00519 | 0.000793 | 5.67E-11 |
| rs1883711 | G | C | 0.031031 | 0.023304 | 0.002205 | 4.23E-26 |
| rs6129734 | G | A | 0.160404 | 0.005736 | 0.001023 | 2.08E-08 |
| rs8126001 | C | T | 0.489728 | -0.00526 | 0.000753 | 2.98E-12 |

**Table S11. Genome-wide significant and independent SNPs that were used as instruments for HDL**

| SNP | effect | other | beta. | se. | pval. | eaf. |
| --- | --- | --- | --- | --- | --- | --- |
| rs2250802 | G | A | 0.034 | 0.0038 | 2.02E-17 | 0.3193 |
| rs12412743 | C | T | 0.0291 | 0.0045 | 1.31E-09 | 0.847 |
| rs970548 | C | A | 0.0258 | 0.0039 | 1.71E-10 | 0.277 |
| rs10761771 | C | T | 0.0198 | 0.0034 | 4.12E-09 | 0.467 |
| rs12740374 | T | G | 0.0343 | 0.0041 | 1.69E-15 | 0.2124 |
| rs10501321 | C | T | 0.0483 | 0.0036 | 3.54E-38 | 0.314 |
| rs12145743 | G | T | 0.0203 | 0.0036 | 1.80E-08 | 0.3311 |
| rs102275 | T | C | 0.0391 | 0.0035 | 6.40E-28 | 0.628 |
| rs12801636 | A | G | 0.0235 | 0.0042 | 3.15E-08 | 0.2243 |
| rs499974 | C | A | 0.0263 | 0.0044 | 1.12E-08 | 0.8245 |
| rs4650994 | G | A | 0.021 | 0.0034 | 6.70E-09 | 0.5172 |
| rs1689797 | C | A | 0.0358 | 0.0036 | 2.85E-21 | 0.6979 |
| rs2241210 | G | A | 0.0332 | 0.0035 | 2.49E-20 | 0.5528 |
| rs11065987 | A | G | 0.0222 | 0.0035 | 1.23E-09 | 0.5778 |
| rs2454722 | G | A | 0.0351 | 0.0044 | 3.31E-14 | 0.1451 |
| rs838876 | A | G | 0.0493 | 0.0039 | 7.33E-33 | 0.3259 |
| rs7306660 | G | A | 0.0345 | 0.0036 | 3.34E-19 | 0.6306 |
| rs4379922 | C | T | 0.0247 | 0.0036 | 9.56E-12 | 0.3496 |
| rs2642438 | G | A | 0.0303 | 0.0039 | 7.78E-14 | 0.7454 |
| rs11045163 | G | A | 0.0217 | 0.0035 | 3.20E-09 | 0.4063 |
| rs4846914 | A | G | 0.0479 | 0.0034 | 3.51E-41 | 0.5844 |
| rs3741414 | T | C | 0.0296 | 0.004 | 6.10E-14 | 0.1913 |
| rs12748152 | C | T | 0.0506 | 0.0062 | 9.74E-16 | 0.92876 |
| rs4660293 | A | G | 0.0353 | 0.004 | 2.86E-18 | 0.7639 |
| rs4983559 | G | A | 0.0197 | 0.0036 | 9.57E-09 | 0.3773 |
| rs492571 | T | C | 0.0663 | 0.009 | 1.27E-12 | 0.95778 |
| rs10468017 | T | C | 0.1179 | 0.0038 | ####### | 0.2757 |
| rs633695 | G | A | 0.0885 | 0.0054 | 7.82E-58 | 0.285 |
| rs424346 | T | C | 0.0679 | 0.0113 | 4.84E-08 | 0.04881 |
| rs2241770 | T | C | 0.0989 | 0.0057 | 6.78E-60 | 0.8971 |
| rs9989419 | G | A | 0.1473 | 0.0036 | ####### | 0.595 |
| rs16965220 | A | C | 0.0219 | 0.0037 | 7.91E-09 | 0.2982 |
| rs16942887 | A | G | 0.0831 | 0.0051 | 8.28E-54 | 0.1332 |
| rs2925979 | C | T | 0.0351 | 0.0037 | 1.32E-19 | 0.7045 |
| rs1877031 | A | G | 0.0336 | 0.0036 | 1.20E-19 | 0.6755 |
| rs4148005 | T | G | 0.0283 | 0.0036 | 5.74E-14 | 0.7005 |
| rs4969178 | G | A | 0.0263 | 0.0035 | 1.53E-12 | 0.6266 |
| rs4939883 | C | T | 0.0799 | 0.0045 | 1.80E-66 | 0.8193 |
| rs6567160 | T | C | 0.0257 | 0.0041 | 2.92E-09 | 0.7691 |
| rs737337 | T | C | 0.0565 | 0.0061 | 4.56E-17 | 0.9314 |
| rs12133576 | A | G | 0.0243 | 0.0035 | 6.15E-11 | 0.3549 |
| rs731839 | A | G | 0.022 | 0.0037 | 3.44E-09 | 0.6583 |
| rs2075650 | A | G | 0.0554 | 0.0051 | 9.72E-26 | 0.8734 |
| rs2288912 | G | C | 0.0297 | 0.0036 | 7.15E-15 | 0.4987 |
| rs103294 | T | C | 0.0523 | 0.0044 | 4.00E-30 | 0.186 |
| rs2278236 | A | G | 0.0331 | 0.0035 | 3.19E-18 | 0.5435 |
| rs6031587 | C | T | 0.0488 | 0.0074 | 1.92E-09 | 0.9314 |
| rs4465830 | A | G | 0.0597 | 0.0044 | 5.18E-40 | 0.7982 |
| rs7607980 | C | T | 0.0447 | 0.0052 | 1.81E-15 | 0.1491 |
| rs676210 | A | G | 0.066 | 0.004 | 2.35E-54 | 0.2309 |
| rs1047891 | C | A | 0.0269 | 0.0039 | 8.73E-10 | 0.6979 |
| rs181360 | T | G | 0.0376 | 0.0042 | 9.24E-18 | 0.8008 |
| rs1515110 | G | T | 0.0323 | 0.0035 | 8.04E-18 | 0.3813 |
| rs2606736 | C | T | 0.0246 | 0.0043 | 4.80E-08 | 0.3945 |
| rs6805251 | T | C | 0.02 | 0.0035 | 1.33E-08 | 0.3813 |
| rs13076253 | A | C | 0.0283 | 0.0048 | 4.96E-09 | 0.8522 |
| rs687339 | C | T | 0.0316 | 0.0042 | 7.11E-13 | 0.2335 |
| rs2013208 | T | C | 0.0254 | 0.0036 | 8.92E-12 | 0.5053 |
| rs13099479 | A | G | 0.036 | 0.0062 | 1.82E-08 | 0.08971 |
| rs2602836 | A | G | 0.0192 | 0.0034 | 4.96E-08 | 0.4274 |
| rs13107325 | C | T | 0.0708 | 0.0078 | 1.07E-15 | 0.92216 |
| rs10019888 | A | G | 0.027 | 0.0046 | 4.90E-08 | 0.8364 |
| rs3822072 | G | A | 0.0251 | 0.0034 | 4.06E-12 | 0.5119 |
| rs6450176 | G | A | 0.0254 | 0.0039 | 6.88E-10 | 0.7216 |
| rs1936800 | C | T | 0.02 | 0.0034 | 3.06E-10 | 0.5277 |
| rs3861397 | A | G | 0.024 | 0.0036 | 8.40E-11 | 0.6583 |
| rs9457931 | A | G | 0.0552 | 0.0073 | 7.30E-13 | 0.9314 |
| rs1980493 | T | C | 0.0318 | 0.0048 | 3.76E-10 | 0.8773 |
| rs205262 | A | G | 0.0283 | 0.0039 | 3.88E-13 | 0.7335 |
| rs998584 | C | A | 0.026 | 0.0038 | 2.27E-11 | 0.4855 |
| rs11765979 | C | A | 0.0412 | 0.0048 | 3.11E-17 | 0.4578 |
| rs17173637 | T | C | 0.0363 | 0.0057 | 1.90E-08 | 0.90237 |
| rs4142995 | G | T | 0.0263 | 0.0037 | 9.37E-12 | 0.6161 |
| rs4917014 | G | T | 0.0222 | 0.0036 | 1.03E-08 | 0.3404 |
| rs702485 | G | A | 0.0243 | 0.0034 | 6.45E-12 | 0.4499 |
| rs17145738 | T | C | 0.0408 | 0.0053 | 4.95E-13 | 0.1174 |
| rs2293889 | G | T | 0.0312 | 0.0035 | 4.27E-17 | 0.5871 |
| rs10808546 | T | C | 0.0409 | 0.0034 | 4.11E-30 | 0.4459 |
| rs10087900 | G | A | 0.0231 | 0.0036 | 2.17E-09 | 0.5607 |
| rs1866956 | T | C | 0.0217 | 0.0037 | 7.96E-10 | 0.6755 |
| rs13702 | C | T | 0.1058 | 0.0038 | ####### | 0.3127 |
| rs4240624 | A | G | 0.0818 | 0.0058 | 1.32E-45 | 0.9248 |
| rs2066714 | C | T | 0.0453 | 0.0071 | 7.26E-10 | 0.1201 |
| rs11789603 | T | C | 0.06 | 0.006 | 3.70E-21 | 0.08971 |
| rs1883025 | C | T | 0.0698 | 0.0041 | 1.50E-65 | 0.7573 |
| rs686030 | A | C | 0.055 | 0.0049 | 4.29E-27 | 0.8588 |
| rs7112577 | G | C | 0.0826 | 0.0129 | 2.34E-10 | NA |
| rs964184 | C | G | 0.1065 | 0.0071 | 6.09E-48 | NA |

**Table S12. Genome-wide significant and independent SNPs that were used as instruments for LDL**

| SNP | effect | other | beta | se. | pval. | eaf. |
| --- | --- | --- | --- | --- | --- | --- |
| rs2419604 | A | G | 0.0302 | 0.004 | 7.49E-14 | 0.3179 |
| rs413380 | C | T | 0.0861 | 0.0098 | 7.62E-17 | 0.9657 |
| rs4970834 | C | T | 0.1503 | 0.0047 | ####### | 0.8127 |
| rs10893499 | A | G | 0.0521 | 0.0053 | 3.86E-21 | 0.1438 |
| rs10832962 | T | C | 0.032 | 0.004 | 6.62E-14 | 0.719 |
| rs267733 | A | G | 0.0331 | 0.0053 | 5.29E-09 | 0.8628 |
| rs174583 | C | T | 0.0522 | 0.0038 | 7.00E-41 | 0.6253 |
| rs3184504 | C | T | 0.0268 | 0.0038 | 4.20E-12 | 0.5343 |
| rs1169288 | C | A | 0.0375 | 0.004 | 6.45E-21 | 0.3338 |
| rs2642438 | G | A | 0.0352 | 0.0042 | 7.32E-16 | 0.7454 |
| rs2587534 | A | G | 0.0391 | 0.0037 | 8.06E-25 | 0.5277 |
| rs10903129 | G | A | 0.0328 | 0.0037 | 3.03E-17 | 0.5369 |
| rs12748152 | T | C | 0.0499 | 0.0066 | 3.21E-12 | 0.07124 |
| rs4942486 | T | C | 0.0243 | 0.0037 | 2.26E-11 | 0.4617 |
| rs8017377 | A | G | 0.0303 | 0.0038 | 2.52E-15 | 0.4591 |
| rs11591147 | G | T | 0.497 | 0.018 | ####### | 0.98285 |
| rs2902875 | T | C | 0.0756 | 0.0126 | 2.19E-09 | 0.0409 |
| rs7551981 | T | G | 0.0472 | 0.0038 | 1.36E-33 | 0.595 |
| rs7534572 | G | C | 0.0407 | 0.0058 | 1.29E-11 | 0.69 |
| rs247616 | C | T | 0.0547 | 0.0041 | 2.57E-37 | 0.7071 |
| rs2000999 | A | G | 0.065 | 0.0046 | 4.22E-41 | 0.1847 |
| rs6504872 | T | C | 0.0274 | 0.0037 | 3.48E-13 | 0.4723 |
| rs1801689 | C | A | 0.1028 | 0.0139 | 9.81E-12 | 0.03694 |
| rs2886232 | T | C | 0.0451 | 0.0064 | 3.88E-11 | 0.1201 |
| rs314253 | T | C | 0.0242 | 0.0038 | 3.44E-10 | 0.6649 |
| rs6511720 | G | T | 0.2209 | 0.0061 | ####### | 0.90237 |
| rs2228603 | C | T | 0.104 | 0.0072 | 4.43E-44 | 0.92876 |
| rs4970712 | C | A | 0.0339 | 0.0044 | 2.46E-13 | 0.8061 |
| rs2965157 | T | C | 0.1886 | 0.0112 | 7.29E-62 | 0.97889 |
| rs7254892 | G | A | 0.4853 | 0.0119 | ####### | 0.96834 |
| rs75687619 | T | G | 0.1735 | 0.0161 | 8.05E-24 | 0.02375 |
| rs12721109 | G | A | 0.4462 | 0.0183 | ####### | 0.98285 |
| rs676388 | C | T | 0.0265 | 0.0039 | 1.31E-11 | 0.4631 |
| rs364585 | G | A | 0.0249 | 0.0038 | 4.28E-10 | 0.6332 |
| rs2328223 | C | A | 0.0299 | 0.005 | 5.63E-09 | 0.2493 |
| rs6016373 | A | G | 0.0349 | 0.0037 | 7.95E-19 | 0.6266 |
| rs6065311 | C | T | 0.0417 | 0.0036 | 1.66E-30 | 0.4604 |
| rs1800961 | C | T | 0.0685 | 0.0106 | 6.03E-10 | 0.9657 |
| rs10490626 | G | A | 0.0508 | 0.0069 | 1.70E-12 | 0.92084 |
| rs2030746 | T | C | 0.0214 | 0.0038 | 8.61E-09 | 0.3984 |
| rs16831243 | T | C | 0.0378 | 0.0055 | 9.06E-12 | 0.1807 |
| rs10195252 | T | C | 0.0238 | 0.0039 | 3.81E-08 | 0.5818 |
| rs1367117 | A | G | 0.1186 | 0.004 | ####### | 0.2876 |
| rs72902576 | T | G | 0.0933 | 0.0133 | 9.58E-12 | 0.96306 |
| rs1250229 | C | T | 0.0243 | 0.0042 | 3.13E-08 | 0.7889 |
| rs5763662 | T | C | 0.0767 | 0.0121 | 1.19E-08 | 0.02507 |
| rs11563251 | T | C | 0.0345 | 0.0062 | 4.50E-08 | 0.1253 |
| rs4253776 | G | A | 0.0311 | 0.0059 | 3.35E-08 | 0.124 |
| rs6544713 | T | C | 0.0806 | 0.0041 | 4.84E-83 | 0.2942 |
| rs6709904 | A | G | 0.055 | 0.0085 | 4.58E-10 | 0.8865 |
| rs2710642 | A | G | 0.0239 | 0.0038 | 6.09E-09 | 0.6187 |
| rs9875338 | G | A | 0.027 | 0.0037 | 2.21E-11 | 0.6121 |
| rs17404153 | G | T | 0.0336 | 0.0054 | 1.83E-09 | 0.8562 |
| rs7640978 | C | T | 0.0392 | 0.0069 | 9.84E-09 | 0.8945 |
| rs6818397 | T | G | 0.0224 | 0.004 | 1.68E-08 | 0.4129 |
| rs4530754 | A | G | 0.0275 | 0.0036 | 3.58E-12 | 0.5818 |
| rs6882076 | C | T | 0.0456 | 0.0038 | 3.31E-31 | 0.6662 |
| rs12916 | C | T | 0.0733 | 0.0038 | 7.79E-78 | 0.4314 |
| rs6909746 | C | T | 0.0263 | 0.0037 | 7.86E-11 | 0.6082 |
| rs112201728 | T | C | 0.0675 | 0.0104 | 8.51E-10 | 0.05805 |
| rs1564348 | C | T | 0.0481 | 0.005 | 2.76E-21 | 0.1451 |
| rs16891156 | C | A | 0.0965 | 0.0171 | 8.23E-09 | 0.01847 |
| rs2315065 | A | C | 0.1102 | 0.0158 | 5.23E-12 | 0.08707 |
| rs3757354 | C | T | 0.0382 | 0.0044 | 2.09E-17 | 0.7902 |
| rs13206249 | G | A | 0.0378 | 0.0062 | 4.53E-08 | 0.7836 |
| rs1408272 | T | G | 0.052 | 0.0083 | 3.68E-09 | 0.94723 |
| rs10947332 | A | G | 0.0504 | 0.0056 | 6.97E-18 | 0.1319 |
| rs2390536 | A | G | 0.0223 | 0.0038 | 2.04E-08 | 0.3681 |
| rs4722551 | C | T | 0.0391 | 0.0049 | 3.95E-14 | 0.1702 |
| rs2073547 | G | A | 0.0485 | 0.0049 | 1.92E-21 | 0.1939 |
| rs2737252 | G | A | 0.0314 | 0.0041 | 7.04E-14 | 0.7441 |
| rs2954029 | A | T | 0.0564 | 0.0036 | 2.10E-50 | 0.5317 |
| rs7832643 | T | G | 0.0339 | 0.0038 | 2.67E-17 | 0.405 |
| rs13277801 | C | T | 0.0338 | 0.0038 | 3.99E-17 | 0.347 |
| rs9987289 | G | A | 0.0714 | 0.0066 | 8.53E-24 | 0.9248 |
| rs1883025 | C | T | 0.0296 | 0.0044 | 6.14E-11 | 0.7573 |
| rs579459 | C | T | 0.0665 | 0.0045 | 2.42E-44 | 0.215 |
| rs3780181 | A | G | 0.0445 | 0.0074 | 1.76E-09 | 0.94723 |
| rs964184 | G | C | 0.0855 | 0.0078 | 2.01E-26 | NA |

**Table S13. Genome-wide significant and independent SNPs that were used as instruments for TG**

| SNP | effect | other_ | beta. | se. | pval.exposure | eaf.exposure |
| --- | --- | --- | --- | --- | --- | --- |
| rs2250802 | A | G | 0.023 | 0.0037 | 1.21E-10 | 0.6807 |
| rs1832007 | A | G | 0.0327 | 0.0047 | 1.72E-12 | 0.8681 |
| rs10761762 | T | C | 0.027 | 0.0033 | 1.06E-17 | 0.533 |
| rs2068888 | G | A | 0.0241 | 0.0034 | 1.68E-11 | 0.5092 |
| rs948690 | T | C | 0.0306 | 0.0052 | 6.57E-09 | 0.6953 |
| rs7350481 | T | C | 0.2254 | 0.0066 | ####### | 0.09763 |
| rs12280753 | T | C | 0.1931 | 0.0064 | ####### | 0.06728 |
| rs10501321 | T | C | 0.0216 | 0.0035 | 1.41E-08 | 0.686 |
| rs174535 | C | T | 0.047 | 0.0034 | 1.73E-41 | 0.3628 |
| rs11057408 | G | T | 0.0258 | 0.0035 | 2.05E-12 | 0.6372 |
| rs1321257 | G | A | 0.0402 | 0.0034 | 5.99E-31 | 0.4063 |
| rs11613352 | C | T | 0.028 | 0.0039 | 9.40E-14 | 0.8087 |
| rs12748152 | T | C | 0.0372 | 0.0059 | 1.10E-09 | 0.07124 |
| rs17513135 | T | C | 0.022 | 0.0039 | 1.63E-08 | 0.2322 |
| rs16948098 | A | G | 0.08 | 0.0089 | 4.84E-17 | 0.0409 |
| rs2043085 | T | C | 0.0327 | 0.0034 | 7.81E-20 | 0.3681 |
| rs588136 | C | T | 0.0495 | 0.0041 | 3.37E-30 | 0.2058 |
| rs3198697 | C | T | 0.0198 | 0.0034 | 2.21E-08 | 0.6174 |
| rs4587594 | G | A | 0.0694 | 0.0035 | 3.50E-82 | 0.69 |
| rs749671 | G | A | 0.0211 | 0.0034 | 6.11E-10 | 0.6055 |
| rs1800775 | C | A | 0.0396 | 0.0035 | 1.33E-26 | 0.5198 |
| rs8077889 | C | A | 0.0252 | 0.0042 | 9.88E-09 | 0.2441 |
| rs10401969 | T | C | 0.121 | 0.0065 | 9.70E-70 | 0.92876 |
| rs731839 | G | A | 0.0224 | 0.0036 | 2.65E-09 | 0.3417 |
| rs439401 | C | T | 0.0659 | 0.0038 | 1.42E-66 | 0.6201 |
| rs3760627 | C | T | 0.0189 | 0.0034 | 5.29E-09 | 0.4683 |
| rs7248104 | G | A | 0.0222 | 0.0034 | 5.05E-10 | 0.5831 |
| rs6029143 | C | T | 0.0388 | 0.0071 | 4.93E-08 | 0.94195 |
| rs4810479 | C | T | 0.0474 | 0.0038 | 2.07E-34 | 0.2876 |
| rs13389219 | C | T | 0.0271 | 0.0034 | 2.60E-15 | 0.591 |
| rs676210 | G | A | 0.0733 | 0.0039 | 3.28E-71 | 0.7691 |
| rs2972146 | T | G | 0.0281 | 0.0034 | 2.97E-15 | 0.6227 |
| rs3761445 | A | G | 0.0232 | 0.0034 | 8.06E-12 | 0.6148 |
| rs1260326 | T | C | 0.1148 | 0.0034 | ####### | 0.4129 |
| rs10440120 | C | A | 0.0306 | 0.0044 | 5.34E-11 | 0.8325 |
| rs645040 | T | G | 0.0293 | 0.004 | 1.83E-12 | 0.7691 |
| rs6831256 | G | A | 0.0258 | 0.0035 | 1.60E-12 | 0.409 |
| rs442177 | T | G | 0.0309 | 0.0033 | 1.32E-18 | 0.5528 |
| rs6882076 | C | T | 0.0286 | 0.0035 | 1.51E-15 | 0.6662 |
| rs9686661 | T | C | 0.0379 | 0.0044 | 2.54E-16 | 0.1768 |
| rs719726 | T | C | 0.0199 | 0.0035 | 2.49E-08 | 0.529 |
| rs634869 | T | C | 0.0272 | 0.0033 | 1.78E-14 | 0.438 |
| rs2665357 | C | A | 0.0212 | 0.0033 | 8.33E-10 | 0.5092 |
| rs2239520 | G | A | 0.0236 | 0.0037 | 4.14E-10 | 0.6266 |
| rs2247056 | C | T | 0.0378 | 0.0039 | 3.86E-21 | 0.7823 |
| rs998584 | A | C | 0.0293 | 0.0037 | 3.42E-15 | 0.5145 |
| rs38855 | A | G | 0.0187 | 0.0033 | 2.11E-08 | 0.5264 |
| rs287621 | T | C | 0.0222 | 0.0037 | 7.67E-09 | 0.2704 |
| rs4719841 | G | A | 0.0232 | 0.0034 | 8.86E-11 | 0.3826 |
| rs11974409 | A | G | 0.0899 | 0.0042 | ####### | 0.8061 |
| rs6995541 | G | A | 0.0265 | 0.0037 | 1.34E-12 | 0.3219 |
| rs2954022 | C | A | 0.078 | 0.0033 | ####### | 0.5303 |
| rs12676857 | C | T | 0.0332 | 0.0046 | 7.29E-12 | 0.1544 |
| rs12678919 | A | G | 0.1702 | 0.0056 | ####### | 0.8786 |
| rs4738684 | A | G | 0.0205 | 0.0035 | 8.82E-09 | 0.3522 |

**Table S14. Genome-wide significant and independent SNPs that were used as instruments for hypertension**

| SNP | effect | other | eaf. | beta.exposure | se.exposure | pval.exposure |
| --- | --- | --- | --- | --- | --- | --- |
| rs880315 | T | C | 0.340624 | 0.012667 | 0.001062 | 8.68E-33 |
| rs3790604 | C | A | 0.07415 | 0.019997 | 0.001916 | 1.67E-25 |
| rs57748895 | A | T | 0.017886 | 0.030764 | 0.003779 | 3.94E-16 |
| rs3753580 | T | C | 0.295059 | -0.0094 | 0.001154 | 3.68E-16 |
| rs35479618 | G | A | 0.017241 | 0.025133 | 0.003843 | 6.18E-11 |
| rs77037886 | A | G | 0.187978 | 0.007312 | 0.001284 | 1.23E-08 |
| rs1745417 | C | T | 0.488446 | 0.00687 | 0.001005 | 8.31E-12 |
| rs2478539 | G | T | 0.400748 | 0.007852 | 0.001021 | 1.50E-14 |
| rs448385 | G | A | 0.440269 | 0.007131 | 0.00101 | 1.64E-12 |
| rs7515635 | T | C | 0.456634 | -0.00574 | 0.001008 | 1.26E-08 |
| rs12116494 | G | T | 0.362841 | 0.005883 | 0.001044 | 1.73E-08 |
| rs61772626 | A | G | 0.123154 | 0.009012 | 0.001525 | 3.40E-09 |
| rs301802 | T | A | 0.41323 | 0.006207 | 0.001019 | 1.12E-09 |
| rs10493818 | C | T | 0.385942 | 0.006848 | 0.00103 | 3.00E-11 |
| rs11692391 | T | C | 0.215788 | -0.00694 | 0.001217 | 1.19E-08 |
| rs72873624 | G | A | 0.094672 | -0.01016 | 0.00171 | 2.87E-09 |
| rs268263 | T | A | 0.246523 | 0.00804 | 0.001172 | 6.74E-12 |
| rs10930990 | C | G | 0.323027 | -0.00652 | 0.00108 | 1.60E-09 |
| rs188315257 | T | G | 0.092096 | -0.01246 | 0.001742 | 8.72E-13 |
| rs1275988 | C | T | 0.384166 | -0.01369 | 0.001031 | 3.01E-40 |
| rs3902740 | T | C | 0.382638 | -0.00665 | 0.001031 | 1.08E-10 |
| rs115262049 | A | T | 0.089237 | -0.01104 | 0.001769 | 4.27E-10 |
| rs17335134 | A | G | 0.275253 | -0.00758 | 0.001127 | 1.76E-11 |
| rs2443708 | T | C | 0.314942 | 0.007175 | 0.001081 | 3.21E-11 |
| rs35432681 | T | C | 0.379951 | 0.006419 | 0.001033 | 5.19E-10 |
| rs1154988 | T | A | 0.226356 | 0.007629 | 0.001196 | 1.81E-10 |
| rs9872754 | C | T | 0.160665 | 0.007524 | 0.001364 | 3.48E-08 |
| rs9844972 | G | C | 0.071355 | 0.011995 | 0.001979 | 1.36E-09 |
| rs55770580 | A | G | 0.401674 | 0.006543 | 0.001023 | 1.60E-10 |
| rs56743174 | G | A | 0.072038 | -0.01095 | 0.001938 | 1.58E-08 |
| rs6773655 | A | G | 0.390175 | 0.007081 | 0.001041 | 1.01E-11 |
| rs2643826 | C | T | 0.452847 | 0.010472 | 0.001009 | 2.99E-25 |
| rs35942721 | C | T | 0.291136 | -0.00767 | 0.001104 | 3.71E-12 |
| rs3821843 | G | A | 0.321898 | 0.00954 | 0.001089 | 1.98E-18 |
| rs7340705 | T | C | 0.32275 | 0.007015 | 0.001078 | 7.70E-11 |
| rs9836592 | C | T | 0.336389 | 0.006409 | 0.001062 | 1.56E-09 |
| rs13127398 | T | A | 0.072291 | -0.01395 | 0.001946 | 7.56E-13 |
| rs56388530 | C | T | 0.239658 | 0.009388 | 0.001173 | 1.22E-15 |
| rs2881854 | C | A | 0.150132 | -0.01001 | 0.001401 | 8.84E-13 |
| rs9286351 | A | G | 0.416988 | 0.005802 | 0.001018 | 1.20E-08 |
| rs893929 | G | A | 0.454213 | -0.00644 | 0.001009 | 1.78E-10 |
| rs2625269 | G | A | 0.487407 | 0.006349 | 0.001005 | 2.71E-10 |
| rs11724647 | T | A | 0.184195 | -0.01048 | 0.001292 | 5.09E-16 |
| rs113591949 | A | G | 0.125611 | 0.009156 | 0.001523 | 1.85E-09 |
| rs28667801 | A | T | 0.406248 | 0.007052 | 0.001025 | 6.10E-12 |
| rs6812640 | C | G | 0.471068 | -0.0065 | 0.001015 | 1.46E-10 |
| rs13125101 | G | A | 0.293256 | 0.020619 | 0.001102 | 4.72E-78 |
| rs140069875 | G | T | 0.453166 | -0.00785 | 0.001046 | 6.00E-14 |
| rs74661587 | A | G | 0.132144 | 0.009813 | 0.001478 | 3.14E-11 |
| rs17677603 | A | G | 0.394263 | 0.00765 | 0.00103 | 1.13E-13 |
| rs7449190 | T | C | 0.141401 | -0.0081 | 0.001447 | 2.20E-08 |
| rs72801474 | G | A | 0.092636 | -0.00988 | 0.001728 | 1.10E-08 |
| rs1650581 | C | G | 0.261812 | 0.010661 | 0.001144 | 1.16E-20 |
| rs7701003 | A | G | 0.368868 | -0.0108 | 0.001038 | 2.35E-25 |
| rs36071027 | C | T | 0.361164 | -0.00765 | 0.001045 | 2.45E-13 |
| rs11955537 | A | G | 0.136957 | -0.00826 | 0.001455 | 1.37E-08 |
| rs12656497 | T | C | 0.403605 | 0.012169 | 0.001021 | 9.82E-33 |
| rs13179413 | C | T | 0.285061 | 0.007472 | 0.001134 | 4.44E-11 |
| rs27687 | T | C | 0.273006 | 0.0069 | 0.001125 | 8.63E-10 |
| rs17419291 | T | C | 0.0871 | -0.01087 | 0.001774 | 9.14E-10 |
| rs11191580 | T | C | 0.077092 | -0.01587 | 0.001873 | 2.37E-17 |
| rs11196549 | G | A | 0.042776 | 0.014041 | 0.002515 | 2.37E-08 |
| rs740746 | G | A | 0.264522 | 0.011104 | 0.001139 | 1.86E-22 |
| rs10749409 | C | G | 0.315668 | -0.00588 | 0.001078 | 4.97E-08 |
| rs75523587 | T | A | 0.183808 | 0.007712 | 0.001292 | 2.37E-09 |
| rs12413195 | T | C | 0.236055 | 0.00771 | 0.001181 | 6.67E-11 |
| rs12258967 | C | G | 0.298302 | -0.01061 | 0.001096 | 3.69E-22 |
| rs2026427 | T | C | 0.285028 | -0.00645 | 0.001128 | 1.04E-08 |
| rs72831343 | T | G | 0.145259 | -0.01905 | 0.001419 | 4.56E-41 |
| rs10995311 | C | G | 0.446542 | -0.00701 | 0.001012 | 4.26E-12 |
| rs61862567 | G | A | 0.492419 | -0.00587 | 0.001003 | 4.90E-09 |
| rs2735348 | A | T | 0.255235 | 0.006708 | 0.001159 | 7.14E-09 |
| rs3891783 | C | G | 0.432367 | -0.01011 | 0.001011 | 1.49E-23 |
| rs604723 | T | C | 0.275208 | 0.01485 | 0.001128 | 1.54E-39 |
| rs11608149 | G | A | 0.261118 | 0.00774 | 0.001148 | 1.57E-11 |
| rs1384013 | G | C | 0.245705 | 0.00646 | 0.001167 | 3.10E-08 |
| rs7110527 | T | A | 0.211341 | -0.01056 | 0.001234 | 1.15E-17 |
| rs4980379 | C | T | 0.36438 | 0.011218 | 0.001044 | 5.96E-27 |
| rs290102 | C | T | 0.434555 | -0.00571 | 0.001018 | 2.09E-08 |
| rs3809060 | G | T | 0.374703 | 0.006469 | 0.001037 | 4.49E-10 |
| rs7118770 | C | T | 0.497569 | 0.006175 | 0.001002 | 7.05E-10 |
| rs7117386 | G | A | 0.210293 | 0.011973 | 0.001228 | 1.80E-22 |
| rs34530191 | A | G | 0.211735 | -0.00682 | 0.001226 | 2.65E-08 |
| rs2943810 | C | G | 0.260907 | 0.006412 | 0.001157 | 3.01E-08 |
| rs557675 | T | G | 0.470152 | -0.0075 | 0.001004 | 8.36E-14 |
| rs1155448 | A | G | 0.404659 | 0.005617 | 0.00102 | 3.61E-08 |
| rs173396 | G | A | 0.428807 | 0.00685 | 0.001011 | 1.26E-11 |
| rs7310615 | C | G | 0.481796 | -0.01141 | 0.001009 | 1.16E-29 |
| rs35429 | A | G | 0.382922 | -0.01095 | 0.001031 | 2.58E-26 |
| rs4981000 | T | C | 0.491549 | -0.00644 | 0.001004 | 1.37E-10 |
| rs7972957 | C | G | 0.271277 | 0.007919 | 0.001126 | 2.06E-12 |
| rs4883481 | T | C | 0.3711 | -0.00752 | 0.001036 | 3.89E-13 |
| rs12426667 | A | C | 0.30024 | -0.00815 | 0.001098 | 1.17E-13 |
| rs11105334 | G | A | 0.380192 | -0.01138 | 0.001068 | 1.68E-26 |
| rs682709 | C | G | 0.333953 | 0.007536 | 0.001074 | 2.32E-12 |
| rs9375459 | C | T | 0.435918 | 0.011888 | 0.001009 | 5.05E-32 |
| rs2105092 | G | A | 0.290668 | -0.00629 | 0.001104 | 1.23E-08 |
| rs57139556 | A | G | 0.071557 | -0.01869 | 0.001943 | 6.88E-22 |
| rs6557149 | C | T | 0.399438 | -0.00597 | 0.001022 | 5.01E-09 |
| rs2251828 | G | A | 0.246699 | 0.006798 | 0.001162 | 4.91E-09 |
| rs375456190 | A | G | 0.063104 | -0.01251 | 0.002081 | 1.86E-09 |
| rs16885627 | C | G | 0.40179 | 0.006471 | 0.001028 | 3.04E-10 |
| rs2744133 | A | G | 0.278469 | -0.00684 | 0.001118 | 9.79E-10 |
| rs198851 | T | G | 0.15108 | -0.01371 | 0.001398 | 1.12E-22 |
| rs3020644 | A | G | 0.358442 | 0.007913 | 0.001044 | 3.51E-14 |
| rs7763350 | A | C | 0.319659 | 0.008344 | 0.001073 | 7.47E-15 |
| rs11751804 | A | G | 0.241035 | 0.006823 | 0.00118 | 7.41E-09 |
| rs1507153 | C | A | 0.377861 | 0.00689 | 0.001035 | 2.78E-11 |
| rs6923212 | T | C | 0.189388 | 0.007663 | 0.001279 | 2.11E-09 |
| rs62481856 | G | A | 0.197142 | 0.00976 | 0.001258 | 8.56E-15 |
| rs10250103 | C | T | 0.478815 | 0.005767 | 0.001008 | 1.06E-08 |
| rs11556924 | C | T | 0.390025 | -0.00769 | 0.001027 | 7.25E-14 |
| rs35769913 | C | T | 0.411493 | -0.00643 | 0.001019 | 2.78E-10 |
| rs7807797 | A | G | 0.191942 | -0.00824 | 0.001272 | 9.29E-11 |
| rs3918226 | C | T | 0.081323 | 0.024418 | 0.001858 | 1.91E-39 |
| rs6464165 | T | C | 0.282734 | 0.00716 | 0.001113 | 1.26E-10 |
| rs11770148 | A | G | 0.171458 | -0.00786 | 0.001337 | 4.13E-09 |
| rs10488611 | G | A | 0.191826 | -0.00776 | 0.001279 | 1.30E-09 |
| rs3735533 | T | C | 0.073662 | 0.016955 | 0.001923 | 1.18E-18 |
| rs6961048 | C | G | 0.100648 | 0.011541 | 0.001667 | 4.48E-12 |
| rs12702586 | G | A | 0.110972 | 0.011047 | 0.001596 | 4.50E-12 |
| rs62477724 | C | G | 0.218447 | 0.007297 | 0.001215 | 1.89E-09 |
| rs7844259 | G | A | 0.257105 | 0.006309 | 0.001155 | 4.76E-08 |
| rs4841436 | C | A | 0.402633 | -0.00804 | 0.001025 | 4.58E-15 |
| rs35783704 | G | A | 0.101314 | -0.01317 | 0.001676 | 3.97E-15 |
| rs12676542 | T | C | 0.339835 | -0.00627 | 0.001057 | 2.94E-09 |
| rs28394055 | C | T | 0.469838 | -0.00651 | 0.001032 | 2.79E-10 |
| rs951914 | G | C | 0.286221 | 0.008865 | 0.001115 | 1.82E-15 |
| rs4873492 | C | T | 0.170758 | 0.007509 | 0.001337 | 1.97E-08 |
| rs2977324 | T | G | 0.299022 | 0.006998 | 0.001098 | 1.88E-10 |
| rs6471502 | G | A | 0.470625 | -0.00566 | 0.001004 | 1.69E-08 |
| rs76038906 | G | T | 0.034679 | 0.018493 | 0.00275 | 1.77E-11 |
| rs532436 | G | A | 0.184639 | -0.00733 | 0.001293 | 1.45E-08 |
| rs6271 | C | T | 0.074057 | -0.01324 | 0.00191 | 4.16E-12 |
| rs76452347 | C | T | 0.204567 | -0.00939 | 0.001288 | 3.04E-13 |
| rs71440054 | T | A | 0.27603 | 0.006863 | 0.001142 | 1.86E-09 |
| rs3803266 | G | C | 0.231111 | -0.00811 | 0.001188 | 8.74E-12 |
| rs112684153 | T | C | 0.073252 | -0.01148 | 0.001981 | 6.85E-09 |
| rs72683923 | T | C | 0.020127 | -0.02304 | 0.003567 | 1.07E-10 |
| rs67007969 | C | A | 0.145569 | 0.008298 | 0.001436 | 7.52E-09 |
| rs2947489 | T | A | 0.427381 | -0.007 | 0.001017 | 5.74E-12 |
| rs4775373 | T | C | 0.360982 | -0.00572 | 0.001045 | 4.31E-08 |
| rs11072508 | C | T | 0.326157 | -0.01116 | 0.001073 | 2.66E-25 |
| rs2627323 | C | T | 0.467635 | 0.007937 | 0.001003 | 2.53E-15 |
| rs8027450 | C | T | 0.322736 | 0.01305 | 0.001075 | 6.76E-34 |
| rs77924615 | G | A | 0.197588 | -0.012 | 0.001269 | 3.19E-21 |
| rs9972727 | A | G | 0.353657 | 0.006549 | 0.001054 | 5.14E-10 |
| rs112767262 | C | T | 0.18767 | 0.007175 | 0.00129 | 2.65E-08 |
| rs732099 | C | A | 0.102868 | -0.00947 | 0.001655 | 1.05E-08 |
| rs10500326 | G | T | 0.234929 | -0.00724 | 0.001182 | 8.91E-10 |
| rs7498127 | G | A | 0.460593 | -0.00593 | 0.001005 | 3.72E-09 |
| rs12920176 | A | C | 0.410872 | 0.006528 | 0.001019 | 1.50E-10 |
| rs55872725 | C | T | 0.40208 | 0.008733 | 0.001021 | 1.23E-17 |
| rs6564889 | T | C | 0.407712 | 0.007912 | 0.001024 | 1.09E-14 |
| rs2460448 | G | A | 0.434123 | -0.00811 | 0.001013 | 1.20E-15 |
| rs8065350 | G | A | 0.242174 | 0.006606 | 0.001193 | 3.08E-08 |
| rs79147094 | A | C | 0.05655 | 0.012035 | 0.002178 | 3.28E-08 |
| rs115725560 | T | A | 0.184221 | 0.007719 | 0.001291 | 2.27E-09 |
| rs2301597 | T | C | 0.424094 | -0.0059 | 0.001016 | 6.35E-09 |
| rs1973407 | G | A | 0.273653 | -0.00745 | 0.001131 | 4.36E-11 |
| rs17637472 | G | A | 0.401434 | 0.00859 | 0.001029 | 7.04E-17 |
| rs3785837 | G | A | 0.231516 | 0.006576 | 0.001201 | 4.37E-08 |
| rs4459609 | C | A | 0.378925 | -0.00953 | 0.001035 | 3.10E-20 |
| rs1436138 | A | G | 0.35909 | -0.00812 | 0.001047 | 9.18E-15 |
| rs74439044 | T | C | 0.096542 | 0.012988 | 0.001693 | 1.68E-14 |
| rs56042504 | G | A | 0.140015 | 0.008141 | 0.00145 | 1.97E-08 |
| rs8093196 | G | T | 0.329042 | 0.007097 | 0.001069 | 3.18E-11 |
| rs72915163 | C | T | 0.257994 | 0.008742 | 0.001158 | 4.29E-14 |
| rs144020965 | G | A | 0.453585 | 0.00649 | 0.001006 | 1.13E-10 |
| rs2287922 | G | A | 0.461899 | 0.006323 | 0.001007 | 3.39E-10 |
| rs73046792 | G | A | 0.164553 | -0.00743 | 0.001349 | 3.56E-08 |
| rs12978472 | C | G | 0.129667 | -0.02059 | 0.001501 | 8.33E-43 |
| rs12463045 | T | C | 0.162809 | -0.00852 | 0.001372 | 5.26E-10 |
| rs6108787 | T | G | 0.47741 | 0.010302 | 0.001004 | 1.07E-24 |
| rs79384779 | C | T | 0.146734 | 0.008314 | 0.001431 | 6.30E-09 |
| rs6031431 | A | G | 0.461053 | 0.006524 | 0.00101 | 1.04E-10 |
| rs73306876 | A | G | 0.117588 | 0.019331 | 0.001561 | 3.28E-35 |
| rs8118848 | G | A | 0.236516 | -0.00873 | 0.00118 | 1.38E-13 |
| rs6108168 | C | A | 0.255259 | -0.01064 | 0.001148 | 1.80E-20 |
| rs2823139 | G | A | 0.33839 | 0.006156 | 0.001064 | 7.11E-09 |
| rs2298359 | T | C | 0.065974 | -0.01351 | 0.002017 | 2.10E-11 |
| rs631462 | G | A | 0.404691 | -0.00619 | 0.001036 | 2.28E-09 |
| rs10211901 | G | A | 0.444947 | 0.005639 | 0.001008 | 2.25E-08 |

**Table S15. Genome-wide significant and independent SNPs that were used as instruments for SBP**

| SNP | beta.e | se. | effect | other_ | eaf. | pval.exposure |
| --- | --- | --- | --- | --- | --- | --- |
| rs1006545 | 0.6846 | 0.048 | T | G | 0.1128 | 3.50E-46 |
| rs11191580 | 1.0995 | 0.055 | T | C | 0.0824 | 7.74E-89 |
| rs117464403 | 0.864 | 0.1199 | A | G | 0.9817 | 5.80E-13 |
| rs12255372 | 0.2358 | 0.0335 | T | G | 0.7117 | 1.94E-12 |
| rs1801253 | 0.4626 | 0.0344 | C | G | 0.2662 | 2.84E-41 |
| rs72842207 | -0.203 | 0.0367 | T | C | 0.7856 | 3.14E-08 |
| rs11592107 | 0.3024 | 0.0326 | A | G | 0.6904 | 1.55E-20 |
| rs7093894 | 0.236 | 0.0427 | A | C | 0.8488 | 3.16E-08 |
| rs7912283 | -0.2144 | 0.0322 | A | G | 0.3532 | 2.94E-11 |
| rs1133400 | -0.2975 | 0.0376 | A | G | 0.214 | 2.53E-15 |
| rs1623474 | 0.3827 | 0.0321 | T | C | 0.6697 | 7.66E-33 |
| rs12258967 | 0.6327 | 0.0337 | C | G | 0.2953 | 1.08E-78 |
| rs3802517 | 0.2527 | 0.0301 | A | T | 0.5382 | 4.65E-17 |
| rs12264186 | 0.2135 | 0.0387 | T | C | 0.8129 | 3.58E-08 |
| rs11252324 | -0.4164 | 0.0573 | T | G | 0.9229 | 3.61E-13 |
| rs4948643 | 0.2258 | 0.0338 | T | C | 0.7181 | 2.40E-11 |
| rs34130368 | -0.3016 | 0.0497 | T | G | 0.883 | 1.28E-09 |
| rs4245599 | -0.1794 | 0.0305 | A | G | 0.5416 | 4.04E-09 |
| rs57946343 | 0.716 | 0.0426 | T | C | 0.1473 | 2.10E-63 |
| rs2236295 | -0.3028 | 0.0309 | T | G | 0.6022 | 1.05E-22 |
| rs2177843 | 0.4394 | 0.0432 | T | C | 0.8495 | 2.80E-24 |
| rs10749572 | -0.203 | 0.0302 | T | G | 0.4556 | 1.88E-11 |
| rs111866816 | 0.3569 | 0.0597 | T | C | 0.9291 | 2.29E-09 |
| rs2689690 | -0.2702 | 0.0316 | T | C | 0.6322 | 1.15E-17 |
| rs2274224 | -0.4517 | 0.0304 | C | G | 0.5676 | 5.99E-50 |
| rs604723 | -0.655 | 0.0339 | T | C | 0.7244 | 2.55E-83 |
| rs629864 | -0.1868 | 0.0319 | T | C | 0.3503 | 4.69E-09 |
| rs7926110 | 0.2603 | 0.0321 | T | G | 0.3267 | 5.71E-16 |
| rs236916 | 0.3166 | 0.0446 | A | G | 0.8652 | 1.31E-12 |
| rs573455 | 0.1994 | 0.0303 | A | G | 0.539 | 4.77E-11 |
| rs11222084 | -0.3363 | 0.0316 | A | T | 0.3621 | 1.80E-26 |
| rs7944927 | 0.2235 | 0.0392 | T | C | 0.2181 | 1.23E-08 |
| rs2014408 | 0.5169 | 0.0373 | T | C | 0.7913 | 1.26E-43 |
| rs7926335 | 0.3135 | 0.0339 | T | C | 0.7309 | 2.52E-20 |
| rs569550 | -0.5765 | 0.0318 | T | G | 0.3963 | 1.33E-73 |
| rs74048190 | -0.4404 | 0.0757 | T | C | 0.0478 | 6.07E-09 |
| rs17762 | 0.4117 | 0.0571 | A | G | 0.9223 | 5.60E-13 |
| rs1382472 | -0.1917 | 0.0307 | A | G | 0.5959 | 4.47E-10 |
| rs871004 | 0.2336 | 0.0317 | A | G | 0.6519 | 1.65E-13 |
| rs10501122 | 0.1916 | 0.0315 | T | C | 0.361 | 1.18E-09 |
| rs11604310 | -0.2778 | 0.0411 | T | C | 0.8345 | 1.46E-11 |
| rs7107356 | -0.4598 | 0.0301 | A | G | 0.5041 | 1.63E-52 |
| rs2904315 | -0.2081 | 0.0325 | A | G | 0.6869 | 1.58E-10 |
| rs4427587 | 0.2062 | 0.0313 | T | C | 0.4381 | 4.28E-11 |
| rs7125196 | 0.4422 | 0.0472 | T | C | 0.1183 | 7.31E-21 |
| rs2306363 | -0.4358 | 0.0376 | T | G | 0.7955 | 5.24E-31 |
| rs7395791 | -0.2162 | 0.0308 | A | G | 0.5581 | 2.19E-12 |
| rs10501410 | 0.4122 | 0.0607 | A | G | 0.9308 | 1.10E-11 |
| rs7927515 | 0.2271 | 0.0319 | A | C | 0.6541 | 1.05E-12 |
| rs2289124 | -0.308 | 0.0415 | A | G | 0.8327 | 1.14E-13 |
| rs360153 | -0.3445 | 0.0306 | T | C | 0.5834 | 1.73E-29 |
| rs5742643 | -0.2233 | 0.0349 | T | C | 0.7513 | 1.53E-10 |
| rs7310615 | 0.585 | 0.0306 | C | G | 0.5184 | 1.32E-81 |
| rs1896326 | -0.2797 | 0.0371 | A | G | 0.7709 | 4.41E-14 |
| rs35444 | 0.4368 | 0.031 | A | G | 0.3862 | 3.47E-45 |
| rs6490019 | -0.2897 | 0.0309 | A | G | 0.6204 | 6.61E-21 |
| rs1169078 | -0.1971 | 0.0327 | C | G | 0.3121 | 1.68E-09 |
| rs2024385 | -0.2642 | 0.0306 | A | T | 0.576 | 5.88E-18 |
| rs117206641 | 0.3154 | 0.0499 | T | C | 0.8892 | 2.66E-10 |
| rs1010064 | 0.3571 | 0.0387 | A | C | 0.1837 | 3.02E-20 |
| rs73075659 | 0.3962 | 0.0321 | A | G | 0.3346 | 5.52E-35 |
| rs3819532 | -0.1875 | 0.0306 | T | C | 0.6087 | 9.44E-10 |
| rs2129869 | -0.2643 | 0.0361 | A | T | 0.2222 | 2.44E-13 |
| rs9651825 | -0.2042 | 0.034 | A | G | 0.2705 | 1.93E-09 |
| rs78998485 | -0.2449 | 0.0346 | C | G | 0.2557 | 1.48E-12 |
| rs61917655 | 0.3427 | 0.0514 | T | C | 0.8986 | 2.68E-11 |
| rs12426261 | 0.3775 | 0.0309 | A | G | 0.6208 | 2.31E-34 |
| rs7134440 | 0.4788 | 0.0562 | T | C | 0.9178 | 1.58E-17 |
| rs7134677 | -0.3851 | 0.0332 | T | C | 0.7022 | 4.46E-31 |
| rs7306710 | -0.2429 | 0.0303 | T | C | 0.519 | 1.03E-15 |
| rs4143175 | 0.2187 | 0.0352 | T | C | 0.7591 | 5.10E-10 |
| rs7963801 | -0.2362 | 0.0311 | T | C | 0.5779 | 2.87E-14 |
| rs6539467 | 0.265 | 0.0404 | A | G | 0.8339 | 5.57E-11 |
| rs17249754 | -0.8446 | 0.0403 | A | G | 0.8317 | 1.25E-97 |
| rs10777213 | -0.1786 | 0.0299 | A | G | 0.4756 | 2.45E-09 |
| rs9549627 | 0.2846 | 0.05 | A | G | 0.8825 | 1.25E-08 |
| rs7331680 | 0.4101 | 0.0423 | T | G | 0.8509 | 3.35E-22 |
| rs483071 | 0.2709 | 0.0313 | T | C | 0.3752 | 5.09E-18 |
| rs9507885 | -0.3208 | 0.0542 | T | C | 0.9047 | 3.23E-09 |
| rs9508495 | -0.3557 | 0.0353 | T | C | 0.2435 | 6.34E-24 |
| rs2065498 | -0.2934 | 0.0403 | T | G | 0.8294 | 3.36E-13 |
| rs7491248 | 0.2163 | 0.0362 | A | G | 0.7761 | 2.38E-09 |
| rs9526707 | -0.2039 | 0.0323 | A | G | 0.6784 | 2.77E-10 |
| rs75961402 | 0.2659 | 0.0418 | A | G | 0.8466 | 1.95E-10 |
| rs17245822 | -0.1899 | 0.0312 | A | C | 0.3733 | 1.15E-09 |
| rs78474310 | -0.4699 | 0.0734 | A | G | 0.0448 | 1.51E-10 |
| rs6562778 | 0.178 | 0.0304 | A | G | 0.5411 | 4.96E-09 |
| rs17562391 | 0.1967 | 0.0306 | T | C | 0.5814 | 1.35E-10 |
| rs75016974 | -0.2513 | 0.0439 | T | C | 0.8577 | 1.05E-08 |
| rs12885878 | -0.2291 | 0.0367 | A | G | 0.7663 | 4.32E-10 |
| rs365990 | 0.225 | 0.0312 | A | G | 0.3658 | 5.95E-13 |
| rs8904 | 0.3061 | 0.0314 | A | G | 0.6322 | 1.71E-22 |
| rs7493678 | -0.189 | 0.0316 | A | T | 0.3486 | 2.31E-09 |
| rs72683923 | 0.9587 | 0.1101 | T | C | 0.0212 | 3.08E-18 |
| rs35413927 | -0.3002 | 0.0328 | A | G | 0.3054 | 5.25E-20 |
| rs57140819 | 0.2415 | 0.0398 | C | G | 0.1732 | 1.30E-09 |
| rs11847049 | -0.2272 | 0.0364 | C | G | 0.2166 | 4.44E-10 |
| rs11159091 | 0.1978 | 0.0303 | A | G | 0.5385 | 6.79E-11 |
| rs7154723 | 0.253 | 0.0309 | A | G | 0.615 | 2.72E-16 |
| rs4606697 | -0.3196 | 0.0523 | A | G | 0.8959 | 9.71E-10 |
| rs8030856 | -0.1764 | 0.031 | C | G | 0.3953 | 1.21E-08 |
| rs28866311 | -0.2762 | 0.0302 | T | G | 0.4737 | 5.45E-20 |
| rs4775769 | -0.4162 | 0.0517 | T | G | 0.9055 | 7.76E-16 |
| rs3098186 | -0.2422 | 0.0303 | T | C | 0.4844 | 1.41E-15 |
| rs2652812 | -0.2516 | 0.0353 | T | C | 0.2456 | 1.03E-12 |
| rs28429256 | 0.215 | 0.0325 | A | G | 0.6658 | 3.89E-11 |
| rs11636952 | 0.5313 | 0.0328 | T | C | 0.6859 | 4.22E-59 |
| rs2627313 | 0.3208 | 0.0303 | T | C | 0.5546 | 3.55E-26 |
| rs2046341 | -0.2542 | 0.0382 | A | G | 0.8079 | 2.74E-11 |
| rs77032376 | -0.2727 | 0.043 | T | C | 0.8515 | 2.35E-10 |
| rs4932373 | -0.635 | 0.0328 | A | C | 0.3258 | 2.49E-83 |
| rs12906962 | -0.2653 | 0.0325 | T | C | 0.324 | 3.28E-16 |
| rs2589218 | -0.2258 | 0.0339 | T | C | 0.2703 | 2.54E-11 |
| rs11641374 | -0.1943 | 0.0309 | A | C | 0.4005 | 3.26E-10 |
| rs77924615 | -0.4081 | 0.039 | A | G | 0.8014 | 1.12E-25 |
| rs12596630 | 0.4278 | 0.0547 | T | C | 0.9097 | 5.01E-15 |
| rs7186298 | -0.2315 | 0.0302 | T | C | 0.5705 | 1.88E-14 |
| rs8044992 | 0.2138 | 0.0331 | T | C | 0.2877 | 1.07E-10 |
| rs72778133 | -0.2417 | 0.0443 | T | C | 0.1422 | 4.98E-08 |
| rs111929315 | 0.3146 | 0.0485 | A | G | 0.1083 | 8.60E-11 |
| rs12446456 | -0.3003 | 0.0302 | T | C | 0.5726 | 2.97E-23 |
| rs34941092 | -0.3225 | 0.0425 | A | G | 0.8502 | 3.23E-14 |
| rs4784541 | -0.2015 | 0.0307 | T | C | 0.5252 | 4.93E-11 |
| rs2060664 | 0.216 | 0.0345 | T | C | 0.2516 | 4.06E-10 |
| rs146550789 | -0.4824 | 0.0778 | T | C | 0.0417 | 5.64E-10 |
| rs62047964 | 0.5115 | 0.0686 | T | C | 0.9378 | 9.29E-14 |
| rs1012089 | -0.192 | 0.0302 | C | G | 0.5248 | 1.95E-10 |
| rs4888408 | 0.3653 | 0.0307 | A | G | 0.4145 | 1.42E-32 |
| rs12926550 | -0.2548 | 0.0324 | A | G | 0.6844 | 3.43E-15 |
| rs3950627 | 0.1851 | 0.0308 | A | C | 0.469 | 1.82E-09 |
| rs6540119 | 0.2016 | 0.0322 | A | T | 0.666 | 3.93E-10 |
| rs908951 | -0.2261 | 0.0315 | T | C | 0.5622 | 7.14E-13 |
| rs9303175 | -0.2048 | 0.0327 | T | G | 0.6537 | 3.65E-10 |
| rs4925159 | 0.2174 | 0.0305 | A | G | 0.5754 | 9.66E-13 |
| rs7218708 | -0.1781 | 0.0303 | A | G | 0.5169 | 4.38E-09 |
| rs11653927 | -0.2796 | 0.0308 | T | C | 0.6155 | 1.17E-19 |
| rs1551355 | 0.2098 | 0.0356 | T | C | 0.7666 | 3.89E-09 |
| rs9899540 | 0.2011 | 0.0316 | A | T | 0.6001 | 1.87E-10 |
| rs7213273 | -0.4 | 0.0315 | A | G | 0.345 | 6.24E-37 |
| rs17608766 | -0.6903 | 0.0433 | T | C | 0.1445 | 2.48E-57 |
| rs3764400 | 0.3748 | 0.0445 | T | C | 0.1365 | 3.69E-17 |
| rs9897429 | 0.2645 | 0.0319 | A | G | 0.48 | 1.19E-16 |
| rs1000423 | 0.4138 | 0.0346 | T | C | 0.2684 | 6.50E-33 |
| rs56288724 | -0.2178 | 0.031 | A | G | 0.4169 | 2.01E-12 |
| rs62076622 | 0.2363 | 0.0377 | A | G | 0.1987 | 3.79E-10 |
| rs6504213 | -0.2982 | 0.0312 | T | C | 0.5818 | 1.25E-21 |
| rs113086489 | 0.3249 | 0.0307 | T | C | 0.4475 | 3.80E-26 |
| rs4511593 | -0.2881 | 0.0318 | T | C | 0.3472 | 1.28E-19 |
| rs1436138 | 0.3119 | 0.0315 | A | G | 0.3633 | 4.73E-23 |
| rs9302885 | 0.2242 | 0.0302 | A | G | 0.5548 | 1.03E-13 |
| rs117285318 | 0.4413 | 0.0589 | T | C | 0.0775 | 6.93E-14 |
| rs11655604 | -0.2033 | 0.0333 | T | C | 0.6421 | 1.09E-09 |
| rs62082230 | -0.1884 | 0.0345 | A | T | 0.7227 | 4.69E-08 |
| rs1154214 | -0.2031 | 0.0306 | T | G | 0.6037 | 3.27E-11 |
| rs56407827 | 0.3603 | 0.034 | T | C | 0.7313 | 2.78E-26 |
| rs11874246 | 0.2856 | 0.0328 | T | C | 0.7037 | 3.23E-18 |
| rs7245140 | -0.3367 | 0.0391 | T | C | 0.1802 | 7.67E-18 |
| rs1437649 | -0.2189 | 0.0357 | A | G | 0.7655 | 8.57E-10 |
| rs665445 | -0.1909 | 0.0334 | A | C | 0.7206 | 1.15E-08 |
| rs10048404 | -0.2607 | 0.0317 | T | C | 0.6299 | 1.91E-16 |
| rs10460108 | 0.2141 | 0.0301 | A | G | 0.5199 | 1.12E-12 |
| rs34413141 | -0.3531 | 0.0393 | A | T | 0.8178 | 2.47E-19 |
| rs167479 | -0.5642 | 0.0327 | T | G | 0.5274 | 7.21E-67 |
| rs698748 | 0.1871 | 0.0325 | A | G | 0.579 | 8.90E-09 |
| rs1077795 | 0.2507 | 0.0344 | A | G | 0.2607 | 3.33E-13 |
| rs149339216 | -0.6912 | 0.0779 | T | C | 0.0434 | 6.93E-19 |
| rs62112908 | -0.2388 | 0.0419 | A | G | 0.1536 | 1.25E-08 |
| rs28572357 | -0.2733 | 0.0308 | A | C | 0.3977 | 6.34E-19 |
| rs1433121 | -0.228 | 0.0326 | T | C | 0.3094 | 2.66E-12 |
| rs33836 | 0.1766 | 0.0304 | T | C | 0.5378 | 6.56E-09 |
| rs10420519 | -0.4921 | 0.0887 | T | G | 0.9653 | 2.86E-08 |
| rs7255933 | 0.2306 | 0.0345 | A | G | 0.7426 | 2.44E-11 |
| rs11672660 | 0.2212 | 0.0381 | T | C | 0.8004 | 6.32E-09 |
| rs571689 | 0.228 | 0.0304 | T | C | 0.4804 | 6.77E-14 |
| rs73046792 | -0.3554 | 0.0426 | A | G | 0.8412 | 7.23E-17 |
| rs68096471 | -0.2098 | 0.0343 | A | G | 0.7341 | 9.26E-10 |
| rs12985940 | 0.4642 | 0.0434 | T | C | 0.1592 | 1.08E-26 |
| rs488834 | -0.3799 | 0.0365 | T | C | 0.2355 | 2.35E-25 |
| rs10776752 | 0.8211 | 0.0576 | T | G | 0.9191 | 4.61E-46 |
| rs59980837 | 1.0997 | 0.1163 | T | G | 0.9822 | 3.32E-21 |
| rs6699618 | 0.9115 | 0.041 | C | G | 0.1599 | ####### |
| rs11585169 | 0.1796 | 0.0308 | A | T | 0.4227 | 5.34E-09 |
| rs76719272 | -0.2738 | 0.0461 | T | C | 0.8688 | 2.97E-09 |
| rs75461554 | -0.3016 | 0.0377 | T | C | 0.7993 | 1.18E-15 |
| rs1889785 | 0.1782 | 0.0304 | A | G | 0.5448 | 4.35E-09 |
| rs7796 | 0.3385 | 0.0314 | C | G | 0.4886 | 5.00E-27 |
| rs12731646 | -0.189 | 0.0307 | T | C | 0.591 | 7.21E-10 |
| rs1043069 | 0.234 | 0.0311 | T | G | 0.3844 | 5.26E-14 |
| rs4651224 | 0.1986 | 0.0306 | T | C | 0.5526 | 9.00E-11 |
| rs12042924 | -0.1807 | 0.0303 | T | C | 0.4716 | 2.62E-09 |
| rs11120093 | -0.1792 | 0.0307 | T | C | 0.5918 | 5.13E-09 |
| rs2724377 | 0.1938 | 0.0301 | A | G | 0.4697 | 1.29E-10 |
| rs7555285 | 0.2294 | 0.0376 | C | G | 0.1989 | 1.05E-09 |
| rs263532 | 0.1798 | 0.0307 | T | C | 0.4245 | 4.72E-09 |
| rs68085857 | 0.274 | 0.0357 | T | C | 0.766 | 1.68E-14 |
| rs4595370 | -0.2092 | 0.0328 | A | G | 0.6988 | 1.73E-10 |
| rs1745417 | 0.2871 | 0.0301 | T | C | 0.4799 | 1.59E-21 |
| rs699 | -0.3748 | 0.0308 | A | G | 0.4072 | 5.59E-34 |
| rs1565440 | 0.1746 | 0.0311 | A | G | 0.6248 | 1.94E-08 |
| rs4926499 | 0.2965 | 0.0438 | C | G | 0.1737 | 1.33E-11 |
| rs404100 | 0.1935 | 0.0303 | T | C | 0.5487 | 1.68E-10 |
| rs34079867 | 0.1992 | 0.0354 | T | C | 0.734 | 1.78E-08 |
| rs4908348 | 0.2366 | 0.033 | T | G | 0.3056 | 8.07E-13 |
| rs2493296 | 0.4183 | 0.0442 | T | C | 0.8575 | 3.14E-21 |
| rs11210029 | -0.203 | 0.0313 | A | G | 0.3678 | 8.92E-11 |
| rs1408945 | -0.3196 | 0.0304 | T | G | 0.5757 | 8.33E-26 |
| rs1209384 | 0.2558 | 0.0313 | A | G | 0.6122 | 2.85E-16 |
| rs778124 | 0.2965 | 0.0311 | A | G | 0.6264 | 1.45E-21 |
| rs61772592 | -0.3181 | 0.0455 | A | G | 0.1255 | 2.86E-12 |
| rs12063372 | 0.1989 | 0.0318 | A | G | 0.6154 | 3.86E-10 |
| rs2232460 | -0.2171 | 0.032 | A | G | 0.6657 | 1.10E-11 |
| rs12136922 | 0.2027 | 0.0304 | A | G | 0.5051 | 2.69E-11 |
| rs658780 | -0.2028 | 0.0347 | T | G | 0.2553 | 5.29E-09 |
| rs786923 | -0.3082 | 0.031 | T | C | 0.3761 | 2.83E-23 |
| rs7514579 | 0.2243 | 0.0361 | A | C | 0.2288 | 5.45E-10 |
| rs2423514 | 0.3011 | 0.0302 | A | G | 0.4589 | 1.77E-23 |
| rs6108787 | -0.4274 | 0.03 | T | G | 0.4704 | 5.38E-46 |
| rs6078093 | -0.1849 | 0.0304 | A | G | 0.572 | 1.20E-09 |
| rs8125763 | 0.1761 | 0.0301 | A | C | 0.5283 | 4.84E-09 |
| rs17812022 | -0.3613 | 0.0525 | T | C | 0.9042 | 5.65E-12 |
| rs6058088 | 0.2832 | 0.0417 | T | G | 0.1561 | 1.14E-11 |
| rs79384779 | 0.3179 | 0.0428 | T | C | 0.8488 | 1.08E-13 |
| rs6029756 | -0.2712 | 0.033 | A | G | 0.6775 | 1.88E-16 |
| rs6031431 | -0.2617 | 0.0304 | A | G | 0.4624 | 7.05E-18 |
| rs2598 | 0.168 | 0.0303 | A | G | 0.467 | 2.87E-08 |
| rs6090907 | -0.3854 | 0.0425 | A | G | 0.853 | 1.29E-19 |
| rs234623 | -0.1804 | 0.0302 | A | G | 0.4959 | 2.43E-09 |
| rs6026744 | -0.7131 | 0.0461 | A | T | 0.1229 | 7.00E-54 |
| rs28374392 | 0.1924 | 0.0338 | T | C | 0.3769 | 1.21E-08 |
| rs6062324 | -0.3294 | 0.0363 | A | G | 0.7636 | 1.18E-19 |
| rs6054139 | 0.2094 | 0.0306 | A | G | 0.394 | 8.23E-12 |
| rs2776037 | -0.1851 | 0.0309 | T | C | 0.5849 | 2.15E-09 |
| rs1882961 | 0.2443 | 0.0326 | T | C | 0.6913 | 6.69E-14 |
| rs2833834 | 0.2177 | 0.0338 | A | C | 0.7235 | 1.22E-10 |
| rs12627651 | 0.3498 | 0.0341 | A | G | 0.7128 | 1.02E-24 |
| rs34487963 | -0.8819 | 0.1244 | A | C | 0.9815 | 1.35E-12 |
| rs7278003 | -0.1876 | 0.0304 | T | C | 0.5622 | 6.63E-10 |
| rs2238787 | 0.2552 | 0.0332 | A | G | 0.708 | 1.45E-14 |
| rs12321 | -0.2292 | 0.0303 | C | G | 0.5672 | 3.81E-14 |
| rs113264678 | 0.4063 | 0.0727 | T | C | 0.954 | 2.26E-08 |
| rs8142376 | 0.1676 | 0.03 | T | C | 0.509 | 2.20E-08 |
| rs148140538 | -0.3252 | 0.0562 | T | C | 0.9192 | 7.39E-09 |
| rs28578714 | 0.2066 | 0.0327 | T | C | 0.3938 | 2.53E-10 |
| rs10207726 | -0.2142 | 0.033 | T | C | 0.704 | 8.06E-11 |
| rs6737318 | 0.2348 | 0.0364 | A | G | 0.2218 | 1.13E-10 |
| rs2580350 | 0.1769 | 0.0307 | A | G | 0.4391 | 8.39E-09 |
| rs17257081 | 0.2274 | 0.0392 | A | G | 0.1935 | 6.35E-09 |
| rs55944332 | -0.2613 | 0.0355 | A | G | 0.2368 | 1.79E-13 |
| rs62170470 | 0.1972 | 0.0321 | T | C | 0.3983 | 7.69E-10 |
| rs62187653 | 0.3286 | 0.0511 | T | C | 0.0971 | 1.23E-10 |
| rs4667454 | 0.2636 | 0.0322 | A | G | 0.3295 | 2.63E-16 |
| rs73029563 | -0.514 | 0.0304 | C | G | 0.5451 | 4.20E-64 |
| rs10048760 | -0.1862 | 0.0301 | T | G | 0.4712 | 6.56E-10 |
| rs71421551 | 0.2374 | 0.0333 | C | G | 0.7115 | 1.05E-12 |
| rs17610485 | 0.1738 | 0.0304 | A | T | 0.4239 | 1.06E-08 |
| rs4894132 | 0.2469 | 0.0342 | T | C | 0.2717 | 5.51E-13 |
| rs12473915 | -0.295 | 0.0375 | A | G | 0.7983 | 3.42E-15 |
| rs13412750 | -0.2889 | 0.0341 | A | G | 0.7292 | 2.33E-17 |
| rs17760259 | -0.2654 | 0.0304 | T | C | 0.4276 | 2.25E-18 |
| rs12693982 | 0.2575 | 0.0309 | T | C | 0.5976 | 7.49E-17 |
| rs3845811 | -0.2942 | 0.0309 | C | G | 0.4339 | 1.88E-21 |
| rs12694277 | -0.2018 | 0.0335 | T | C | 0.7054 | 1.80E-09 |
| rs2161967 | 0.2836 | 0.0307 | T | G | 0.5721 | 2.87E-20 |
| rs3828282 | 0.1857 | 0.0318 | C | G | 0.5721 | 5.29E-09 |
| rs10804330 | 0.2351 | 0.0306 | T | C | 0.4332 | 1.62E-14 |
| rs1044822 | -0.248 | 0.0424 | T | C | 0.8518 | 5.16E-09 |
| rs3754944 | 0.1768 | 0.0308 | A | C | 0.4125 | 9.30E-09 |
| rs145042302 | -0.5886 | 0.0972 | A | G | 0.97 | 1.39E-09 |
| rs2384063 | 0.3266 | 0.0357 | T | C | 0.2393 | 6.33E-20 |
| rs1275988 | -0.541 | 0.0308 | T | C | 0.3888 | 4.42E-69 |
| rs13420463 | 0.3143 | 0.036 | A | G | 0.2266 | 2.72E-18 |
| rs4952609 | 0.2124 | 0.0347 | A | G | 0.2561 | 9.60E-10 |
| rs115262049 | 0.5893 | 0.0552 | A | T | 0.0868 | 1.29E-26 |
| rs12464602 | -0.2437 | 0.0315 | A | G | 0.3792 | 1.02E-14 |
| rs13016772 | 0.2522 | 0.0355 | T | C | 0.2349 | 1.23E-12 |
| rs2249105 | 0.2927 | 0.0313 | A | G | 0.3679 | 7.63E-21 |
| rs10188003 | 0.1883 | 0.0307 | T | C | 0.607 | 8.80E-10 |
| rs6731373 | 0.1913 | 0.0326 | A | G | 0.6508 | 4.18E-09 |
| rs6732123 | -0.1737 | 0.0307 | C | G | 0.5826 | 1.52E-08 |
| rs4577304 | -0.1767 | 0.0302 | T | C | 0.4767 | 4.99E-09 |
| rs72847885 | 0.2413 | 0.0318 | A | G | 0.337 | 3.08E-14 |
| rs9848170 | 0.3231 | 0.0307 | C | G | 0.403 | 7.01E-26 |
| rs12637573 | -0.1731 | 0.0302 | A | G | 0.5282 | 9.95E-09 |
| rs6438857 | 0.2736 | 0.0305 | T | C | 0.4226 | 3.13E-19 |
| rs9880098 | 0.3081 | 0.0308 | A | G | 0.6054 | 1.59E-23 |
| rs1199330 | -0.2654 | 0.047 | A | G | 0.1176 | 1.65E-08 |
| rs9876694 | 0.4713 | 0.0651 | T | C | 0.9416 | 4.64E-13 |
| rs11925504 | -0.2901 | 0.0305 | A | G | 0.4279 | 1.78E-21 |
| rs4408839 | -0.2301 | 0.0345 | A | G | 0.2567 | 2.43E-11 |
| rs79539362 | 0.4003 | 0.0504 | T | C | 0.1008 | 2.09E-15 |
| rs17684859 | -0.2241 | 0.034 | T | C | 0.2665 | 4.24E-11 |
| rs3980686 | -0.4998 | 0.0487 | T | G | 0.8925 | 1.03E-24 |
| rs1290784 | 0.4124 | 0.0303 | T | C | 0.5517 | 2.97E-42 |
| rs2111557 | 0.1764 | 0.0302 | T | C | 0.5325 | 5.22E-09 |
| rs4955575 | 0.2158 | 0.0348 | A | C | 0.2539 | 5.63E-10 |
| rs262986 | -0.2371 | 0.0305 | A | G | 0.5296 | 7.67E-15 |
| rs13091418 | -0.2234 | 0.0325 | C | G | 0.3341 | 6.15E-12 |
| rs9869437 | -0.2001 | 0.0318 | A | C | 0.6477 | 3.22E-10 |
| rs189267552 | -0.8664 | 0.139 | A | T | 0.9868 | 4.55E-10 |
| rs2643826 | 0.4473 | 0.0306 | T | C | 0.5495 | 1.74E-48 |
| rs68115553 | -0.6445 | 0.1143 | A | G | 0.0199 | 1.74E-08 |
| rs743395 | 0.2597 | 0.0317 | T | C | 0.6166 | 2.55E-16 |
| rs6788984 | 0.2999 | 0.0432 | A | G | 0.1437 | 3.81E-12 |
| rs1052501 | 0.2262 | 0.0412 | T | C | 0.1671 | 4.14E-08 |
| rs6771917 | -0.3793 | 0.0355 | T | C | 0.7523 | 1.39E-26 |
| rs7615099 | 0.1891 | 0.0321 | A | G | 0.3325 | 3.90E-09 |
| rs6445583 | 0.2774 | 0.0349 | A | G | 0.2535 | 1.90E-15 |
| rs3772219 | 0.2733 | 0.0324 | A | C | 0.3176 | 3.10E-17 |
| rs7618284 | -0.1891 | 0.0331 | C | G | 0.6606 | 1.10E-08 |
| rs4499560 | -0.2199 | 0.0326 | A | T | 0.6829 | 1.46E-11 |
| rs1375564 | 0.2579 | 0.0315 | T | C | 0.3605 | 2.84E-16 |
| rs13107325 | -0.9086 | 0.0592 | T | C | 0.9261 | 4.22E-53 |
| rs11097909 | -0.3628 | 0.043 | T | C | 0.8528 | 3.35E-17 |
| rs1493132 | -0.1766 | 0.0318 | T | C | 0.3397 | 2.73E-08 |
| rs1814951 | -0.3231 | 0.0466 | A | G | 0.1215 | 3.91E-12 |
| rs4834792 | 0.1973 | 0.0303 | A | T | 0.5204 | 7.24E-11 |
| rs7439567 | 0.2537 | 0.0309 | T | C | 0.5894 | 2.31E-16 |
| rs72719160 | -0.2243 | 0.0324 | A | T | 0.3171 | 4.34E-12 |
| rs2353940 | -0.2075 | 0.0358 | T | C | 0.2493 | 6.85E-09 |
| rs73855810 | 0.2732 | 0.0434 | A | G | 0.8594 | 3.04E-10 |
| rs7683728 | -0.3654 | 0.0304 | T | C | 0.4688 | 2.43E-33 |
| rs12643599 | 0.3134 | 0.0313 | A | G | 0.3605 | 1.23E-23 |
| rs17035181 | 0.3074 | 0.0429 | T | G | 0.1448 | 7.61E-13 |
| rs869396 | -0.2115 | 0.0305 | A | C | 0.5341 | 4.12E-12 |
| rs2610990 | -0.2903 | 0.0343 | A | G | 0.7359 | 2.86E-17 |
| rs34535756 | 0.478 | 0.0786 | T | C | 0.9606 | 1.18E-09 |
| rs1290933 | -0.2847 | 0.0327 | A | C | 0.3081 | 3.17E-18 |
| rs55924432 | 0.2651 | 0.0317 | T | C | 0.599 | 5.70E-17 |
| rs2498323 | 0.3171 | 0.0517 | A | G | 0.902 | 8.52E-10 |
| rs2291434 | -0.2622 | 0.0303 | T | G | 0.4665 | 5.10E-18 |
| rs12511987 | -0.2329 | 0.0399 | T | G | 0.1774 | 5.39E-09 |
| rs62309747 | -0.2244 | 0.0304 | A | G | 0.5266 | 1.59E-13 |
| rs60991988 | 0.3789 | 0.0498 | T | G | 0.1069 | 2.82E-14 |
| rs13107261 | -0.1778 | 0.0314 | A | G | 0.6313 | 1.57E-08 |
| rs5020545 | -0.2179 | 0.0305 | T | C | 0.5563 | 9.71E-13 |
| rs12509595 | -0.8367 | 0.0334 | T | C | 0.2923 | ####### |
| rs6823199 | 0.2094 | 0.0348 | T | C | 0.2562 | 1.72E-09 |
| rs17010957 | -0.534 | 0.043 | T | C | 0.1463 | 1.78E-35 |
| rs13149209 | 0.281 | 0.0367 | T | C | 0.2227 | 1.97E-14 |
| rs11241313 | -0.2071 | 0.0326 | T | C | 0.6888 | 2.23E-10 |
| rs1624823 | 0.3371 | 0.0313 | A | G | 0.6199 | 4.26E-27 |
| rs9327297 | 0.2747 | 0.0319 | C | G | 0.3324 | 8.07E-18 |
| rs758179 | -0.2091 | 0.0367 | C | G | 0.2244 | 1.19E-08 |
| rs6892983 | 0.3427 | 0.0307 | A | C | 0.5978 | 7.11E-29 |
| rs10069690 | 0.3098 | 0.0369 | T | C | 0.7418 | 4.47E-17 |
| rs702395 | 0.2318 | 0.0305 | T | C | 0.5631 | 3.24E-14 |
| rs2913920 | 0.2418 | 0.0359 | T | C | 0.235 | 1.62E-11 |
| rs7725413 | -0.1985 | 0.0359 | T | C | 0.2301 | 3.07E-08 |
| rs1957563 | 0.3629 | 0.0342 | T | C | 0.735 | 2.32E-26 |
| rs11960210 | 0.4727 | 0.0313 | T | C | 0.3755 | 1.25E-51 |
| rs13358657 | -0.388 | 0.0445 | A | G | 0.1332 | 2.95E-18 |
| rs3860770 | -0.2663 | 0.0333 | A | G | 0.7084 | 1.20E-15 |
| rs12153395 | -0.3303 | 0.0486 | A | G | 0.8853 | 1.07E-11 |
| rs12656497 | -0.6382 | 0.0307 | T | C | 0.5966 | 7.14E-96 |
| rs10941043 | -0.2585 | 0.0332 | T | G | 0.2902 | 6.42E-15 |
| rs4957026 | 0.1982 | 0.0323 | A | G | 0.6601 | 8.12E-10 |
| rs2113077 | 0.2097 | 0.0305 | A | G | 0.5697 | 6.09E-12 |
| rs1694068 | 0.2657 | 0.0311 | A | T | 0.3861 | 1.18E-17 |
| rs10043077 | -0.1931 | 0.0324 | T | C | 0.361 | 2.52E-09 |
| rs34496659 | 0.4545 | 0.0616 | A | G | 0.9298 | 1.54E-13 |
| rs6870654 | 0.2136 | 0.0347 | T | C | 0.2546 | 7.58E-10 |
| rs4286632 | 0.211 | 0.0343 | A | G | 0.2694 | 7.64E-10 |
| rs7703560 | -0.2246 | 0.0333 | A | G | 0.2998 | 1.51E-11 |
| rs246973 | 0.2479 | 0.0335 | T | C | 0.7118 | 1.45E-13 |
| rs6452769 | -0.3143 | 0.0377 | A | G | 0.7947 | 7.82E-17 |
| rs76443575 | -0.5233 | 0.0816 | C | G | 0.9641 | 1.40E-10 |
| rs1871190 | 0.1954 | 0.0324 | T | G | 0.6651 | 1.66E-09 |
| rs9486916 | 0.2657 | 0.0385 | T | C | 0.8021 | 5.42E-12 |
| rs961764 | -0.1909 | 0.0305 | C | G | 0.5746 | 3.75E-10 |
| rs1630736 | -0.1706 | 0.0309 | T | C | 0.535 | 3.52E-08 |
| rs10782230 | 0.2106 | 0.0302 | A | G | 0.5155 | 2.91E-12 |
| rs9401913 | 0.5202 | 0.0305 | A | G | 0.5613 | 3.66E-65 |
| rs9349379 | 0.2664 | 0.0312 | A | G | 0.407 | 1.31E-17 |
| rs2327429 | 0.2 | 0.0338 | T | C | 0.2917 | 3.16E-09 |
| rs8180684 | 0.2134 | 0.0335 | T | C | 0.7104 | 1.80E-10 |
| rs7765526 | 0.201 | 0.0307 | A | G | 0.5367 | 5.88E-11 |
| rs17080102 | -0.8085 | 0.0594 | C | G | 0.9306 | 3.52E-42 |
| rs1293969 | -0.1988 | 0.0347 | T | C | 0.2516 | 1.03E-08 |
| rs509833 | 0.329 | 0.044 | A | G | 0.8614 | 7.08E-14 |
| rs2745599 | 0.2164 | 0.0317 | A | G | 0.448 | 8.96E-12 |
| rs12661036 | -0.2104 | 0.0374 | T | C | 0.225 | 1.82E-08 |
| rs7744902 | -0.4088 | 0.0593 | A | G | 0.9234 | 5.64E-12 |
| rs9368222 | 0.2281 | 0.0339 | A | C | 0.7312 | 1.84E-11 |
| rs2655445 | -0.2018 | 0.0312 | A | G | 0.3933 | 9.58E-11 |
| rs79782817 | 0.5324 | 0.0499 | T | G | 0.8973 | 1.43E-26 |
| rs116025100 | 0.5365 | 0.0851 | A | G | 0.9617 | 2.86E-10 |
| rs2753960 | 0.4466 | 0.0309 | T | G | 0.5801 | 2.66E-47 |
| rs2815063 | 0.2755 | 0.0458 | A | C | 0.8685 | 1.76E-09 |
| rs7763558 | 0.3363 | 0.0321 | A | G | 0.6759 | 1.17E-25 |
| rs11967262 | -0.1715 | 0.0311 | C | G | 0.4868 | 3.43E-08 |
| rs78648104 | -0.4287 | 0.0541 | T | C | 0.0925 | 2.37E-15 |
| rs1575290 | 0.1973 | 0.0301 | T | C | 0.5267 | 5.59E-11 |
| rs1984195 | 0.2409 | 0.0303 | A | G | 0.5113 | 1.77E-15 |
| rs9361836 | 0.2196 | 0.0324 | T | C | 0.6828 | 1.25E-11 |
| rs6921291 | 0.3575 | 0.0385 | T | C | 0.8093 | 1.58E-20 |
| rs2392929 | -0.7507 | 0.0379 | T | G | 0.2027 | 1.96E-87 |
| rs34072724 | -0.2422 | 0.0303 | A | G | 0.5111 | 1.37E-15 |
| rs35680304 | 0.2694 | 0.031 | T | C | 0.4071 | 3.76E-18 |
| rs75672964 | 0.5885 | 0.0839 | T | C | 0.9582 | 2.35E-12 |
| rs73727605 | 0.3616 | 0.0623 | A | G | 0.9337 | 6.60E-09 |
| rs3918226 | 0.664 | 0.0575 | T | C | 0.9189 | 8.46E-31 |
| rs10224210 | -0.3831 | 0.034 | T | C | 0.2789 | 1.60E-29 |
| rs1870735 | 0.206 | 0.0311 | C | G | 0.5469 | 3.61E-11 |
| rs3807925 | -0.1859 | 0.0319 | A | G | 0.3504 | 5.39E-09 |
| rs28688791 | -0.3222 | 0.038 | T | C | 0.1982 | 2.34E-17 |
| rs6959688 | -0.2344 | 0.031 | A | G | 0.4019 | 4.22E-14 |
| rs112509803 | -0.2641 | 0.0477 | C | G | 0.8862 | 3.18E-08 |
| rs10282122 | -0.302 | 0.0327 | T | C | 0.3316 | 2.46E-20 |
| rs3735533 | -0.91 | 0.0577 | T | C | 0.9257 | 5.29E-56 |
| rs6961048 | -0.5304 | 0.0497 | C | G | 0.104 | 1.43E-26 |
| rs977184 | -0.184 | 0.0314 | T | C | 0.3748 | 4.86E-09 |
| rs11977526 | -0.3213 | 0.0312 | A | G | 0.5991 | 6.62E-25 |
| rs73049928 | -0.2382 | 0.0392 | A | G | 0.1939 | 1.20E-09 |
| rs12668436 | -0.2151 | 0.035 | T | C | 0.2459 | 7.88E-10 |
| rs848445 | -0.2025 | 0.0339 | T | C | 0.7149 | 2.28E-09 |
| rs42377 | -0.3153 | 0.0331 | A | G | 0.6955 | 1.69E-21 |
| rs79069610 | -0.4005 | 0.0727 | T | C | 0.05 | 3.68E-08 |
| rs35783704 | -0.4619 | 0.0507 | A | G | 0.8958 | 8.81E-20 |
| rs1821002 | 0.3794 | 0.0307 | C | G | 0.5892 | 5.19E-35 |
| rs7830607 | -0.206 | 0.0327 | A | G | 0.6954 | 3.09E-10 |
| rs2470004 | -0.3454 | 0.0392 | T | C | 0.1825 | 1.28E-18 |
| rs4598218 | 0.1911 | 0.0313 | T | C | 0.3842 | 1.00E-09 |
| rs7012866 | -0.2325 | 0.0301 | T | G | 0.5009 | 1.21E-14 |
| rs4440615 | -0.2201 | 0.0312 | A | G | 0.3679 | 1.87E-12 |
| rs4961293 | 0.2268 | 0.0303 | T | C | 0.5487 | 7.35E-14 |
| rs7463212 | -0.2753 | 0.0305 | A | T | 0.4555 | 1.81E-19 |
| rs71499040 | 0.2215 | 0.0338 | C | G | 0.2924 | 5.63E-11 |
| rs10866828 | 0.2476 | 0.0355 | T | C | 0.7504 | 3.19E-12 |
| rs7821832 | 0.4222 | 0.0348 | T | G | 0.2553 | 6.67E-34 |
| rs77375686 | -0.3467 | 0.0485 | A | G | 0.1117 | 8.38E-13 |
| rs1906672 | 0.2966 | 0.0358 | A | G | 0.7681 | 1.20E-16 |
| rs4873492 | 0.3431 | 0.0403 | T | C | 0.8276 | 1.61E-17 |
| rs2354862 | 0.2507 | 0.0317 | A | C | 0.3593 | 2.42E-15 |
| rs13253358 | 0.2127 | 0.033 | T | C | 0.7021 | 1.13E-10 |
| rs2126474 | -0.2601 | 0.0306 | T | G | 0.5875 | 1.87E-17 |
| rs9918879 | -0.2984 | 0.0499 | T | G | 0.897 | 2.28E-09 |
| rs148401029 | -0.4623 | 0.0848 | A | C | 0.9648 | 4.97E-08 |
| rs10091532 | -0.2067 | 0.0305 | A | C | 0.5832 | 1.33E-11 |
| rs843093 | -0.2085 | 0.0338 | A | G | 0.2912 | 6.95E-10 |
| rs2613203 | -0.2681 | 0.0389 | A | T | 0.1852 | 5.81E-12 |
| rs10980408 | -0.7606 | 0.0827 | T | C | 0.0359 | 3.83E-20 |
| rs2900568 | -0.1889 | 0.03 | T | C | 0.4816 | 2.96E-10 |
| rs34025993 | 0.223 | 0.0308 | A | G | 0.586 | 4.71E-13 |
| rs7854147 | 0.3056 | 0.0461 | A | G | 0.123 | 3.29E-11 |
| rs13289468 | 0.2488 | 0.0306 | A | C | 0.4257 | 3.93E-16 |
| rs6271 | -0.5547 | 0.0611 | T | C | 0.9265 | 1.18E-19 |
| rs11145807 | 0.2135 | 0.0322 | A | G | 0.5943 | 3.54E-11 |
| rs9886665 | 0.2048 | 0.0343 | T | C | 0.7329 | 2.47E-09 |
| rs4553000 | -0.2035 | 0.03 | T | C | 0.4859 | 1.09E-11 |
| rs76452347 | -0.2974 | 0.0397 | T | C | 0.795 | 7.13E-14 |
| rs927315 | 0.1689 | 0.0303 | T | C | 0.5287 | 2.44E-08 |
| rs60191654 | -0.2382 | 0.0385 | A | G | 0.1882 | 5.88E-10 |
| rs10746963 | -0.2177 | 0.0388 | A | G | 0.8166 | 2.05E-08 |
| rs1332813 | 0.2203 | 0.0314 | T | C | 0.6486 | 2.32E-12 |
| rs7045409 | -0.1862 | 0.0313 | A | T | 0.6331 | 2.55E-09 |

**Table S16. Genome-wide significant and independent SNPs that were used as instruments for DBP**

| SNP | beta. | se. | effect_ | other_ | eaf.exposure | pval.exposure |
| --- | --- | --- | --- | --- | --- | --- |
| rs1006545 | 0.3633 | 0.0275 | T | G | 0.1125 | 7.96E-40 |
| rs2273654 | 0.1165 | 0.0175 | T | C | 0.4392 | 2.75E-11 |
| rs12414028 | -0.5141 | 0.0313 | A | T | 0.9119 | 1.60E-60 |
| rs2067831 | -0.128 | 0.0195 | C | G | 0.7277 | 5.08E-11 |
| rs2484294 | 0.3165 | 0.0196 | A | G | 0.2673 | 1.17E-58 |
| rs72842207 | -0.2112 | 0.0211 | T | C | 0.7851 | 1.10E-23 |
| rs11592107 | 0.1203 | 0.0187 | A | G | 0.6906 | 1.23E-10 |
| rs10490923 | 0.1533 | 0.0262 | A | G | 0.8743 | 5.02E-09 |
| rs9419374 | 0.1164 | 0.0185 | A | G | 0.646 | 3.44E-10 |
| rs1133400 | -0.1318 | 0.0215 | A | G | 0.2148 | 8.30E-10 |
| rs6602177 | -0.1203 | 0.0207 | T | C | 0.2927 | 6.52E-09 |
| rs1623474 | 0.2234 | 0.0184 | T | C | 0.67 | 6.24E-34 |
| rs12258967 | 0.354 | 0.0193 | C | G | 0.2958 | 3.27E-75 |
| rs3802517 | 0.1286 | 0.0173 | A | T | 0.5385 | 9.29E-14 |
| rs1265842 | 0.1113 | 0.0174 | T | C | 0.5166 | 1.70E-10 |
| rs2487926 | 0.0972 | 0.0176 | A | G | 0.4295 | 3.31E-08 |
| rs3006583 | -0.1303 | 0.0222 | T | C | 0.1886 | 4.66E-09 |
| rs11252324 | -0.2339 | 0.0328 | T | G | 0.923 | 1.03E-12 |
| rs4948643 | 0.1591 | 0.0194 | T | C | 0.718 | 2.26E-16 |
| rs34130368 | -0.2027 | 0.0284 | T | G | 0.8828 | 8.77E-13 |
| rs72831343 | 0.4936 | 0.0248 | T | G | 0.1419 | 4.77E-88 |
| rs2236295 | -0.207 | 0.0177 | T | G | 0.6008 | 1.42E-31 |
| rs35506078 | -0.1348 | 0.0183 | T | C | 0.3366 | 1.54E-13 |
| rs12247028 | -0.1396 | 0.0188 | A | G | 0.3678 | 1.18E-13 |
| rs2274224 | -0.2788 | 0.0174 | C | G | 0.5675 | 1.24E-57 |
| rs604723 | -0.3848 | 0.0194 | T | C | 0.7247 | 2.32E-87 |
| rs66682451 | 0.1348 | 0.0194 | A | G | 0.2747 | 3.44E-12 |
| rs7106104 | -0.1186 | 0.0193 | T | C | 0.281 | 7.72E-10 |
| rs12790943 | -0.1002 | 0.0175 | T | C | 0.5787 | 1.14E-08 |
| rs12574332 | 0.2072 | 0.0266 | T | C | 0.8773 | 6.14E-15 |
| rs4936099 | 0.1745 | 0.0178 | A | C | 0.4011 | 1.16E-22 |
| rs7107711 | 0.1263 | 0.021 | C | G | 0.2218 | 1.67E-09 |
| rs4756782 | 0.1551 | 0.0234 | A | C | 0.835 | 3.52E-11 |
| rs28570096 | 0.1396 | 0.0188 | T | C | 0.6906 | 1.15E-13 |
| rs10832586 | -0.3083 | 0.0216 | A | C | 0.2016 | 2.53E-46 |
| rs7926335 | 0.1804 | 0.0195 | T | C | 0.7301 | 2.05E-20 |
| rs79889784 | -0.3941 | 0.0717 | T | G | 0.9824 | 3.86E-08 |
| rs569550 | -0.2688 | 0.0181 | T | G | 0.3954 | 1.23E-49 |
| rs147081004 | 0.1411 | 0.0257 | A | C | 0.1444 | 4.09E-08 |
| rs10500932 | 0.2784 | 0.0333 | A | G | 0.9257 | 5.79E-17 |
| rs962369 | 0.1684 | 0.0189 | T | C | 0.3013 | 6.02E-19 |
| rs7933758 | -0.1138 | 0.0191 | T | C | 0.6953 | 2.58E-09 |
| rs10838702 | 0.2375 | 0.0178 | T | G | 0.6125 | 1.27E-40 |
| rs11040503 | -0.17 | 0.0236 | A | C | 0.8143 | 5.65E-13 |
| rs751984 | 0.3937 | 0.0275 | T | C | 0.1174 | 1.38E-46 |
| rs35927325 | 0.2221 | 0.0364 | T | C | 0.9386 | 1.01E-09 |
| rs2306363 | -0.2643 | 0.0216 | T | G | 0.7952 | 1.63E-34 |
| rs11228613 | 0.1741 | 0.0212 | T | G | 0.2163 | 2.10E-16 |
| rs504217 | 0.2745 | 0.0335 | T | C | 0.9264 | 2.51E-16 |
| rs7115331 | -0.1266 | 0.0192 | T | G | 0.2857 | 3.92E-11 |
| rs11021221 | -0.1877 | 0.0233 | A | T | 0.8332 | 6.93E-16 |
| rs61909958 | 0.1275 | 0.0228 | C | G | 0.1881 | 2.21E-08 |
| rs360153 | -0.2198 | 0.0175 | T | C | 0.5828 | 4.37E-36 |
| rs11112548 | 0.2742 | 0.0443 | A | T | 0.0444 | 5.80E-10 |
| rs116063464 | 0.2017 | 0.0369 | A | G | 0.9399 | 4.68E-08 |
| rs7137828 | -0.5027 | 0.0176 | T | C | 0.4817 | ####### |
| rs35443 | -0.2661 | 0.0178 | C | G | 0.6142 | 1.20E-50 |
| rs6490019 | -0.1778 | 0.0178 | A | G | 0.6203 | 2.10E-23 |
| rs1790123 | 0.1991 | 0.0218 | T | C | 0.1968 | 6.87E-20 |
| rs2271139 | -0.1247 | 0.0192 | A | C | 0.714 | 8.23E-11 |
| rs61912333 | 0.1191 | 0.0176 | C | G | 0.5037 | 1.13E-11 |
| rs4306343 | -0.317 | 0.0193 | A | T | 0.7212 | 8.22E-61 |
| rs6487076 | 0.174 | 0.0209 | A | G | 0.223 | 8.69E-17 |
| rs55935819 | 0.1271 | 0.0181 | A | G | 0.6364 | 1.96E-12 |
| rs12229480 | 0.1359 | 0.0193 | T | C | 0.2775 | 2.11E-12 |
| rs1669907 | 0.1158 | 0.0191 | T | G | 0.6968 | 1.36E-09 |
| rs61917655 | 0.2246 | 0.0297 | T | C | 0.8989 | 3.72E-14 |
| rs7967705 | 0.2694 | 0.0178 | T | C | 0.6196 | 1.54E-51 |
| rs7306947 | -0.205 | 0.0342 | T | G | 0.072 | 2.14E-09 |
| rs6580970 | -0.1661 | 0.0191 | T | C | 0.7013 | 4.03E-18 |
| rs6581101 | -0.1261 | 0.0179 | A | C | 0.3962 | 2.05E-12 |
| rs7959649 | 0.1166 | 0.0202 | T | C | 0.7576 | 8.14E-09 |
| rs521033 | -0.1802 | 0.0253 | A | G | 0.1364 | 1.10E-12 |
| rs710698 | 0.1059 | 0.0176 | A | G | 0.4135 | 1.89E-09 |
| rs2681485 | 0.2945 | 0.0176 | A | G | 0.4024 | 1.31E-62 |
| rs11108209 | -0.1901 | 0.03 | T | C | 0.0932 | 2.40E-10 |
| rs675605 | -0.1205 | 0.0196 | C | G | 0.2841 | 8.46E-10 |
| rs7990017 | 0.1039 | 0.0185 | T | C | 0.5267 | 1.92E-08 |
| rs7491960 | -0.1288 | 0.018 | T | C | 0.508 | 8.44E-13 |
| rs7321688 | 0.1507 | 0.0205 | A | C | 0.7675 | 1.99E-13 |
| rs682681 | -0.1454 | 0.0185 | T | C | 0.6665 | 4.47E-15 |
| rs61948065 | -0.1737 | 0.027 | A | C | 0.1212 | 1.17E-10 |
| rs9508495 | -0.1944 | 0.0204 | T | C | 0.2431 | 1.34E-21 |
| rs56256111 | 0.1926 | 0.0263 | A | G | 0.8558 | 2.60E-13 |
| rs7992292 | 0.1367 | 0.0231 | A | G | 0.176 | 3.19E-09 |
| rs9526707 | -0.1217 | 0.0186 | A | G | 0.6778 | 6.59E-11 |
| rs9563529 | 0.1222 | 0.0215 | T | G | 0.7957 | 1.38E-08 |
| rs3861113 | 0.2126 | 0.0322 | A | C | 0.9175 | 3.95E-11 |
| rs12866098 | 0.1033 | 0.0186 | A | G | 0.6577 | 2.73E-08 |
| rs1215469 | -0.1383 | 0.0211 | A | C | 0.7705 | 5.23E-11 |
| rs55684003 | 0.122 | 0.0189 | A | G | 0.3041 | 1.01E-10 |
| rs8014182 | -0.1942 | 0.0257 | T | C | 0.8681 | 3.94E-14 |
| rs7350752 | -0.1504 | 0.0268 | A | G | 0.8759 | 1.97E-08 |
| rs17880989 | 0.4014 | 0.0591 | A | G | 0.9741 | 1.11E-11 |
| rs1950500 | 0.1396 | 0.019 | T | C | 0.7081 | 2.20E-13 |
| rs4424827 | -0.0981 | 0.0175 | T | C | 0.4331 | 2.11E-08 |
| rs7155504 | 0.2286 | 0.0317 | T | C | 0.0876 | 5.16E-13 |
| rs72683923 | 0.5325 | 0.0635 | T | C | 0.0212 | 5.02E-17 |
| rs35413927 | -0.1274 | 0.0189 | A | G | 0.3049 | 1.77E-11 |
| rs12148044 | 0.1371 | 0.023 | A | G | 0.8266 | 2.66E-09 |
| rs227426 | 0.1119 | 0.0175 | T | G | 0.4381 | 1.75E-10 |
| rs2239268 | 0.1097 | 0.019 | A | G | 0.2995 | 7.40E-09 |
| rs4903064 | 0.1543 | 0.0206 | T | C | 0.2355 | 7.84E-14 |
| rs10873612 | -0.1096 | 0.0179 | T | C | 0.4039 | 9.51E-10 |
| rs11070245 | -0.1287 | 0.0174 | T | G | 0.5321 | 1.57E-13 |
| rs2925345 | 0.189 | 0.0174 | T | C | 0.5324 | 1.60E-27 |
| rs17678552 | -0.1649 | 0.0182 | T | C | 0.3439 | 1.33E-19 |
| rs7169864 | -0.1132 | 0.0205 | T | C | 0.7678 | 3.40E-08 |
| rs28429256 | 0.1636 | 0.0188 | A | G | 0.6656 | 2.83E-18 |
| rs2469141 | 0.1351 | 0.0238 | T | C | 0.1628 | 1.39E-08 |
| rs3743111 | 0.1517 | 0.0178 | A | G | 0.387 | 1.62E-17 |
| rs11636952 | 0.3997 | 0.0189 | T | C | 0.6869 | 5.21E-99 |
| rs57708073 | 0.1907 | 0.0214 | A | G | 0.2608 | 4.73E-19 |
| rs2627313 | 0.151 | 0.0175 | T | C | 0.5543 | 5.86E-18 |
| rs77032376 | -0.173 | 0.0249 | T | C | 0.8521 | 3.64E-12 |
| rs4932373 | -0.3664 | 0.0189 | A | C | 0.3257 | 7.71E-84 |
| rs3743369 | 0.104 | 0.0179 | A | G | 0.3722 | 6.82E-09 |
| rs12906962 | -0.2378 | 0.0188 | T | C | 0.3233 | 8.73E-37 |
| rs2589218 | -0.1207 | 0.0196 | T | C | 0.2698 | 6.90E-10 |
| rs77924615 | -0.3163 | 0.0224 | A | G | 0.8018 | 3.72E-45 |
| rs12596630 | 0.2606 | 0.0314 | T | C | 0.9095 | 1.03E-16 |
| rs9937801 | 0.1554 | 0.0174 | T | C | 0.4308 | 4.81E-19 |
| rs80095680 | -0.1566 | 0.0198 | A | G | 0.2633 | 2.81E-15 |
| rs917522 | 0.1665 | 0.0273 | T | C | 0.115 | 1.04E-09 |
| rs12446456 | -0.181 | 0.0175 | T | C | 0.5727 | 3.99E-25 |
| rs7192407 | 0.1019 | 0.0174 | T | C | 0.528 | 4.53E-09 |
| rs62030049 | 0.1336 | 0.0209 | A | G | 0.2404 | 1.55E-10 |
| rs9932220 | -0.1591 | 0.021 | A | G | 0.7823 | 3.76E-14 |
| rs12919839 | -0.1098 | 0.0192 | T | C | 0.7159 | 1.04E-08 |
| rs45474499 | 0.3562 | 0.0415 | T | C | 0.9527 | 8.50E-18 |
| rs28544928 | 0.1543 | 0.0199 | T | G | 0.2535 | 9.13E-15 |
| rs12444212 | 0.1291 | 0.0226 | T | C | 0.1826 | 1.06E-08 |
| rs11859505 | -0.1037 | 0.0181 | A | G | 0.5805 | 9.76E-09 |
| rs8046697 | -0.1289 | 0.0179 | T | C | 0.5833 | 6.10E-13 |
| rs12929303 | 0.1572 | 0.0174 | A | G | 0.4675 | 1.58E-19 |
| rs79286081 | -0.1631 | 0.0299 | A | G | 0.8979 | 4.83E-08 |
| rs908951 | -0.1983 | 0.0181 | T | C | 0.563 | 7.73E-28 |
| rs9893005 | -0.1205 | 0.0176 | C | G | 0.4647 | 7.90E-12 |
| rs1043809 | 0.159 | 0.0223 | T | C | 0.1917 | 9.77E-13 |
| rs4362428 | -0.1127 | 0.0176 | A | C | 0.5913 | 1.45E-10 |
| rs76954792 | 0.1213 | 0.0208 | T | C | 0.7678 | 5.06E-09 |
| rs28661492 | -0.1359 | 0.0222 | T | C | 0.7978 | 9.56E-10 |
| rs2239917 | 0.1731 | 0.0176 | T | C | 0.5748 | 9.69E-23 |
| rs62064603 | -0.1335 | 0.0229 | T | C | 0.8144 | 5.52E-09 |
| rs55938136 | 0.1408 | 0.0225 | A | G | 0.2249 | 4.21E-10 |
| rs3916033 | -0.1233 | 0.0185 | T | C | 0.4435 | 2.42E-11 |
| rs8081301 | 0.1278 | 0.0197 | A | T | 0.2705 | 8.67E-11 |
| rs9889262 | 0.2283 | 0.018 | A | T | 0.6334 | 7.11E-37 |
| rs3785837 | 0.1453 | 0.0213 | A | G | 0.2365 | 9.57E-12 |
| rs6504163 | -0.1842 | 0.0183 | T | C | 0.3763 | 6.30E-24 |
| rs1867624 | 0.1412 | 0.0178 | T | C | 0.3853 | 2.08E-15 |
| rs12601936 | -0.1429 | 0.0178 | A | G | 0.6107 | 1.07E-15 |
| rs1436138 | 0.1991 | 0.0182 | A | G | 0.3633 | 7.33E-28 |
| rs7217916 | 0.1111 | 0.0179 | A | G | 0.6146 | 5.63E-10 |
| rs138420351 | 0.5568 | 0.0854 | T | C | 0.984 | 7.11E-11 |
| rs74439044 | -0.3496 | 0.0294 | T | C | 0.0983 | 1.38E-32 |
| rs11077961 | 0.1073 | 0.0186 | A | G | 0.3676 | 8.55E-09 |
| rs11665020 | -0.1423 | 0.0187 | C | G | 0.678 | 2.78E-14 |
| rs11664194 | -0.1078 | 0.0176 | A | T | 0.5397 | 8.69E-10 |
| rs10164193 | -0.2196 | 0.0327 | T | G | 0.0777 | 1.87E-11 |
| rs11661473 | 0.2007 | 0.0196 | A | G | 0.7317 | 1.54E-24 |
| rs58693787 | 0.1584 | 0.0202 | A | G | 0.2458 | 3.82E-15 |
| rs4102481 | -0.1248 | 0.019 | T | G | 0.3049 | 4.87E-11 |
| rs2062011 | -0.1138 | 0.0209 | A | T | 0.7509 | 4.80E-08 |
| rs1903752 | -0.0987 | 0.0178 | T | C | 0.4614 | 3.20E-08 |
| rs4891258 | -0.1159 | 0.0187 | A | G | 0.3174 | 5.72E-10 |
| rs7227492 | 0.181 | 0.0227 | T | C | 0.1822 | 1.43E-15 |
| rs387865 | -0.1059 | 0.0191 | T | C | 0.6938 | 3.17E-08 |
| rs167479 | -0.362 | 0.0188 | T | G | 0.5278 | 1.67E-82 |
| rs1077795 | 0.1987 | 0.0199 | A | G | 0.2611 | 1.62E-23 |
| rs72999033 | 0.2793 | 0.0358 | T | C | 0.9342 | 5.95E-15 |
| rs7257694 | 0.1837 | 0.0178 | T | C | 0.5997 | 6.28E-25 |
| rs8108717 | 0.1323 | 0.0179 | A | G | 0.6084 | 1.39E-13 |
| rs73036520 | 0.1557 | 0.0202 | C | G | 0.7457 | 1.34E-14 |
| rs2548459 | -0.132 | 0.0176 | T | C | 0.5195 | 5.95E-14 |
| rs73046792 | -0.1518 | 0.0245 | A | G | 0.8408 | 5.87E-10 |
| rs10424224 | 0.1042 | 0.0182 | T | C | 0.6416 | 1.05E-08 |
| rs7258382 | 0.2624 | 0.0248 | T | C | 0.1611 | 3.03E-26 |
| rs2009733 | 0.1217 | 0.0176 | A | G | 0.5005 | 5.10E-12 |
| rs488834 | -0.1931 | 0.0208 | T | C | 0.2359 | 1.94E-20 |
| rs10776752 | 0.4573 | 0.033 | T | G | 0.9195 | 1.25E-43 |
| rs57748895 | -0.6627 | 0.0666 | A | T | 0.0179 | 2.49E-23 |
| rs55857306 | -0.5224 | 0.0235 | A | G | 0.8398 | ####### |
| rs72704264 | 0.117 | 0.0212 | C | G | 0.7828 | 3.60E-08 |
| rs1819663 | 0.1147 | 0.0174 | A | G | 0.4929 | 4.63E-11 |
| rs76719272 | -0.1438 | 0.0264 | T | C | 0.8685 | 4.86E-08 |
| rs1889785 | 0.1255 | 0.0174 | A | G | 0.5449 | 5.61E-13 |
| rs7524019 | 0.1036 | 0.0174 | T | C | 0.508 | 2.60E-09 |
| rs12405515 | -0.1698 | 0.0174 | T | G | 0.4298 | 1.92E-22 |
| rs34645159 | -0.133 | 0.0174 | A | G | 0.4987 | 2.07E-14 |
| rs150816167 | -0.2873 | 0.0446 | T | C | 0.0451 | 1.17E-10 |
| rs4651224 | 0.1102 | 0.0175 | T | C | 0.5531 | 3.39E-10 |
| rs882624 | -0.1571 | 0.0185 | T | C | 0.6675 | 2.33E-17 |
| rs2169137 | 0.1588 | 0.0194 | C | G | 0.2713 | 3.17E-16 |
| rs1502358 | -0.1127 | 0.0185 | A | G | 0.3187 | 1.13E-09 |
| rs68085857 | 0.191 | 0.0205 | T | C | 0.766 | 9.83E-21 |
| rs12088448 | -0.1544 | 0.0182 | A | C | 0.356 | 2.53E-17 |
| rs602521 | 0.1351 | 0.0195 | A | G | 0.7344 | 3.97E-12 |
| rs1745417 | 0.1708 | 0.0173 | T | C | 0.4807 | 4.69E-23 |
| rs699 | -0.2359 | 0.0177 | A | G | 0.407 | 1.30E-40 |
| rs3943093 | 0.2477 | 0.0184 | T | C | 0.6766 | 3.95E-41 |
| rs4926499 | 0.1694 | 0.0248 | C | G | 0.174 | 9.37E-12 |
| rs6686889 | 0.1918 | 0.0199 | T | C | 0.7467 | 6.95E-22 |
| rs12728150 | -0.2045 | 0.0318 | A | G | 0.081 | 1.28E-10 |
| rs2493296 | 0.2496 | 0.0254 | T | C | 0.8581 | 7.45E-23 |
| rs2146315 | -0.1197 | 0.0205 | T | C | 0.7682 | 5.03E-09 |
| rs710249 | 0.1501 | 0.0174 | C | G | 0.5736 | 6.43E-18 |
| rs4926901 | 0.0984 | 0.018 | A | G | 0.6452 | 4.82E-08 |
| rs4926923 | 0.1918 | 0.0308 | T | C | 0.0883 | 4.75E-10 |
| rs61772592 | -0.1509 | 0.0261 | A | G | 0.1257 | 7.42E-09 |
| rs10493408 | 0.1584 | 0.0255 | A | C | 0.8669 | 5.09E-10 |
| rs34517439 | -0.2514 | 0.0279 | A | C | 0.8801 | 2.02E-19 |
| rs786921 | -0.1145 | 0.0176 | A | G | 0.4043 | 8.63E-11 |
| rs17396055 | -0.115 | 0.0184 | A | G | 0.6676 | 4.13E-10 |
| rs693974 | -0.1847 | 0.0177 | T | C | 0.3964 | 1.76E-25 |
| rs1327235 | -0.3018 | 0.0173 | A | G | 0.4714 | 4.76E-68 |
| rs6078393 | 0.1205 | 0.0176 | T | G | 0.4106 | 7.66E-12 |
| rs4814837 | -0.1003 | 0.0184 | T | C | 0.6576 | 4.62E-08 |
| rs2376997 | -0.1389 | 0.0218 | A | C | 0.7511 | 1.94E-10 |
| rs13042148 | -0.1674 | 0.0244 | T | C | 0.8463 | 7.24E-12 |
| rs7265695 | 0.1967 | 0.0219 | T | C | 0.1965 | 2.48E-19 |
| rs6031431 | -0.1153 | 0.0175 | A | G | 0.4622 | 4.94E-11 |
| rs2598 | 0.1387 | 0.0175 | A | G | 0.4674 | 1.94E-15 |
| rs79044887 | 0.2427 | 0.0245 | C | G | 0.1476 | 4.01E-23 |
| rs234623 | -0.1191 | 0.0174 | A | G | 0.4959 | 8.56E-12 |
| rs6026739 | -0.5032 | 0.0266 | A | T | 0.1226 | 1.49E-79 |
| rs79208229 | 0.2128 | 0.0326 | T | G | 0.9125 | 6.53E-11 |
| rs35213536 | 0.2044 | 0.0205 | T | G | 0.7533 | 2.54E-23 |
| rs6108168 | -0.1901 | 0.0199 | A | C | 0.7454 | 1.10E-21 |
| rs1882961 | 0.1272 | 0.0188 | T | C | 0.6912 | 1.40E-11 |
| rs1003763 | -0.1484 | 0.0203 | C | G | 0.753 | 2.75E-13 |
| rs12627514 | -0.2164 | 0.0196 | C | G | 0.2897 | 1.99E-28 |
| rs34487963 | -0.5734 | 0.0712 | A | C | 0.9815 | 8.18E-16 |
| rs7278003 | -0.1293 | 0.0176 | T | C | 0.5615 | 1.78E-13 |
| rs5992929 | 0.1684 | 0.0193 | T | C | 0.7166 | 3.07E-18 |
| rs134041 | 0.1223 | 0.0175 | T | C | 0.564 | 3.05E-12 |
| rs12321 | -0.1492 | 0.0175 | C | G | 0.5667 | 1.44E-17 |
| rs5753630 | 0.107 | 0.0175 | A | G | 0.4382 | 8.76E-10 |
| rs28377357 | -0.1243 | 0.019 | A | G | 0.7062 | 6.03E-11 |
| rs62158170 | 0.1645 | 0.0211 | A | G | 0.2166 | 6.63E-15 |
| rs13001283 | 0.1522 | 0.0239 | A | G | 0.8404 | 1.92E-10 |
| rs4954192 | -0.1225 | 0.0179 | T | C | 0.6128 | 8.15E-12 |
| rs55944332 | -0.2365 | 0.0204 | A | G | 0.2368 | 3.27E-31 |
| rs12990959 | -0.1271 | 0.0187 | T | C | 0.3125 | 1.11E-11 |
| rs2444769 | 0.158 | 0.0219 | A | C | 0.2051 | 4.85E-13 |
| rs7572130 | -0.1796 | 0.0287 | A | G | 0.1042 | 4.12E-10 |
| rs73029563 | -0.2592 | 0.0174 | C | G | 0.545 | 5.77E-50 |
| rs75717699 | -0.4667 | 0.0541 | T | G | 0.0305 | 6.71E-18 |
| rs1518460 | 0.1342 | 0.0189 | A | G | 0.2918 | 1.26E-12 |
| rs12693302 | -0.2378 | 0.0181 | A | G | 0.3482 | 2.16E-39 |
| rs7592578 | -0.1998 | 0.0224 | T | G | 0.8062 | 4.71E-19 |
| rs1373780 | 0.1246 | 0.0224 | C | G | 0.8155 | 2.58E-08 |
| rs824523 | 0.1226 | 0.0183 | A | C | 0.6656 | 2.26E-11 |
| rs4673253 | -0.1177 | 0.0197 | C | G | 0.2655 | 2.44E-09 |
| rs11692619 | -0.1281 | 0.0184 | T | C | 0.6393 | 3.31E-12 |
| rs1263671 | -0.1394 | 0.0238 | T | C | 0.1632 | 4.69E-09 |
| rs4675682 | -0.1409 | 0.0173 | T | C | 0.4622 | 4.49E-16 |
| rs1035673 | 0.1625 | 0.0176 | T | C | 0.6032 | 3.00E-20 |
| rs13004222 | 0.2944 | 0.0393 | C | G | 0.051 | 7.11E-14 |
| rs1039897 | -0.1085 | 0.0183 | A | G | 0.3497 | 3.26E-09 |
| rs10804330 | 0.1331 | 0.0176 | T | C | 0.4329 | 4.60E-14 |
| rs1044822 | -0.1334 | 0.0243 | T | C | 0.8512 | 4.14E-08 |
| rs4507125 | -0.1244 | 0.0211 | A | C | 0.2136 | 3.60E-09 |
| rs11687089 | 0.1739 | 0.0175 | T | C | 0.4171 | 2.79E-23 |
| rs1275988 | -0.2945 | 0.0177 | T | C | 0.3889 | 1.92E-62 |
| rs11684340 | 0.1249 | 0.021 | A | C | 0.2176 | 2.75E-09 |
| rs2160236 | -0.1421 | 0.0181 | C | G | 0.6208 | 4.31E-15 |
| rs76326501 | 0.3618 | 0.0305 | A | C | 0.0911 | 2.17E-32 |
| rs4952668 | -0.192 | 0.018 | A | G | 0.3763 | 1.13E-26 |
| rs2586970 | -0.1493 | 0.0175 | A | G | 0.5639 | 1.56E-17 |
| rs2421200 | -0.1097 | 0.0173 | T | G | 0.5118 | 2.59E-10 |
| rs1876490 | 0.1364 | 0.0192 | A | G | 0.2833 | 1.16E-12 |
| rs6546810 | -0.12 | 0.0181 | T | C | 0.3525 | 3.16E-11 |
| rs311564 | -0.133 | 0.0183 | A | G | 0.6539 | 4.23E-13 |
| rs62155750 | -0.2177 | 0.0196 | A | G | 0.3074 | 8.27E-29 |
| rs112393817 | 0.116 | 0.0211 | C | G | 0.2174 | 3.80E-08 |
| rs11923667 | 0.1175 | 0.0177 | A | T | 0.5929 | 3.10E-11 |
| rs28675079 | -0.1444 | 0.0222 | A | G | 0.8133 | 8.34E-11 |
| rs347585 | 0.1506 | 0.0189 | T | C | 0.2986 | 1.57E-15 |
| rs12152463 | 0.1006 | 0.0174 | T | C | 0.5749 | 8.02E-09 |
| rs4141663 | -0.1496 | 0.0175 | T | C | 0.5784 | 1.41E-17 |
| rs4077158 | -0.1832 | 0.0173 | T | C | 0.5286 | 3.09E-26 |
| rs9289557 | -0.119 | 0.0207 | T | C | 0.7396 | 8.68E-09 |
| rs6763931 | 0.1383 | 0.0173 | A | G | 0.5562 | 1.48E-15 |
| rs1687295 | 0.2061 | 0.0194 | T | C | 0.7296 | 2.99E-26 |
| rs36117336 | -0.147 | 0.0198 | T | C | 0.2562 | 1.10E-13 |
| rs78809139 | -0.2281 | 0.0288 | A | G | 0.8986 | 2.58E-15 |
| rs78151625 | -0.1869 | 0.0233 | T | C | 0.1658 | 1.04E-15 |
| rs62234672 | 0.1248 | 0.0229 | A | C | 0.8248 | 4.92E-08 |
| rs16853198 | 0.3386 | 0.0327 | A | G | 0.0762 | 4.44E-25 |
| rs1528293 | 0.2764 | 0.0173 | A | T | 0.5079 | 1.48E-57 |
| rs62294352 | -0.1605 | 0.0223 | T | C | 0.7843 | 6.07E-13 |
| rs6779368 | -0.1791 | 0.0184 | A | G | 0.3423 | 2.28E-22 |
| rs147501096 | -0.1955 | 0.0341 | C | G | 0.928 | 9.94E-09 |
| rs4244200 | -0.1215 | 0.0193 | C | G | 0.7201 | 3.23E-10 |
| rs6777317 | 0.1249 | 0.0195 | A | G | 0.7101 | 1.51E-10 |
| rs2643826 | 0.1857 | 0.0175 | T | C | 0.5492 | 2.83E-26 |
| rs7427249 | -0.1098 | 0.0176 | A | G | 0.42 | 4.34E-10 |
| rs3864004 | 0.1004 | 0.0173 | A | G | 0.5315 | 6.28E-09 |
| rs114714860 | 0.33 | 0.0236 | C | G | 0.8317 | 1.42E-44 |
| rs6442105 | -0.2485 | 0.0185 | A | G | 0.6726 | 3.10E-41 |
| rs6445590 | 0.1284 | 0.0174 | A | G | 0.5455 | 1.65E-13 |
| rs3772219 | 0.1754 | 0.0185 | A | C | 0.3193 | 2.94E-21 |
| rs1675383 | 0.1488 | 0.0174 | A | C | 0.5569 | 1.47E-17 |
| rs3774702 | 0.147 | 0.0228 | A | G | 0.8232 | 1.18E-10 |
| rs6795735 | -0.1438 | 0.0176 | T | C | 0.5891 | 3.05E-16 |
| rs7623706 | 0.0975 | 0.0176 | A | G | 0.4349 | 2.84E-08 |
| rs11923343 | -0.1138 | 0.0181 | A | G | 0.6396 | 3.10E-10 |
| rs13107325 | -0.6747 | 0.0339 | T | C | 0.9258 | 3.72E-88 |
| rs12503341 | -0.2993 | 0.0462 | A | G | 0.9606 | 9.43E-11 |
| rs4245929 | 0.12 | 0.0181 | A | T | 0.6352 | 3.59E-11 |
| rs13118687 | -0.1496 | 0.0175 | A | G | 0.5298 | 1.37E-17 |
| rs66887589 | -0.161 | 0.0174 | T | C | 0.4779 | 1.83E-20 |
| rs9286351 | -0.1412 | 0.0177 | A | G | 0.4188 | 1.61E-15 |
| rs72719149 | -0.1279 | 0.0186 | T | C | 0.3164 | 6.34E-12 |
| rs13124515 | -0.1052 | 0.0187 | T | C | 0.6869 | 1.98E-08 |
| rs1123037 | -0.1608 | 0.0173 | A | T | 0.5231 | 1.58E-20 |
| rs13139571 | -0.2408 | 0.0203 | A | C | 0.7634 | 2.29E-32 |
| rs1425486 | -0.1331 | 0.0187 | T | C | 0.6793 | 1.11E-12 |
| rs16896276 | -0.1309 | 0.0198 | A | T | 0.7375 | 3.82E-11 |
| rs61789369 | -0.3039 | 0.0436 | A | G | 0.0435 | 3.07E-12 |
| rs28667801 | -0.1622 | 0.018 | A | T | 0.407 | 1.90E-19 |
| rs11721984 | -0.1409 | 0.0177 | T | C | 0.5468 | 1.89E-15 |
| rs62301873 | -0.1734 | 0.0284 | A | G | 0.1061 | 1.06E-09 |
| rs11945489 | -0.1392 | 0.0192 | T | C | 0.7091 | 3.99E-13 |
| rs13152154 | -0.1186 | 0.0195 | T | C | 0.2707 | 1.23E-09 |
| rs12509595 | -0.4972 | 0.0192 | T | C | 0.2924 | ####### |
| rs72976750 | -0.1718 | 0.0251 | T | C | 0.1396 | 7.37E-12 |
| rs7694000 | -0.0965 | 0.0175 | A | T | 0.4613 | 3.47E-08 |
| rs9326869 | 0.1096 | 0.02 | T | C | 0.7513 | 3.99E-08 |
| rs369625 | -0.1053 | 0.0178 | C | G | 0.5957 | 3.57E-09 |
| rs1582931 | 0.2161 | 0.0175 | A | G | 0.5252 | 4.51E-35 |
| rs17677603 | -0.2 | 0.0178 | A | G | 0.3837 | 3.90E-29 |
| rs10069690 | 0.1615 | 0.021 | T | C | 0.7419 | 1.42E-14 |
| rs55747751 | -0.2239 | 0.0331 | A | G | 0.919 | 1.39E-11 |
| rs4912840 | -0.1485 | 0.0245 | A | G | 0.8453 | 1.25E-09 |
| rs3776299 | 0.1266 | 0.0175 | A | G | 0.5441 | 5.06E-13 |
| rs78909293 | 0.321 | 0.0429 | T | C | 0.0449 | 7.31E-14 |
| rs2921604 | -0.096 | 0.0176 | T | C | 0.4633 | 4.46E-08 |
| rs3117736 | 0.2374 | 0.0196 | T | C | 0.7339 | 9.71E-34 |
| rs11960210 | 0.2474 | 0.018 | T | C | 0.3751 | 3.36E-43 |
| rs13358657 | -0.224 | 0.0255 | A | G | 0.1332 | 1.70E-18 |
| rs6556384 | -0.152 | 0.0221 | A | C | 0.1895 | 5.91E-12 |
| rs114503346 | -0.2678 | 0.0426 | T | C | 0.9539 | 3.10E-10 |
| rs55993676 | -0.2097 | 0.0191 | T | G | 0.7084 | 3.82E-28 |
| rs1177764 | -0.3067 | 0.0177 | C | G | 0.5949 | 1.42E-67 |
| rs10941043 | -0.1269 | 0.019 | T | G | 0.2906 | 2.52E-11 |
| rs4645335 | 0.1142 | 0.0185 | A | G | 0.664 | 7.04E-10 |
| rs7737851 | -0.1256 | 0.022 | T | C | 0.8056 | 1.11E-08 |
| rs6875967 | 0.1344 | 0.0181 | A | G | 0.6479 | 1.21E-13 |
| rs10054208 | 0.1187 | 0.0185 | T | C | 0.6383 | 1.49E-10 |
| rs12515541 | 0.1156 | 0.0177 | T | G | 0.3928 | 6.23E-11 |
| rs1848510 | 0.1256 | 0.0181 | A | G | 0.6377 | 4.10E-12 |
| rs10062049 | 0.2208 | 0.0255 | T | C | 0.8641 | 4.50E-18 |
| rs2307111 | -0.1742 | 0.0178 | T | C | 0.3966 | 1.62E-22 |
| rs4704514 | 0.1087 | 0.0193 | T | C | 0.7167 | 1.71E-08 |
| rs62380354 | 0.1825 | 0.0291 | A | C | 0.1096 | 3.68E-10 |
| rs13355146 | 0.1224 | 0.0178 | T | C | 0.6168 | 6.39E-12 |
| rs55770741 | -0.1281 | 0.0175 | T | C | 0.4387 | 2.20E-13 |
| rs1871190 | 0.1078 | 0.0186 | T | G | 0.6656 | 6.63E-09 |
| rs72613227 | -0.1884 | 0.0285 | A | T | 0.1269 | 3.87E-11 |
| rs7767235 | -0.1181 | 0.0182 | A | C | 0.6468 | 7.95E-11 |
| rs509067 | -0.1436 | 0.0175 | T | C | 0.5863 | 2.65E-16 |
| rs11153730 | 0.1551 | 0.0173 | T | C | 0.4906 | 2.57E-19 |
| rs76785130 | -0.4285 | 0.0662 | A | G | 0.0199 | 9.36E-11 |
| rs13215166 | -0.3094 | 0.0174 | A | G | 0.4415 | 1.79E-70 |
| rs9399137 | 0.1148 | 0.0197 | T | C | 0.2619 | 5.83E-09 |
| rs636202 | 0.1023 | 0.0174 | T | C | 0.5185 | 4.40E-09 |
| rs9791312 | -0.1225 | 0.0184 | A | C | 0.3452 | 2.89E-11 |
| rs62434124 | -0.4853 | 0.0338 | T | C | 0.9289 | 7.83E-47 |
| rs9478282 | -0.1994 | 0.0279 | T | C | 0.8884 | 8.70E-13 |
| rs2569882 | 0.1199 | 0.0182 | T | C | 0.4342 | 4.28E-11 |
| rs9365555 | 0.1254 | 0.0187 | A | G | 0.3259 | 1.96E-11 |
| rs11961593 | -0.3158 | 0.0349 | T | C | 0.9315 | 1.49E-19 |
| rs1322639 | -0.1584 | 0.0209 | A | G | 0.2234 | 3.87E-14 |
| rs35261542 | 0.1196 | 0.0195 | A | C | 0.7321 | 9.29E-10 |
| rs6934891 | 0.1275 | 0.0177 | A | G | 0.5745 | 5.21E-13 |
| rs2744133 | 0.1435 | 0.0193 | A | G | 0.2749 | 1.17E-13 |
| rs9467545 | -0.2545 | 0.0237 | A | T | 0.1572 | 7.39E-27 |
| rs198851 | 0.3889 | 0.0244 | T | G | 0.8504 | 2.93E-57 |
| rs1265157 | 0.1444 | 0.0187 | C | G | 0.3521 | 1.18E-14 |
| rs440454 | -0.2602 | 0.0192 | A | G | 0.684 | 7.52E-42 |
| rs115447786 | 0.2904 | 0.0455 | T | C | 0.9573 | 1.75E-10 |
| rs6905288 | 0.1759 | 0.0179 | A | G | 0.4319 | 7.79E-23 |
| rs881858 | 0.1553 | 0.0191 | A | G | 0.306 | 4.65E-16 |
| rs2397060 | -0.161 | 0.0251 | T | C | 0.1405 | 1.46E-10 |
| rs1114347 | -0.1792 | 0.0173 | A | G | 0.4823 | 3.32E-25 |
| rs62413546 | -0.1877 | 0.032 | T | C | 0.9153 | 4.58E-09 |
| rs504691 | -0.1177 | 0.0177 | A | C | 0.5998 | 3.14E-11 |
| rs1984195 | 0.1736 | 0.0173 | A | G | 0.5117 | 1.43E-23 |
| rs9406076 | 0.101 | 0.0185 | T | C | 0.6722 | 4.65E-08 |
| rs16875357 | -0.1205 | 0.0203 | T | G | 0.2431 | 2.70E-09 |
| rs3798293 | -0.1328 | 0.021 | A | G | 0.2165 | 2.70E-10 |
| rs4556017 | -0.1601 | 0.0247 | T | C | 0.1476 | 9.67E-11 |
| rs2191046 | 0.1184 | 0.0197 | T | G | 0.2646 | 1.78E-09 |
| rs73033340 | 0.5312 | 0.0525 | A | G | 0.0362 | 5.06E-24 |
| rs11556924 | -0.181 | 0.0181 | T | C | 0.6173 | 1.83E-23 |
| rs13237249 | 0.1366 | 0.0177 | T | C | 0.602 | 1.03E-14 |
| rs75511781 | -0.3721 | 0.047 | A | G | 0.0425 | 2.45E-15 |
| rs7800558 | 0.096 | 0.0175 | T | C | 0.4219 | 4.46E-08 |
| rs13240040 | 0.1186 | 0.019 | A | G | 0.3164 | 3.98E-10 |
| rs1044608 | -0.2018 | 0.0339 | C | G | 0.0767 | 2.76E-09 |
| rs3918226 | 0.6117 | 0.0329 | T | C | 0.9187 | 5.31E-77 |
| rs4726006 | 0.1339 | 0.02 | A | G | 0.7452 | 2.39E-11 |
| rs6464165 | -0.217 | 0.0195 | T | C | 0.2809 | 7.34E-29 |
| rs9638084 | 0.1154 | 0.0178 | A | G | 0.6022 | 8.51E-11 |
| rs17432462 | -0.1036 | 0.0179 | T | C | 0.3766 | 7.31E-09 |
| rs6959688 | -0.1269 | 0.0178 | A | G | 0.4015 | 1.02E-12 |
| rs4507656 | -0.1487 | 0.0199 | C | G | 0.3066 | 8.69E-14 |
| rs2906152 | -0.1873 | 0.0181 | A | G | 0.3696 | 5.55E-25 |
| rs4722548 | -0.1346 | 0.0176 | T | C | 0.3996 | 1.99E-14 |
| rs3735533 | -0.487 | 0.0331 | T | C | 0.9258 | 6.32E-49 |
| rs6961048 | -0.2729 | 0.0286 | C | G | 0.1038 | 1.28E-21 |
| rs342977 | -0.1577 | 0.0205 | A | G | 0.2285 | 1.67E-14 |
| rs2854746 | 0.113 | 0.018 | C | G | 0.6003 | 3.26E-10 |
| rs17454517 | 0.1216 | 0.0174 | A | G | 0.5064 | 2.65E-12 |
| rs1178979 | 0.1504 | 0.0221 | T | C | 0.1953 | 9.96E-12 |
| rs5010183 | 0.1195 | 0.018 | T | C | 0.372 | 2.86E-11 |
| rs3807101 | -0.1743 | 0.0265 | T | C | 0.877 | 4.57E-11 |
| rs1449596 | -0.1085 | 0.0181 | C | G | 0.6455 | 1.92E-09 |
| rs7788746 | -0.1644 | 0.0183 | T | G | 0.3309 | 3.19E-19 |
| rs2978098 | 0.1548 | 0.0176 | A | C | 0.4535 | 1.33E-18 |
| rs142449193 | -0.2573 | 0.0426 | T | C | 0.954 | 1.51E-09 |
| rs2957468 | 0.1377 | 0.0185 | A | G | 0.6646 | 8.43E-14 |
| rs35091929 | 0.1828 | 0.0177 | T | C | 0.6032 | 6.46E-25 |
| rs722783 | -0.2093 | 0.0208 | A | G | 0.7784 | 9.03E-24 |
| rs9918907 | -0.1188 | 0.021 | A | G | 0.2162 | 1.59E-08 |
| rs7012891 | -0.1391 | 0.0205 | T | C | 0.2367 | 1.20E-11 |
| rs4909314 | 0.1339 | 0.0177 | A | T | 0.6052 | 3.41E-14 |
| rs4074812 | -0.1336 | 0.0175 | A | G | 0.4465 | 2.07E-14 |
| rs3802230 | -0.1605 | 0.0174 | A | C | 0.4554 | 2.75E-20 |
| rs62503324 | 0.2033 | 0.0204 | T | C | 0.7603 | 2.11E-23 |
| rs951914 | 0.1904 | 0.0193 | C | G | 0.2874 | 5.06E-23 |
| rs17832905 | 0.1923 | 0.0346 | A | C | 0.9283 | 2.81E-08 |
| rs17321041 | 0.2313 | 0.0363 | T | C | 0.9367 | 1.78E-10 |
| rs1906672 | 0.1402 | 0.0205 | A | G | 0.7676 | 8.48E-12 |
| rs10087280 | 0.1381 | 0.0232 | A | G | 0.1683 | 2.54E-09 |
| rs4873492 | 0.1401 | 0.0231 | T | C | 0.8275 | 1.28E-09 |
| rs2442618 | -0.1315 | 0.0177 | T | C | 0.4277 | 1.21E-13 |
| rs11778153 | 0.1192 | 0.0182 | T | C | 0.3569 | 5.84E-11 |
| rs6983239 | 0.1159 | 0.0211 | T | G | 0.7812 | 3.71E-08 |
| rs148401029 | -0.3122 | 0.0486 | A | C | 0.9648 | 1.32E-10 |
| rs56345595 | 0.1329 | 0.0177 | A | G | 0.4152 | 5.20E-14 |
| rs73276406 | 0.1564 | 0.0246 | C | G | 0.8543 | 1.99E-10 |
| rs4743021 | -0.108 | 0.0194 | T | C | 0.3147 | 2.41E-08 |
| rs10980408 | -0.3745 | 0.0477 | T | C | 0.0358 | 4.17E-15 |
| rs10759697 | 0.1308 | 0.0173 | A | G | 0.5094 | 3.94E-14 |
| rs2133386 | -0.1322 | 0.0176 | A | C | 0.5673 | 5.21E-14 |
| rs507666 | -0.2854 | 0.0223 | A | G | 0.8128 | 2.27E-37 |
| rs6271 | -0.4313 | 0.0352 | T | C | 0.9263 | 1.72E-34 |
| rs11145807 | 0.155 | 0.0184 | A | G | 0.5942 | 4.10E-17 |
| rs4615669 | -0.114 | 0.0174 | A | G | 0.4403 | 6.10E-11 |
| rs12216886 | 0.1292 | 0.0221 | T | G | 0.1923 | 4.76E-09 |
| rs1243876 | -0.1063 | 0.019 | T | C | 0.2988 | 2.14E-08 |
| rs76452347 | -0.2246 | 0.0229 | T | C | 0.7947 | 9.37E-23 |
| rs12337056 | 0.1364 | 0.0228 | T | C | 0.8239 | 2.18E-09 |
| rs11141731 | -0.1258 | 0.0207 | T | C | 0.772 | 1.31E-09 |
| rs1332812 | -0.1145 | 0.0181 | A | T | 0.3531 | 2.71E-10 |

**Table S17. Genome-wide significant and independent SNPs that were used as instruments for PP**

| SNP | beta. | se. | effect | other | eaf.exposure | pval.exposure |
| --- | --- | --- | --- | --- | --- | --- |
| rs11190709 | 0.337 | 0.0328 | A | G | 0.1119 | 8.41E-25 |
| rs138112129 | 0.4006 | 0.0624 | A | T | 0.9654 | 1.34E-10 |
| rs112913898 | -0.5815 | 0.0376 | A | G | 0.9179 | 5.68E-54 |
| rs10885409 | -0.1879 | 0.0205 | T | C | 0.4675 | 5.17E-20 |
| rs10787515 | -0.1354 | 0.0206 | T | C | 0.4785 | 4.73E-11 |
| rs72830615 | 0.1794 | 0.0211 | A | G | 0.4017 | 1.59E-17 |
| rs11257655 | 0.139 | 0.0252 | T | C | 0.7904 | 3.67E-08 |
| rs1133400 | -0.1609 | 0.0255 | A | G | 0.2143 | 2.96E-10 |
| rs1779240 | -0.2018 | 0.0241 | A | G | 0.2357 | 5.21E-17 |
| rs12258967 | 0.2786 | 0.0229 | C | G | 0.2956 | 3.67E-34 |
| rs11010470 | 0.1153 | 0.0205 | T | C | 0.5006 | 1.97E-08 |
| rs2148306 | -0.1802 | 0.0207 | A | C | 0.4227 | 2.78E-18 |
| rs9337951 | 0.2583 | 0.0227 | A | G | 0.6585 | 4.24E-30 |
| rs3006576 | 0.1923 | 0.0224 | T | C | 0.296 | 9.05E-18 |
| rs12264186 | 0.1972 | 0.0263 | T | C | 0.8127 | 6.90E-14 |
| rs4245599 | -0.1548 | 0.0207 | A | G | 0.5421 | 7.70E-14 |
| rs7099368 | 0.1417 | 0.0209 | T | C | 0.41 | 1.16E-11 |
| rs57946343 | 0.2405 | 0.0289 | T | C | 0.1475 | 8.47E-17 |
| rs6415872 | 0.1215 | 0.0206 | A | G | 0.5087 | 3.70E-09 |
| rs7095472 | 0.1156 | 0.0211 | A | G | 0.5308 | 4.12E-08 |
| rs55947600 | -0.1895 | 0.0206 | A | G | 0.5377 | 4.10E-20 |
| rs10887914 | 0.1461 | 0.0205 | T | C | 0.5373 | 1.13E-12 |
| rs7070115 | -0.2565 | 0.0208 | A | G | 0.4304 | 4.63E-35 |
| rs11187998 | -0.1403 | 0.0206 | A | G | 0.5648 | 1.06E-11 |
| rs604723 | -0.2689 | 0.023 | T | C | 0.7245 | 1.46E-31 |
| rs56287081 | 0.2018 | 0.0262 | A | G | 0.811 | 1.40E-14 |
| rs1834596 | -0.1325 | 0.0216 | T | C | 0.6538 | 9.37E-10 |
| rs10890706 | -0.1622 | 0.0223 | T | C | 0.3163 | 3.36E-13 |
| rs573455 | 0.2515 | 0.0206 | A | G | 0.5386 | 2.37E-34 |
| rs78799967 | -0.4695 | 0.0689 | T | C | 0.9735 | 9.39E-12 |
| rs11222084 | -0.4972 | 0.0214 | A | T | 0.3626 | ####### |
| rs10736585 | 0.1945 | 0.0217 | T | C | 0.3355 | 2.82E-19 |
| rs11603014 | 0.1733 | 0.0258 | A | G | 0.8033 | 1.89E-11 |
| rs1519125 | 0.1403 | 0.0209 | C | G | 0.6066 | 2.02E-11 |
| rs10832778 | 0.2211 | 0.0212 | C | G | 0.6191 | 1.41E-25 |
| rs686722 | 0.3174 | 0.022 | T | C | 0.6381 | 4.20E-47 |
| rs10766533 | 0.1255 | 0.0229 | A | T | 0.2885 | 4.07E-08 |
| rs74048200 | -0.2268 | 0.039 | A | G | 0.0861 | 6.05E-09 |
| rs11031051 | -0.172 | 0.0222 | A | C | 0.3096 | 1.04E-14 |
| rs4922591 | -0.1513 | 0.0214 | T | C | 0.6133 | 1.39E-12 |
| rs11037808 | 0.1366 | 0.0224 | T | C | 0.3052 | 9.92E-10 |
| rs714417 | -0.2229 | 0.0224 | T | C | 0.7001 | 2.53E-23 |
| rs7107356 | -0.234 | 0.0205 | A | G | 0.5044 | 3.24E-30 |
| rs11607056 | -0.1829 | 0.0218 | T | C | 0.6728 | 4.77E-17 |
| rs4980515 | 0.1628 | 0.0205 | T | C | 0.5012 | 2.29E-15 |
| rs144822931 | 0.5092 | 0.0797 | T | C | 0.0175 | 1.71E-10 |
| rs7119612 | -0.246 | 0.0354 | T | C | 0.9059 | 3.64E-12 |
| rs1687692 | 0.1594 | 0.026 | A | G | 0.7998 | 8.57E-10 |
| rs4075289 | 0.1539 | 0.0233 | T | G | 0.293 | 4.12E-11 |
| rs2289125 | -0.3847 | 0.0255 | A | C | 0.7798 | 1.82E-51 |
| rs11021221 | 0.1813 | 0.0276 | A | T | 0.8336 | 4.79E-11 |
| rs1520222 | 0.1285 | 0.0232 | A | G | 0.2626 | 2.89E-08 |
| rs11065861 | 0.1684 | 0.0249 | A | G | 0.2158 | 1.37E-11 |
| rs1061651 | 0.1304 | 0.0239 | T | C | 0.2648 | 4.95E-08 |
| rs35429 | 0.1777 | 0.0212 | A | G | 0.3869 | 4.94E-17 |
| rs11609905 | -0.1817 | 0.0229 | T | C | 0.7252 | 2.27E-15 |
| rs7313556 | -0.1396 | 0.0214 | A | G | 0.652 | 6.79E-11 |
| rs10770612 | 0.3421 | 0.0259 | A | G | 0.2025 | 5.79E-40 |
| rs704191 | 0.1628 | 0.0206 | T | C | 0.5369 | 2.74E-15 |
| rs3819532 | -0.1337 | 0.0209 | T | C | 0.6088 | 1.45E-10 |
| rs61915422 | 0.2031 | 0.0315 | C | G | 0.1295 | 1.14E-10 |
| rs11052722 | 0.1127 | 0.0206 | A | G | 0.4807 | 4.73E-08 |
| rs486098 | 0.1536 | 0.0232 | T | C | 0.7309 | 3.68E-11 |
| rs2261608 | 0.1704 | 0.0213 | A | T | 0.6488 | 1.35E-15 |
| rs150857355 | 0.5272 | 0.0765 | C | G | 0.9783 | 5.50E-12 |
| rs58278271 | -0.2276 | 0.0368 | A | G | 0.9154 | 6.39E-10 |
| rs67772913 | 0.2307 | 0.0225 | A | G | 0.3041 | 1.14E-24 |
| rs1351394 | -0.1811 | 0.0204 | T | C | 0.5108 | 6.53E-19 |
| rs4842266 | -0.1675 | 0.0222 | A | G | 0.3138 | 4.95E-14 |
| rs111478946 | -0.4623 | 0.0276 | A | G | 0.8331 | 8.22E-63 |
| rs114697502 | -0.3813 | 0.0378 | T | C | 0.9193 | 5.83E-24 |
| rs7977311 | -0.199 | 0.032 | T | C | 0.8844 | 4.88E-10 |
| rs3742182 | -0.1863 | 0.0261 | T | C | 0.1899 | 9.00E-13 |
| rs9549328 | 0.2164 | 0.0247 | T | C | 0.7696 | 1.77E-18 |
| rs7321688 | 0.1888 | 0.0242 | A | C | 0.7672 | 6.48E-15 |
| rs629445 | -0.1319 | 0.021 | A | G | 0.6106 | 3.53E-10 |
| rs7338758 | 0.1575 | 0.024 | T | C | 0.7561 | 5.49E-11 |
| rs9532798 | 0.1395 | 0.0241 | T | C | 0.2416 | 7.62E-09 |
| rs7491248 | 0.1544 | 0.0247 | A | G | 0.776 | 3.94E-10 |
| rs4304924 | -0.1198 | 0.0208 | A | G | 0.4323 | 8.67E-09 |
| rs4287430 | -0.1143 | 0.0209 | A | T | 0.5619 | 4.55E-08 |
| rs17562391 | 0.1713 | 0.0209 | T | C | 0.5818 | 2.32E-16 |
| rs75016974 | -0.219 | 0.0299 | T | C | 0.858 | 2.50E-13 |
| rs11626434 | -0.1345 | 0.0219 | C | G | 0.6384 | 7.87E-10 |
| rs8017780 | 0.162 | 0.0251 | A | C | 0.7861 | 1.14E-10 |
| rs365990 | 0.3079 | 0.0213 | A | G | 0.3663 | 1.75E-47 |
| rs696 | 0.2107 | 0.0214 | T | C | 0.6317 | 7.48E-23 |
| rs142004400 | 0.3306 | 0.0566 | A | C | 0.0361 | 5.24E-09 |
| rs8009633 | 0.2072 | 0.0245 | C | G | 0.7644 | 2.36E-17 |
| rs2215590 | 0.1732 | 0.0235 | T | C | 0.7451 | 1.67E-13 |
| rs1866628 | 0.1201 | 0.0205 | T | C | 0.5232 | 4.82E-09 |
| rs11627326 | 0.1546 | 0.0227 | C | G | 0.7129 | 9.78E-12 |
| rs753361 | 0.1289 | 0.0215 | A | G | 0.5789 | 2.03E-09 |
| rs17732513 | -0.1465 | 0.0216 | T | C | 0.649 | 1.10E-11 |
| rs8010344 | 0.1513 | 0.0269 | A | G | 0.1799 | 1.81E-08 |
| rs7154431 | -0.2001 | 0.021 | A | T | 0.3855 | 1.77E-21 |
| rs11629850 | 0.1177 | 0.0205 | A | G | 0.4712 | 9.65E-09 |
| rs7178506 | -0.1229 | 0.0216 | T | C | 0.3882 | 1.30E-08 |
| rs2015637 | 0.5012 | 0.0345 | T | C | 0.0996 | 8.89E-48 |
| rs3098186 | -0.1735 | 0.0207 | T | C | 0.4844 | 4.40E-17 |
| rs17271730 | 0.1631 | 0.0213 | A | G | 0.3628 | 2.13E-14 |
| rs55962736 | -0.1562 | 0.0205 | T | G | 0.4906 | 2.49E-14 |
| rs1965942 | 0.1276 | 0.0217 | A | G | 0.4614 | 3.78E-09 |
| rs3784790 | -0.1544 | 0.0231 | C | G | 0.2676 | 2.37E-11 |
| rs4491476 | -0.1678 | 0.0211 | A | G | 0.5903 | 1.89E-15 |
| rs11634851 | -0.1857 | 0.0206 | C | G | 0.4647 | 1.61E-19 |
| rs11637880 | 0.1146 | 0.021 | C | G | 0.5818 | 4.52E-08 |
| rs7497304 | 0.2821 | 0.0223 | T | G | 0.6731 | 1.39E-36 |
| rs11632112 | -0.1645 | 0.0241 | C | G | 0.7598 | 8.66E-12 |
| rs11248862 | 0.2282 | 0.0315 | A | G | 0.875 | 4.68E-13 |
| rs30232 | -0.1234 | 0.0209 | A | G | 0.4175 | 3.37E-09 |
| rs3915425 | 0.1913 | 0.022 | T | C | 0.3182 | 4.00E-18 |
| rs200528 | -0.2295 | 0.0258 | A | G | 0.8069 | 6.38E-19 |
| rs59945160 | -0.1399 | 0.0256 | C | G | 0.2353 | 4.47E-08 |
| rs8052826 | 0.1683 | 0.0254 | A | G | 0.7887 | 3.40E-11 |
| rs7214 | 0.1255 | 0.0207 | T | G | 0.4313 | 1.32E-09 |
| rs9937815 | -0.1386 | 0.022 | A | G | 0.3266 | 2.87E-10 |
| rs37060 | 0.1489 | 0.0237 | A | G | 0.7535 | 3.12E-10 |
| rs8053909 | -0.1309 | 0.0228 | C | G | 0.3395 | 8.80E-09 |
| rs12149704 | 0.7476 | 0.0466 | A | G | 0.937 | 5.46E-58 |
| rs62055086 | 0.1854 | 0.0243 | T | C | 0.7078 | 2.55E-14 |
| rs7500448 | 0.3589 | 0.0239 | A | G | 0.2536 | 3.62E-51 |
| rs28651151 | 0.1448 | 0.0231 | T | G | 0.2752 | 3.59E-10 |
| rs7499959 | -0.1405 | 0.0232 | C | G | 0.3046 | 1.51E-09 |
| rs75570604 | 0.2057 | 0.0361 | C | G | 0.9057 | 1.23E-08 |
| rs138285687 | -0.3304 | 0.0517 | T | C | 0.9567 | 1.68E-10 |
| rs11656495 | -0.1253 | 0.0219 | A | T | 0.44 | 1.07E-08 |
| rs324075 | 0.2009 | 0.0276 | A | G | 0.1869 | 3.52E-13 |
| rs12603813 | -0.2683 | 0.0237 | T | C | 0.2527 | 8.31E-30 |
| rs17608766 | -0.5274 | 0.0295 | T | C | 0.1443 | 2.12E-71 |
| rs9747001 | -0.209 | 0.0251 | A | G | 0.7917 | 8.84E-17 |
| rs2288277 | -0.2645 | 0.036 | T | C | 0.9096 | 2.16E-13 |
| rs2109019 | -0.2464 | 0.0256 | A | C | 0.7882 | 5.20E-22 |
| rs56288724 | -0.2377 | 0.0211 | A | G | 0.4174 | 1.99E-29 |
| rs4968716 | 0.1364 | 0.0211 | T | C | 0.4798 | 9.84E-11 |
| rs6504252 | -0.2951 | 0.0504 | T | C | 0.9481 | 4.70E-09 |
| rs4796514 | -0.2313 | 0.021 | T | C | 0.3914 | 4.11E-28 |
| rs222837 | 0.1265 | 0.0209 | T | C | 0.4886 | 1.34E-09 |
| rs34587622 | -0.2084 | 0.035 | T | C | 0.8903 | 2.49E-09 |
| rs62062581 | 0.1903 | 0.0282 | T | G | 0.1684 | 1.56E-11 |
| rs78378222 | 1.0488 | 0.0945 | T | G | 0.0139 | 1.28E-28 |
| rs9302885 | 0.1208 | 0.0206 | A | G | 0.5545 | 4.12E-09 |
| rs929581 | 0.1318 | 0.0213 | T | C | 0.3544 | 5.73E-10 |
| rs9957388 | -0.2373 | 0.0218 | C | G | 0.6732 | 1.65E-27 |
| rs11874246 | 0.1574 | 0.0224 | T | C | 0.7043 | 1.93E-12 |
| rs7236548 | 0.3621 | 0.0264 | A | C | 0.8152 | 8.48E-43 |
| rs11872627 | -0.2532 | 0.0298 | T | C | 0.8611 | 1.77E-17 |
| rs663640 | -0.1547 | 0.025 | T | C | 0.783 | 5.94E-10 |
| rs12172847 | -0.1278 | 0.0218 | A | G | 0.6775 | 4.68E-09 |
| rs1047922 | -0.2121 | 0.0297 | T | C | 0.1516 | 9.70E-13 |
| rs34413141 | -0.162 | 0.0267 | A | T | 0.8176 | 1.39E-09 |
| rs17248720 | -0.2289 | 0.0326 | T | C | 0.8824 | 2.08E-12 |
| rs74179970 | 0.272 | 0.0387 | A | G | 0.9188 | 2.09E-12 |
| rs3760994 | -0.144 | 0.0218 | A | G | 0.5084 | 4.27E-11 |
| rs7245814 | -0.2909 | 0.0345 | A | G | 0.9031 | 3.75E-17 |
| rs8102624 | 0.5573 | 0.0393 | A | G | 0.9228 | 1.11E-45 |
| rs28572357 | -0.1504 | 0.0209 | A | C | 0.3971 | 6.83E-13 |
| rs117557920 | 0.2363 | 0.0275 | A | G | 0.8057 | 8.99E-18 |
| rs1800470 | -0.15 | 0.0213 | A | G | 0.3766 | 1.76E-12 |
| rs7412 | -0.3769 | 0.0391 | T | C | 0.9182 | 5.73E-22 |
| rs74889068 | 0.2053 | 0.0298 | A | G | 0.8547 | 5.48E-12 |
| rs916904 | 0.1199 | 0.0217 | A | G | 0.6293 | 3.33E-08 |
| rs36047283 | 0.3743 | 0.0334 | A | G | 0.1235 | 3.42E-29 |
| rs10776752 | 0.3735 | 0.0392 | T | G | 0.9199 | 1.51E-21 |
| rs17037452 | 0.3833 | 0.0278 | A | G | 0.1608 | 2.83E-43 |
| rs307359 | -0.3344 | 0.0442 | A | G | 0.9312 | 3.92E-14 |
| rs11585169 | 0.1557 | 0.0209 | A | T | 0.4225 | 8.30E-14 |
| rs75461554 | -0.1959 | 0.0256 | T | C | 0.7993 | 1.84E-14 |
| rs7796 | 0.2021 | 0.0213 | C | G | 0.4883 | 2.83E-21 |
| rs12138150 | -0.1954 | 0.0208 | T | C | 0.5979 | 5.66E-21 |
| rs58232567 | -0.32 | 0.0523 | A | G | 0.9561 | 9.62E-10 |
| rs3753802 | 0.1161 | 0.0209 | T | C | 0.3972 | 2.71E-08 |
| rs10914057 | 0.1155 | 0.0209 | T | C | 0.3998 | 3.49E-08 |
| rs4559481 | -0.1174 | 0.021 | A | G | 0.5181 | 2.30E-08 |
| rs4950838 | 0.212 | 0.0346 | T | C | 0.0985 | 8.60E-10 |
| rs558248 | 0.1915 | 0.0212 | A | G | 0.378 | 1.61E-19 |
| rs263532 | 0.1186 | 0.0208 | T | C | 0.4244 | 1.25E-08 |
| rs17042848 | 0.1497 | 0.0265 | A | T | 0.8161 | 1.61E-08 |
| rs2820443 | 0.1857 | 0.0224 | T | C | 0.2911 | 1.30E-16 |
| rs11580654 | -0.2087 | 0.035 | A | G | 0.9043 | 2.56E-09 |
| rs2493134 | -0.1344 | 0.0209 | T | C | 0.4068 | 1.35E-10 |
| rs150266910 | 0.1731 | 0.0267 | T | C | 0.8232 | 9.57E-11 |
| rs6598886 | 0.218 | 0.0367 | T | C | 0.088 | 2.97E-09 |
| rs143167197 | -0.3069 | 0.0419 | A | G | 0.0715 | 2.49E-13 |
| rs2493296 | 0.1894 | 0.03 | T | C | 0.8576 | 2.64E-10 |
| rs4360494 | 0.2978 | 0.0215 | C | G | 0.4487 | 1.29E-43 |
| rs12045477 | -0.176 | 0.0228 | T | C | 0.7119 | 1.16E-14 |
| rs1198982 | -0.1242 | 0.0211 | A | G | 0.3879 | 3.93E-09 |
| rs72664332 | 0.2758 | 0.0354 | A | C | 0.0922 | 6.81E-15 |
| rs17535443 | -0.3649 | 0.023 | A | G | 0.727 | 7.60E-57 |
| rs949827 | 0.1523 | 0.0218 | T | C | 0.3253 | 2.63E-12 |
| rs9661802 | 0.1377 | 0.0218 | A | C | 0.3345 | 2.66E-10 |
| rs72676189 | -0.204 | 0.0294 | A | G | 0.1405 | 3.62E-12 |
| rs385437 | 0.1626 | 0.0297 | A | G | 0.1397 | 4.51E-08 |
| rs786923 | -0.1984 | 0.021 | T | C | 0.3764 | 3.98E-21 |
| rs12032588 | -0.1294 | 0.0208 | T | G | 0.6007 | 5.12E-10 |
| rs2143618 | -0.2211 | 0.0277 | A | G | 0.1638 | 1.41E-15 |
| rs2206815 | -0.3609 | 0.0207 | A | C | 0.5022 | 4.27E-68 |
| rs6078000 | -0.1946 | 0.0226 | A | G | 0.2844 | 8.02E-18 |
| rs73900405 | 0.1651 | 0.0237 | A | G | 0.2482 | 3.34E-12 |
| rs6141767 | 0.2147 | 0.0286 | C | G | 0.8435 | 5.63E-14 |
| rs6031431 | -0.1575 | 0.0207 | A | G | 0.4625 | 2.72E-14 |
| rs11906149 | 0.2276 | 0.0318 | C | G | 0.8793 | 7.69E-13 |
| rs8118848 | -0.1881 | 0.0249 | A | G | 0.7597 | 4.35E-14 |
| rs4347920 | -0.1263 | 0.0211 | A | C | 0.5966 | 2.23E-09 |
| rs2229742 | 0.2206 | 0.034 | C | G | 0.8961 | 8.63E-11 |
| rs2255055 | -0.1259 | 0.0211 | T | C | 0.6184 | 2.32E-09 |
| rs2834440 | 0.1174 | 0.021 | A | G | 0.3796 | 2.27E-08 |
| rs112005532 | -0.4967 | 0.0574 | T | C | 0.0393 | 4.70E-18 |
| rs12627651 | 0.134 | 0.0232 | A | G | 0.7128 | 8.16E-09 |
| rs68100343 | 0.1425 | 0.0235 | T | C | 0.7352 | 1.28E-09 |
| rs4819852 | 0.2486 | 0.0228 | A | G | 0.7129 | 8.73E-28 |
| rs9608690 | -0.2489 | 0.0409 | A | G | 0.9319 | 1.15E-09 |
| rs5753103 | 0.1377 | 0.0206 | A | G | 0.549 | 2.62E-11 |
| rs139919 | -0.2476 | 0.0272 | T | C | 0.1803 | 8.04E-20 |
| rs6006987 | 0.1312 | 0.0229 | A | C | 0.7231 | 1.08E-08 |
| rs6747874 | 0.1704 | 0.0247 | A | G | 0.7775 | 5.02E-12 |
| rs150194832 | -0.2147 | 0.0354 | C | G | 0.9067 | 1.35E-09 |
| rs4664080 | -0.135 | 0.0209 | A | G | 0.6022 | 1.03E-10 |
| rs72874178 | -0.3788 | 0.0242 | A | G | 0.7655 | 4.29E-55 |
| rs75758489 | -0.1763 | 0.0291 | T | C | 0.1777 | 1.36E-09 |
| rs560887 | -0.1904 | 0.0223 | T | C | 0.7011 | 1.57E-17 |
| rs72884380 | -0.1245 | 0.021 | T | C | 0.3793 | 3.11E-09 |
| rs2358891 | 0.1593 | 0.0241 | A | G | 0.7545 | 4.08E-11 |
| rs10497529 | -0.4509 | 0.0575 | A | G | 0.9648 | 4.67E-15 |
| rs7603849 | -0.1564 | 0.0205 | A | C | 0.522 | 2.28E-14 |
| rs1344652 | -0.3455 | 0.0219 | A | G | 0.6834 | 3.93E-56 |
| rs62111832 | 0.2373 | 0.0414 | A | G | 0.9336 | 9.78E-09 |
| rs1469760 | -0.1891 | 0.0208 | T | C | 0.4162 | 1.16E-19 |
| rs3845811 | -0.1613 | 0.021 | C | G | 0.4339 | 1.58E-14 |
| rs12476956 | 0.1307 | 0.0211 | T | C | 0.5485 | 6.15E-10 |
| rs1250259 | -0.2782 | 0.0233 | A | T | 0.2619 | 8.61E-33 |
| rs4674114 | -0.2116 | 0.0256 | A | G | 0.799 | 1.28E-16 |
| rs4441458 | -0.1289 | 0.0227 | T | C | 0.7171 | 1.42E-08 |
| rs12052878 | -0.1497 | 0.022 | A | G | 0.6802 | 1.11E-11 |
| rs11685352 | -0.1715 | 0.0264 | A | G | 0.8162 | 8.80E-11 |
| rs1275957 | -0.2082 | 0.0214 | T | G | 0.4082 | 2.62E-22 |
| rs4952955 | -0.1424 | 0.0258 | T | C | 0.7983 | 3.48E-08 |
| rs6544652 | -0.1441 | 0.024 | T | C | 0.7642 | 1.95E-09 |
| rs11690961 | 0.3036 | 0.0319 | A | C | 0.1167 | 1.93E-21 |
| rs13409792 | 0.175 | 0.0294 | A | G | 0.1404 | 2.58E-09 |
| rs4672081 | -0.142 | 0.0206 | T | C | 0.435 | 4.98E-12 |
| rs925484 | -0.1492 | 0.0209 | C | G | 0.4007 | 8.66E-13 |
| rs2540951 | 0.2243 | 0.021 | A | G | 0.3787 | 1.26E-26 |
| rs6731373 | 0.1336 | 0.0221 | A | G | 0.6503 | 1.43E-09 |
| rs7578166 | 0.1383 | 0.0209 | A | C | 0.6139 | 4.06E-11 |
| rs11689667 | 0.2029 | 0.0206 | T | C | 0.4556 | 7.36E-23 |
| rs2175337 | 0.1656 | 0.021 | A | C | 0.3892 | 3.52E-15 |
| rs7058 | 0.1804 | 0.0206 | T | G | 0.537 | 1.86E-18 |
| rs9848170 | 0.1926 | 0.0208 | C | G | 0.403 | 2.43E-20 |
| rs1599116 | -0.1682 | 0.0292 | T | G | 0.1455 | 8.66E-09 |
| rs6806529 | 0.1372 | 0.0209 | A | C | 0.5662 | 5.81E-11 |
| rs3796205 | -0.1211 | 0.0216 | C | G | 0.643 | 2.03E-08 |
| rs60672471 | 0.1919 | 0.0333 | T | C | 0.1071 | 7.96E-09 |
| rs62270945 | 0.5276 | 0.0651 | T | C | 0.9711 | 5.17E-16 |
| rs62278541 | 0.1677 | 0.0214 | A | G | 0.3526 | 4.84E-15 |
| rs6766170 | -0.165 | 0.0207 | A | C | 0.5067 | 1.36E-15 |
| rs9860302 | -0.1365 | 0.0227 | A | G | 0.2809 | 1.89E-09 |
| rs76183925 | -0.2019 | 0.0331 | T | C | 0.1103 | 1.12E-09 |
| rs9835962 | -0.2514 | 0.0392 | A | C | 0.9251 | 1.40E-10 |
| rs1918973 | 0.1253 | 0.0205 | A | G | 0.5416 | 9.91E-10 |
| rs12630450 | 0.1947 | 0.0235 | A | G | 0.2671 | 1.22E-16 |
| rs263017 | 0.1356 | 0.0204 | A | G | 0.5047 | 3.23E-11 |
| rs1773242 | -0.119 | 0.0218 | C | G | 0.3568 | 4.97E-08 |
| rs9310608 | -0.1673 | 0.0295 | T | C | 0.8565 | 1.45E-08 |
| rs2055120 | -0.5578 | 0.0722 | A | G | 0.0223 | 1.11E-14 |
| rs75487052 | 0.2625 | 0.021 | A | T | 0.392 | 9.46E-36 |
| rs7640747 | -0.1579 | 0.0214 | C | G | 0.383 | 1.62E-13 |
| rs12636123 | 0.1283 | 0.0235 | T | G | 0.3446 | 4.75E-08 |
| rs6788984 | 0.1882 | 0.0293 | A | G | 0.144 | 1.31E-10 |
| rs9839213 | -0.5316 | 0.0279 | T | C | 0.8299 | 4.03E-81 |
| rs10433615 | 0.1655 | 0.021 | T | C | 0.6065 | 3.40E-15 |
| rs7630745 | 0.1636 | 0.0215 | T | C | 0.3409 | 2.73E-14 |
| rs56090516 | -0.1304 | 0.0216 | T | C | 0.3377 | 1.56E-09 |
| rs9835724 | 0.1626 | 0.0221 | A | G | 0.3148 | 1.82E-13 |
| rs9860290 | -0.1737 | 0.0252 | A | G | 0.7908 | 5.22E-12 |
| rs1229984 | -0.5111 | 0.0607 | T | C | 0.9607 | 3.57E-17 |
| rs13107325 | -0.2527 | 0.0401 | T | C | 0.926 | 2.91E-10 |
| rs77301788 | -0.1633 | 0.0221 | T | C | 0.3094 | 1.67E-13 |
| rs11933087 | -0.1213 | 0.0218 | A | T | 0.3662 | 2.81E-08 |
| rs78806058 | -0.1873 | 0.0312 | A | G | 0.8644 | 1.95E-09 |
| rs11100902 | 0.1572 | 0.0207 | A | G | 0.5157 | 3.21E-14 |
| rs10305838 | -0.2542 | 0.0293 | T | C | 0.1408 | 4.65E-18 |
| rs4076789 | -0.1281 | 0.0231 | A | G | 0.7341 | 2.93E-08 |
| rs4691670 | -0.2359 | 0.0205 | T | C | 0.4671 | 1.11E-30 |
| rs1850507 | 0.168 | 0.026 | T | G | 0.1966 | 1.10E-10 |
| rs999958 | -0.2208 | 0.0205 | A | C | 0.5172 | 5.69E-27 |
| rs2610990 | -0.1586 | 0.0233 | A | G | 0.7364 | 1.06E-11 |
| rs231708 | -0.2098 | 0.0221 | C | G | 0.3084 | 2.60E-21 |
| rs2498323 | 0.2957 | 0.035 | A | G | 0.9018 | 3.07E-17 |
| rs28702684 | -0.1347 | 0.0205 | C | G | 0.5023 | 4.76E-11 |
| rs2102397 | 0.1616 | 0.021 | A | C | 0.4936 | 1.58E-14 |
| rs60991988 | 0.5294 | 0.0337 | T | G | 0.1069 | 1.44E-55 |
| rs6851147 | -0.1898 | 0.0231 | C | G | 0.2732 | 1.98E-16 |
| rs28418670 | -0.1335 | 0.0206 | C | G | 0.5605 | 8.69E-11 |
| rs10857147 | -0.3582 | 0.0232 | A | T | 0.283 | 8.02E-54 |
| rs6823199 | 0.1567 | 0.0236 | T | C | 0.2565 | 3.07E-11 |
| rs17010957 | -0.3456 | 0.0292 | T | C | 0.1461 | 2.30E-32 |
| rs13149209 | 0.1769 | 0.0249 | T | C | 0.2224 | 1.24E-12 |
| rs79409628 | -0.3086 | 0.0368 | T | G | 0.9154 | 5.24E-17 |
| rs71594307 | 0.2664 | 0.0488 | A | G | 0.9513 | 4.83E-08 |
| rs1644318 | -0.2308 | 0.021 | T | C | 0.3866 | 3.92E-28 |
| rs75887402 | 0.5292 | 0.065 | T | C | 0.029 | 4.05E-16 |
| rs13189347 | -0.1408 | 0.0206 | A | C | 0.448 | 8.13E-12 |
| rs2242652 | 0.1577 | 0.0281 | A | G | 0.8049 | 1.93E-08 |
| rs702395 | 0.1437 | 0.0207 | T | C | 0.5634 | 4.17E-12 |
| rs853170 | 0.152 | 0.0233 | T | C | 0.2628 | 7.13E-11 |
| rs256824 | -0.1408 | 0.0232 | T | C | 0.7293 | 1.36E-09 |
| rs7707563 | 0.1545 | 0.0238 | T | C | 0.7555 | 8.84E-11 |
| rs10076730 | 0.2217 | 0.0211 | T | C | 0.3749 | 8.02E-26 |
| rs10052777 | -0.2457 | 0.021 | T | C | 0.3931 | 1.70E-31 |
| rs251252 | 0.1249 | 0.022 | T | C | 0.6694 | 1.35E-08 |
| rs12153395 | -0.2134 | 0.033 | A | G | 0.8855 | 1.00E-10 |
| rs7733331 | -0.3378 | 0.0209 | T | C | 0.6008 | 6.01E-59 |
| rs10941043 | -0.1272 | 0.0225 | T | G | 0.29 | 1.65E-08 |
| rs10939913 | -0.1299 | 0.0234 | C | G | 0.7433 | 2.99E-08 |
| rs1694068 | 0.128 | 0.0211 | A | T | 0.3859 | 1.22E-09 |
| rs9291825 | -0.1274 | 0.0206 | A | G | 0.5162 | 5.91E-10 |
| rs72761109 | 0.1583 | 0.0223 | T | C | 0.6971 | 1.19E-12 |
| rs10076149 | 0.1788 | 0.0205 | C | G | 0.4644 | 2.89E-18 |
| rs13356445 | -0.1415 | 0.025 | T | C | 0.7827 | 1.45E-08 |
| rs76443575 | -0.3167 | 0.0553 | C | G | 0.964 | 9.98E-09 |
| rs9486916 | 0.1842 | 0.0261 | T | C | 0.8025 | 1.84E-12 |
| rs4946265 | 0.1546 | 0.0205 | A | G | 0.488 | 5.38E-14 |
| rs11154027 | 0.1439 | 0.0208 | T | C | 0.539 | 4.47E-12 |
| rs73767089 | 0.2843 | 0.0332 | T | C | 0.1082 | 1.08E-17 |
| rs13199674 | 0.2204 | 0.0207 | A | G | 0.5571 | 1.65E-26 |
| rs9349379 | 0.2677 | 0.0212 | A | G | 0.4068 | 1.32E-36 |
| rs7763294 | -0.1551 | 0.022 | T | G | 0.6838 | 1.87E-12 |
| rs2328473 | -0.126 | 0.0209 | A | G | 0.5983 | 1.71E-09 |
| rs57139556 | 0.3087 | 0.0399 | A | G | 0.0714 | 1.02E-14 |
| rs9340985 | -0.4763 | 0.033 | T | C | 0.1095 | 4.20E-47 |
| rs13206305 | -0.156 | 0.0259 | T | C | 0.8029 | 1.78E-09 |
| rs7774311 | -0.3547 | 0.0288 | A | G | 0.1511 | 9.11E-35 |
| rs12180739 | -0.184 | 0.0207 | C | G | 0.5576 | 6.67E-19 |
| rs56255660 | 0.2339 | 0.0236 | A | C | 0.7422 | 4.23E-23 |
| rs12216497 | 0.1307 | 0.0206 | T | C | 0.4384 | 2.25E-10 |
| rs9356816 | -0.1292 | 0.0236 | A | G | 0.2498 | 4.35E-08 |
| rs6926677 | 0.2137 | 0.0259 | C | G | 0.8054 | 1.60E-16 |
| rs3134950 | 0.2928 | 0.0222 | A | C | 0.3758 | 8.16E-40 |
| rs2395655 | 0.1566 | 0.0211 | A | G | 0.3882 | 1.08E-13 |
| rs4594944 | -0.1256 | 0.0221 | A | G | 0.3159 | 1.38E-08 |
| rs1563788 | 0.2119 | 0.0226 | T | C | 0.7134 | 5.81E-21 |
| rs631441 | -0.1543 | 0.0222 | T | G | 0.3053 | 3.56E-12 |
| rs12201429 | 0.3763 | 0.0298 | T | C | 0.8618 | 1.50E-36 |
| rs12195276 | -0.1819 | 0.0231 | T | C | 0.2748 | 3.49E-15 |
| rs6920534 | 0.2738 | 0.0357 | T | C | 0.0973 | 1.62E-14 |
| rs9392172 | -0.1845 | 0.0205 | C | G | 0.4641 | 2.57E-19 |
| rs1124000 | 0.1237 | 0.022 | A | G | 0.6804 | 1.92E-08 |
| rs60255247 | 0.2491 | 0.033 | A | C | 0.1125 | 4.53E-14 |
| rs2983896 | 0.2127 | 0.0249 | A | G | 0.7815 | 1.26E-17 |
| rs72943207 | 0.1439 | 0.0258 | A | G | 0.7981 | 2.33E-08 |
| rs12705090 | -0.2361 | 0.0262 | T | C | 0.8109 | 2.17E-19 |
| rs12536419 | -0.6985 | 0.0285 | A | C | 0.1581 | ####### |
| rs1997571 | -0.1448 | 0.0208 | A | G | 0.409 | 3.46E-12 |
| rs11770163 | 0.1202 | 0.0217 | C | G | 0.6656 | 2.91E-08 |
| rs35680304 | 0.1848 | 0.021 | T | C | 0.407 | 1.60E-18 |
| rs141212865 | 0.1497 | 0.0263 | A | C | 0.1944 | 1.21E-08 |
| rs73727606 | 0.2532 | 0.0411 | A | G | 0.9286 | 6.99E-10 |
| rs73158180 | -0.1693 | 0.023 | A | C | 0.2966 | 1.76E-13 |
| rs10261098 | 0.168 | 0.0285 | T | C | 0.8458 | 3.95E-09 |
| rs2107595 | 0.4435 | 0.0282 | A | G | 0.8414 | 8.24E-56 |
| rs62449490 | 0.1137 | 0.0207 | T | G | 0.4588 | 3.72E-08 |
| rs2969036 | 0.1308 | 0.023 | T | G | 0.693 | 1.36E-08 |
| rs6461992 | -0.4361 | 0.04 | A | G | 0.9261 | 1.03E-27 |
| rs6961048 | -0.2668 | 0.0338 | C | G | 0.1036 | 2.67E-15 |
| rs977184 | -0.1947 | 0.0213 | T | C | 0.3741 | 6.86E-20 |
| rs17171688 | -0.3916 | 0.0509 | A | G | 0.9541 | 1.50E-14 |
| rs11977526 | -0.4308 | 0.0211 | A | G | 0.5982 | 1.75E-92 |
| rs2344402 | 0.1496 | 0.0214 | T | C | 0.4038 | 2.62E-12 |
| rs1091811 | -0.1745 | 0.0275 | A | G | 0.8313 | 2.28E-10 |
| rs848445 | -0.1309 | 0.023 | T | C | 0.7146 | 1.24E-08 |
| rs6951894 | -0.1416 | 0.0208 | A | G | 0.5757 | 8.85E-12 |
| rs42377 | -0.3175 | 0.0225 | A | G | 0.6956 | 2.42E-45 |
| rs13263821 | -0.1987 | 0.0265 | C | G | 0.8079 | 5.93E-14 |
| rs6601523 | -0.205 | 0.0208 | A | G | 0.4099 | 7.91E-23 |
| rs28499085 | 0.1569 | 0.023 | A | G | 0.2746 | 9.35E-12 |
| rs7341594 | 0.5069 | 0.0248 | A | G | 0.7797 | 5.56E-93 |
| rs13279275 | -0.1501 | 0.0252 | A | C | 0.2343 | 2.66E-09 |
| rs4440615 | -0.2492 | 0.0212 | A | G | 0.368 | 8.22E-32 |
| rs7011889 | 0.1167 | 0.0207 | A | C | 0.4454 | 1.64E-08 |
| rs7821832 | 0.2137 | 0.0236 | T | G | 0.2552 | 1.54E-19 |
| rs11988241 | -0.1323 | 0.0236 | T | C | 0.256 | 2.02E-08 |
| rs2953930 | 0.1712 | 0.0304 | T | C | 0.8676 | 1.76E-08 |
| rs10958683 | -0.1694 | 0.0248 | C | G | 0.2242 | 8.13E-12 |
| rs2978456 | -0.1781 | 0.0212 | T | C | 0.4487 | 5.14E-17 |
| rs4873492 | 0.2202 | 0.0273 | T | C | 0.8277 | 8.05E-16 |
| rs2354862 | 0.1272 | 0.0215 | A | C | 0.3597 | 3.11E-09 |
| rs1350100 | 0.1653 | 0.0208 | A | G | 0.5549 | 1.80E-15 |
| rs1449544 | 0.1927 | 0.0205 | A | C | 0.4565 | 6.68E-21 |
| rs16939351 | -0.29 | 0.0437 | A | G | 0.9406 | 3.34E-11 |
| rs4551303 | -0.1873 | 0.0221 | T | C | 0.6835 | 2.06E-17 |
| rs34917849 | 0.2846 | 0.0308 | C | G | 0.873 | 2.52E-20 |
| rs10988442 | 0.1809 | 0.0212 | A | G | 0.3801 | 1.38E-17 |
| rs7857437 | 0.3696 | 0.0591 | T | C | 0.9682 | 4.13E-10 |
| rs13290326 | -0.1562 | 0.0204 | T | C | 0.4988 | 2.11E-14 |
| rs7023208 | -0.1263 | 0.022 | C | G | 0.3266 | 9.19E-09 |
| rs2241003 | -0.1724 | 0.0212 | C | G | 0.367 | 4.00E-16 |
| rs7854147 | 0.2778 | 0.0314 | A | G | 0.1227 | 8.16E-19 |
| rs142378207 | 0.3473 | 0.0304 | A | G | 0.8689 | 3.50E-30 |
| rs3780190 | -0.1406 | 0.0208 | A | G | 0.5365 | 1.35E-11 |
| rs4977492 | -0.1252 | 0.0216 | T | C | 0.3379 | 6.70E-09 |
| rs4977575 | -0.1682 | 0.0206 | C | G | 0.4934 | 2.94E-16 |
| rs4553000 | -0.1464 | 0.0204 | T | C | 0.4861 | 7.47E-13 |
| rs34587684 | 0.1448 | 0.0254 | T | C | 0.7958 | 1.15E-08 |
| rs7853859 | 0.1212 | 0.0212 | T | C | 0.3671 | 1.11E-08 |

**Table S18. Genome-wide significant and independent SNPs that were used as instruments for BMI**

| SNP | effect | other | eaf | beta | se.exposure | pval.exposure |
| --- | --- | --- | --- | --- | --- | --- |
| rs10004685 | T | C | 0.1574 | -0.0137 | 0.002 | 9.60E-12 |
| rs10005035 | C | G | 0.7252 | 0.0244 | 0.0016 | 2.70E-52 |
| rs10010325 | A | C | 0.4926 | 0.0289 | 0.0014 | 6.80E-89 |
| rs10029732 | A | G | 0.522 | -0.0182 | 0.0014 | 3.80E-37 |
| rs10039254 | A | G | 0.6313 | -0.0123 | 0.0015 | 8.90E-17 |
| rs10040658 | A | G | 0.3716 | 0.0175 | 0.0015 | 1.40E-31 |
| rs10047435 | T | G | 0.6629 | 0.0144 | 0.0016 | 1.80E-20 |
| rs10049224 | T | G | 0.8158 | 0.0121 | 0.0018 | 4.80E-11 |
| rs10052957 | A | G | 0.3214 | 0.0136 | 0.0015 | 1.40E-18 |
| rs10082476 | A | G | 0.7613 | 0.0231 | 0.0017 | 2.20E-42 |
| rs10089933 | T | C | 0.7581 | -0.0236 | 0.0017 | 7.70E-46 |
| rs10096149 | T | C | 0.7231 | 0.0127 | 0.0016 | 1.80E-15 |
| rs10115038 | A | G | 0.7202 | -0.0106 | 0.0016 | 2.30E-11 |
| rs10119624 | A | G | 0.6698 | 0.0181 | 0.0015 | 3.10E-32 |
| rs10131337 | T | C | 0.2371 | 0.0212 | 0.0017 | 2.20E-34 |
| rs10140101 | T | C | 0.4458 | 0.0191 | 0.0014 | 5.30E-40 |
| rs1014719 | T | G | 0.5591 | 0.0281 | 0.0014 | 1.50E-86 |
| rs10151805 | T | C | 0.752 | -0.0133 | 0.0017 | 2.70E-15 |
| rs10151995 | C | G | 0.08507 | -0.0173 | 0.0027 | 6.30E-11 |
| rs1018800 | A | T | 0.6386 | -0.0193 | 0.0015 | 4.40E-37 |
| rs1023522 | A | G | 0.523 | -0.0273 | 0.0014 | 6.60E-81 |
| rs10283100 | A | G | 0.05835 | -0.0474 | 0.0033 | 1.80E-46 |
| rs1028580 | T | C | 0.8524 | -0.012 | 0.002 | 4.20E-09 |
| rs10419247 | T | G | 0.03379 | -0.0292 | 0.0039 | 8.00E-14 |
| rs10422762 | A | G | 0.4767 | 0.0116 | 0.0015 | 1.10E-15 |
| rs10431126 | C | G | 0.7228 | 0.0131 | 0.0016 | 2.70E-16 |
| rs1043542 | A | G | 0.3851 | 0.0163 | 0.0016 | 6.70E-26 |
| rs10437112 | A | G | 0.5405 | 0.0106 | 0.0014 | 1.10E-13 |
| rs1044145 | T | C | 0.4447 | 0.0113 | 0.0014 | 4.00E-15 |
| rs1044670 | A | G | 0.148 | -0.0165 | 0.002 | 1.60E-16 |
| rs10459573 | A | C | 0.634 | 0.0264 | 0.0015 | 4.20E-70 |
| rs1046310 | T | G | 0.5478 | -0.0163 | 0.0015 | 8.20E-29 |
| rs1046934 | A | C | 0.6575 | -0.0452 | 0.0015 | ###### |
| rs1047014 | T | C | 0.7439 | -0.0312 | 0.0017 | 3.10E-77 |
| rs1047255 | C | G | 0.765 | 0.021 | 0.0017 | 3.10E-37 |
| rs10483727 | T | C | 0.3975 | 0.0384 | 0.0015 | ###### |
| rs1049212 | A | G | 0.44 | 0.0171 | 0.0015 | 4.90E-32 |
| rs10492237 | T | C | 0.90482 | 0.0317 | 0.0025 | 2.50E-38 |
| rs10493166 | A | C | 0.1195 | 0.016 | 0.0022 | 1.40E-13 |
| rs10495565 | A | G | 0.6898 | -0.0155 | 0.0015 | 8.50E-24 |
| rs10497854 | A | C | 0.03572 | 0.0251 | 0.0039 | 1.10E-10 |
| rs10501140 | T | C | 0.308 | -0.0128 | 0.0016 | 3.80E-16 |
| rs1050190 | T | G | 0.2798 | -0.0126 | 0.0016 | 5.50E-15 |
| rs10502235 | A | G | 0.84 | 0.0242 | 0.0019 | 1.10E-35 |
| rs10502974 | A | C | 0.5555 | 0.0103 | 0.0014 | 1.50E-12 |
| rs10504649 | A | G | 0.719 | -0.0374 | 0.0016 | ###### |
| rs1050969 | T | C | 0.98147 | -0.0422 | 0.006 | 2.00E-12 |
| rs1051547 | T | C | 0.4406 | 0.0114 | 0.0015 | 7.40E-15 |
| rs10517658 | A | G | 0.08489 | -0.0224 | 0.0026 | 2.50E-17 |
| rs10518271 | T | C | 0.4557 | -0.0092 | 0.0015 | 2.20E-10 |
| rs10519731 | A | G | 0.8456 | -0.0153 | 0.002 | 2.90E-14 |
| rs10520585 | C | G | 0.7606 | -0.0193 | 0.0017 | 1.00E-29 |
| rs1054249 | A | G | 0.1835 | 0.0119 | 0.0019 | 1.90E-10 |
| rs1062557 | A | C | 0.7441 | 0.0098 | 0.0017 | 4.20E-09 |
| rs10746732 | A | G | 0.7735 | -0.0226 | 0.0018 | 4.60E-37 |
| rs10767967 | T | G | 0.4229 | -0.0085 | 0.0015 | 4.70E-09 |
| rs10770705 | A | C | 0.3281 | 0.0231 | 0.0015 | 1.30E-52 |
| rs10789509 | T | C | 0.7103 | -0.0124 | 0.0016 | 1.60E-15 |
| rs10799445 | A | C | 0.7757 | 0.0334 | 0.0017 | 1.00E-88 |
| rs10804564 | A | G | 0.5862 | 0.0081 | 0.0014 | 2.30E-08 |
| rs10811869 | T | C | 0.3515 | -0.0102 | 0.0015 | 1.60E-11 |
| rs10823149 | T | G | 0.5403 | 0.023 | 0.0015 | 1.20E-56 |
| rs10838738 | A | G | 0.6462 | 0.0291 | 0.0015 | 3.10E-84 |
| rs10843178 | C | G | 0.3154 | -0.0393 | 0.0015 | ###### |
| rs10843390 | T | C | 0.2753 | 0.0214 | 0.0016 | 3.60E-41 |
| rs10846654 | A | G | 0.6452 | -0.0273 | 0.0015 | 1.10E-72 |
| rs10853981 | A | G | 0.34 | -0.0242 | 0.0015 | 3.50E-55 |
| rs10858868 | C | G | 0.4825 | -0.0096 | 0.0014 | 1.90E-11 |
| rs10859597 | T | C | 0.7993 | -0.0132 | 0.0018 | 2.00E-13 |
| rs10861678 | A | G | 0.2694 | -0.02 | 0.0016 | 1.00E-34 |
| rs10863210 | A | G | 0.0466 | -0.024 | 0.0037 | 8.00E-11 |
| rs10866910 | T | C | 0.7313 | -0.016 | 0.0016 | 8.30E-23 |
| rs10868439 | A | G | 0.4721 | -0.0232 | 0.0014 | 6.40E-58 |
| rs10874746 | T | C | 0.3354 | -0.021 | 0.0015 | 9.30E-46 |
| rs10888577 | T | C | 0.7178 | 0.0129 | 0.0016 | 6.60E-16 |
| rs10890154 | T | C | 0.5419 | -0.0093 | 0.0014 | 9.20E-11 |
| rs10906041 | T | C | 0.8703 | 0.02 | 0.0021 | 2.20E-21 |
| rs10916617 | T | C | 0.2177 | -0.0221 | 0.0017 | 1.20E-37 |
| rs10931818 | T | G | 0.2816 | 0.0089 | 0.0016 | 2.10E-08 |
| rs10936579 | C | G | 0.1143 | 0.0181 | 0.0022 | 2.50E-16 |
| rs10948007 | T | C | 0.07758 | 0.0246 | 0.0026 | 2.80E-21 |
| rs10949593 | T | C | 0.3342 | 0.0098 | 0.0015 | 9.70E-11 |
| rs10963680 | A | G | 0.2444 | -0.0169 | 0.0017 | 3.40E-23 |
| rs10973733 | A | G | 0.2413 | -0.0094 | 0.0017 | 3.30E-08 |
| rs10979739 | T | C | 0.7034 | 0.0111 | 0.0016 | 1.70E-12 |
| rs10995319 | T | C | 0.7709 | 0.0192 | 0.0017 | 6.50E-30 |
| rs10997909 | T | G | 0.7332 | -0.0168 | 0.0016 | 6.30E-25 |
| rs10998347 | A | G | 0.411 | 0.0212 | 0.0015 | 6.00E-47 |
| rs11001272 | A | G | 0.676 | -0.0096 | 0.0015 | 2.40E-10 |
| rs11008865 | T | C | 0.1675 | 0.0114 | 0.0019 | 2.60E-09 |
| rs11014285 | A | G | 0.1651 | 0.024 | 0.0021 | 1.60E-31 |
| rs11014382 | T | C | 0.352 | 0.0113 | 0.0015 | 7.90E-14 |
| rs11022538 | A | T | 0.05736 | -0.0182 | 0.0031 | 3.60E-09 |
| rs11055989 | A | G | 0.678 | -0.0105 | 0.0016 | 1.40E-11 |
| rs11061710 | T | C | 0.9399 | 0.0233 | 0.0031 | 4.30E-14 |
| rs11065286 | T | C | 0.4351 | 0.0157 | 0.0014 | 6.50E-28 |
| rs11070641 | T | C | 0.778 | 0.0202 | 0.0017 | 1.50E-31 |
| rs11075053 | T | G | 0.7364 | -0.0147 | 0.0017 | 8.80E-19 |
| rs11077395 | T | C | 0.1784 | 0.0144 | 0.0019 | 2.00E-14 |
| rs11087321 | C | G | 0.7559 | 0.0096 | 0.0017 | 2.00E-08 |
| rs11104829 | A | G | 0.7132 | 0.0148 | 0.0016 | 1.70E-20 |
| rs11107154 | T | C | 0.7097 | 0.0303 | 0.0016 | 6.80E-83 |
| rs11122824 | A | T | 0.1919 | -0.0175 | 0.0019 | 5.10E-21 |
| rs11130252 | A | G | 0.1303 | 0.0322 | 0.0022 | 3.00E-50 |
| rs11133373 | C | G | 0.6837 | -0.0139 | 0.0016 | 1.50E-18 |
| rs11134683 | T | C | 0.6539 | 0.0124 | 0.0015 | 7.30E-16 |
| rs11144688 | A | G | 0.1238 | -0.05 | 0.0023 | ###### |
| rs11159706 | T | G | 0.3003 | 0.0093 | 0.0016 | 3.70E-09 |
| rs11161617 | T | C | 0.7715 | 0.0146 | 0.0017 | 4.90E-18 |
| rs11164653 | T | C | 0.4074 | 0.0245 | 0.0015 | 8.70E-64 |
| rs11198898 | A | C | 0.233 | 0.029 | 0.0017 | 5.80E-64 |
| rs11204272 | T | C | 0.02526 | -0.0311 | 0.0047 | 2.70E-11 |
| rs11205303 | T | C | 0.5918 | -0.049 | 0.0015 | ###### |
| rs11209745 | A | G | 0.7361 | -0.0148 | 0.0016 | 2.50E-20 |
| rs11233661 | A | G | 0.6225 | 0.0144 | 0.0015 | 7.00E-22 |
| rs11245333 | A | C | 0.4102 | 0.0206 | 0.0015 | 1.50E-44 |
| rs11247362 | T | C | 0.8963 | 0.0528 | 0.0024 | ###### |
| rs1126309 | A | G | 0.4978 | -0.0081 | 0.0014 | 1.70E-08 |
| rs11582932 | T | G | 0.6463 | 0.0105 | 0.0015 | 1.10E-12 |
| rs11599252 | T | C | 0.08811 | 0.0245 | 0.0026 | 6.90E-22 |
| rs11607537 | T | C | 0.05256 | 0.0341 | 0.0031 | 8.00E-28 |
| rs11612228 | T | C | 0.3497 | 0.0262 | 0.0015 | 1.50E-65 |
| rs11616390 | T | C | 0.90242 | -0.0205 | 0.0025 | 1.10E-16 |
| rs11640223 | T | G | 0.2103 | -0.0161 | 0.0018 | 9.40E-20 |
| rs11642740 | A | G | 0.6023 | -0.0201 | 0.0015 | 2.50E-42 |
| rs11654523 | T | C | 0.9282 | 0.035 | 0.0032 | 3.60E-28 |
| rs11658228 | T | C | 0.2508 | -0.0124 | 0.0017 | 2.20E-13 |
| rs11661645 | A | G | 0.306 | 0.0163 | 0.0016 | 1.10E-24 |
| rs11664 | A | G | 0.2787 | -0.013 | 0.0016 | 2.50E-15 |
| rs11664336 | A | T | 0.5661 | -0.0361 | 0.0015 | ###### |
| rs1167084 | A | G | 0.6852 | 0.0097 | 0.0015 | 2.60E-10 |
| rs11679414 | A | G | 0.3112 | 0.0097 | 0.0015 | 2.40E-10 |
| rs11681299 | T | C | 0.2798 | 0.0303 | 0.0016 | 7.10E-84 |
| rs1169093 | T | C | 0.1301 | 0.0155 | 0.0022 | 5.40E-13 |
| rs11698420 | T | G | 0.2519 | 0.0125 | 0.0017 | 4.40E-14 |
| rs11708067 | A | G | 0.7681 | 0.0169 | 0.0017 | 9.90E-24 |
| rs11722554 | A | G | 0.03274 | -0.0496 | 0.0039 | 3.30E-36 |
| rs1173727 | T | C | 0.4035 | 0.0368 | 0.0015 | ###### |
| rs11743919 | T | C | 0.2116 | -0.0165 | 0.0017 | 1.70E-21 |
| rs1179726 | A | G | 0.5441 | -0.0095 | 0.0014 | 2.70E-11 |
| rs1180342 | T | G | 0.3661 | 0.0087 | 0.0015 | 1.20E-08 |
| rs11873192 | A | G | 0.7967 | 0.0103 | 0.0018 | 2.00E-08 |
| rs11903298 | A | G | 0.90622 | 0.0259 | 0.0027 | 4.20E-22 |
| rs11924583 | A | G | 0.681 | -0.0095 | 0.0016 | 8.20E-10 |
| rs11940262 | A | G | 0.3704 | 0.0112 | 0.0015 | 4.10E-14 |
| rs11944380 | T | C | 0.1377 | -0.0174 | 0.0021 | 2.70E-16 |
| rs11960388 | A | T | 0.1269 | 0.0122 | 0.0022 | 3.40E-08 |
| rs1199735 | C | G | 0.8831 | 0.0293 | 0.0023 | 7.40E-38 |
| rs12048049 | C | G | 0.7154 | -0.0368 | 0.0016 | ###### |
| rs12130243 | T | G | 0.802 | 0.0228 | 0.0017 | 4.50E-39 |
| rs12136856 | C | G | 0.3347 | 0.0122 | 0.0015 | 3.50E-16 |
| rs12145922 | A | C | 0.5556 | 0.0266 | 0.0014 | 5.70E-77 |
| rs12187790 | A | G | 0.8075 | 0.01 | 0.0018 | 4.70E-08 |
| rs12188208 | A | C | 0.776 | 0.0243 | 0.0017 | 2.90E-46 |
| rs12198115 | T | G | 0.7199 | -0.0118 | 0.0016 | 2.90E-13 |
| rs12206717 | A | G | 0.05513 | -0.0427 | 0.0031 | 2.80E-43 |
| rs12211255 | A | C | 0.105 | 0.0428 | 0.0023 | 1.30E-74 |
| rs12312144 | A | C | 0.1814 | 0.0136 | 0.0019 | 6.20E-13 |
| rs12322888 | T | C | 0.2032 | 0.0354 | 0.0018 | 7.40E-89 |
| rs12352385 | A | C | 0.8569 | 0.014 | 0.0021 | 2.50E-11 |
| rs12358995 | T | C | 0.1946 | -0.0113 | 0.0019 | 1.20E-09 |
| rs12362444 | A | G | 0.5455 | -0.0084 | 0.0014 | 6.40E-09 |
| rs12368672 | C | G | 0.6002 | 0.0095 | 0.0015 | 1.00E-10 |
| rs12385976 | T | C | 0.9617 | 0.0949 | 0.0036 | ###### |
| rs12410416 | T | C | 0.7878 | -0.0449 | 0.0017 | ###### |
| rs12419284 | T | C | 0.07905 | -0.017 | 0.0027 | 4.50E-10 |
| rs12450371 | T | C | 0.3485 | 0.0165 | 0.0015 | 6.30E-27 |
| rs12452415 | A | G | 0.5997 | 0.0118 | 0.0015 | 7.40E-16 |
| rs12477144 | C | G | 0.3123 | 0.0122 | 0.0015 | 2.40E-15 |
| rs12513126 | A | T | 0.4651 | -0.0093 | 0.0015 | 1.90E-10 |
| rs12513181 | A | C | 0.7412 | -0.019 | 0.0016 | 5.10E-31 |
| rs12519649 | T | C | 0.8746 | 0.0172 | 0.0022 | 1.20E-15 |
| rs12538407 | A | G | 0.5926 | 0.0301 | 0.0015 | 1.20E-94 |
| rs12543983 | A | T | 0.8579 | -0.0139 | 0.002 | 9.60E-12 |
| rs12546802 | A | T | 0.2549 | -0.0118 | 0.0017 | 7.90E-13 |
| rs12553641 | A | G | 0.2726 | 0.0095 | 0.0017 | 1.00E-08 |
| rs1255529 | A | G | 0.4381 | 0.0102 | 0.0015 | 4.50E-12 |
| rs1256640 | T | C | 0.1084 | 0.0148 | 0.0024 | 3.70E-10 |
| rs12572775 | A | T | 0.4422 | -0.0287 | 0.0014 | 2.40E-88 |
| rs12620067 | T | C | 0.8574 | 0.0152 | 0.002 | 4.20E-14 |
| rs12635129 | A | T | 0.7698 | 0.016 | 0.0017 | 5.10E-20 |
| rs12669267 | T | C | 0.1302 | -0.0261 | 0.0022 | 1.90E-31 |
| rs12698831 | A | G | 0.1551 | -0.0176 | 0.002 | 3.50E-19 |
| rs12699924 | A | G | 0.51 | 0.0114 | 0.0014 | 2.30E-15 |
| rs12743493 | A | G | 0.3761 | 0.0139 | 0.0016 | 4.90E-19 |
| rs12755321 | T | G | 0.8844 | -0.0145 | 0.0022 | 3.40E-11 |
| rs12775504 | A | C | 0.7981 | 0.0132 | 0.0019 | 2.00E-12 |
| rs12779328 | T | C | 0.2833 | -0.0301 | 0.0016 | 6.60E-79 |
| rs1279683 | A | G | 0.3369 | -0.0092 | 0.0016 | 1.20E-08 |
| rs1281962 | C | G | 0.5251 | -0.0086 | 0.0014 | 1.40E-09 |
| rs12826630 | T | C | 0.2008 | 0.0129 | 0.0018 | 4.30E-13 |
| rs12855 | T | C | 0.09255 | 0.0436 | 0.0025 | 1.40E-68 |
| rs12893199 | T | C | 0.3741 | 0.0123 | 0.0015 | 6.40E-16 |
| rs12899214 | A | G | 0.4507 | 0.0088 | 0.0014 | 8.90E-10 |
| rs12902421 | T | C | 0.98012 | -0.0706 | 0.0052 | 3.10E-42 |
| rs12914042 | C | G | 0.413 | 0.0137 | 0.0015 | 1.20E-20 |
| rs12924101 | A | C | 0.83 | 0.0199 | 0.0019 | 1.20E-25 |
| rs12931841 | A | G | 0.6413 | -0.0113 | 0.0015 | 4.20E-14 |
| rs12975319 | A | G | 0.3044 | 0.0226 | 0.0016 | 2.30E-45 |
| rs12982656 | T | C | 0.2285 | 0.0213 | 0.0017 | 1.50E-34 |
| rs12982744 | C | G | 0.6071 | -0.0351 | 0.0015 | ###### |
| rs12996345 | A | T | 0.8521 | -0.0192 | 0.002 | 2.90E-21 |
| rs12996789 | C | G | 0.9634 | 0.0323 | 0.0039 | 2.30E-16 |
| rs13026081 | T | C | 0.3224 | 0.0096 | 0.0015 | 5.60E-10 |
| rs13043313 | T | C | 0.7145 | 0.0175 | 0.0016 | 4.60E-28 |
| rs13120076 | A | T | 0.2499 | -0.0134 | 0.0017 | 7.00E-16 |
| rs13209835 | T | G | 0.3562 | -0.0135 | 0.0015 | 4.50E-20 |
| rs1320998 | A | G | 0.449 | 0.0081 | 0.0014 | 1.60E-08 |
| rs13250995 | A | G | 0.1673 | -0.0141 | 0.0019 | 2.00E-13 |
| rs13257899 | A | T | 0.6617 | -0.0152 | 0.0015 | 9.30E-23 |
| rs1326384 | T | C | 0.4885 | 0.0085 | 0.0015 | 7.80E-09 |
| rs13282289 | A | G | 0.7701 | -0.016 | 0.0017 | 1.80E-20 |
| rs13290190 | A | G | 0.7153 | 0.0265 | 0.0016 | 4.90E-62 |
| rs134040 | A | G | 0.5356 | 0.0127 | 0.0015 | 8.60E-18 |
| rs13406323 | A | G | 0.4363 | -0.0086 | 0.0015 | 5.80E-09 |
| rs13436031 | A | C | 0.2344 | -0.0136 | 0.0017 | 1.10E-15 |
| rs1346794 | C | G | 0.7777 | 0.059 | 0.0018 | ###### |
| rs1351438 | A | C | 0.1998 | 0.0155 | 0.0018 | 1.50E-17 |
| rs136029 | A | G | 0.418 | 0.0168 | 0.0015 | 7.40E-30 |
| rs1366594 | A | C | 0.5388 | 0.0277 | 0.0014 | 1.30E-82 |
| rs13675 | T | C | 0.7947 | -0.0296 | 0.0018 | 4.10E-63 |
| rs1371325 | A | G | 0.4022 | 0.0096 | 0.0015 | 8.40E-11 |
| rs13835 | A | C | 0.4305 | -0.0134 | 0.0015 | 3.40E-20 |
| rs1389371 | T | C | 0.6263 | 0.0106 | 0.0015 | 5.40E-13 |
| rs1393786 | A | C | 0.2743 | -0.0293 | 0.0016 | 8.90E-74 |
| rs1403987 | A | G | 0.3469 | -0.0222 | 0.0015 | 3.50E-49 |
| rs1418201 | A | T | 0.7074 | 0.0544 | 0.0016 | ###### |
| rs1430157 | T | C | 0.3171 | 0.0139 | 0.0015 | 2.40E-19 |
| rs1467847 | C | G | 0.5681 | 0.0221 | 0.0015 | 1.30E-50 |
| rs1483831 | A | G | 0.2277 | 0.014 | 0.0017 | 9.10E-17 |
| rs1490271 | A | C | 0.1273 | -0.0201 | 0.0022 | 5.70E-20 |
| rs1490384 | T | C | 0.493 | 0.0436 | 0.0014 | ###### |
| rs1497411 | T | C | 0.04987 | -0.0276 | 0.0033 | 2.90E-17 |
| rs14976 | T | C | 0.2911 | 0.0169 | 0.0015 | 9.20E-28 |
| rs1512102 | A | G | 0.7354 | -0.0114 | 0.0016 | 1.80E-12 |
| rs1517397 | T | C | 0.4043 | -0.0085 | 0.0015 | 6.40E-09 |
| rs1533269 | A | C | 0.3107 | -0.0176 | 0.0016 | 3.10E-29 |
| rs1539733 | T | C | 0.2661 | 0.0098 | 0.0016 | 1.30E-09 |
| rs1544144 | T | C | 0.4739 | 0.0172 | 0.0014 | 1.50E-33 |
| rs1556052 | A | G | 0.4363 | -0.0158 | 0.0015 | 2.30E-27 |
| rs1561819 | A | G | 0.492 | 0.0146 | 0.0014 | 6.60E-24 |
| rs1572416 | A | C | 0.1748 | -0.0119 | 0.0018 | 1.30E-10 |
| rs1574220 | T | C | 0.91967 | -0.0286 | 0.0028 | 2.60E-25 |
| rs157845 | T | C | 0.2591 | 0.0112 | 0.0016 | 9.80E-12 |
| rs1581130 | A | G | 0.554 | -0.0177 | 0.0015 | 5.00E-34 |
| rs1584239 | T | C | 0.8516 | -0.0205 | 0.002 | 8.10E-25 |
| rs1599473 | T | G | 0.2477 | -0.0255 | 0.0017 | 2.90E-52 |
| rs1624841 | T | C | 0.1949 | 0.0125 | 0.0019 | 1.50E-11 |
| rs1638038 | C | G | 0.3632 | 0.018 | 0.0015 | 6.00E-32 |
| rs1653024 | T | C | 0.1668 | 0.016 | 0.0019 | 2.00E-16 |
| rs16824599 | A | G | 0.1181 | -0.0243 | 0.0022 | 1.10E-27 |
| rs16833107 | T | C | 0.1185 | -0.0263 | 0.0023 | 1.80E-31 |
| rs16844364 | A | G | 0.2063 | -0.0177 | 0.0018 | 9.90E-22 |
| rs16887114 | A | T | 0.93499 | -0.0169 | 0.0028 | 1.50E-09 |
| rs16918178 | T | C | 0.96808 | -0.0237 | 0.0042 | 1.40E-08 |
| rs16925042 | A | T | 0.8704 | -0.0209 | 0.0021 | 1.40E-22 |
| rs16926774 | T | C | 0.3088 | 0.0178 | 0.0016 | 8.60E-30 |
| rs16930370 | T | C | 0.8198 | -0.0172 | 0.0019 | 1.50E-19 |
| rs16977018 | T | G | 0.7325 | -0.0207 | 0.0017 | 8.40E-36 |
| rs16978236 | A | G | 0.8904 | 0.0141 | 0.0023 | 8.70E-10 |
| rs16987211 | T | C | 0.92551 | 0.029 | 0.0027 | 4.40E-26 |
| rs17007958 | C | G | 0.8355 | 0.0166 | 0.002 | 4.10E-16 |
| rs17039434 | C | G | 0.2004 | -0.0178 | 0.0018 | 3.10E-23 |
| rs17085675 | A | T | 0.712 | -0.0189 | 0.0016 | 1.60E-32 |
| rs17117836 | T | C | 0.1027 | -0.0146 | 0.0024 | 9.80E-10 |
| rs17135027 | A | G | 0.06034 | -0.0218 | 0.0031 | 3.40E-12 |
| rs17143141 | A | G | 0.1602 | 0.0341 | 0.002 | 4.20E-68 |
| rs17148514 | T | C | 0.2406 | -0.0176 | 0.0017 | 1.20E-24 |
| rs17172639 | A | G | 0.115 | 0.031 | 0.0024 | 1.40E-39 |
| rs17179740 | A | G | 0.4168 | -0.0103 | 0.0015 | 3.80E-12 |
| rs1718067 | T | C | 0.6811 | 0.0092 | 0.0015 | 2.30E-09 |
| rs17197086 | T | C | 0.1231 | 0.0303 | 0.0022 | 1.80E-42 |
| rs17197938 | T | C | 0.2418 | -0.0152 | 0.0017 | 1.80E-19 |
| rs172179 | C | G | 0.5914 | -0.0134 | 0.0015 | 2.00E-19 |
| rs17221614 | A | T | 0.08893 | -0.0309 | 0.0026 | 8.60E-33 |
| rs17222916 | A | C | 0.839 | -0.0227 | 0.002 | 7.90E-31 |
| rs17230678 | A | G | 0.06209 | -0.0157 | 0.0029 | 3.90E-08 |
| rs17318596 | A | G | 0.3603 | 0.0291 | 0.0015 | 1.20E-81 |
| rs17329188 | A | G | 0.7295 | 0.0113 | 0.0016 | 4.40E-12 |
| rs17335495 | C | G | 0.97889 | 0.04 | 0.0053 | 5.30E-14 |
| rs17339199 | T | C | 0.06045 | 0.0201 | 0.0033 | 5.80E-10 |
| rs17340572 | A | C | 0.8006 | -0.0119 | 0.0017 | 1.10E-11 |
| rs17357954 | T | G | 0.2227 | 0.0398 | 0.0017 | ###### |
| rs17369123 | T | C | 0.1819 | 0.0269 | 0.0018 | 1.60E-49 |
| rs17380639 | T | C | 0.05361 | -0.0276 | 0.0032 | 5.10E-18 |
| rs17391694 | T | C | 0.1189 | 0.0343 | 0.0021 | 1.30E-57 |
| rs17400325 | T | C | 0.96194 | -0.0502 | 0.0035 | 4.20E-46 |
| rs17409588 | T | C | 0.6825 | -0.0164 | 0.0015 | 3.70E-26 |
| rs17419937 | A | G | 0.8205 | -0.011 | 0.0019 | 6.10E-09 |
| rs17428810 | T | C | 0.6881 | 0.0102 | 0.0015 | 4.70E-11 |
| rs174383 | A | G | 0.7482 | -0.0102 | 0.0016 | 7.30E-10 |
| rs1744759 | C | G | 0.8535 | 0.0324 | 0.002 | 1.40E-58 |
| rs17467825 | A | G | 0.7145 | 0.0104 | 0.0016 | 5.30E-11 |
| rs17472200 | C | G | 0.7183 | 0.0181 | 0.0016 | 1.30E-29 |
| rs17475053 | C | G | 0.7211 | -0.0234 | 0.0016 | 1.70E-47 |
| rs17497947 | T | G | 0.2001 | -0.02 | 0.0017 | 2.00E-30 |
| rs17567417 | C | G | 0.4749 | 0.0196 | 0.0014 | 4.40E-43 |
| rs17583662 | A | G | 0.6252 | -0.0193 | 0.0015 | 9.60E-39 |
| rs17700154 | A | G | 0.03128 | 0.0254 | 0.0045 | 1.50E-08 |
| rs17714469 | A | G | 0.1119 | 0.0187 | 0.0022 | 9.80E-17 |
| rs17728834 | A | T | 0.7628 | -0.0137 | 0.0017 | 1.00E-15 |
| rs17731432 | T | G | 0.08899 | -0.0273 | 0.0026 | 2.80E-26 |
| rs17742342 | A | C | 0.8033 | -0.013 | 0.0018 | 2.60E-13 |
| rs17757552 | A | C | 0.92253 | -0.0168 | 0.0027 | 5.20E-10 |
| rs17770213 | T | C | 0.5585 | -0.0153 | 0.0014 | 2.50E-26 |
| rs1777220 | T | G | 0.3901 | -0.0094 | 0.0015 | 1.50E-10 |
| rs17780143 | A | G | 0.0532 | 0.0223 | 0.0032 | 2.80E-12 |
| rs17787912 | A | G | 0.7993 | 0.0101 | 0.0018 | 2.90E-08 |
| rs17813106 | A | C | 0.92148 | -0.0214 | 0.0027 | 1.60E-15 |
| rs1786263 | T | G | 0.6111 | -0.015 | 0.0015 | 3.50E-24 |
| rs178880 | T | C | 0.7661 | -0.0108 | 0.0018 | 1.50E-09 |
| rs1799908 | A | T | 0.4073 | -0.0176 | 0.0015 | 6.30E-34 |
| rs1802 | T | C | 0.681 | 0.0178 | 0.0015 | 9.90E-32 |
| rs1815314 | A | G | 0.4161 | -0.0244 | 0.0015 | 1.00E-62 |
| rs184304 | T | C | 0.128 | -0.0183 | 0.0021 | 1.50E-17 |
| rs1849336 | T | C | 0.7248 | -0.0125 | 0.0016 | 3.30E-15 |
| rs1864828 | A | T | 0.1532 | 0.0136 | 0.0019 | 2.90E-12 |
| rs1866750 | T | C | 0.7755 | 0.0136 | 0.0018 | 8.70E-15 |
| rs1874259 | T | C | 0.5082 | 0.0354 | 0.0014 | ###### |
| rs1885243 | T | C | 0.0863 | 0.0326 | 0.0026 | 9.10E-37 |
| rs1927639 | C | G | 0.6206 | -0.0134 | 0.0015 | 1.70E-19 |
| rs1950500 | T | C | 0.2907 | 0.0287 | 0.0016 | 4.00E-73 |
| rs1961460 | A | G | 0.3181 | 0.0459 | 0.0016 | ###### |
| rs1966265 | A | G | 0.2348 | 0.0453 | 0.0017 | ###### |
| rs1978600 | T | C | 0.06513 | -0.0437 | 0.0033 | 6.40E-40 |
| rs1989789 | C | G | 0.3293 | 0.0098 | 0.0015 | 2.10E-10 |
| rs1991083 | T | C | 0.6811 | 0.0299 | 0.0015 | 2.90E-83 |
| rs2008592 | A | G | 0.92359 | 0.0162 | 0.0028 | 4.10E-09 |
| rs2013265 | T | C | 0.253 | -0.0288 | 0.0016 | 2.40E-68 |
| rs2016755 | A | G | 0.5632 | -0.0125 | 0.0015 | 1.20E-17 |
| rs201762 | T | G | 0.7718 | 0.0527 | 0.0017 | ###### |
| rs2020383 | T | C | 0.5381 | -0.0159 | 0.0014 | 3.60E-28 |
| rs2022187 | T | C | 0.1318 | 0.0131 | 0.0021 | 2.70E-10 |
| rs2025151 | C | G | 0.8029 | -0.0466 | 0.0018 | ###### |
| rs2037341 | A | G | 0.4155 | -0.0083 | 0.0015 | 8.90E-09 |
| rs2046158 | T | C | 0.1711 | -0.0166 | 0.0019 | 4.10E-18 |
| rs2047137 | T | C | 0.3663 | -0.0086 | 0.0015 | 1.10E-08 |
| rs2070776 | A | G | 0.3391 | -0.0427 | 0.0015 | ###### |
| rs2072268 | A | G | 0.4766 | -0.0152 | 0.0015 | 1.90E-25 |
| rs2072346 | T | C | 0.8088 | 0.0235 | 0.0019 | 3.20E-36 |
| rs2089983 | T | C | 0.6384 | -0.0193 | 0.0015 | 2.70E-38 |
| rs2112798 | T | C | 0.1866 | -0.0141 | 0.002 | 7.00E-13 |
| rs2131371 | A | C | 0.3167 | -0.0236 | 0.0016 | 1.70E-52 |
| rs2133952 | T | C | 0.3761 | -0.0151 | 0.0015 | 1.70E-24 |
| rs2148031 | T | G | 0.5652 | 0.01 | 0.0014 | 5.70E-12 |
| rs2151284 | C | G | 0.3479 | 0.0098 | 0.0015 | 2.30E-10 |
| rs2152464 | A | C | 0.6103 | -0.0109 | 0.0015 | 2.00E-13 |
| rs2166898 | A | G | 0.1643 | -0.0303 | 0.0019 | 1.80E-56 |
| rs217181 | T | C | 0.2076 | 0.0193 | 0.0018 | 7.70E-26 |
| rs2181343 | A | C | 0.3681 | -0.0164 | 0.0015 | 4.90E-27 |
| rs2181834 | T | G | 0.5575 | 0.0214 | 0.0014 | 7.90E-50 |
| rs2203715 | T | C | 0.7565 | -0.0171 | 0.0016 | 6.50E-26 |
| rs221068 | A | G | 0.448 | 0.0214 | 0.0015 | 5.30E-49 |
| rs2215047 | A | G | 0.7484 | 0.0138 | 0.0017 | 7.50E-17 |
| rs2215151 | T | G | 0.266 | 0.0115 | 0.0017 | 4.60E-12 |
| rs2216999 | A | T | 0.46 | 0.0143 | 0.0015 | 1.00E-22 |
| rs2218378 | A | G | 0.6247 | -0.0144 | 0.0015 | 2.80E-22 |
| rs2219320 | T | C | 0.7423 | 0.0255 | 0.0016 | 2.40E-54 |
| rs2227901 | A | G | 0.1936 | 0.0337 | 0.0018 | 2.90E-75 |
| rs2230033 | A | G | 0.5595 | -0.0235 | 0.0015 | 2.20E-58 |
| rs2237886 | T | C | 0.1049 | 0.0476 | 0.0024 | 4.40E-90 |
| rs2239560 | A | G | 0.1511 | 0.0269 | 0.002 | 1.00E-39 |
| rs224292 | A | G | 0.3525 | 0.0128 | 0.0015 | 1.50E-17 |
| rs2245698 | T | G | 0.3749 | 0.0174 | 0.0015 | 1.80E-31 |
| rs2246410 | A | G | 0.2743 | -0.0149 | 0.0017 | 6.90E-19 |
| rs2247341 | A | G | 0.3399 | 0.0262 | 0.0015 | 3.90E-67 |
| rs2255083 | A | C | 0.06139 | -0.0192 | 0.003 | 2.00E-10 |
| rs2272023 | A | C | 0.7511 | -0.0342 | 0.0017 | 3.80E-91 |
| rs2273145 | T | C | 0.2184 | 0.037 | 0.0017 | 1.60E-99 |
| rs2273608 | T | C | 0.09618 | 0.0247 | 0.0025 | 1.80E-22 |
| rs2277138 | T | C | 0.6042 | 0.0352 | 0.0015 | ###### |
| rs227723 | T | C | 0.3072 | 0.0262 | 0.0016 | 4.00E-63 |
| rs2279008 | T | C | 0.7461 | 0.0245 | 0.0017 | 1.10E-47 |
| rs228289 | T | G | 0.7642 | 0.0229 | 0.0017 | 1.40E-40 |
| rs2284746 | C | G | 0.4762 | -0.0398 | 0.0014 | ###### |
| rs2287499 | C | G | 0.885 | 0.0167 | 0.0023 | 9.70E-13 |
| rs2289195 | A | G | 0.4149 | 0.0375 | 0.0014 | ###### |
| rs2295080 | T | G | 0.6958 | -0.0183 | 0.0016 | 1.90E-31 |
| rs2295124 | A | G | 0.2703 | -0.0219 | 0.0017 | 2.00E-39 |
| rs2296316 | T | C | 0.542 | 0.0247 | 0.0015 | 5.50E-64 |
| rs2302580 | T | C | 0.4157 | -0.0243 | 0.0015 | 3.50E-57 |
| rs2303262 | T | C | 0.781 | -0.0229 | 0.0018 | 3.70E-38 |
| rs2303745 | T | G | 0.8347 | -0.0118 | 0.002 | 2.10E-09 |
| rs2304376 | A | G | 0.7644 | -0.0163 | 0.0017 | 2.80E-22 |
| rs2306596 | A | C | 0.5264 | 0.0163 | 0.0014 | 1.30E-29 |
| rs2310357 | T | C | 0.283 | -0.0158 | 0.0016 | 4.10E-22 |
| rs2322193 | A | C | 0.3172 | 0.0144 | 0.0016 | 6.10E-20 |
| rs2347705 | T | G | 0.6047 | -0.015 | 0.0015 | 3.20E-24 |
| rs2354584 | A | G | 0.8708 | -0.0136 | 0.0022 | 4.20E-10 |
| rs235763 | T | C | 0.5869 | 0.021 | 0.0015 | 6.00E-45 |
| rs2361797 | A | G | 0.447 | -0.0097 | 0.0015 | 5.90E-11 |
| rs2372048 | T | C | 0.1606 | 0.0122 | 0.002 | 4.30E-10 |
| rs2411257 | T | C | 0.7627 | 0.0122 | 0.0017 | 3.70E-13 |
| rs2414 | T | C | 0.392 | -0.0128 | 0.0015 | 6.30E-17 |
| rs2421501 | A | G | 0.5426 | -0.0105 | 0.0014 | 2.20E-13 |
| rs2429034 | T | C | 0.05221 | 0.0184 | 0.0033 | 3.50E-08 |
| rs2442778 | A | G | 0.95165 | 0.0418 | 0.0033 | 7.90E-37 |
| rs2450909 | T | C | 0.4505 | -0.0116 | 0.0014 | 7.20E-16 |
| rs246185 | T | C | 0.677 | -0.0274 | 0.0016 | 1.80E-66 |
| rs2514841 | T | C | 0.4681 | 0.0108 | 0.0014 | 9.10E-14 |
| rs2515462 | A | G | 0.33 | 0.0095 | 0.0015 | 7.20E-10 |
| rs2568189 | A | G | 0.8216 | 0.015 | 0.0019 | 2.50E-15 |
| rs2569881 | A | G | 0.1317 | 0.0175 | 0.0022 | 4.30E-16 |
| rs2578234 | A | G | 0.7737 | -0.028 | 0.0018 | 2.30E-57 |
| rs2581830 | T | C | 0.4116 | 0.0305 | 0.0015 | 1.50E-97 |
| rs2583236 | C | G | 0.406 | 0.0093 | 0.0015 | 1.80E-10 |
| rs2586255 | A | G | 0.2031 | -0.0131 | 0.0019 | 5.60E-12 |
| rs258790 | T | C | 0.6054 | -0.0088 | 0.0015 | 2.60E-09 |
| rs2596503 | A | G | 0.2022 | 0.0257 | 0.0018 | 4.80E-48 |
| rs2597513 | T | C | 0.8972 | -0.0372 | 0.0023 | 7.80E-57 |
| rs2615074 | A | G | 0.3765 | 0.0096 | 0.0015 | 7.50E-11 |
| rs2624195 | T | G | 0.751 | 0.0145 | 0.0017 | 2.80E-18 |
| rs263640 | A | G | 0.09495 | -0.0252 | 0.0024 | 6.10E-26 |
| rs2654970 | A | G | 0.2354 | 0.0178 | 0.0017 | 1.70E-24 |
| rs2682560 | T | C | 0.1939 | 0.0238 | 0.0018 | 4.00E-38 |
| rs26840 | T | C | 0.4228 | 0.0269 | 0.0015 | 3.60E-73 |
| rs2707608 | T | C | 0.3642 | 0.0113 | 0.0015 | 1.40E-13 |
| rs2712270 | A | G | 0.127 | 0.0172 | 0.0022 | 1.50E-15 |
| rs2715094 | A | G | 0.7618 | -0.016 | 0.0017 | 3.90E-22 |
| rs2715553 | A | G | 0.552 | 0.02 | 0.0015 | 1.70E-42 |
| rs2731646 | A | G | 0.4865 | 0.0121 | 0.0014 | 4.10E-17 |
| rs273945 | A | C | 0.4256 | -0.0167 | 0.0015 | 4.00E-30 |
| rs274679 | A | C | 0.3388 | 0.0134 | 0.0015 | 8.20E-19 |
| rs2763273 | T | C | 0.2255 | -0.0273 | 0.0017 | 3.70E-59 |
| rs2764082 | T | C | 0.5564 | -0.0088 | 0.0015 | 2.10E-09 |
| rs2775130 | A | C | 0.3152 | -0.0094 | 0.0016 | 4.30E-09 |
| rs2789517 | A | G | 0.8785 | 0.0229 | 0.0022 | 3.10E-26 |
| rs2803900 | A | T | 0.8463 | -0.0119 | 0.002 | 3.60E-09 |
| rs2815547 | T | C | 0.2466 | -0.0127 | 0.0017 | 2.20E-14 |
| rs2816166 | C | G | 0.4857 | 0.0086 | 0.0014 | 1.60E-09 |
| rs2816928 | T | G | 0.4882 | 0.0097 | 0.0014 | 7.90E-12 |
| rs2818052 | A | G | 0.1361 | 0.0167 | 0.0021 | 1.50E-15 |
| rs2828801 | A | G | 0.5308 | 0.008 | 0.0015 | 4.20E-08 |
| rs2830581 | A | G | 0.1549 | -0.0177 | 0.002 | 4.30E-19 |
| rs2835191 | A | G | 0.09775 | 0.0137 | 0.0024 | 2.30E-08 |
| rs2835676 | T | C | 0.3337 | 0.0111 | 0.0015 | 7.30E-13 |
| rs2853406 | A | G | 0.07484 | 0.0426 | 0.0026 | 6.20E-60 |
| rs2856321 | A | G | 0.6471 | -0.029 | 0.0015 | 6.40E-85 |
| rs2874670 | A | G | 0.4585 | 0.0129 | 0.0014 | 6.30E-19 |
| rs2897347 | T | C | 0.8857 | 0.0143 | 0.0024 | 4.30E-09 |
| rs291224 | C | G | 0.4244 | 0.0112 | 0.0015 | 1.50E-14 |
| rs2925155 | T | C | 0.2595 | -0.0264 | 0.0017 | 9.40E-57 |
| rs293710 | T | C | 0.6864 | 0.0315 | 0.0015 | 3.00E-93 |
| rs2939930 | A | G | 0.6051 | -0.0144 | 0.0015 | 1.30E-22 |
| rs2950241 | A | G | 0.747 | -0.0135 | 0.0016 | 3.10E-16 |
| rs2961827 | A | C | 0.3556 | 0.0136 | 0.0015 | 1.50E-19 |
| rs2966379 | T | C | 0.7961 | 0.0109 | 0.0018 | 5.80E-10 |
| rs2986551 | T | C | 0.7655 | -0.0113 | 0.0017 | 1.70E-11 |
| rs299377 | T | C | 0.6832 | 0.0303 | 0.0016 | 2.10E-84 |
| rs2994329 | T | C | 0.1961 | 0.0166 | 0.0018 | 4.40E-21 |
| rs299642 | A | G | 0.6252 | 0.0127 | 0.0015 | 1.90E-17 |
| rs3011904 | A | G | 0.5855 | 0.0158 | 0.0014 | 8.40E-28 |
| rs3020407 | A | G | 0.6813 | -0.0174 | 0.0016 | 9.20E-29 |
| rs3088026 | T | C | 0.08162 | 0.0312 | 0.0026 | 8.60E-33 |
| rs3101356 | C | G | 0.2934 | 0.0088 | 0.0016 | 2.10E-08 |
| rs3110697 | A | G | 0.4188 | 0.0085 | 0.0015 | 4.80E-09 |
| rs3116168 | T | C | 0.2803 | -0.0376 | 0.0016 | ###### |
| rs3133570 | A | T | 0.7155 | 0.0095 | 0.0016 | 4.00E-09 |
| rs318095 | T | C | 0.4618 | 0.0285 | 0.0014 | 1.30E-87 |
| rs329276 | A | T | 0.5219 | 0.0091 | 0.0014 | 2.80E-10 |
| rs332139 | T | C | 0.7999 | -0.0204 | 0.0018 | 8.90E-30 |
| rs33852 | A | G | 0.6679 | -0.0279 | 0.0015 | 6.80E-75 |
| rs33992134 | T | C | 0.2976 | 0.0096 | 0.0018 | 3.70E-08 |
| rs343935 | T | C | 0.8366 | -0.022 | 0.002 | 1.70E-29 |
| rs350903 | T | C | 0.469 | -0.0095 | 0.0015 | 8.90E-10 |
| rs353406 | A | T | 0.2449 | 0.0134 | 0.0017 | 1.20E-15 |
| rs354196 | A | G | 0.473 | -0.013 | 0.0014 | 2.50E-19 |
| rs3733526 | A | G | 0.8137 | -0.0102 | 0.0018 | 3.20E-08 |
| rs3739707 | A | C | 0.2499 | -0.0218 | 0.0017 | 1.20E-38 |
| rs3757868 | A | G | 0.1808 | 0.0164 | 0.0019 | 7.00E-19 |
| rs3760318 | A | G | 0.3813 | -0.0447 | 0.0015 | ###### |
| rs3763631 | C | G | 0.6921 | 0.0169 | 0.0016 | 1.80E-27 |
| rs3771586 | T | C | 0.6255 | 0.0175 | 0.0015 | 6.70E-33 |
| rs3776069 | T | C | 0.8483 | -0.017 | 0.002 | 2.50E-17 |
| rs3790085 | A | G | 0.5552 | 0.024 | 0.0014 | 5.90E-62 |
| rs3794782 | C | G | 0.3895 | 0.0148 | 0.0015 | 1.10E-23 |
| rs37974 | A | G | 0.5147 | -0.0207 | 0.0015 | 3.40E-45 |
| rs3800406 | A | G | 0.8895 | -0.0513 | 0.0025 | 4.60E-97 |
| rs3808876 | A | G | 0.4299 | -0.0114 | 0.0015 | 2.70E-14 |
| rs3812040 | T | C | 0.7322 | 0.0221 | 0.0016 | 2.20E-43 |
| rs3812163 | A | T | 0.541 | -0.0404 | 0.0014 | ###### |
| rs3816769 | T | C | 0.657 | -0.0102 | 0.0015 | 8.00E-12 |
| rs3825199 | A | G | 0.7811 | -0.0521 | 0.0017 | ###### |
| rs3829835 | T | C | 0.3543 | 0.026 | 0.0015 | 2.30E-69 |
| rs3852401 | A | G | 0.91879 | 0.0215 | 0.0026 | 2.30E-16 |
| rs3868143 | T | C | 0.92411 | 0.0349 | 0.0027 | 4.70E-37 |
| rs388415 | T | C | 0.683 | -0.0113 | 0.0016 | 3.80E-12 |
| rs3885668 | T | C | 0.5714 | -0.022 | 0.0014 | 9.90E-53 |
| rs3890593 | A | G | 0.2912 | -0.0157 | 0.0016 | 2.10E-22 |
| rs4026608 | T | C | 0.6165 | 0.0258 | 0.0015 | 1.90E-67 |
| rs4072910 | C | G | 0.4621 | -0.0293 | 0.0015 | 2.60E-83 |
| rs4072980 | A | G | 0.418 | 0.02 | 0.0015 | 3.50E-42 |
| rs4073455 | T | G | 0.449 | 0.0184 | 0.0015 | 3.50E-34 |
| rs4077074 | T | C | 0.3005 | -0.0186 | 0.0016 | 1.10E-29 |
| rs4146115 | C | G | 0.4467 | 0.0107 | 0.0015 | 1.70E-13 |
| rs4236337 | A | C | 0.6803 | 0.0084 | 0.0015 | 3.70E-08 |
| rs4246302 | A | G | 0.6949 | -0.0239 | 0.0016 | 1.10E-51 |
| rs4278208 | A | G | 0.7957 | 0.0147 | 0.0018 | 1.10E-16 |
| rs4279453 | T | C | 0.5651 | 0.0188 | 0.0014 | 7.90E-39 |
| rs4282339 | A | G | 0.2077 | -0.0368 | 0.0018 | 1.70E-97 |
| rs4287972 | T | C | 0.4836 | -0.0186 | 0.0016 | 1.30E-32 |
| rs4291276 | A | G | 0.2328 | 0.0287 | 0.0017 | 1.90E-64 |
| rs4302848 | A | T | 0.5327 | 0.0087 | 0.0015 | 3.60E-09 |
| rs4316494 | A | G | 0.5158 | -0.0218 | 0.0015 | 1.90E-50 |
| rs4320932 | T | C | 0.8044 | 0.0316 | 0.0019 | 1.50E-61 |
| rs4325905 | C | G | 0.3093 | 0.0469 | 0.0015 | ###### |
| rs434818 | A | G | 0.87 | 0.0152 | 0.0021 | 4.10E-13 |
| rs4373007 | C | G | 0.8346 | 0.0297 | 0.002 | 7.20E-50 |
| rs4377985 | A | C | 0.5219 | -0.016 | 0.0014 | 1.10E-28 |
| rs439223 | A | G | 0.07261 | -0.0256 | 0.0028 | 1.70E-19 |
| rs4394764 | A | G | 0.1984 | -0.0114 | 0.0018 | 3.60E-10 |
| rs4404990 | T | C | 0.4254 | 0.0126 | 0.0015 | 3.10E-18 |
| rs4425077 | C | G | 0.5779 | -0.0146 | 0.0014 | 5.50E-24 |
| rs4436722 | A | G | 0.8129 | 0.0211 | 0.0019 | 9.50E-30 |
| rs4437632 | A | C | 0.7351 | 0.0099 | 0.0016 | 1.70E-09 |
| rs4444108 | A | G | 0.1352 | 0.0118 | 0.0021 | 3.70E-08 |
| rs4461027 | T | C | 0.5833 | 0.0299 | 0.0015 | 1.70E-91 |
| rs4470224 | A | T | 0.7993 | 0.0105 | 0.0018 | 6.30E-09 |
| rs4484937 | T | C | 0.1614 | 0.0136 | 0.0019 | 8.60E-13 |
| rs448513 | T | C | 0.6825 | 0.01 | 0.0015 | 5.50E-11 |
| rs4502032 | A | C | 0.3983 | 0.0113 | 0.0015 | 8.90E-15 |
| rs450986 | C | G | 0.09203 | 0.0267 | 0.0025 | 7.20E-27 |
| rs4519409 | A | C | 0.35 | -0.0134 | 0.0015 | 1.00E-18 |
| rs4520026 | A | G | 0.7663 | 0.0167 | 0.0017 | 5.70E-22 |
| rs4523278 | T | C | 0.5544 | -0.0126 | 0.0014 | 1.80E-18 |
| rs4533267 | A | G | 0.2843 | 0.0336 | 0.0016 | ###### |
| rs4539979 | T | C | 0.6731 | 0.022 | 0.0015 | 1.20E-47 |
| rs4548769 | C | G | 0.6957 | -0.0238 | 0.0016 | 5.20E-51 |
| rs4559164 | C | G | 0.4132 | -0.0083 | 0.0015 | 1.30E-08 |
| rs4561893 | T | G | 0.05028 | 0.031 | 0.0033 | 1.90E-21 |
| rs4567655 | T | C | 0.5405 | -0.0204 | 0.0015 | 6.90E-45 |
| rs4568061 | A | C | 0.3344 | -0.0102 | 0.0015 | 1.50E-11 |
| rs4640244 | A | G | 0.5999 | 0.0227 | 0.0015 | 3.00E-51 |
| rs4640402 | A | C | 0.5889 | -0.0123 | 0.0014 | 1.20E-17 |
| rs4646450 | A | G | 0.1618 | 0.0197 | 0.002 | 1.10E-23 |
| rs4653828 | A | G | 0.2502 | 0.0134 | 0.0016 | 2.10E-16 |
| rs4655345 | A | G | 0.6003 | 0.0215 | 0.0015 | 1.50E-49 |
| rs467238 | A | G | 0.2335 | 0.0166 | 0.0017 | 1.80E-22 |
| rs4686904 | T | C | 0.6488 | -0.0203 | 0.0015 | 7.00E-42 |
| rs4695794 | C | G | 0.7952 | -0.0101 | 0.0018 | 1.30E-08 |
| rs4699261 | A | G | 0.3204 | -0.011 | 0.0017 | 4.10E-11 |
| rs4715019 | A | T | 0.2709 | 0.0156 | 0.0016 | 1.30E-22 |
| rs4719815 | A | C | 0.0908 | -0.0211 | 0.0024 | 3.60E-18 |
| rs4722834 | T | C | 0.6096 | 0.0146 | 0.0015 | 8.30E-23 |
| rs4733792 | T | C | 0.4035 | -0.0101 | 0.0015 | 7.00E-12 |
| rs4735639 | A | C | 0.90692 | 0.0164 | 0.0025 | 3.20E-11 |
| rs473902 | T | G | 0.91242 | 0.0701 | 0.0027 | ###### |
| rs4758684 | A | G | 0.3313 | -0.0152 | 0.0015 | 4.20E-23 |
| rs4760168 | T | G | 0.3368 | -0.018 | 0.0015 | 3.80E-32 |
| rs4764861 | T | C | 0.5805 | 0.025 | 0.0015 | 5.60E-65 |
| rs4767473 | A | G | 0.8642 | 0.0246 | 0.0021 | 2.10E-30 |
| rs478259 | A | G | 0.8533 | 0.0117 | 0.0021 | 1.50E-08 |
| rs4784463 | A | G | 0.5393 | -0.0107 | 0.0015 | 1.80E-13 |
| rs4790404 | T | C | 0.5413 | -0.008 | 0.0015 | 4.20E-08 |
| rs4800451 | T | C | 0.7415 | 0.0572 | 0.0017 | ###### |
| rs4802216 | T | C | 0.3373 | -0.0087 | 0.0015 | 1.00E-08 |
| rs4852777 | C | G | 0.5945 | -0.0297 | 0.0014 | 1.00E-93 |
| rs4865615 | C | G | 0.3328 | 0.0315 | 0.0015 | 7.50E-96 |
| rs4912090 | T | C | 0.771 | -0.0174 | 0.0017 | 5.20E-25 |
| rs4924564 | A | T | 0.3483 | 0.0156 | 0.0015 | 3.40E-25 |
| rs4939837 | A | G | 0.3551 | 0.019 | 0.0016 | 3.20E-34 |
| rs4951022 | T | C | 0.7271 | -0.015 | 0.0016 | 2.40E-21 |
| rs495360 | A | C | 0.4967 | -0.0121 | 0.0015 | 7.20E-16 |
| rs4986172 | T | C | 0.3457 | -0.0306 | 0.0015 | 3.00E-89 |
| rs508347 | T | C | 0.2927 | 0.0472 | 0.0016 | ###### |
| rs519384 | A | T | 0.2802 | 0.0333 | 0.0016 | 1.00E-96 |
| rs521765 | C | G | 0.848 | 0.0179 | 0.0023 | 2.20E-15 |
| rs522377 | T | C | 0.4349 | -0.0278 | 0.0014 | 6.40E-85 |
| rs528396 | T | C | 0.6745 | 0.0109 | 0.0016 | 3.20E-12 |
| rs531790 | T | C | 0.8874 | -0.0141 | 0.0023 | 9.40E-10 |
| rs540652 | T | C | 0.4668 | 0.0231 | 0.0014 | 8.40E-60 |
| rs542380 | A | G | 0.1742 | -0.0174 | 0.0019 | 1.90E-20 |
| rs546333 | A | G | 0.7131 | 0.014 | 0.0016 | 1.90E-18 |
| rs5742915 | T | C | 0.5517 | -0.0326 | 0.0015 | ###### |
| rs5754387 | C | G | 0.2005 | -0.0142 | 0.0018 | 6.90E-15 |
| rs5757318 | A | T | 0.8424 | -0.0212 | 0.0021 | 4.50E-24 |
| rs585447 | A | G | 0.8344 | 0.0116 | 0.0019 | 1.80E-09 |
| rs5930 | A | G | 0.3856 | 0.009 | 0.0015 | 2.10E-09 |
| rs597437 | C | G | 0.2951 | -0.0136 | 0.0016 | 1.10E-17 |
| rs598132 | T | C | 0.04841 | 0.0177 | 0.0032 | 4.30E-08 |
| rs601055 | A | G | 0.8234 | 0.0214 | 0.0019 | 1.80E-29 |
| rs6010778 | T | C | 0.6658 | -0.0132 | 0.0016 | 5.80E-17 |
| rs6012927 | A | G | 0.651 | 0.012 | 0.0015 | 4.50E-15 |
| rs601318 | C | G | 0.6805 | -0.0158 | 0.0015 | 1.30E-24 |
| rs6020170 | A | G | 0.8086 | 0.0207 | 0.0018 | 9.50E-30 |
| rs602633 | T | G | 0.2199 | 0.0153 | 0.0017 | 5.50E-19 |
| rs6031858 | T | G | 0.2803 | 0.017 | 0.0016 | 1.90E-25 |
| rs6053961 | A | G | 0.4847 | -0.0098 | 0.0015 | 2.30E-11 |
| rs6060959 | A | G | 0.3107 | -0.0097 | 0.0016 | 2.20E-09 |
| rs606452 | A | C | 0.1332 | 0.0488 | 0.0021 | ###### |
| rs6088765 | T | G | 0.5684 | -0.0496 | 0.0015 | ###### |
| rs6107581 | A | C | 0.8946 | 0.0262 | 0.0024 | 3.80E-27 |
| rs6107848 | A | G | 0.3714 | 0.0459 | 0.0015 | ###### |
| rs6136938 | A | G | 0.4339 | -0.0096 | 0.0015 | 4.10E-11 |
| rs6137297 | T | C | 0.6669 | -0.0192 | 0.0015 | 1.00E-35 |
| rs619698 | A | C | 0.3546 | 0.0169 | 0.0016 | 8.20E-27 |
| rs6414859 | T | C | 0.229 | -0.028 | 0.0017 | 1.50E-61 |
| rs6457873 | T | C | 0.2886 | 0.0104 | 0.0016 | 3.90E-11 |
| rs6468409 | T | C | 0.782 | 0.0117 | 0.0017 | 1.00E-11 |
| rs6486361 | A | G | 0.3373 | -0.0088 | 0.0015 | 9.10E-09 |
| rs648831 | T | C | 0.5281 | 0.0291 | 0.0014 | 8.70E-91 |
| rs6489269 | A | G | 0.2818 | 0.0097 | 0.0016 | 1.50E-09 |
| rs6498960 | T | C | 0.1805 | -0.0104 | 0.0019 | 3.80E-08 |
| rs652112 | T | C | 0.8906 | -0.0322 | 0.0023 | 4.00E-44 |
| rs6539029 | A | G | 0.1234 | -0.0221 | 0.0021 | 2.20E-25 |
| rs6549499 | T | C | 0.513 | 0.0106 | 0.0014 | 1.20E-13 |
| rs6556079 | A | G | 0.5958 | -0.024 | 0.0015 | 7.40E-61 |
| rs6561319 | A | C | 0.6312 | 0.0157 | 0.0015 | 2.40E-25 |
| rs6562757 | A | G | 0.303 | 0.0144 | 0.0016 | 9.80E-20 |
| rs6567160 | T | C | 0.7702 | -0.0297 | 0.0017 | 6.50E-67 |
| rs6568938 | C | G | 0.723 | 0.022 | 0.0016 | 1.80E-42 |
| rs6580254 | C | G | 0.7236 | -0.0108 | 0.0016 | 1.40E-11 |
| rs6581626 | A | G | 0.4668 | 0.02 | 0.0014 | 2.30E-44 |
| rs658851 | T | C | 0.3919 | -0.0198 | 0.0015 | 3.30E-42 |
| rs6597233 | C | G | 0.1891 | -0.0201 | 0.0018 | 8.20E-28 |
| rs6598473 | A | G | 0.1198 | -0.0139 | 0.0023 | 8.00E-10 |
| rs661885 | A | C | 0.2841 | -0.0094 | 0.0016 | 6.20E-09 |
| rs6658835 | A | G | 0.7358 | -0.0214 | 0.0016 | 4.00E-40 |
| rs6659148 | T | C | 0.497 | 0.0094 | 0.0014 | 3.80E-11 |
| rs6667049 | T | C | 0.123 | 0.0208 | 0.0022 | 1.30E-21 |
| rs6689960 | A | G | 0.3996 | 0.0095 | 0.0015 | 6.50E-11 |
| rs6691924 | T | C | 0.8873 | 0.0278 | 0.0023 | 5.20E-34 |
| rs6714546 | A | G | 0.2861 | -0.0307 | 0.0016 | 6.90E-84 |
| rs6719073 | C | G | 0.3419 | 0.0166 | 0.0015 | 9.90E-27 |
| rs6739701 | A | G | 0.5701 | -0.0151 | 0.0015 | 2.90E-25 |
| rs6739772 | A | G | 0.2952 | -0.0172 | 0.0016 | 5.10E-28 |
| rs6747243 | T | C | 0.5666 | -0.01 | 0.0014 | 2.20E-12 |
| rs6756818 | A | T | 0.2527 | 0.0193 | 0.0016 | 2.50E-32 |
| rs6759952 | T | C | 0.4082 | -0.0251 | 0.0014 | 9.90E-69 |
| rs6765930 | A | G | 0.2197 | -0.0385 | 0.0017 | ###### |
| rs6768839 | T | C | 0.7674 | 0.0094 | 0.0017 | 2.10E-08 |
| rs6786420 | T | C | 0.7143 | -0.0157 | 0.0016 | 5.00E-23 |
| rs679064 | A | G | 0.1194 | 0.0169 | 0.0022 | 1.80E-14 |
| rs6792408 | A | G | 0.2519 | -0.0332 | 0.0017 | 1.30E-88 |
| rs6798330 | T | C | 0.94288 | -0.0208 | 0.0035 | 1.90E-09 |
| rs6802104 | T | C | 0.6279 | -0.0191 | 0.0015 | 4.70E-35 |
| rs6822154 | A | C | 0.3279 | -0.0147 | 0.0015 | 2.60E-22 |
| rs6827794 | T | C | 0.2101 | -0.025 | 0.0018 | 3.40E-45 |
| rs6831817 | A | G | 0.5568 | -0.0114 | 0.0015 | 4.90E-15 |
| rs683503 | T | C | 0.2555 | 0.0128 | 0.0016 | 1.90E-15 |
| rs683633 | T | C | 0.8599 | -0.0218 | 0.0021 | 3.70E-26 |
| rs6838153 | A | G | 0.6698 | -0.0228 | 0.0015 | 4.90E-50 |
| rs6880702 | A | G | 0.6535 | -0.0098 | 0.0015 | 9.00E-11 |
| rs6892868 | A | G | 0.2573 | -0.0119 | 0.0016 | 2.70E-13 |
| rs689505 | T | C | 0.3633 | -0.0099 | 0.0015 | 3.20E-11 |
| rs6919321 | A | G | 0.6019 | 0.0204 | 0.0015 | 2.10E-44 |
| rs6920372 | A | G | 0.4089 | -0.0238 | 0.0014 | 8.90E-62 |
| rs6926954 | T | G | 0.685 | -0.0106 | 0.0016 | 1.00E-11 |
| rs6927242 | A | G | 0.5478 | -0.0098 | 0.0014 | 1.10E-11 |
| rs6949085 | T | C | 0.3922 | -0.0104 | 0.0015 | 1.90E-12 |
| rs6949428 | A | G | 0.671 | -0.0212 | 0.0015 | 4.40E-44 |
| rs6950680 | A | G | 0.6182 | 0.017 | 0.0015 | 6.00E-31 |
| rs6956540 | T | C | 0.5958 | 0.0134 | 0.0015 | 5.60E-20 |
| rs6958277 | A | G | 0.5816 | -0.0109 | 0.0015 | 8.30E-14 |
| rs696 | T | C | 0.3801 | -0.0153 | 0.0015 | 1.40E-23 |
| rs6962887 | T | G | 0.6785 | 0.0226 | 0.0016 | 2.60E-46 |
| rs696343 | A | G | 0.8886 | 0.0131 | 0.0023 | 9.50E-09 |
| rs696835 | T | C | 0.2181 | 0.0165 | 0.0017 | 1.20E-21 |
| rs6974757 | C | G | 0.4242 | 0.0119 | 0.0015 | 4.70E-16 |
| rs6977081 | T | G | 0.3456 | 0.0277 | 0.0016 | 2.60E-71 |
| rs6981216 | A | G | 0.7884 | -0.0111 | 0.0018 | 3.30E-10 |
| rs6997004 | A | G | 0.8359 | 0.0184 | 0.002 | 2.10E-20 |
| rs702691 | T | G | 0.3454 | -0.0181 | 0.0015 | 6.70E-34 |
| rs7033940 | C | G | 0.1184 | -0.0138 | 0.0023 | 1.10E-09 |
| rs704660 | T | C | 0.4042 | 0.0188 | 0.0015 | 9.40E-38 |
| rs7078243 | A | C | 0.4834 | 0.0085 | 0.0014 | 3.30E-09 |
| rs7089424 | T | G | 0.6682 | -0.0155 | 0.0015 | 3.10E-24 |
| rs7093473 | T | C | 0.8855 | -0.0288 | 0.0023 | 6.30E-37 |
| rs7107012 | A | G | 0.572 | 0.0177 | 0.0014 | 1.70E-34 |
| rs7110302 | T | C | 0.3538 | -0.0211 | 0.0015 | 1.10E-42 |
| rs713140 | A | G | 0.6345 | 0.0157 | 0.0015 | 5.90E-26 |
| rs7154721 | T | C | 0.5706 | 0.0295 | 0.0015 | 2.60E-91 |
| rs7162542 | C | G | 0.4384 | -0.0483 | 0.0014 | ###### |
| rs7168331 | C | G | 0.3851 | -0.0161 | 0.0015 | 1.80E-27 |
| rs7176856 | T | G | 0.7495 | -0.0157 | 0.0017 | 5.50E-21 |
| rs7195553 | A | G | 0.3074 | -0.018 | 0.0016 | 4.80E-30 |
| rs719756 | A | T | 0.4491 | -0.0218 | 0.0014 | 2.30E-52 |
| rs720390 | A | G | 0.3748 | 0.0329 | 0.0015 | ###### |
| rs7205337 | A | G | 0.7935 | -0.0355 | 0.0018 | 6.50E-85 |
| rs7210446 | A | G | 0.5771 | 0.017 | 0.0014 | 7.50E-32 |
| rs7219215 | T | C | 0.8539 | 0.0177 | 0.002 | 4.80E-18 |
| rs723149 | A | G | 0.4422 | 0.0239 | 0.0015 | 2.20E-59 |
| rs723587 | A | G | 0.1061 | 0.0246 | 0.0024 | 4.00E-24 |
| rs7253538 | A | G | 0.1437 | 0.0158 | 0.0021 | 5.00E-14 |
| rs726547 | A | G | 0.04952 | -0.0541 | 0.0035 | 6.30E-55 |
| rs7273787 | A | G | 0.6632 | -0.025 | 0.0015 | 1.70E-60 |
| rs7307562 | T | G | 0.3934 | 0.0081 | 0.0015 | 3.00E-08 |
| rs7314810 | T | C | 0.3391 | -0.0088 | 0.0015 | 5.70E-09 |
| rs7319045 | A | G | 0.3806 | 0.0266 | 0.0015 | 4.70E-71 |
| rs732855 | A | G | 0.6304 | 0.0092 | 0.0015 | 8.60E-10 |
| rs7334355 | A | G | 0.6238 | -0.0123 | 0.0015 | 1.80E-16 |
| rs7334370 | A | G | 0.3132 | 0.0101 | 0.0016 | 1.40E-10 |
| rs7336104 | T | C | 0.1756 | -0.0148 | 0.0019 | 1.30E-14 |
| rs7338171 | A | G | 0.8603 | -0.0177 | 0.0021 | 2.80E-17 |
| rs739984 | A | G | 0.1883 | -0.0115 | 0.0018 | 3.40E-10 |
| rs7466269 | A | G | 0.6509 | 0.0325 | 0.0015 | ###### |
| rs7478426 | T | C | 0.07074 | 0.0207 | 0.0031 | 1.30E-11 |
| rs7487625 | A | C | 0.7717 | 0.054 | 0.0017 | ###### |
| rs7534091 | A | G | 0.7493 | 0.0419 | 0.0016 | ###### |
| rs7561119 | T | C | 0.8952 | 0.0417 | 0.0024 | 6.90E-70 |
| rs7564469 | T | C | 0.8475 | -0.0126 | 0.002 | 1.20E-10 |
| rs757081 | C | G | 0.6692 | -0.0182 | 0.0015 | 6.50E-33 |
| rs7585767 | A | G | 0.5104 | 0.0132 | 0.0015 | 2.10E-19 |
| rs759285 | T | G | 0.8754 | -0.018 | 0.0022 | 2.20E-16 |
| rs7602070 | A | G | 0.7968 | -0.0296 | 0.0018 | 2.30E-60 |
| rs7614810 | A | G | 0.7846 | -0.0119 | 0.0018 | 1.20E-10 |
| rs7641020 | T | G | 0.5691 | -0.0085 | 0.0014 | 3.70E-09 |
| rs7652486 | A | G | 0.689 | -0.0092 | 0.0015 | 2.50E-09 |
| rs7672919 | T | G | 0.1554 | -0.0678 | 0.002 | ###### |
| rs768023 | A | G | 0.6254 | 0.0212 | 0.0015 | 2.40E-47 |
| rs7688346 | A | G | 0.848 | 0.0215 | 0.0021 | 1.10E-24 |
| rs7692554 | A | G | 0.4435 | 0.0526 | 0.0015 | ###### |
| rs7697556 | T | C | 0.4846 | 0.0315 | 0.0014 | ###### |
| rs7701414 | A | G | 0.5469 | -0.0351 | 0.0014 | ###### |
| rs7721618 | T | G | 0.8518 | -0.0156 | 0.002 | 5.10E-15 |
| rs7733998 | T | C | 0.7156 | 0.0118 | 0.0016 | 3.30E-13 |
| rs7742369 | A | G | 0.8236 | -0.0626 | 0.0019 | ###### |
| rs7752292 | A | T | 0.3671 | 0.026 | 0.0015 | 1.70E-70 |
| rs778338 | A | G | 0.4173 | -0.0166 | 0.0014 | 8.10E-31 |
| rs7793196 | A | G | 0.8258 | -0.0158 | 0.0019 | 2.20E-17 |
| rs7794103 | T | C | 0.5031 | 0.0094 | 0.0014 | 5.80E-11 |
| rs7796089 | C | G | 0.3389 | 0.0143 | 0.0015 | 1.50E-21 |
| rs7804260 | A | G | 0.4145 | -0.0109 | 0.0015 | 7.40E-14 |
| rs7815955 | A | T | 0.7955 | 0.0478 | 0.0018 | ###### |
| rs7831581 | T | C | 0.6211 | 0.0161 | 0.0015 | 1.20E-27 |
| rs7833723 | A | G | 0.4421 | -0.0122 | 0.0014 | 2.70E-17 |
| rs7849585 | T | G | 0.3297 | 0.03 | 0.0015 | 1.50E-85 |
| rs7853634 | T | C | 0.8023 | 0.0413 | 0.0018 | ###### |
| rs7866461 | C | G | 0.7315 | 0.0125 | 0.0017 | 3.80E-13 |
| rs7896371 | T | C | 0.4549 | 0.0104 | 0.0015 | 1.80E-12 |
| rs790156 | A | G | 0.5631 | -0.0159 | 0.0014 | 3.70E-28 |
| rs7904976 | A | G | 0.6139 | -0.0092 | 0.0015 | 3.10E-09 |
| rs7910211 | T | C | 0.8225 | -0.0173 | 0.002 | 1.50E-18 |
| rs7928703 | T | G | 0.5039 | 0.0118 | 0.0015 | 7.50E-16 |
| rs793887 | A | C | 0.95101 | -0.0184 | 0.0034 | 4.50E-08 |
| rs7947870 | A | G | 0.9368 | -0.0188 | 0.003 | 3.80E-10 |
| rs7948782 | T | C | 0.1469 | -0.0315 | 0.002 | 8.40E-54 |
| rs7960695 | A | G | 0.07641 | 0.0191 | 0.0027 | 1.40E-12 |
| rs7961913 | C | G | 0.2187 | -0.0113 | 0.0018 | 1.30E-10 |
| rs7978559 | A | G | 0.3176 | 0.0179 | 0.0015 | 1.70E-31 |
| rs7982373 | A | G | 0.77 | 0.026 | 0.0017 | 8.00E-53 |
| rs798565 | A | G | 0.2914 | -0.055 | 0.0016 | ###### |
| rs7987131 | A | G | 0.7096 | -0.0112 | 0.0016 | 1.00E-11 |
| rs7989711 | T | C | 0.3075 | -0.0131 | 0.0016 | 4.70E-17 |
| rs8028537 | A | G | 0.5098 | -0.0291 | 0.0015 | 6.80E-89 |
| rs8037574 | T | C | 0.7235 | 0.0202 | 0.0016 | 1.10E-35 |
| rs8046199 | A | G | 0.2649 | -0.0091 | 0.0017 | 4.40E-08 |
| rs8052560 | A | C | 0.7782 | 0.0266 | 0.0018 | 2.60E-50 |
| rs8053537 | A | G | 0.8002 | -0.0109 | 0.0018 | 2.00E-09 |
| rs8058684 | A | G | 0.3113 | 0.0265 | 0.0016 | 6.40E-64 |
| rs8067165 | C | G | 0.3952 | -0.0203 | 0.0015 | 1.30E-40 |
| rs806794 | A | G | 0.7062 | 0.0586 | 0.0016 | ###### |
| rs8070737 | T | G | 0.1838 | 0.013 | 0.0019 | 3.80E-12 |
| rs8086549 | A | C | 0.5507 | -0.0113 | 0.0015 | 6.30E-15 |
| rs8088885 | T | C | 0.8707 | -0.0137 | 0.0022 | 6.60E-10 |
| rs8090312 | A | G | 0.6918 | 0.0218 | 0.0016 | 1.20E-42 |
| rs8097893 | A | G | 0.95522 | 0.0356 | 0.0035 | 9.20E-24 |
| rs811133 | T | C | 0.1944 | -0.0141 | 0.0018 | 2.60E-14 |
| rs8113006 | C | G | 0.8 | -0.0263 | 0.0018 | 5.10E-48 |
| rs8179 | T | C | 0.2115 | 0.0672 | 0.0018 | ###### |
| rs8180991 | C | G | 0.7662 | 0.0266 | 0.0017 | 6.70E-53 |
| rs822531 | T | C | 0.7934 | 0.0436 | 0.0018 | ###### |
| rs826941 | A | G | 0.8028 | -0.0133 | 0.0018 | 9.40E-14 |
| rs827135 | T | C | 0.5842 | -0.0207 | 0.0015 | 4.90E-46 |
| rs835109 | T | C | 0.1527 | -0.0155 | 0.002 | 6.20E-15 |
| rs8413 | T | C | 0.572 | -0.019 | 0.0015 | 3.80E-39 |
| rs852928 | T | C | 0.6402 | -0.0152 | 0.0015 | 3.30E-25 |
| rs862049 | T | C | 0.6517 | 0.0294 | 0.0015 | 9.60E-85 |
| rs877382 | T | C | 0.7294 | 0.0133 | 0.0017 | 2.30E-15 |
| rs878995 | T | G | 0.05741 | -0.0222 | 0.0031 | 1.30E-12 |
| rs881980 | T | G | 0.8697 | -0.0136 | 0.0021 | 1.80E-10 |
| rs884215 | A | G | 0.5897 | 0.0172 | 0.0015 | 8.40E-32 |
| rs884522 | T | C | 0.3101 | -0.0095 | 0.0016 | 1.00E-09 |
| rs899286 | A | G | 0.1786 | -0.0146 | 0.0019 | 4.90E-14 |
| rs903347 | T | C | 0.5876 | 0.0111 | 0.0014 | 1.60E-14 |
| rs914327 | C | G | 0.7899 | -0.0111 | 0.0018 | 4.60E-10 |
| rs9217 | T | C | 0.6438 | -0.0321 | 0.0015 | ###### |
| rs927381 | T | C | 0.5535 | 0.0191 | 0.0015 | 9.70E-39 |
| rs9291926 | T | G | 0.4857 | 0.0183 | 0.0015 | 2.20E-36 |
| rs9300607 | A | C | 0.5083 | 0.0114 | 0.0015 | 8.30E-15 |
| rs9320592 | T | C | 0.276 | -0.0198 | 0.0016 | 1.20E-35 |
| rs9326750 | A | G | 0.2276 | 0.0266 | 0.0017 | 1.10E-54 |
| rs9341860 | T | C | 0.554 | 0.0299 | 0.0014 | 5.30E-97 |
| rs935728 | T | C | 0.3317 | 0.0124 | 0.0016 | 1.40E-15 |
| rs9360232 | A | G | 0.3962 | -0.009 | 0.0015 | 6.00E-10 |
| rs9364192 | A | G | 0.4235 | -0.0158 | 0.0014 | 4.50E-28 |
| rs9366426 | T | C | 0.428 | -0.0129 | 0.0015 | 4.90E-19 |
| rs9382529 | T | C | 0.895 | 0.0141 | 0.0023 | 1.50E-09 |
| rs939108 | C | G | 0.92242 | 0.0228 | 0.0029 | 1.70E-15 |
| rs9391253 | A | T | 0.6787 | -0.0477 | 0.0015 | ###### |
| rs9397448 | A | G | 0.4574 | 0.0312 | 0.0014 | ###### |
| rs9399365 | A | T | 0.7437 | -0.0118 | 0.0017 | 1.10E-12 |
| rs941873 | A | G | 0.4716 | -0.0292 | 0.0015 | 2.60E-89 |
| rs9439799 | T | C | 0.6661 | -0.0089 | 0.0015 | 3.50E-09 |
| rs9463078 | A | G | 0.3939 | 0.0229 | 0.0015 | 2.00E-55 |
| rs947099 | A | G | 0.3502 | 0.0142 | 0.0015 | 2.50E-21 |
| rs951366 | T | C | 0.6087 | -0.0176 | 0.0015 | 1.10E-33 |
| rs9536548 | T | C | 0.2164 | -0.0127 | 0.0017 | 2.40E-13 |
| rs9545588 | A | G | 0.6218 | -0.0125 | 0.0015 | 1.30E-16 |
| rs9548241 | T | C | 0.4795 | -0.008 | 0.0014 | 3.00E-08 |
| rs9549699 | A | T | 0.2311 | 0.0123 | 0.0017 | 9.40E-13 |
| rs955748 | A | G | 0.2422 | -0.0246 | 0.0017 | 7.10E-49 |
| rs956237 | A | G | 0.5724 | -0.0112 | 0.0014 | 5.80E-15 |
| rs9574556 | C | G | 0.2063 | 0.0186 | 0.0018 | 2.70E-25 |
| rs958225 | A | T | 0.05367 | 0.0349 | 0.0033 | 2.40E-26 |
| rs9611950 | A | G | 0.4885 | -0.0123 | 0.0014 | 1.70E-17 |
| rs9614470 | A | T | 0.1991 | 0.024 | 0.0018 | 6.00E-39 |
| rs9615956 | T | C | 0.0936 | 0.0142 | 0.0026 | 3.10E-08 |
| rs9625622 | T | C | 0.03555 | -0.0357 | 0.0039 | 2.70E-20 |
| rs963292 | T | C | 0.4894 | -0.0284 | 0.0014 | 3.40E-87 |
| rs9650315 | T | G | 0.1361 | -0.0609 | 0.0021 | ###### |
| rs9756 | T | C | 0.407 | -0.0101 | 0.0014 | 2.50E-12 |
| rs9765848 | T | C | 0.181 | -0.0158 | 0.0019 | 2.00E-16 |
| rs9782976 | T | C | 0.09109 | -0.0253 | 0.0025 | 1.50E-24 |
| rs9806108 | A | C | 0.7669 | 0.0107 | 0.0017 | 6.00E-10 |
| rs9807193 | T | C | 0.3594 | -0.0138 | 0.0015 | 5.60E-20 |
| rs981057 | T | C | 0.6038 | 0.0128 | 0.0015 | 4.10E-18 |
| rs9815115 | T | C | 0.94668 | -0.0199 | 0.0031 | 1.70E-10 |
| rs9818319 | C | G | 0.2589 | 0.0107 | 0.0017 | 8.90E-11 |
| rs9820833 | C | G | 0.7081 | -0.0122 | 0.0016 | 3.20E-14 |
| rs9833926 | A | G | 0.5329 | 0.0233 | 0.0014 | 7.70E-60 |
| rs9834547 | A | G | 0.8418 | -0.0176 | 0.002 | 1.60E-18 |
| rs9837001 | A | G | 0.5738 | -0.0201 | 0.0014 | 2.50E-44 |
| rs9843296 | T | C | 0.8404 | -0.0262 | 0.002 | 3.10E-40 |
| rs9844162 | A | T | 0.5527 | 0.014 | 0.0015 | 7.10E-22 |
| rs9875575 | A | G | 0.2398 | -0.0108 | 0.0017 | 1.40E-10 |
| rs9892365 | A | G | 0.3389 | 0.047 | 0.0015 | ###### |
| rs989393 | T | C | 0.7134 | 0.0197 | 0.0016 | 1.30E-35 |
| rs991946 | T | C | 0.4739 | -0.0173 | 0.0014 | 4.00E-34 |
| rs9925273 | A | G | 0.8119 | 0.0113 | 0.0019 | 2.10E-09 |
| rs9941239 | A | G | 0.2517 | 0.0226 | 0.0017 | 1.70E-39 |
| rs9941255 | A | G | 0.1983 | -0.0175 | 0.0018 | 8.50E-22 |
| rs9949076 | T | C | 0.3218 | 0.0161 | 0.0016 | 6.20E-24 |
| rs9969804 | A | C | 0.4366 | 0.0245 | 0.0014 | 7.60E-65 |
| rs997400 | T | C | 0.4591 | -0.0254 | 0.0014 | 3.10E-71 |

**Table S19. Genome-wide significant and independent SNPs that were used as instruments for smoking initiation**

| SNP | other | effect | eaf | pval | beta | se.exposure |
| --- | --- | --- | --- | --- | --- | --- |
| rs301805 | T | G | 0.559 | 2.80E-09 | 0.021468 | 0.003613 |
| rs3001723 | G | A | 0.321 | 8.12E-18 | 0.033512 | 0.003898 |
| rs6669839 | C | T | 0.204 | 3.36E-09 | 0.026004 | 0.004395 |
| rs2186122 | A | T | 0.561 | 3.61E-13 | 0.026057 | 0.003586 |
| rs7555507 | C | T | 0.496 | 1.14E-11 | -0.02414 | 0.003556 |
| rs2050586 | G | C | 0.355 | 3.00E-08 | -0.02055 | 0.003708 |
| rs12042107 | T | C | 0.527 | 4.22E-10 | -0.02228 | 0.003568 |
| rs12027999 | T | C | 0.124 | 5.76E-10 | -0.03309 | 0.005339 |
| rs2046850 | C | T | 0.187 | 3.03E-08 | -0.02481 | 0.004478 |
| rs6728726 | T | C | 0.829 | 6.73E-14 | 0.035449 | 0.004733 |
| rs1004787 | G | A | 0.581 | 5.27E-17 | 0.029923 | 0.003571 |
| rs1518393 | A | C | 0.631 | 2.03E-08 | 0.020536 | 0.003659 |
| rs7585579 | C | G | 0.505 | 1.88E-09 | 0.0224 | 0.003728 |
| rs266047 | G | A | 0.529 | 3.36E-16 | -0.03051 | 0.003739 |
| rs35702515 | G | T | 0.162 | 2.43E-09 | 0.025244 | 0.004231 |
| rs13030994 | G | A | 0.485 | 3.56E-24 | 0.036092 | 0.003556 |
| rs1445649 | T | C | 0.525 | 1.68E-11 | 0.023993 | 0.003565 |
| rs12474587 | G | T | 0.404 | 1.25E-14 | 0.027633 | 0.003582 |
| rs6433897 | T | C | 0.754 | 3.16E-08 | 0.022448 | 0.004058 |
| rs2107300 | C | G | 0.845 | 3.27E-08 | -0.0272 | 0.004925 |
| rs4674993 | A | G | 0.207 | 1.32E-08 | -0.02521 | 0.004436 |
| rs11721059 | C | T | 0.474 | 2.17E-08 | 0.019936 | 0.003563 |
| rs12632110 | A | G | 0.647 | 4.78E-10 | -0.02338 | 0.003753 |
| rs11712680 | A | C | 0.174 | 3.51E-09 | -0.02705 | 0.004578 |
| rs6788098 | A | T | 0.623 | 1.91E-17 | -0.03135 | 0.003689 |
| rs7631735 | G | T | 0.603 | 2.79E-08 | 0.020227 | 0.003645 |
| rs1154693 | A | G | 0.856 | 3.12E-11 | 0.032622 | 0.004912 |
| rs292071 | T | C | 0.244 | 4.08E-09 | 0.023582 | 0.004009 |
| rs993700 | T | C | 0.766 | 1.53E-09 | -0.02593 | 0.004292 |
| rs1160685 | C | G | 0.478 | 7.20E-09 | 0.020772 | 0.003589 |
| rs13145728 | G | C | 0.358 | 2.14E-10 | -0.02325 | 0.003663 |
| rs10001365 | G | A | 0.405 | 6.65E-12 | -0.02499 | 0.003642 |
| rs6893752 | A | G | 0.766 | 3.25E-09 | -0.0241 | 0.004074 |
| rs4571506 | C | T | 0.492 | 1.09E-14 | -0.02757 | 0.003569 |
| rs12186738 | G | T | 0.154 | 3.42E-11 | -0.03326 | 0.005021 |
| rs72789632 | C | T | 0.12 | 5.02E-10 | -0.03289 | 0.005286 |
| rs1385108 | C | T | 0.239 | 3.00E-09 | 0.024662 | 0.004157 |
| rs4044321 | A | G | 0.642 | 6.08E-14 | -0.02784 | 0.003711 |
| rs222449 | A | T | 0.793 | 1.08E-08 | -0.02532 | 0.004428 |
| rs10498846 | C | T | 0.473 | 6.62E-09 | 0.02061 | 0.003556 |
| rs9401770 | G | A | 0.273 | 3.47E-12 | 0.027731 | 0.003986 |
| rs3800227 | A | G | 0.701 | 1.93E-08 | 0.022812 | 0.004058 |
| rs240963 | T | C | 0.836 | 2.16E-17 | -0.04104 | 0.004837 |
| rs4236259 | T | G | 0.499 | 3.35E-12 | -0.02477 | 0.003557 |
| rs10260968 | G | A | 0.597 | 1.75E-08 | -0.02032 | 0.003609 |
| rs13246563 | C | G | 0.526 | 3.45E-10 | -0.02346 | 0.003738 |
| rs12112638 | A | G | 0.275 | 1.34E-09 | -0.02453 | 0.004043 |
| rs11768481 | C | A | 0.347 | 7.00E-10 | -0.0232 | 0.003764 |
| rs12333760 | T | C | 0.204 | 1.44E-09 | -0.02905 | 0.004801 |
| rs10233018 | A | G | 0.503 | 2.75E-14 | 0.027069 | 0.003557 |
| rs10279261 | G | A | 0.619 | 5.00E-09 | -0.02142 | 0.003663 |
| rs1565735 | T | A | 0.212 | 3.42E-17 | -0.03762 | 0.004461 |
| rs13261666 | G | T | 0.522 | 3.90E-14 | -0.02689 | 0.003556 |
| rs12545053 | A | G | 0.397 | 2.43E-08 | 0.020281 | 0.003637 |
| rs10956809 | G | C | 0.442 | 6.32E-09 | -0.02078 | 0.00358 |
| rs1899896 | C | T | 0.286 | 1.04E-11 | 0.026448 | 0.003887 |
| rs4543592 | T | C | 0.468 | 7.46E-10 | 0.021931 | 0.003562 |
| rs10114490 | G | A | 0.198 | 1.81E-08 | -0.02551 | 0.004532 |
| rs2378662 | G | A | 0.556 | 4.16E-09 | 0.020948 | 0.003566 |
| rs10905461 | T | C | 0.718 | 7.35E-09 | -0.02396 | 0.004145 |
| rs10159545 | C | G | 0.375 | 1.84E-12 | 0.02625 | 0.003727 |
| rs7921378 | G | C | 0.463 | 8.26E-13 | -0.02546 | 0.003558 |
| rs12356821 | G | C | 0.14 | 6.27E-15 | 0.03937 | 0.005049 |
| rs9423279 | C | G | 0.641 | 3.21E-08 | -0.02051 | 0.003708 |
| rs4523689 | A | G | 0.408 | 1.55E-08 | -0.02061 | 0.003643 |
| rs6265 | C | T | 0.203 | 3.77E-12 | -0.03179 | 0.004578 |
| rs7929518 | A | G | 0.765 | 1.56E-08 | 0.024238 | 0.004285 |
| rs7938812 | T | G | 0.424 | 2.71E-33 | 0.043791 | 0.003637 |
| rs11057005 | A | G | 0.43 | 4.85E-09 | -0.02093 | 0.003579 |
| rs4759228 | G | C | 0.27 | 3.58E-08 | -0.02169 | 0.003934 |
| rs7969559 | A | G | 0.688 | 7.31E-10 | -0.02438 | 0.003959 |
| rs1971318 | C | T | 0.141 | 7.06E-09 | 0.028507 | 0.004925 |
| rs3904512 | G | A | 0.429 | 3.23E-09 | -0.02116 | 0.003577 |
| rs9540729 | A | T | 0.501 | 3.82E-08 | -0.01955 | 0.003558 |
| rs7322872 | C | T | 0.782 | 3.58E-09 | -0.02557 | 0.004335 |
| rs76214862 | A | C | 0.202 | 3.99E-08 | -0.02499 | 0.004547 |
| rs1435741 | G | A | 0.425 | 2.64E-16 | 0.029415 | 0.003591 |
| rs12441907 | C | A | 0.186 | 1.06E-10 | -0.02921 | 0.004523 |
| rs7197072 | C | T | 0.238 | 2.77E-09 | -0.02477 | 0.004169 |
| rs12923427 | C | T | 0.204 | 4.44E-08 | -0.02391 | 0.004372 |
| rs4785836 | T | C | 0.398 | 2.26E-08 | -0.02047 | 0.003659 |
| rs1050847 | C | T | 0.505 | 1.67E-09 | -0.02162 | 0.003589 |
| rs11658881 | A | G | 0.418 | 2.43E-08 | 0.020136 | 0.003611 |
| rs11078713 | A | G | 0.454 | 2.23E-08 | -0.02017 | 0.003606 |
| rs7224742 | C | T | 0.595 | 1.43E-08 | -0.02071 | 0.003655 |
| rs72896886 | G | C | 0.144 | 2.75E-08 | -0.02689 | 0.004837 |
| rs6508144 | C | G | 0.563 | 7.97E-09 | -0.02069 | 0.003586 |
| rs11872397 | G | A | 0.252 | 1.43E-09 | -0.02477 | 0.004095 |
| rs76608582 | C | A | 0.0389 | 1.94E-09 | -0.04956 | 0.00826 |
| rs1555445 | A | T | 0.337 | 3.65E-09 | 0.022555 | 0.003823 |
| rs56820925 | C | T | 0.347 | 1.73E-08 | -0.02186 | 0.003877 |
| rs117143374 | T | C | 0.12 | 2.76E-08 | 0.02929 | 0.005269 |
| rs134529 | T | C | 0.349 | 4.85E-08 | -0.01998 | 0.003661 |

**Table S20. Genome-wide significant and independent SNPs that were used as instruments for smoking heaviness**

| SNP | other | effect | eaf. | beta | se.exposure | pval.exposure |
| --- | --- | --- | --- | --- | --- | --- |
| rs2072659 | C | G | 0.105 | -0.06525 | 0.009247 | 1.71E-12 |
| rs2084533 | C | T | 0.319 | 0.033641 | 0.005901 | 1.22E-08 |
| rs7431710 | G | A | 0.644 | -0.03496 | 0.00581 | 1.82E-09 |
| rs11725618 | T | C | 0.287 | 0.036064 | 0.006158 | 4.67E-09 |
| rs787362 | T | A | 0.452 | 0.030477 | 0.005574 | 4.50E-08 |
| rs806798 | T | C | 0.543 | -0.03085 | 0.005532 | 2.48E-08 |
| rs215600 | G | A | 0.64 | -0.04925 | 0.005753 | 1.10E-17 |
| rs73229090 | C | A | 0.113 | 0.055489 | 0.008763 | 2.44E-10 |
| rs58379124 | T | C | 0.748 | 0.066939 | 0.006502 | 9.00E-25 |
| rs790564 | A | C | 0.719 | -0.04089 | 0.006193 | 3.97E-11 |
| rs3025383 | T | C | 0.18 | -0.05784 | 0.007045 | 2.22E-16 |
| rs7951365 | T | C | 0.306 | 0.038951 | 0.005968 | 6.63E-11 |
| rs75494138 | C | T | 0.0618 | 0.059876 | 0.010568 | 1.45E-08 |
| rs7928017 | C | A | 0.413 | -0.03293 | 0.005558 | 3.14E-09 |
| rs632811 | A | G | 0.351 | -0.03671 | 0.00641 | 1.03E-08 |
| rs11852372 | A | C | 0.328 | 0.182472 | 0.005911 | ###### |
| rs2386571 | A | C | 0.57 | -0.03187 | 0.005565 | 1.03E-08 |
| rs4785587 | G | A | 0.511 | -0.03362 | 0.005535 | 1.27E-09 |
| rs895330 | C | G | 0.206 | -0.039 | 0.007017 | 2.68E-08 |
| rs34406232 | C | A | 0.0259 | -0.14699 | 0.016697 | 1.33E-18 |
| rs56113850 | T | C | 0.568 | 0.107205 | 0.005604 | 1.10E-81 |
| rs2424888 | G | A | 0.405 | 0.033485 | 0.005636 | 2.76E-09 |
| rs2273500 | T | C | 0.159 | 0.068094 | 0.007796 | 2.47E-18 |

**Table S21. Genome-wide significant and independent SNPs that were used as instruments for alcohol consumption**

| SNP | other | effect | eaf. | pval. | beta.exposure | se.exposure |
| --- | --- | --- | --- | --- | --- | --- |
| rs10753661 | G | A | 0.702 | 4.24E-08 | -0.01137 | 0.002075 |
| rs28680958 | G | A | 0.23 | 9.78E-09 | -0.01358 | 0.002368 |
| rs1260326 | T | C | 0.595 | 3.33E-33 | 0.023812 | 0.001984 |
| rs62135521 | G | T | 0.0378 | 9.91E-09 | -0.02636 | 0.004595 |
| rs528301 | G | A | 0.605 | 1.25E-15 | 0.015592 | 0.001949 |
| rs6739804 | T | C | 0.66 | 4.72E-10 | -0.01297 | 0.002082 |
| rs4233567 | C | T | 0.34 | 3.83E-10 | -0.01259 | 0.002011 |
| rs28732378 | A | G | 0.729 | 2.24E-14 | -0.01673 | 0.002191 |
| rs28712821 | G | A | 0.594 | 1.10E-46 | 0.028334 | 0.001974 |
| rs16854020 | G | A | 0.127 | 4.82E-10 | 0.018083 | 0.002907 |
| rs1229984 | T | C | 0.953 | ###### | 0.188115 | 0.006179 |
| rs78234152 | G | A | 0.0986 | 2.18E-19 | 0.027654 | 0.003071 |
| rs13107325 | C | T | 0.0654 | 1.23E-20 | -0.03645 | 0.003913 |
| rs331939 | G | A | 0.339 | 4.50E-09 | -0.0119 | 0.002029 |
| rs4916723 | A | C | 0.404 | 8.07E-09 | -0.01127 | 0.001953 |
| rs55872084 | G | T | 0.218 | 1.98E-08 | 0.012731 | 0.002268 |
| rs10085696 | A | G | 0.201 | 1.24E-10 | -0.01605 | 0.002495 |
| rs2299409 | G | A | 0.493 | 4.80E-08 | -0.01055 | 0.001933 |
| rs6951574 | T | C | 0.459 | 4.44E-11 | 0.012753 | 0.001936 |
| rs28601761 | C | G | 0.405 | 7.60E-09 | 0.011299 | 0.001955 |
| rs55932213 | A | G | 0.701 | 1.80E-08 | 0.012477 | 0.002216 |
| rs2049045 | G | C | 0.189 | 3.97E-08 | -0.01377 | 0.002505 |
| rs4752999 | C | T | 0.321 | 2.03E-12 | -0.01456 | 0.00207 |
| rs4309187 | A | C | 0.697 | 1.37E-12 | 0.014791 | 0.002088 |
| rs17542254 | A | G | 0.251 | 8.96E-10 | 0.013142 | 0.002146 |
| rs1387766 | G | A | 0.622 | 4.79E-08 | -0.01083 | 0.001983 |
| rs34704785 | C | T | 0.412 | 4.52E-08 | -0.01058 | 0.001935 |
| rs1123285 | C | G | 0.339 | 1.36E-09 | -0.01242 | 0.00205 |
| rs28929474 | C | T | 0.0154 | 2.39E-11 | -0.04768 | 0.00714 |
| rs153106 | T | C | 0.409 | 3.63E-12 | -0.01361 | 0.001959 |
| rs79616692 | G | C | 0.11 | 2.38E-09 | 0.018809 | 0.003152 |
| rs11860773 | T | C | 0.176 | 8.35E-10 | -0.01501 | 0.002444 |
| rs13332432 | C | G | 0.296 | 5.94E-11 | 0.014005 | 0.002141 |
| rs34121753 | A | G | 0.532 | 1.39E-08 | 0.011069 | 0.001951 |
| rs76640332 | G | A | 0.204 | 1.47E-18 | -0.02101 | 0.002389 |
| rs838145 | G | A | 0.584 | 3.87E-16 | -0.0158 | 0.00194 |
| rs6106989 | G | A | 0.628 | 3.81E-08 | 0.010899 | 0.001983 |

**Table S22. Genome-wide significant and independent SNPs that were used as instruments for coffee intake**

| SNP | effect | other | eaf. | beta | se. | pval. |
| --- | --- | --- | --- | --- | --- | --- |
| rs10489219 | G | A | 0.095253 | 0.01758 | 0.003124 | 1.82E-08 |
| rs10127720 | T | C | 0.260105 | 0.014183 | 0.00209 | 1.15E-11 |
| rs1260326 | T | C | 0.392544 | 0.013595 | 0.00187 | 3.59E-13 |
| rs13387090 | G | T | 0.169111 | -0.01694 | 0.002446 | 4.36E-12 |
| rs12514566 | G | A | 0.336 | -0.01073 | 0.001933 | 2.84E-08 |
| rs3814424 | C | T | 0.156617 | 0.020359 | 0.002515 | 5.78E-16 |
| rs597045 | A | T | 0.305105 | -0.01106 | 0.002009 | 3.71E-08 |
| rs67595273 | A | C | 0.174647 | -0.01405 | 0.00244 | 8.61E-09 |
| rs9398171 | C | T | 0.287853 | 0.011618 | 0.00202 | 8.82E-09 |
| rs570263 | T | C | 0.369047 | -0.01069 | 0.001894 | 1.65E-08 |
| rs2465037 | C | A | 0.342263 | -0.01139 | 0.001933 | 3.80E-09 |
| rs4410790 | T | C | 0.36449 | 0.039316 | 0.001898 | 3.23E-95 |
| rs73075167 | A | T | 0.129804 | -0.01573 | 0.002761 | 1.23E-08 |
| rs7811609 | C | T | 0.374197 | 0.010462 | 0.001887 | 2.93E-08 |
| rs34060476 | A | G | 0.134758 | 0.019823 | 0.002678 | 1.33E-13 |
| rs1057868 | C | T | 0.285308 | 0.020698 | 0.002021 | 1.30E-24 |
| rs13271359 | C | T | 0.259578 | -0.01202 | 0.002098 | 1.01E-08 |
| rs2472297 | C | T | 0.26772 | 0.047203 | 0.002059 | ###### |
| rs56094641 | A | G | 0.403264 | 0.018814 | 0.001863 | 5.54E-24 |
| rs4925114 | A | G | 0.343713 | 0.011986 | 0.001931 | 5.36E-10 |
| rs57918684 | G | A | 0.154859 | 0.014161 | 0.002531 | 2.22E-08 |
| rs66723169 | C | A | 0.230866 | 0.017077 | 0.002174 | 4.04E-15 |
| rs56113850 | T | C | 0.422344 | 0.012731 | 0.001849 | 5.73E-12 |
| rs6062682 | C | T | 0.465213 | 0.01136 | 0.001856 | 9.28E-10 |
| rs5760378 | A | C | 0.224457 | -0.01457 | 0.002188 | 2.79E-11 |

**Table S23. Genome-wide significant and independent SNPs that were used as instruments for tea intake**

| SNP | effect_ | other | eaf. | beta. | se | pval.exposure |
| --- | --- | --- | --- | --- | --- | --- |
| rs11204711 | A | G | 0.383748 | 0.015488 | 0.002497 | 5.53E-10 |
| rs11487328 | G | C | 0.379686 | -0.01486 | 0.002473 | 1.87E-09 |
| rs182050989 | C | T | 0.028533 | -0.0396 | 0.007189 | 3.62E-08 |
| rs1156588 | A | G | 0.210625 | -0.01599 | 0.002931 | 4.87E-08 |
| rs2071207 | T | C | 0.474531 | -0.01322 | 0.002389 | 3.16E-08 |
| rs2117137 | A | G | 0.405401 | 0.014377 | 0.00243 | 3.28E-09 |
| rs1481012 | A | G | 0.113206 | -0.02577 | 0.003771 | 8.27E-12 |
| rs185115295 | T | C | 0.271365 | -0.01801 | 0.002686 | 2.02E-11 |
| rs11022751 | T | C | 0.269456 | 0.01748 | 0.002696 | 9.00E-11 |
| rs977474 | C | T | 0.166751 | 0.021052 | 0.003215 | 5.86E-11 |
| rs2465018 | G | A | 0.229969 | 0.023435 | 0.002852 | 2.10E-16 |
| rs713598 | C | G | 0.400683 | 0.013972 | 0.002433 | 9.40E-09 |
| rs4410790 | T | C | 0.365419 | 0.03893 | 0.00248 | 1.58E-55 |
| rs6462899 | T | A | 0.376239 | 0.013743 | 0.002471 | 2.68E-08 |
| rs6467958 | T | C | 0.266624 | -0.0225 | 0.0027 | 7.88E-17 |
| rs12591786 | C | T | 0.157503 | -0.02063 | 0.003323 | 5.35E-10 |
| rs57292194 | T | C | 0.068547 | 0.026474 | 0.004729 | 2.16E-08 |
| rs2472297 | C | T | 0.266904 | 0.053514 | 0.002693 | 8.13E-88 |
| rs4808940 | C | G | 0.335149 | 0.015286 | 0.002534 | 1.61E-09 |
| rs2273447 | A | T | 0.204734 | 0.019982 | 0.002962 | 1.51E-11 |
| rs4817505 | T | C | 0.393271 | 0.014568 | 0.002449 | 2.70E-09 |
| rs9624470 | G | A | 0.419467 | 0.025493 | 0.002428 | 8.84E-26 |
| rs73424602 | C | T | 0.401794 | -0.01511 | 0.002432 | 5.27E-10 |

**Table S24. Genome-wide significant and independent SNPs that were used as instruments for insomnia**

| SNP | effect | other | eaf. | beta. | se | pval. |
| --- | --- | --- | --- | --- | --- | --- |
| rs113851554 | T | G | 0.0506 | 0.206201 | 0.014 | 1.56E-51 |
| rs1064939 | A | T | 0.9784 | 0.130151 | 0.02 | 2.16E-10 |
| rs72899452 | T | C | 0.0648 | 0.074179 | 0.012 | 1.00E-09 |
| rs55972276 | A | C | 0.1366 | 0.07325 | 0.009 | 4.19E-17 |
| rs77641763 | T | C | 0.122 | 0.07139 | 0.009 | 6.53E-15 |
| rs2286729 | A | G | 0.0862 | 0.069526 | 0.011 | 5.37E-11 |
| rs118166957 | T | C | 0.1591 | 0.067659 | 0.008 | 1.95E-16 |
| rs62158170 | A | G | 0.7856 | 0.065788 | 0.007 | 1.20E-19 |
| rs62264767 | A | C | 0.8531 | 0.064851 | 0.008 | 1.63E-14 |
| rs699844 | A | G | 0.9195 | 0.060154 | 0.011 | 4.11E-08 |
| rs28611339 | T | G | 0.1282 | 0.058269 | 0.009 | 8.46E-11 |
| rs1015438 | A | G | 0.1882 | 0.058269 | 0.008 | 2.51E-14 |
| rs6465151 | T | C | 0.1134 | 0.05638 | 0.009 | 1.90E-09 |
| rs16903122 | T | C | 0.2487 | 0.055435 | 0.007 | 9.04E-16 |
| rs34490907 | C | G | 0.8878 | 0.053541 | 0.009 | 1.76E-08 |
| rs1927902 | T | C | 0.2542 | 0.052592 | 0.007 | 1.15E-14 |
| rs1620977 | A | G | 0.2696 | 0.051643 | 0.007 | 2.27E-14 |
| rs11756035 | C | G | 0.1281 | 0.050693 | 0.009 | 1.29E-08 |
| rs62429521 | A | C | 0.1456 | 0.050693 | 0.008 | 1.78E-09 |
| rs908668 | T | C | 0.2081 | 0.049742 | 0.007 | 1.41E-11 |
| rs35322724 | A | C | 0.5774 | 0.04879 | 0.006 | 3.75E-16 |
| rs62068188 | T | C | 0.8339 | 0.04879 | 0.008 | 1.18E-09 |
| rs9931543 | T | C | 0.7359 | 0.047837 | 0.007 | 1.11E-12 |
| rs17223714 | A | G | 0.7885 | 0.045929 | 0.007 | 2.44E-10 |
| rs429358 | T | C | 0.8458 | 0.045929 | 0.008 | 2.13E-08 |
| rs12310246 | A | G | 0.2489 | 0.044973 | 0.007 | 4.74E-11 |
| rs830716 | C | G | 0.7133 | 0.044973 | 0.007 | 8.68E-12 |
| rs116466468 | T | C | 0.7593 | 0.044017 | 0.007 | 2.11E-10 |
| rs742760 | A | T | 0.8155 | 0.043059 | 0.008 | 2.48E-08 |
| rs10800992 | T | C | 0.4431 | 0.042101 | 0.006 | 3.84E-12 |
| rs55772859 | A | C | 0.3106 | 0.042101 | 0.006 | 4.82E-11 |
| rs17005118 | A | G | 0.264 | 0.042101 | 0.007 | 6.13E-10 |
| rs35539975 | A | G | 0.7785 | 0.042101 | 0.007 | 4.49E-09 |
| rs12666306 | A | G | 0.5017 | 0.042101 | 0.006 | 2.24E-12 |
| rs12030482 | A | T | 0.2201 | 0.041142 | 0.007 | 8.16E-09 |
| rs17025198 | A | G | 0.2043 | 0.041142 | 0.007 | 2.19E-08 |
| rs715338 | A | G | 0.5776 | 0.041142 | 0.006 | 7.85E-12 |
| rs12991815 | C | G | 0.4241 | 0.040182 | 0.006 | 3.02E-11 |
| rs4592425 | T | G | 0.6965 | 0.040182 | 0.006 | 4.31E-10 |
| rs11149313 | A | G | 0.7297 | 0.040182 | 0.007 | 2.38E-09 |
| rs6019663 | T | C | 0.293 | 0.040182 | 0.007 | 6.47E-10 |
| rs6808140 | T | C | 0.5053 | 0.039221 | 0.006 | 5.35E-11 |
| rs35110063 | A | G | 0.4266 | 0.039221 | 0.006 | 8.82E-11 |
| rs1147852 | A | G | 0.3095 | 0.039221 | 0.006 | 9.94E-10 |
| rs324017 | A | C | 0.294 | 0.039221 | 0.007 | 1.61E-09 |
| rs6562066 | T | C | 0.3688 | 0.039221 | 0.006 | 1.38E-10 |
| rs1038093 | T | C | 0.6282 | 0.039221 | 0.006 | 2.47E-10 |
| rs11090039 | A | G | 0.2871 | 0.039221 | 0.007 | 1.82E-09 |
| rs6888135 | A | C | 0.4965 | 0.038259 | 0.006 | 1.21E-10 |
| rs6756610 | C | G | 0.6291 | 0.037296 | 0.006 | 1.14E-09 |
| rs7040224 | A | G | 0.3161 | 0.037296 | 0.006 | 4.24E-09 |
| rs5877 | T | C | 0.6691 | 0.036332 | 0.006 | 1.23E-08 |
| rs1530938 | A | G | 0.4423 | 0.036332 | 0.006 | 8.82E-10 |
| rs10756571 | T | C | 0.6853 | 0.036332 | 0.006 | 1.80E-08 |
| rs2221119 | C | G | 0.443 | 0.036332 | 0.006 | 2.00E-09 |
| rs34967082 | A | G | 0.4136 | 0.035367 | 0.006 | 4.34E-09 |
| rs2216427 | C | G | 0.6527 | 0.035367 | 0.006 | 1.60E-08 |
| rs6601080 | A | G | 0.6762 | 0.035367 | 0.006 | 2.21E-08 |
| rs2598293 | T | C | 0.4763 | 0.035367 | 0.006 | 2.48E-09 |
| rs871994 | A | C | 0.4352 | 0.035367 | 0.006 | 5.50E-09 |
| rs1167132 | T | C | 0.3917 | 0.035367 | 0.006 | 8.73E-09 |
| rs176644 | T | G | 0.4036 | 0.035367 | 0.006 | 9.49E-09 |
| rs12605642 | T | G | 0.4864 | 0.035367 | 0.006 | 2.13E-09 |
| rs9964420 | A | C | 0.301 | 0.035367 | 0.007 | 4.54E-08 |
| rs72820274 | A | G | 0.417 | 0.034401 | 0.006 | 1.28E-08 |
| rs13138995 | A | G | 0.3896 | 0.034401 | 0.006 | 1.97E-08 |
| rs2030672 | C | G | 0.5589 | 0.034401 | 0.006 | 1.10E-08 |
| rs10898940 | A | C | 0.5173 | 0.034401 | 0.006 | 8.09E-09 |
| rs1567084 | A | G | 0.4981 | 0.033435 | 0.006 | 2.14E-08 |
| rs1580173 | A | G | 0.5608 | 0.033435 | 0.006 | 2.28E-08 |
| rs4588900 | A | G | 0.5164 | 0.033435 | 0.006 | 1.57E-08 |
| rs28552587 | A | G | 0.5641 | 0.033435 | 0.006 | 3.30E-08 |
| rs10955647 | T | G | 0.5321 | 0.033435 | 0.006 | 1.84E-08 |
| rs667730 | T | C | 0.5788 | 0.033435 | 0.006 | 2.26E-08 |
| rs647905 | T | C | 0.5409 | 0.033435 | 0.006 | 2.87E-08 |
| rs4858708 | A | T | 0.5305 | -0.03356 | 0.006 | 1.23E-08 |
| rs2364921 | T | C | 0.4691 | -0.03356 | 0.006 | 2.13E-08 |
| rs190073 | A | G | 0.4141 | -0.03356 | 0.006 | 2.86E-08 |
| rs11588755 | A | G | 0.522 | -0.03459 | 0.006 | 5.14E-09 |
| rs11119409 | T | C | 0.5866 | -0.03459 | 0.006 | 1.19E-08 |
| rs728017 | A | G | 0.3864 | -0.03459 | 0.006 | 9.51E-09 |
| rs1731951 | A | T | 0.4434 | -0.03459 | 0.006 | 1.36E-08 |
| rs4788203 | A | G | 0.4334 | -0.03459 | 0.006 | 6.32E-09 |
| rs910187 | A | G | 0.373 | -0.03459 | 0.006 | 1.63E-08 |
| rs12520974 | T | C | 0.4846 | -0.03563 | 0.006 | 1.69E-09 |
| rs701394 | A | G | 0.6376 | -0.03563 | 0.006 | 6.83E-09 |
| rs17367725 | T | C | 0.3513 | -0.03563 | 0.006 | 9.29E-09 |
| rs10758593 | A | G | 0.3989 | -0.03563 | 0.006 | 4.90E-09 |
| rs7402939 | T | C | 0.376 | -0.03563 | 0.006 | 5.19E-09 |
| rs2838787 | A | G | 0.3924 | -0.03563 | 0.006 | 7.65E-09 |
| rs6702604 | A | G | 0.5843 | -0.03666 | 0.006 | 1.30E-09 |
| rs823247 | T | C | 0.479 | -0.03666 | 0.006 | 5.25E-10 |
| rs7599697 | T | C | 0.3583 | -0.03666 | 0.006 | 5.00E-09 |
| rs2388840 | A | G | 0.5757 | -0.03666 | 0.006 | 1.37E-09 |
| rs7475916 | C | G | 0.3533 | -0.03666 | 0.006 | 6.70E-09 |
| rs4767645 | T | G | 0.4614 | -0.03666 | 0.006 | 6.47E-10 |
| rs6510033 | A | G | 0.7253 | -0.03666 | 0.007 | 4.66E-08 |
| rs10944696 | A | G | 0.2978 | -0.0377 | 0.007 | 7.99E-09 |
| rs6973090 | A | G | 0.25 | -0.0377 | 0.007 | 4.31E-08 |
| rs671985 | A | G | 0.4516 | -0.0377 | 0.006 | 2.79E-10 |
| rs11001276 | A | T | 0.74 | -0.0377 | 0.007 | 2.52E-08 |
| rs214934 | A | T | 0.3123 | -0.0377 | 0.006 | 3.16E-09 |
| rs6589988 | A | G | 0.6757 | -0.0377 | 0.006 | 4.70E-09 |
| rs1536053 | T | C | 0.3157 | -0.0377 | 0.006 | 6.04E-09 |
| rs3184470 | A | G | 0.3507 | -0.0377 | 0.006 | 9.73E-10 |
| rs8076183 | T | C | 0.4485 | -0.0377 | 0.006 | 2.75E-10 |
| rs7571486 | A | G | 0.251 | -0.03874 | 0.007 | 1.40E-08 |
| rs4502882 | T | C | 0.6578 | -0.03874 | 0.006 | 7.96E-10 |
| rs12251016 | A | T | 0.6559 | -0.03874 | 0.006 | 3.89E-10 |
| rs224029 | T | C | 0.3995 | -0.03874 | 0.006 | 2.51E-10 |
| rs566673 | T | G | 0.5351 | -0.03874 | 0.006 | 1.18E-10 |
| rs521484 | A | G | 0.7668 | -0.03978 | 0.007 | 1.53E-08 |
| rs2389631 | A | C | 0.6666 | -0.03978 | 0.006 | 2.03E-10 |
| rs2089358 | T | C | 0.7038 | -0.04082 | 0.007 | 2.75E-10 |
| rs1289939 | T | C | 0.2325 | -0.04082 | 0.007 | 6.00E-09 |
| rs11803128 | A | G | 0.6541 | -0.04082 | 0.006 | 6.85E-11 |
| rs4664299 | T | C | 0.2349 | -0.04082 | 0.007 | 4.95E-09 |
| rs3774751 | T | G | 0.4621 | -0.04082 | 0.006 | 7.32E-12 |
| rs7044885 | C | G | 0.4417 | -0.04082 | 0.006 | 5.67E-12 |
| rs6734957 | T | G | 0.2388 | -0.04186 | 0.007 | 1.82E-09 |
| rs62301574 | C | G | 0.7996 | -0.04186 | 0.007 | 1.37E-08 |
| rs9889282 | A | C | 0.6129 | -0.04186 | 0.006 | 4.70E-12 |
| rs314281 | T | C | 0.4531 | -0.04291 | 0.006 | 6.03E-13 |
| rs10761240 | A | G | 0.3963 | -0.04291 | 0.006 | 2.12E-12 |
| rs12912299 | T | C | 0.4893 | -0.04291 | 0.006 | 4.42E-13 |
| rs60565673 | T | G | 0.6211 | -0.04291 | 0.006 | 1.59E-12 |
| rs12983032 | A | G | 0.3434 | -0.04291 | 0.006 | 1.07E-11 |
| rs694786 | T | C | 0.4605 | -0.04395 | 0.006 | 1.97E-13 |
| rs17083297 | A | C | 0.1766 | -0.04395 | 0.008 | 1.60E-08 |
| rs6967168 | T | G | 0.7544 | -0.04395 | 0.007 | 1.39E-10 |
| rs524859 | A | G | 0.3601 | -0.04395 | 0.006 | 1.48E-12 |
| rs61921611 | T | C | 0.692 | -0.04395 | 0.006 | 7.84E-12 |
| rs7214267 | A | G | 0.581 | -0.04395 | 0.006 | 5.09E-13 |
| rs11605348 | A | G | 0.3495 | -0.045 | 0.006 | 7.01E-13 |
| rs16990210 | T | C | 0.8478 | -0.04604 | 0.008 | 1.97E-08 |
| rs10947690 | A | G | 0.7408 | -0.04709 | 0.007 | 4.04E-12 |
| rs4702 | A | G | 0.5562 | -0.04814 | 0.006 | 6.78E-16 |
| rs8180817 | C | G | 0.4304 | -0.04919 | 0.006 | 1.83E-16 |
| rs76145129 | T | G | 0.1238 | -0.05024 | 0.009 | 2.73E-08 |
| rs1031654 | A | C | 0.7996 | -0.05129 | 0.007 | 3.88E-12 |
| rs2431108 | T | C | 0.672 | -0.0534 | 0.006 | 7.83E-17 |
| rs4709655 | T | C | 0.1191 | -0.05446 | 0.009 | 3.09E-09 |
| rs28582096 | A | G | 0.205 | -0.05446 | 0.007 | 1.74E-13 |
| rs4981170 | A | G | 0.1943 | -0.05446 | 0.008 | 7.33E-13 |
| rs72657797 | T | C | 0.1759 | -0.05551 | 0.008 | 1.52E-12 |
| rs8180457 | T | C | 0.1573 | -0.05551 | 0.008 | 1.12E-11 |
| rs73671843 | A | G | 0.1257 | -0.05551 | 0.009 | 5.49E-10 |
| rs13010288 | T | G | 0.1326 | -0.05975 | 0.009 | 9.26E-12 |
| rs62383308 | A | G | 0.0805 | -0.05975 | 0.011 | 3.98E-08 |
| rs17643634 | T | C | 0.165 | -0.05975 | 0.008 | 1.34E-13 |
| rs66674044 | A | T | 0.8573 | -0.05975 | 0.009 | 2.18E-12 |
| rs6119267 | C | G | 0.6891 | -0.05975 | 0.006 | 2.32E-20 |
| rs492858 | T | C | 0.0759 | -0.06614 | 0.011 | 3.46E-09 |
| rs10947428 | T | C | 0.7858 | -0.06828 | 0.007 | 9.06E-21 |
| rs79693059 | C | G | 0.9158 | -0.07257 | 0.011 | 1.61E-11 |
| rs9527083 | A | G | 0.6705 | -0.0758 | 0.006 | 1.61E-32 |
| rs13135092 | A | G | 0.9175 | -0.08883 | 0.011 | 2.53E-16 |
| rs17520265 | A | G | 0.0342 | -0.09102 | 0.016 | 2.87E-08 |

**Table S25. Genome-wide significant and independent SNPs that were used as instruments for overall sleep duration**

| SNP | effect | other | eaf. | beta | se. | pval. |
| --- | --- | --- | --- | --- | --- | --- |
| rs915416 | C | G | 0.289947 | 0.019259 | 0.002495 | 9.90E-15 |
| rs269054 | T | A | 0.577924 | -0.01364 | 0.002293 | 2.10E-09 |
| rs61796569 | C | T | 0.730417 | -0.01544 | 0.002564 | 1.50E-09 |
| rs12567114 | G | A | 0.724198 | -0.01483 | 0.00254 | 4.30E-09 |
| rs62120041 | T | C | 0.933902 | 0.026111 | 0.004575 | 9.60E-09 |
| rs374153 | C | T | 0.158085 | 0.017612 | 0.003103 | 9.10E-09 |
| rs2717076 | C | T | 0.372584 | -0.01841 | 0.002341 | 3.10E-15 |
| rs75539574 | A | C | 0.914208 | -0.03625 | 0.004065 | 6.90E-19 |
| rs7556815 | G | A | 0.780856 | -0.04072 | 0.00274 | 1.30E-49 |
| rs35662245 | T | A | 0.661256 | -0.0146 | 0.002393 | 1.40E-09 |
| rs11885663 | C | T | 0.752191 | -0.01622 | 0.002618 | 8.60E-10 |
| rs10173260 | T | C | 0.393765 | -0.01284 | 0.002313 | 2.90E-08 |
| rs112230981 | A | G | 0.94984 | 0.031528 | 0.005228 | 2.20E-09 |
| rs17732997 | C | G | 0.569098 | 0.012935 | 0.002288 | 1.20E-08 |
| rs7644809 | T | C | 0.421606 | 0.013062 | 0.002301 | 1.60E-08 |
| rs13088093 | T | G | 0.663683 | -0.01627 | 0.002402 | 7.00E-12 |
| rs2192528 | A | G | 0.480065 | 0.013369 | 0.002269 | 2.70E-09 |
| rs17427571 | A | G | 0.684313 | 0.013826 | 0.002435 | 1.30E-08 |
| rs35531607 | T | C | 0.525917 | -0.01284 | 0.002273 | 1.50E-08 |
| rs13109404 | T | G | 0.928024 | 0.031204 | 0.004408 | 1.40E-12 |
| rs365663 | A | G | 0.545963 | 0.014629 | 0.002279 | 1.00E-10 |
| rs465700 | C | G | 0.107834 | 0.022515 | 0.003674 | 1.40E-09 |
| rs56372231 | C | T | 0.665907 | -0.01694 | 0.0024 | 2.20E-12 |
| rs11567976 | C | T | 0.429092 | -0.0128 | 0.002285 | 2.10E-08 |
| rs151014368 | G | A | 0.793742 | -0.01609 | 0.00282 | 9.10E-09 |
| rs34556183 | A | G | 0.719606 | 0.016923 | 0.002523 | 2.30E-11 |
| rs113113059 | T | C | 0.78 | 0.016141 | 0.002737 | 8.40E-09 |
| rs9382445 | T | C | 0.62305 | 0.014536 | 0.002334 | 4.80E-10 |
| rs2231265 | A | G | 0.227711 | -0.01496 | 0.002699 | 2.70E-08 |
| rs9345234 | A | C | 0.421984 | -0.01301 | 0.002299 | 1.80E-08 |
| rs34731055 | C | T | 0.81911 | -0.01946 | 0.002948 | 3.70E-11 |
| rs2079070 | C | G | 0.264613 | 0.017548 | 0.002566 | 7.50E-12 |
| rs7806045 | T | C | 0.754703 | 0.014792 | 0.002626 | 1.40E-08 |
| rs4841498 | C | T | 0.48696 | 0.013995 | 0.002269 | 1.20E-09 |
| rs73219758 | G | A | 0.708064 | 0.016401 | 0.002495 | 5.60E-11 |
| rs10973207 | G | T | 0.842323 | -0.02043 | 0.003124 | 6.00E-11 |
| rs1776776 | T | C | 0.873832 | 0.019963 | 0.003411 | 4.90E-09 |
| rs12246842 | A | G | 0.459815 | 0.013395 | 0.002274 | 3.90E-09 |
| rs10761674 | C | T | 0.477334 | 0.012333 | 0.002266 | 4.20E-08 |
| rs11190970 | G | A | 0.798661 | 0.015379 | 0.002823 | 4.60E-08 |
| rs7915425 | T | C | 0.174682 | 0.019064 | 0.00299 | 2.00E-10 |
| rs1517572 | A | C | 0.419464 | -0.01464 | 0.002295 | 1.50E-10 |
| rs4592416 | A | G | 0.535593 | -0.01468 | 0.00227 | 9.30E-11 |
| rs11039544 | G | A | 0.837779 | 0.018389 | 0.003074 | 1.90E-09 |
| rs174560 | T | C | 0.685785 | -0.01358 | 0.002437 | 2.80E-08 |
| rs12791153 | A | T | 0.918911 | -0.02355 | 0.004217 | 1.90E-08 |
| rs1553132 | A | G | 0.741567 | -0.01451 | 0.002584 | 2.50E-08 |
| rs1939455 | G | T | 0.879446 | 0.020425 | 0.003561 | 1.20E-08 |
| rs1079727 | T | C | 0.842182 | -0.01829 | 0.003103 | 5.30E-09 |
| rs3751046 | A | G | 0.853507 | -0.01941 | 0.003208 | 1.10E-09 |
| rs34354917 | C | A | 0.710472 | 0.013746 | 0.002501 | 3.90E-08 |
| rs4767550 | A | G | 0.585862 | -0.0143 | 0.00231 | 6.30E-10 |
| rs6575005 | T | C | 0.757854 | 0.015564 | 0.002642 | 4.40E-09 |
| rs10483350 | A | G | 0.804582 | -0.01737 | 0.002868 | 1.50E-09 |
| rs61985058 | C | T | 0.856824 | -0.01859 | 0.003229 | 1.30E-08 |
| rs55658675 | C | T | 0.644938 | 0.013142 | 0.002369 | 2.00E-08 |
| rs11621908 | C | T | 0.917141 | 0.024095 | 0.004163 | 5.60E-09 |
| rs8038326 | A | G | 0.72691 | 0.01592 | 0.002541 | 2.80E-10 |
| rs3095508 | C | A | 0.593529 | 0.015352 | 0.002304 | 3.10E-11 |
| rs11643715 | C | G | 0.709058 | -0.0139 | 0.002497 | 3.20E-08 |
| rs9937053 | G | A | 0.576695 | 0.016935 | 0.00229 | 1.20E-13 |
| rs7198661 | T | C | 0.502318 | 0.013383 | 0.002274 | 3.90E-09 |
| rs3027234 | C | T | 0.772849 | 0.015154 | 0.002706 | 2.30E-08 |
| rs205024 | C | T | 0.616265 | -0.01383 | 0.002327 | 3.90E-09 |
| rs8072993 | T | G | 0.363492 | -0.01748 | 0.002822 | 4.20E-10 |
| rs147114641 | C | A | 0.773722 | 0.01598 | 0.002704 | 3.10E-09 |
| rs2696429 | G | A | 0.773626 | 0.016888 | 0.002708 | 4.00E-10 |
| rs12607679 | T | C | 0.737717 | 0.020139 | 0.002593 | 8.30E-15 |
| rs10421649 | T | A | 0.44303 | -0.0133 | 0.002295 | 6.90E-09 |
| rs2072727 | T | C | 0.43617 | 0.013243 | 0.002285 | 7.90E-09 |

**Table S26. Genome-wide significant and independent SNPs that were used as instruments for short sleep duration**

| SNP | effect | other | eaf. | beta. | se. | pval. |
| --- | --- | --- | --- | --- | --- | --- |
| rs7524118 | T | C | 0.291624 | -0.00576 | 0.001054 | 4.90E-08 |
| rs2186122 | A | T | 0.438434 | -0.00567 | 0.000972 | 4.80E-09 |
| rs12567114 | G | A | 0.7246 | 0.006325 | 0.001077 | 4.10E-09 |
| rs2820313 | A | G | 0.658888 | -0.00601 | 0.00101 | 2.30E-09 |
| rs1380703 | A | G | 0.616469 | -0.00676 | 0.001005 | 1.60E-11 |
| rs2863957 | C | A | 0.781508 | 0.01019 | 0.001161 | 2.60E-18 |
| rs2014830 | C | T | 0.698128 | 0.005786 | 0.00105 | 2.70E-08 |
| rs17005118 | G | A | 0.735064 | -0.00648 | 0.001087 | 2.50E-09 |
| rs13107325 | C | T | 0.925472 | -0.01327 | 0.001828 | 2.50E-13 |
| rs12518468 | T | C | 0.671544 | -0.00589 | 0.001021 | 8.50E-09 |
| rs3776864 | A | C | 0.66721 | 0.005724 | 0.001019 | 1.70E-08 |
| rs4585442 | A | G | 0.688977 | -0.00635 | 0.001036 | 8.10E-10 |
| rs2517827 | C | A | 0.688933 | -0.00593 | 0.001036 | 5.70E-09 |
| rs12661667 | C | T | 0.736505 | -0.00602 | 0.001087 | 2.80E-08 |
| rs9367621 | T | A | 0.43104 | 0.005445 | 0.00097 | 1.60E-08 |
| rs9321171 | C | T | 0.540122 | 0.005354 | 0.000966 | 4.20E-08 |
| rs11763750 | G | A | 0.814346 | 0.007212 | 0.001234 | 5.10E-09 |
| rs1229762 | C | T | 0.335499 | -0.00724 | 0.001017 | 1.00E-12 |
| rs60882754 | A | T | 0.938985 | 0.011304 | 0.002001 | 1.80E-08 |
| rs1607227 | G | T | 0.704938 | 0.006369 | 0.001055 | 1.50E-09 |
| rs7939345 | T | G | 0.207569 | 0.006498 | 0.001182 | 4.00E-08 |
| rs17388803 | A | C | 0.894352 | -0.00983 | 0.001587 | 6.50E-10 |
| rs59779556 | T | G | 0.553827 | 0.005491 | 0.000966 | 2.00E-08 |
| rs205024 | C | T | 0.616724 | 0.00551 | 0.000986 | 2.70E-08 |
| rs12963463 | C | T | 0.299425 | 0.007114 | 0.00106 | 1.90E-11 |
| rs5757675 | G | T | 0.259528 | 0.006455 | 0.001099 | 2.70E-09 |

**Table S27. Genome-wide significant and independent SNPs that were used as instruments for long sleep duration**

| SNP | effect | other | eaf | beta | se | pval |
| --- | --- | --- | --- | --- | --- | --- |
| rs7534398 | T | A | 0.798618 | -0.00508 | 0.000908 | 2.10E-08 |
| rs6737318 | A | G | 0.778159 | -0.00638 | 0.000877 | 3.40E-13 |
| rs10899257 | G | A | 0.855527 | -0.00564 | 0.001031 | 4.60E-08 |
| rs75458655 | C | T | 0.977027 | -0.01673 | 0.002423 | 5.40E-12 |
| rs3751046 | A | G | 0.852658 | -0.00577 | 0.001027 | 2.00E-08 |
| rs17817288 | A | G | 0.518127 | 0.004188 | 0.000727 | 8.90E-09 |
| rs147114641 | C | A | 0.77403 | 0.005689 | 0.000868 | 4.90E-11 |
| rs146467757 | G | T | 0.772153 | 0.005638 | 0.00087 | 8.10E-11 |
| rs572857764 | T | A | 0.775271 | 0.005627 | 0.000878 | 1.30E-10 |
| rs62073915 | A | G | 0.77868 | 0.006056 | 0.000891 | 1.00E-11 |

**Table S28. Genome-wide significant and independent SNPs that were used as instruments for educational level**

| SNP | effect | other_ | eaf | beta | se | pval |
| --- | --- | --- | --- | --- | --- | --- |
| rs13090388 | C | T | 0.6905 | -0.02852 | 0.00184 | 4.29E-54 |
| rs7029718 | G | A | 0.5646 | -0.02439 | 0.00174 | 1.85E-44 |
| rs9372625 | A | G | 0.4133 | 0.02383 | 0.00176 | 6.76E-42 |
| rs1334297 | A | G | 0.784 | 0.02449 | 0.00192 | 3.06E-37 |
| rs4700393 | G | A | 0.5289 | 0.02086 | 0.0017 | 1.51E-34 |
| rs11123818 | G | A | 0.6054 | -0.02081 | 0.00175 | 1.72E-32 |
| rs34316 | C | A | 0.5799 | -0.02016 | 0.00177 | 3.35E-30 |
| rs10773002 | T | A | 0.7211 | -0.02191 | 0.00197 | 8.68E-29 |
| rs9964724 | C | T | 0.3401 | -0.01978 | 0.00183 | 2.66E-27 |
| rs3747631 | G | C | 0.7721 | -0.02207 | 0.00208 | 2.97E-26 |
| rs1620977 | G | A | 0.6905 | -0.02046 | 0.00195 | 1.14E-25 |
| rs2819336 | T | C | 0.3384 | 0.01828 | 0.00177 | 5.46E-25 |
| rs11678980 | G | A | 0.5544 | 0.01744 | 0.00172 | 4.29E-24 |
| rs10189857 | G | A | 0.4184 | -0.01725 | 0.00171 | 6.70E-24 |
| rs1008078 | T | C | 0.4099 | -0.01738 | 0.00173 | 1.20E-23 |
| rs73344830 | G | A | 0.602 | -0.0172 | 0.00172 | 1.95E-23 |
| rs4787457 | G | A | 0.3146 | -0.01741 | 0.00176 | 3.73E-23 |
| rs1689510 | G | C | 0.6565 | -0.01761 | 0.0018 | 1.40E-22 |
| rs1566085 | T | G | 0.5697 | 0.01645 | 0.00171 | 6.90E-22 |
| rs10963297 | G | C | 0.2517 | 0.01904 | 0.00198 | 7.36E-22 |
| rs1455350 | T | A | 0.5289 | 0.01614 | 0.0017 | 2.61E-21 |
| rs13428598 | T | C | 0.3793 | 0.01649 | 0.00175 | 3.90E-21 |
| rs1391438 | T | C | 0.3146 | 0.0167 | 0.00183 | 5.79E-20 |
| rs79265434 | A | G | 0.8827 | -0.02331 | 0.00262 | 6.08E-19 |
| rs4382592 | T | G | 0.301 | -0.01636 | 0.00185 | 1.01E-18 |
| rs7924036 | T | G | 0.5391 | 0.01501 | 0.0017 | 1.07E-18 |
| rs176218 | G | T | 0.7993 | -0.01883 | 0.00215 | 1.85E-18 |
| rs9616906 | G | A | 0.5765 | -0.01497 | 0.00172 | 2.92E-18 |
| rs6557171 | C | T | 0.7245 | 0.01567 | 0.00181 | 4.15E-18 |
| rs66568921 | G | T | 0.3639 | 0.01565 | 0.00182 | 7.49E-18 |
| rs74998289 | T | G | 0.7602 | 0.01821 | 0.00213 | 1.31E-17 |
| rs35417702 | C | T | 0.4235 | 0.01445 | 0.0017 | 1.93E-17 |
| rs1618725 | T | C | 0.5204 | 0.01477 | 0.00174 | 2.22E-17 |
| rs11601122 | G | A | 0.1497 | -0.01947 | 0.0023 | 2.24E-17 |
| rs10765775 | A | G | 0.3963 | 0.01488 | 0.00176 | 2.62E-17 |
| rs12375949 | T | C | 0.4303 | -0.01447 | 0.00172 | 3.31E-17 |
| rs12028010 | C | T | 0.2228 | -0.01696 | 0.00202 | 4.51E-17 |
| rs62444881 | C | T | 0.8095 | -0.01815 | 0.00217 | 5.79E-17 |
| rs9349956 | A | C | 0.7602 | -0.01881 | 0.00225 | 6.28E-17 |
| rs3897821 | A | G | 0.6497 | 0.01502 | 0.0018 | 8.25E-17 |
| rs2179152 | C | T | 0.6429 | 0.01455 | 0.00176 | 1.21E-16 |
| rs12643771 | C | T | 0.6888 | -0.01518 | 0.00184 | 1.61E-16 |
| rs55736314 | C | G | 0.5833 | -0.01431 | 0.00174 | 1.63E-16 |
| rs2725370 | T | C | 0.2891 | -0.01536 | 0.00187 | 1.97E-16 |
| rs12468040 | G | T | 0.6037 | -0.01432 | 0.00175 | 2.46E-16 |
| rs72828517 | C | T | 0.1412 | 0.01836 | 0.00224 | 2.83E-16 |
| rs406413 | A | T | 0.7891 | 0.01695 | 0.00209 | 4.84E-16 |
| rs10875121 | G | C | 0.1429 | -0.01834 | 0.00226 | 5.53E-16 |
| rs1964927 | G | A | 0.6378 | -0.01423 | 0.00177 | 9.90E-16 |
| rs8020034 | G | A | 0.7942 | -0.01782 | 0.00223 | 1.17E-15 |
| rs2971970 | T | G | 0.2279 | -0.01654 | 0.00207 | 1.25E-15 |
| rs62184480 | T | C | 0.2449 | -0.01528 | 0.00191 | 1.28E-15 |
| rs56391344 | A | G | 0.2381 | 0.01571 | 0.00197 | 1.34E-15 |
| rs16846463 | A | G | 0.8827 | 0.02256 | 0.00283 | 1.38E-15 |
| rs6493265 | C | T | 0.6105 | 0.01385 | 0.00174 | 1.70E-15 |
| rs363096 | C | T | 0.5748 | 0.01363 | 0.00172 | 2.04E-15 |
| rs13141210 | C | T | 0.4915 | -0.01361 | 0.00172 | 2.26E-15 |
| rs2545798 | T | A | 0.5051 | 0.01346 | 0.00171 | 3.11E-15 |
| rs55771711 | C | G | 0.2279 | 0.01555 | 0.00199 | 5.41E-15 |
| rs2347526 | T | C | 0.3622 | -0.01395 | 0.00179 | 6.84E-15 |
| rs34853711 | C | G | 0.2466 | -0.01596 | 0.00206 | 9.88E-15 |
| rs3026996 | A | C | 0.716 | 0.01537 | 0.00199 | 1.05E-14 |
| rs35309068 | G | T | 0.466 | 0.01321 | 0.00171 | 1.15E-14 |
| rs1569092 | A | G | 0.182 | 0.01807 | 0.00234 | 1.16E-14 |
| rs613872 | G | T | 0.1718 | 0.0175 | 0.00227 | 1.20E-14 |
| rs17489649 | A | G | 0.6735 | 0.0139 | 0.00181 | 1.57E-14 |
| rs4757957 | C | G | 0.6514 | 0.0141 | 0.00184 | 1.81E-14 |
| rs72807818 | G | A | 0.8759 | -0.01915 | 0.00252 | 2.79E-14 |
| rs115000530 | T | A | 0.06122 | 0.02892 | 0.00381 | 3.30E-14 |
| rs10215082 | A | G | 0.4388 | -0.01303 | 0.00172 | 3.33E-14 |
| rs76076331 | C | T | 0.869 | -0.01873 | 0.00248 | 4.40E-14 |
| rs62097985 | C | T | 0.5884 | 0.01288 | 0.00172 | 6.06E-14 |
| rs2998315 | G | A | 0.5782 | 0.01269 | 0.00171 | 1.12E-13 |
| rs11657342 | A | G | 0.3554 | 0.01404 | 0.00191 | 1.94E-13 |
| rs790647 | C | A | 0.7653 | 0.01482 | 0.00202 | 2.17E-13 |
| rs12134151 | C | G | 0.5221 | -0.01245 | 0.0017 | 2.42E-13 |
| rs36083520 | C | T | 0.1667 | 0.01629 | 0.00223 | 2.60E-13 |
| rs4810227 | A | G | 0.6344 | 0.01272 | 0.00175 | 3.57E-13 |
| rs35475880 | G | T | 0.8163 | 0.01511 | 0.00208 | 3.80E-13 |
| rs4073894 | A | G | 0.1769 | 0.01524 | 0.00211 | 5.40E-13 |
| rs59123361 | A | G | 0.1054 | -0.02094 | 0.00291 | 5.87E-13 |
| rs7863447 | G | A | 0.1667 | -0.01678 | 0.00233 | 5.91E-13 |
| rs4726070 | G | A | 0.3793 | -0.01251 | 0.00174 | 5.95E-13 |
| rs17425572 | G | A | 0.5425 | -0.01224 | 0.0017 | 6.89E-13 |
| rs79269403 | G | A | 0.7772 | -0.01447 | 0.00204 | 1.17E-12 |
| rs2067854 | A | G | 0.182 | 0.01477 | 0.00209 | 1.38E-12 |
| rs320693 | C | G | 0.4728 | 0.01204 | 0.0017 | 1.58E-12 |
| rs13422673 | C | T | 0.5153 | 0.01201 | 0.0017 | 1.74E-12 |
| rs17598675 | C | T | 0.5187 | 0.01199 | 0.0017 | 1.75E-12 |
| rs11081529 | T | C | 0.7432 | 0.01311 | 0.00186 | 1.82E-12 |
| rs11871429 | A | G | 0.7959 | 0.01425 | 0.00202 | 1.92E-12 |
| rs1584469 | T | C | 0.3112 | -0.01303 | 0.00185 | 2.10E-12 |
| rs35532491 | T | A | 0.1071 | 0.02007 | 0.00286 | 2.42E-12 |
| rs17563464 | A | C | 0.2041 | -0.01477 | 0.00212 | 2.89E-12 |
| rs77702622 | A | G | 0.07653 | -0.02447 | 0.00351 | 2.99E-12 |
| rs2570497 | C | T | 0.3265 | 0.01233 | 0.00177 | 3.03E-12 |
| rs6805241 | C | T | 0.1973 | -0.01413 | 0.00203 | 3.09E-12 |
| rs969512 | T | A | 0.2959 | 0.01249 | 0.00179 | 3.22E-12 |
| rs6731373 | G | A | 0.6633 | 0.01256 | 0.00181 | 3.47E-12 |
| rs11635092 | A | G | 0.3639 | -0.01231 | 0.00177 | 3.89E-12 |
| rs6867851 | C | G | 0.4235 | -0.012 | 0.00173 | 3.97E-12 |
| rs7920624 | T | A | 0.4932 | -0.01181 | 0.0017 | 3.97E-12 |
| rs1051474 | T | C | 0.7262 | -0.01301 | 0.00188 | 4.86E-12 |
| rs575113 | A | G | 0.2772 | 0.01285 | 0.00186 | 5.30E-12 |
| rs4352658 | T | C | 0.09014 | -0.0212 | 0.00308 | 5.55E-12 |
| rs9936270 | T | C | 0.3078 | -0.0136 | 0.00198 | 6.43E-12 |
| rs9882532 | C | T | 0.3639 | -0.01208 | 0.00177 | 8.17E-12 |
| rs76878669 | C | G | 0.7466 | 0.01399 | 0.00205 | 8.67E-12 |
| rs113520408 | A | G | 0.2857 | 0.01304 | 0.00192 | 1.02E-11 |
| rs1882273 | G | C | 0.6531 | 0.01231 | 0.00181 | 1.09E-11 |
| rs175325 | A | T | 0.5816 | -0.01179 | 0.00174 | 1.11E-11 |
| rs35039375 | G | A | 0.09354 | -0.01983 | 0.00293 | 1.22E-11 |
| rs301800 | T | C | 0.1803 | 0.01516 | 0.00224 | 1.33E-11 |
| rs10862376 | T | A | 0.8639 | -0.01616 | 0.00239 | 1.40E-11 |
| rs2287838 | A | G | 0.534 | -0.01152 | 0.00171 | 1.53E-11 |
| rs401687 | G | C | 0.5442 | -0.01144 | 0.0017 | 1.86E-11 |
| rs1106090 | A | G | 0.6259 | 0.01173 | 0.00175 | 2.09E-11 |
| rs7977614 | G | A | 0.3078 | 0.01325 | 0.00198 | 2.09E-11 |
| rs12602286 | T | G | 0.8861 | 0.01701 | 0.00255 | 2.37E-11 |
| rs16995054 | C | T | 0.7993 | 0.0139 | 0.00208 | 2.52E-11 |
| rs17565975 | A | G | 0.5306 | -0.01142 | 0.00171 | 2.56E-11 |
| rs3788556 | T | C | 0.4592 | 0.01138 | 0.00171 | 2.78E-11 |
| rs7993663 | T | C | 0.6429 | -0.0118 | 0.00178 | 3.25E-11 |
| rs17048855 | A | G | 0.3248 | 0.01184 | 0.00179 | 3.27E-11 |
| rs10456918 | A | C | 0.818 | -0.01485 | 0.00224 | 3.67E-11 |
| rs10240905 | T | C | 0.3316 | -0.01167 | 0.00177 | 3.79E-11 |
| rs9938678 | A | T | 0.7568 | -0.01355 | 0.00205 | 4.12E-11 |
| rs1143770 | C | T | 0.4082 | -0.01136 | 0.00172 | 4.31E-11 |
| rs6803651 | G | T | 0.585 | -0.01131 | 0.00172 | 4.36E-11 |
| rs9933256 | A | G | 0.5918 | 0.01134 | 0.00172 | 4.57E-11 |
| rs11694904 | T | C | 0.3384 | 0.01215 | 0.00185 | 4.78E-11 |
| rs28513670 | G | A | 0.1531 | 0.01477 | 0.00225 | 5.06E-11 |
| rs8008382 | T | C | 0.3129 | -0.01208 | 0.00185 | 6.12E-11 |
| rs7233920 | A | G | 0.216 | -0.01315 | 0.00202 | 7.13E-11 |
| rs9436866 | C | A | 0.09524 | 0.01882 | 0.00289 | 7.45E-11 |
| rs6123924 | A | G | 0.8401 | 0.01528 | 0.00235 | 7.55E-11 |
| rs17551064 | G | A | 0.1599 | -0.01493 | 0.0023 | 8.62E-11 |
| rs11732657 | A | G | 0.7007 | -0.01274 | 0.00197 | 9.54E-11 |
| rs2923431 | C | G | 0.6446 | 0.0114 | 0.00176 | 9.84E-11 |
| rs2302761 | T | C | 0.1905 | 0.01354 | 0.00209 | 1.00E-10 |
| rs9289300 | T | C | 0.8163 | -0.01512 | 0.00234 | 1.10E-10 |
| rs10073890 | G | A | 0.7364 | -0.01262 | 0.00196 | 1.11E-10 |
| rs2052285 | A | G | 0.5765 | 0.01123 | 0.00175 | 1.34E-10 |
| rs192436652 | C | T | 0.97789 | 0.03497 | 0.00545 | 1.35E-10 |
| rs9342482 | T | G | 0.2908 | 0.01264 | 0.00197 | 1.36E-10 |
| rs837080 | C | T | 0.4932 | 0.01092 | 0.0017 | 1.43E-10 |
| rs2256965 | G | A | 0.5425 | -0.01128 | 0.00176 | 1.59E-10 |
| rs11663602 | A | C | 0.2568 | -0.01213 | 0.0019 | 1.64E-10 |
| rs2447535 | A | G | 0.2755 | -0.01181 | 0.00185 | 1.69E-10 |
| rs4972400 | G | A | 0.648 | -0.01156 | 0.00181 | 1.70E-10 |
| rs1291818 | C | T | 0.5153 | -0.01085 | 0.0017 | 1.78E-10 |
| rs466047 | A | T | 0.5 | 0.01085 | 0.0017 | 1.80E-10 |
| rs79523955 | A | G | 0.90476 | 0.01802 | 0.00283 | 1.87E-10 |
| rs6513959 | A | G | 0.7211 | 0.01177 | 0.00185 | 1.88E-10 |
| rs67890737 | C | A | 0.6735 | 0.01141 | 0.00179 | 2.01E-10 |
| rs736282 | C | T | 0.5153 | -0.01082 | 0.0017 | 2.07E-10 |
| rs337637 | G | A | 0.6633 | -0.01123 | 0.00177 | 2.11E-10 |
| rs9529119 | C | G | 0.1973 | 0.01295 | 0.00204 | 2.13E-10 |
| rs10887801 | G | T | 0.5629 | -0.01087 | 0.00171 | 2.27E-10 |
| rs9556958 | C | T | 0.4711 | 0.0108 | 0.0017 | 2.38E-10 |
| rs7803932 | A | G | 0.1565 | 0.0143 | 0.00226 | 2.44E-10 |
| rs77719387 | A | T | 0.01701 | -0.04597 | 0.00726 | 2.46E-10 |
| rs4384309 | G | A | 0.5204 | -0.0109 | 0.00172 | 2.52E-10 |
| rs11023749 | A | G | 0.6701 | 0.01132 | 0.0018 | 2.96E-10 |
| rs1558727 | C | T | 0.5153 | 0.01069 | 0.0017 | 3.09E-10 |
| rs76608582 | C | A | 0.95918 | -0.02798 | 0.00445 | 3.11E-10 |
| rs9503598 | A | G | 0.4388 | 0.01079 | 0.00171 | 3.12E-10 |
| rs12332731 | A | T | 0.2024 | 0.01374 | 0.00218 | 3.12E-10 |
| rs31940 | G | A | 0.8656 | -0.01548 | 0.00246 | 3.24E-10 |
| rs10994777 | G | A | 0.8605 | -0.0146 | 0.00232 | 3.36E-10 |
| rs7796203 | G | A | 0.4745 | 0.01074 | 0.00171 | 3.60E-10 |
| rs7808399 | A | G | 0.4524 | -0.0107 | 0.00171 | 3.78E-10 |
| rs13145650 | C | T | 0.09184 | 0.01918 | 0.00306 | 3.80E-10 |
| rs60483752 | G | C | 0.4184 | -0.01078 | 0.00172 | 3.89E-10 |
| rs4839155 | T | G | 0.75 | 0.01251 | 0.002 | 3.94E-10 |
| rs1827540 | G | A | 0.466 | -0.0106 | 0.0017 | 4.50E-10 |
| rs13010566 | A | C | 0.4388 | -0.0106 | 0.0017 | 4.59E-10 |
| rs36119825 | G | A | 0.5306 | -0.01063 | 0.00171 | 4.82E-10 |
| rs2478208 | C | G | 0.5 | -0.0106 | 0.0017 | 4.82E-10 |
| rs10460095 | G | A | 0.4133 | 0.01066 | 0.00171 | 4.87E-10 |
| rs34485537 | T | C | 0.3895 | 0.01075 | 0.00173 | 5.67E-10 |
| rs73301698 | A | G | 0.2262 | -0.01291 | 0.00208 | 5.81E-10 |
| rs892612 | C | A | 0.8418 | 0.01464 | 0.00237 | 6.63E-10 |
| rs10940921 | G | T | 0.5697 | -0.01089 | 0.00177 | 7.00E-10 |
| rs13029509 | A | G | 0.4677 | -0.01049 | 0.0017 | 7.17E-10 |
| rs10205801 | A | G | 0.5068 | -0.01053 | 0.00171 | 7.17E-10 |
| rs17126938 | T | C | 0.8793 | -0.01536 | 0.0025 | 8.14E-10 |
| rs112806496 | G | C | 0.08503 | 0.0187 | 0.00305 | 8.28E-10 |
| rs12574281 | A | C | 0.6003 | -0.01077 | 0.00176 | 8.85E-10 |
| rs9914918 | G | A | 0.7177 | -0.01155 | 0.00189 | 8.90E-10 |
| rs7603132 | G | A | 0.8452 | -0.01317 | 0.00215 | 9.17E-10 |
| rs2820314 | A | C | 0.6837 | 0.011 | 0.0018 | 9.34E-10 |
| rs4328757 | T | C | 0.6514 | 0.01067 | 0.00174 | 9.39E-10 |
| rs3800546 | G | C | 0.2704 | -0.01183 | 0.00194 | 9.73E-10 |
| rs1363862 | G | A | 0.7398 | 0.01171 | 0.00192 | 1.02E-09 |
| rs11222609 | G | A | 0.335 | -0.01084 | 0.00178 | 1.05E-09 |
| rs28373063 | G | C | 0.7925 | -0.01389 | 0.00229 | 1.23E-09 |
| rs7257460 | T | C | 0.7296 | 0.01145 | 0.00189 | 1.25E-09 |
| rs7332724 | T | C | 0.2619 | -0.01149 | 0.00189 | 1.26E-09 |
| rs7031698 | C | T | 0.7755 | 0.01248 | 0.00206 | 1.26E-09 |
| rs9679654 | T | C | 0.5153 | -0.01042 | 0.00172 | 1.29E-09 |
| rs1582173 | C | T | 0.2602 | -0.01189 | 0.00196 | 1.33E-09 |
| rs77025239 | A | G | 0.1088 | -0.01422 | 0.00234 | 1.33E-09 |
| rs62103084 | T | C | 0.1429 | 0.01693 | 0.0028 | 1.42E-09 |
| rs67885444 | T | C | 0.1718 | 0.01406 | 0.00232 | 1.48E-09 |
| rs6122735 | T | C | 0.4133 | 0.0105 | 0.00174 | 1.49E-09 |
| rs4442732 | G | A | 0.5969 | -0.01063 | 0.00176 | 1.49E-09 |
| rs10772644 | G | C | 0.1071 | -0.01614 | 0.00267 | 1.50E-09 |
| rs35316276 | C | T | 0.7058 | -0.01173 | 0.00194 | 1.52E-09 |
| rs12519073 | C | T | 0.7619 | 0.01221 | 0.00202 | 1.60E-09 |
| rs9704097 | A | C | 0.4728 | -0.0103 | 0.00171 | 1.61E-09 |
| rs60904894 | A | C | 0.5 | -0.01025 | 0.0017 | 1.62E-09 |
| rs62183776 | T | C | 0.1905 | -0.01308 | 0.00217 | 1.65E-09 |
| rs1105307 | A | G | 0.2449 | -0.01173 | 0.00195 | 1.67E-09 |
| rs6959891 | G | A | 0.2959 | -0.01136 | 0.00189 | 1.74E-09 |
| rs74643044 | T | C | 0.97279 | -0.02323 | 0.00386 | 1.80E-09 |
| rs2885198 | A | G | 0.4983 | 0.01025 | 0.0017 | 1.81E-09 |
| rs80171383 | A | G | 0.1241 | 0.0145 | 0.00241 | 1.83E-09 |
| rs1671770 | C | A | 0.8061 | -0.01342 | 0.00223 | 1.91E-09 |
| rs62157915 | T | C | 0.94048 | -0.02091 | 0.00348 | 1.96E-09 |
| rs242093 | A | G | 0.5476 | -0.01031 | 0.00172 | 2.07E-09 |
| rs10760023 | C | G | 0.6684 | -0.01095 | 0.00183 | 2.12E-09 |
| rs12503522 | C | T | 0.7517 | 0.01125 | 0.00188 | 2.24E-09 |
| rs4846724 | G | A | 0.5085 | -0.01018 | 0.0017 | 2.26E-09 |
| rs78721320 | G | A | 0.7959 | -0.01307 | 0.00219 | 2.28E-09 |
| rs6731967 | C | G | 0.2092 | -0.01186 | 0.00199 | 2.36E-09 |
| rs4778058 | C | T | 0.5221 | 0.01017 | 0.0017 | 2.40E-09 |
| rs11772580 | G | T | 0.7466 | 0.01199 | 0.00201 | 2.57E-09 |
| rs510706 | G | C | 0.3844 | -0.01071 | 0.0018 | 2.73E-09 |
| rs894067 | G | A | 0.6071 | -0.01041 | 0.00175 | 2.74E-09 |
| rs3890802 | A | G | 0.2687 | -0.01133 | 0.00191 | 2.74E-09 |
| rs115454970 | T | G | 0.3044 | -0.01185 | 0.00199 | 2.76E-09 |
| rs4984541 | A | G | 0.7585 | -0.01233 | 0.00207 | 2.77E-09 |
| rs117799466 | G | C | 0.6224 | -0.01173 | 0.00198 | 2.91E-09 |
| rs3013014 | G | A | 0.3895 | 0.01024 | 0.00172 | 2.92E-09 |
| rs710629 | A | G | 0.6565 | 0.01053 | 0.00177 | 2.96E-09 |
| rs730384 | G | A | 0.5442 | -0.01016 | 0.00171 | 3.01E-09 |
| rs71646142 | C | T | 0.8265 | -0.01286 | 0.00217 | 3.11E-09 |
| rs1427298 | C | T | 0.5884 | -0.0102 | 0.00172 | 3.28E-09 |
| rs4964046 | G | A | 0.335 | 0.01053 | 0.00178 | 3.36E-09 |
| rs117468730 | G | A | 0.9881 | 0.03521 | 0.00597 | 3.79E-09 |
| rs1866823 | A | G | 0.551 | 0.01009 | 0.00171 | 3.81E-09 |
| rs10856785 | T | C | 0.7296 | -0.01132 | 0.00192 | 3.83E-09 |
| rs113615161 | T | C | 0.1395 | -0.01472 | 0.0025 | 3.97E-09 |
| rs2016392 | A | G | 0.2296 | -0.01215 | 0.00207 | 4.35E-09 |
| rs2414072 | A | T | 0.4864 | -0.01005 | 0.00171 | 4.35E-09 |
| rs13130765 | C | G | 0.4558 | -0.01014 | 0.00173 | 4.69E-09 |
| rs17110109 | C | T | 0.3776 | 0.01023 | 0.00175 | 4.71E-09 |
| rs11620355 | G | A | 0.8844 | -0.01756 | 0.003 | 4.77E-09 |
| rs12647336 | A | G | 0.8571 | -0.01375 | 0.00235 | 4.82E-09 |
| rs111821073 | C | T | 0.8367 | -0.01385 | 0.00237 | 4.85E-09 |
| rs7012546 | C | T | 0.5799 | -0.01009 | 0.00172 | 4.93E-09 |
| rs1925576 | A | G | 0.5578 | -0.00997 | 0.00171 | 4.94E-09 |
| rs7833201 | C | G | 0.1344 | -0.01532 | 0.00262 | 5.05E-09 |
| rs6938002 | G | A | 0.6037 | 0.01008 | 0.00173 | 5.41E-09 |
| rs4369924 | A | G | 0.1684 | 0.01362 | 0.00234 | 5.82E-09 |
| rs1592757 | C | G | 0.3759 | -0.01045 | 0.0018 | 5.89E-09 |
| rs12682775 | T | C | 0.7857 | -0.01187 | 0.00204 | 5.99E-09 |
| rs9320493 | G | A | 0.8639 | -0.01394 | 0.0024 | 6.13E-09 |
| rs34394051 | G | A | 0.1599 | 0.01392 | 0.0024 | 6.20E-09 |
| rs1595973 | C | T | 0.4405 | 0.01002 | 0.00173 | 6.56E-09 |
| rs4945424 | A | C | 0.415 | -0.00992 | 0.00171 | 6.94E-09 |
| rs663234 | G | C | 0.6122 | -0.01005 | 0.00174 | 7.39E-09 |
| rs72840994 | G | T | 0.182 | 0.01247 | 0.00216 | 7.77E-09 |
| rs59480703 | G | C | 0.8367 | 0.01237 | 0.00215 | 8.32E-09 |
| rs2905426 | G | T | 0.3554 | -0.01037 | 0.00181 | 9.26E-09 |
| rs77128898 | T | C | 0.02211 | -0.02769 | 0.00482 | 9.47E-09 |
| rs152603 | G | A | 0.3861 | 0.01019 | 0.00177 | 9.47E-09 |
| rs532799 | G | A | 0.6701 | 0.01072 | 0.00188 | 1.08E-08 |
| rs3809634 | A | G | 0.665 | -0.01058 | 0.00185 | 1.09E-08 |
| rs702606 | T | C | 0.8299 | 0.01427 | 0.0025 | 1.12E-08 |
| rs12655753 | A | G | 0.02381 | -0.02903 | 0.00509 | 1.18E-08 |
| rs743316 | T | C | 0.818 | 0.01185 | 0.00208 | 1.20E-08 |
| rs7543462 | A | G | 0.4201 | -0.00973 | 0.00171 | 1.25E-08 |
| rs72486027 | C | T | 0.7211 | 0.01123 | 0.00197 | 1.25E-08 |
| rs4870482 | G | C | 0.2585 | -0.01083 | 0.0019 | 1.27E-08 |
| rs9386319 | G | A | 0.4269 | 0.00991 | 0.00174 | 1.27E-08 |
| rs113182709 | G | A | 0.97619 | -0.03225 | 0.00567 | 1.29E-08 |
| rs2182505 | T | C | 0.2636 | 0.01086 | 0.00192 | 1.64E-08 |
| rs818415 | T | G | 0.818 | -0.01235 | 0.00219 | 1.72E-08 |
| rs225291 | G | A | 0.8027 | -0.01205 | 0.00214 | 1.84E-08 |
| rs622169 | C | T | 0.5323 | -0.00999 | 0.00178 | 1.89E-08 |
| rs9995567 | G | A | 0.6173 | -0.00998 | 0.00178 | 1.93E-08 |
| rs7594904 | C | T | 0.4184 | 0.00969 | 0.00173 | 2.05E-08 |
| rs2283076 | A | G | 0.7857 | 0.01143 | 0.00204 | 2.07E-08 |
| rs7773815 | A | C | 0.4847 | -0.00953 | 0.0017 | 2.08E-08 |
| rs11752914 | T | C | 0.8027 | 0.01208 | 0.00216 | 2.13E-08 |
| rs4895650 | T | C | 0.5357 | 0.00965 | 0.00172 | 2.16E-08 |
| rs62439690 | G | A | 0.733 | 0.01087 | 0.00194 | 2.18E-08 |
| rs74701752 | G | T | 0.90476 | -0.01591 | 0.00285 | 2.38E-08 |
| rs112687095 | G | A | 0.835 | -0.01325 | 0.00238 | 2.42E-08 |
| rs1381247 | C | T | 0.2908 | -0.01013 | 0.00182 | 2.46E-08 |
| rs16854920 | T | C | 0.6446 | -0.01007 | 0.00181 | 2.51E-08 |
| rs7928622 | T | A | 0.3078 | 0.01011 | 0.00181 | 2.52E-08 |
| rs1947114 | G | A | 0.2551 | 0.01071 | 0.00192 | 2.64E-08 |
| rs77835879 | A | G | 0.90986 | 0.01601 | 0.00288 | 2.68E-08 |
| rs2554835 | G | A | 0.602 | -0.00974 | 0.00175 | 2.69E-08 |
| rs277828 | C | A | 0.7432 | 0.01091 | 0.00196 | 2.71E-08 |
| rs11681861 | G | T | 0.1565 | -0.01435 | 0.00259 | 2.88E-08 |
| rs7864396 | C | A | 0.3656 | 0.00979 | 0.00177 | 2.95E-08 |
| rs12804787 | A | G | 0.93197 | 0.01814 | 0.00327 | 2.96E-08 |
| rs795230 | T | C | 0.4184 | 0.00952 | 0.00172 | 2.97E-08 |
| rs73874335 | C | T | 0.94048 | 0.0199 | 0.00361 | 3.40E-08 |
| rs4667029 | G | C | 0.3878 | 0.00961 | 0.00174 | 3.44E-08 |
| rs11627087 | A | G | 0.91497 | 0.01788 | 0.00325 | 3.71E-08 |
| rs12940014 | C | T | 0.5204 | 0.00936 | 0.0017 | 3.73E-08 |
| rs2901616 | A | G | 0.517 | 0.00941 | 0.00171 | 3.77E-08 |
| rs7650602 | T | C | 0.5714 | -0.00939 | 0.00171 | 4.11E-08 |
| rs4888746 | G | A | 0.3776 | -0.00952 | 0.00174 | 4.15E-08 |
| rs7278859 | A | T | 0.6922 | -0.01013 | 0.00185 | 4.15E-08 |
| rs7315713 | A | T | 0.3367 | 0.01022 | 0.00187 | 4.34E-08 |
| rs4766424 | G | C | 0.91156 | -0.0141 | 0.00258 | 4.43E-08 |
| rs4733264 | C | G | 0.631 | -0.00954 | 0.00174 | 4.53E-08 |
| rs535307 | A | G | 0.3231 | 0.01004 | 0.00184 | 4.73E-08 |
| rs9384679 | T | C | 0.4082 | -0.00959 | 0.00176 | 4.88E-08 |
| rs1450782 | G | T | 0.602 | -0.00945 | 0.00173 | 4.93E-08 |
| rs7016302 | G | C | 0.1769 | 0.01243 | 0.00228 | 4.98E-08 |

**Additional file 7. MRlap package**

**Title:** MRlap is an R-package to perform two-sample Mendelian Randomization (MR) analyses using (potentially) overlapping samples

Version 0.0.0.9000

**Description:** MR estimates can be subject to different types of biases due to the overlap between the exposure and outcome samples, the use of weak instruments and Winner’s curse. Our approach simultaneously accounts and corrects for all these biases, using cross-trait LD-score regression (LDSC) to approximate the overlap. It requires only GWAS summary statistics. Estimating the corrected effect using our approach can be performed as a sensitivity analysis: if the corrected effect do not significantly differ from the observed effect, then IVW_MR estimate can be safely used. However, when there is a significant difference, corrected effects should be preferred as they should be less biased, independently of the sample overlap.
